# Aryne-mediated direct assembly of *O*-bridged seven-membered cyclic diaryliodoniums: access to oxaheteropines and cannabinols *via* skeletal modification

**DOI:** 10.1039/d6sc05160j

**Published:** 2026-07-28

**Authors:** Shungo Kamioka, Shunya Morohashi, Jun Kikuchi, Eunsang Kwon, Naohiko Yoshikai

**Affiliations:** a Graduate School of Pharmaceutical Sciences, Tohoku University 6-3 Aoba, Aramaki, Aoba-ku Sendai 980-8578 Japan naohiko.yoshikai.c5@tohoku.ac.jp; b Research and Analytical Center for Giant Molecules, Graduate School of Science, Tohoku University 6-3 Aoba, Aramaki, Aoba-ku Sendai 980-8578 Japan

## Abstract

Cyclic diaryliodonium salts are versatile intermediates for the synthesis of functionalized aromatic compounds and polycyclic systems. Their preparation, however, typically relies on oxidative cyclization of preorganized iodoaryl–aryl frameworks, and medium-sized cyclic diaryliodoniums remain largely underexplored within this synthetic paradigm. Here we report an aryne-mediated direct assembly of *O*-bridged seven-membered cyclic diaryliodoniums from (pseudo)cyclic diaryliodoniums bearing an *O*-nucleophilic benzyl alcohol-derived ligand and a dummy 2,4,6-trimethoxyphenyl ligand. The transformation involves aryne engagement of the oxygen and intramolecular ambiphilic capture by the iodine(iii) center, followed by protonation of the dummy ligand to furnish the cyclic diaryliodonium products. These cyclic diaryliodoniums serve as versatile platforms for skeletal modification, enabling access to oxaheteropine frameworks through iodine replacement and a concise synthesis of cannabinol *via* iodine deletion.

## Abstract 2

A direct annulation of arynes with ambiphilic diaryliodonium reagents provides unprecedented oxygen-bridged seven-membered cyclic diaryliodonium salts. Their skeletal modification enables concise access to oxaheteropines and cannabinoids.

## Graphical abstract

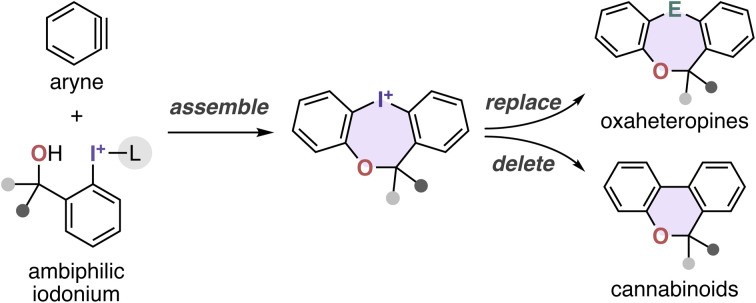

## Introduction

**Scheme 1:**
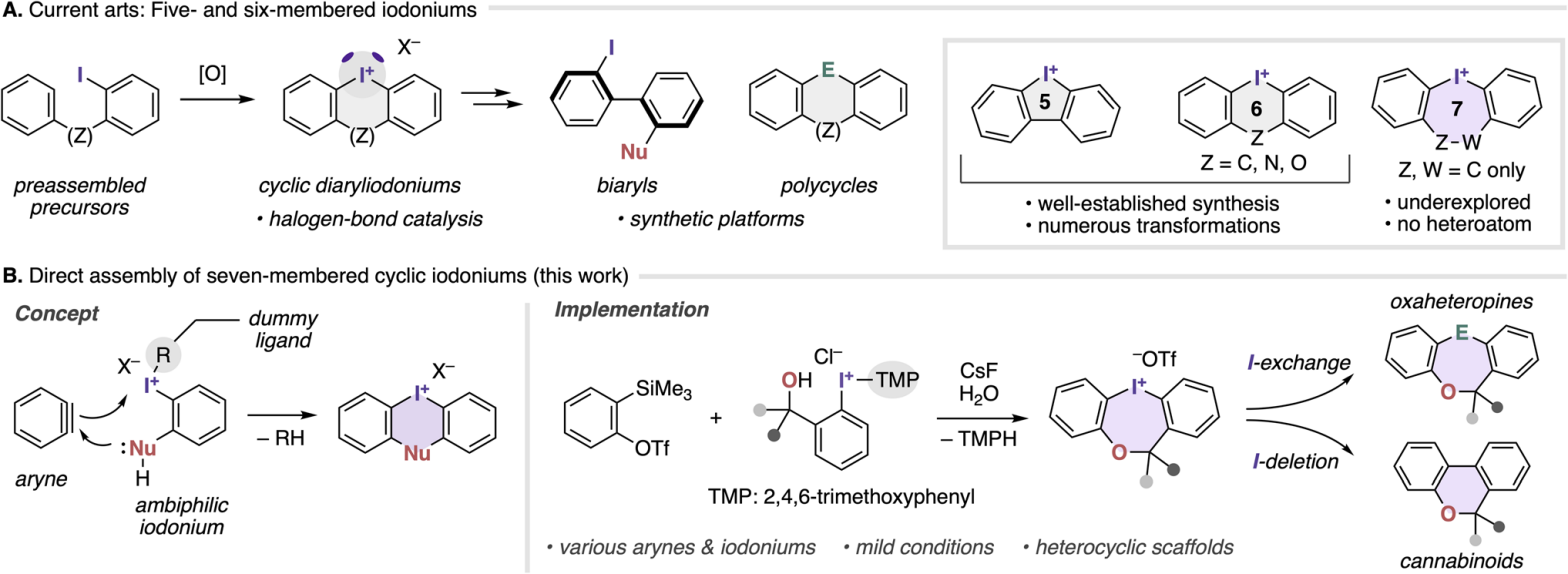
Cyclic diaryliodonium salts: current arts and present approach.

Cyclic diaryliodonium salts have emerged as versatile intermediates in organic synthesis, enabling the preparation of functionalized aromatics and structurally diverse polycyclic frameworks (Scheme 1A).^1–5^ In addition to their synthetic utility as electrophilic aryl-transfer reagents, cyclic diaryliodoniums have also attracted attention in areas such as halogen-bond catalysis^6,7^ and supramolecular chemistry.^8–10^ Accordingly, the development of new cyclic diaryliodonium scaffolds and methods for their construction continues to attract considerable interest. The synthesis of cyclic diaryliodonium salts typically relies on oxidative cyclization of preorganized iodoarene–arene frameworks, in which two aryl units are connected by an appropriate linker prior to oxidation of iodine(i) to iodine(iii).^1–3,11^ This strategy has proven broadly effective and has enabled the preparation of a wide range of five- and six-membered cyclic diaryliodoniums, including those bridged by heteroatoms such as oxygen and nitrogen.

In contrast, seven-membered cyclic diaryliodonium salts remain comparatively rare.^12^ Only a few examples featuring simple carbon-based bridges, such as methylene- or vinylene-linked frameworks, have been reported to date,^13–15^ and the structural diversity of such medium-sized cyclic diaryliodoniums remains extremely limited. Notably, heteroatom-bridged variants analogous to those well established in six-membered systems have not been described, leaving a largely unexplored region of chemical space in cyclic diaryliodonium chemistry. As such, the utility of seven-membered cyclic diaryliodonium salts as building blocks has received far less attention than that of their five- and six-membered congeners.^16^

Here we report an aryne-mediated direct assembly of *O*-bridged seven-membered cyclic diaryliodoniums from (pseudo)cyclic diaryliodoniums bearing an *O*-nucleophilic benzyl alcohol-derived ligand and a 2,4,6-trimethoxyphenyl (TMP) dummy ligand (Scheme 1B).^17,18^ The transformation involves aryne engagement of the oxygen followed by intramolecular ambiphilic capture by the iodine(iii) center, enabling rapid construction of an *O*-bridged seven-membered cyclic diaryliodonium framework. The resulting cyclic diaryliodoniums serve as versatile platforms for skeletal modification, providing access to oxaheteropine frameworks through iodine replacement and enabling a concise synthesis of cannabinol *via* iodine deletion.

## Results and discussion

Motivated by our continuing interest in aryne-mediated transformations of hypervalent iodine compounds,^19–22^ we wondered whether a diaryliodonium salt bearing a pendant oxygen moiety^23,24^ could engage arynes as an ambiphilic reactant.^25,26^ In such a process, nucleophilic addition of the oxygen to the electrophilic aryne would generate a developing aryl anion that could subsequently undergo intramolecular capture at the electrophilic iodine(iii) center. This sequence would enable the direct assembly of oxygen-bridged cyclic triaryliodane frameworks, which, upon protonation of the exocyclic aryl ligand,^27^ would give rise to the corresponding iodonium salts.

**Scheme 2:**
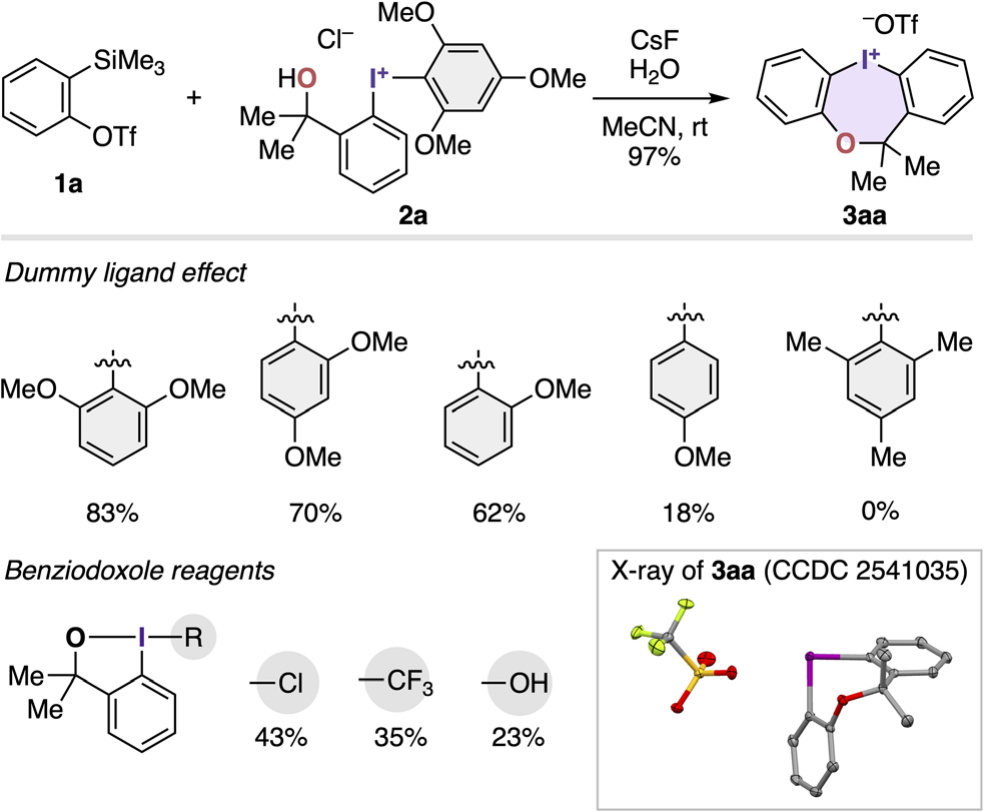
Survey of ambiphilic iodine(iii) reagents for annulation with benzyne. The reaction was performed using 0.20 mmol of 1a, 0.09–0.10 mmol of iodine(iii) reagent (2a or its analogue), 0.40 mmol of CsF, and 0.10 mmol of H_2_O in MeCN (0.5 mL) at room temperature for 18 h.

Based on this hypothesis, we synthesized a series of pseudocyclic diaryliodonium chlorides bearing a dimethylbenzyl alcohol-derived ligand and a dummy aryl ligand (see the SI for the procedure) and examined their reactions toward benzyne generated from *ortho*-silylaryl triflate 1a (Scheme 2; see also Tables S1 and S2 for additional data). Among these salts, the one bearing 2,4,6-trimethoxyphenyl ligand (2a) emerged as a particularly efficient reagent. The reaction of 1a and 2a in the presence of CsF and H_2_O in MeCN proceeded smoothly at room temperature, providing the desired cyclic iodonium salt 3aa in 97% yield. The addition of water (∼1 equiv.) proved crucial for achieving consistent yields. In the absence of water, the reaction exhibited significant variability depending on the reaction conditions and operator, whereas controlled addition of water (0.5–5 equiv.) ensured reproducible formation of 3aa. The importance of the methoxy substitution became evident from the diminished yields observed with related compounds bearing 2,6-dimethoxyphenyl (83%), 2,4-dimethoxyphenyl (70%), 2-methoxyphenyl (62%), and 4-methoxyphenyl (18%) ligands. Compounds bearing mesityl or phenyl ligands instead produced complex mixtures without detectable formation of 3aa. On the other hand, benziodoxole-type reagents containing chloro or hydroxy ligands, as well as the Togni reagent, also participated in the annulation, albeit in modest yields (23–43%).

The structure of 3aa was confirmed by X-ray crystallographic analysis (Scheme 2, inset), revealing the formation of an oxygen-bridged seven-membered diaryliodonium framework.^28^ Although the seven-membered ring adopts a folded conformation, the C–I–C angle is close to 90° (89.7°), consistent with the characteristic L-shaped geometry of iodine(iii) centers. In five-membered cyclic iodonium structures, the C–I–C angle deviates significantly from this ideal geometry due to severe ring strain (typically *ca.* 81–82°),^29^ whereas six-membered analogues are already capable of accommodating near-orthogonal geometries.^30,31^ The present seven-membered system likewise allows the iodine center to adopt its preferred coordination geometry, as evidenced by the nearly orthogonal arrangement observed in 3aa. The triflate counteranion occupies one of the two σ-holes of the iodine center at a distance of 2.81 Å, while the other σ-hole is engaged by an oxygen atom of another triflate anion in the crystal packing.

**Scheme 3:**
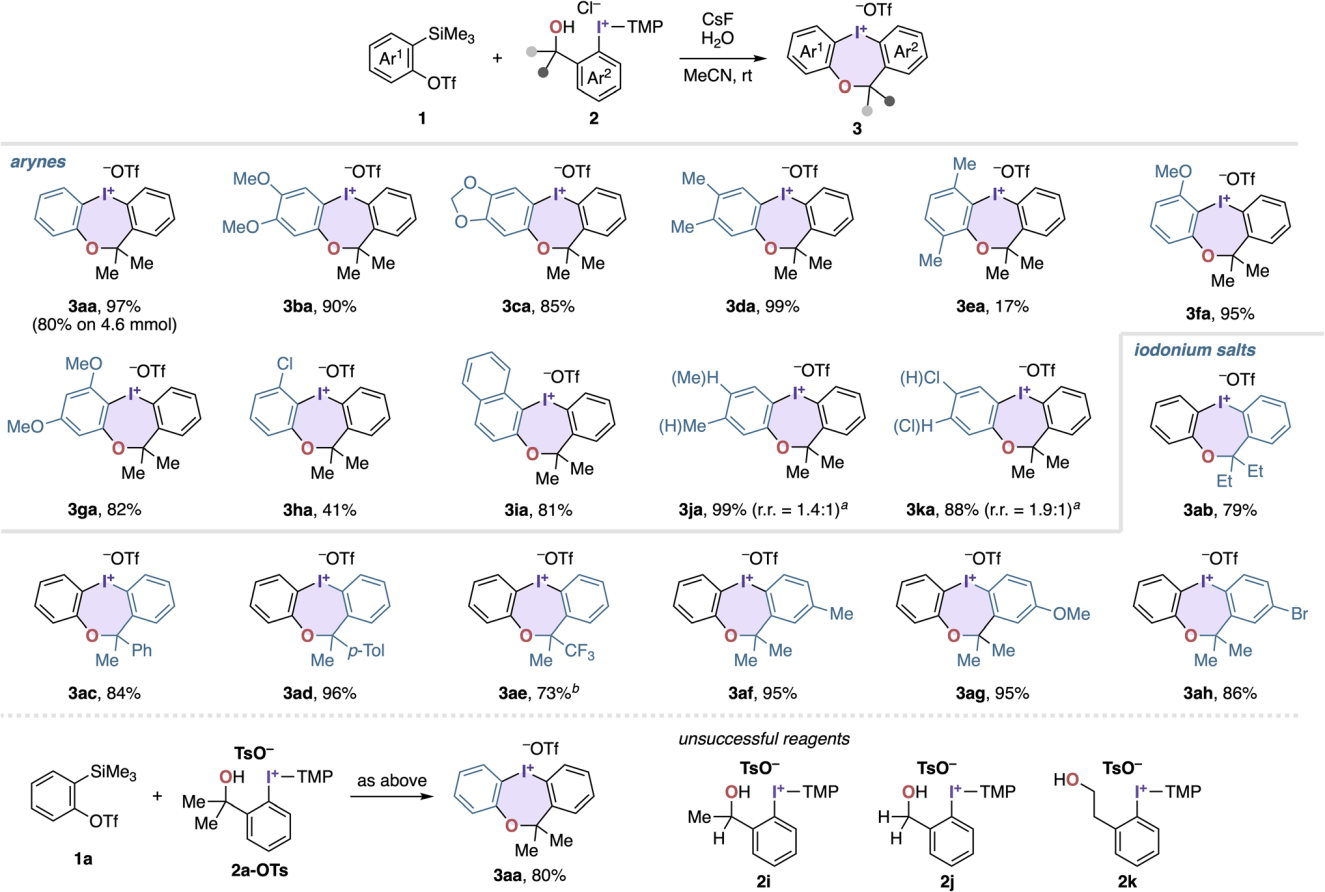
Scope of aryne-mediated assembly of various seven-membered cyclic diaryliodonium salts. TMP = 2,4,6-trimethoxyphenyl. Unless otherwise noted, the reaction was performed on a 0.092 mmol scale. ^*a*^r.r. denotes regioisomer ratio determined by ^1^H NMR analysis. ^*b*^The I(iii) reagent (2e) is a neutral benziodoxole-type compound.

Having identified the optimal dummy ligand and reaction conditions, we next explored the scope of the annulative synthesis of seven-membered iodonium salts (Scheme 3). Symmetric 4,5-disubstituted benzynes underwent smooth annulation to afford the desired products 3ba–3da in high yields. In contrast, 3,6-dimethylbenzyne reacted sluggishly, providing the corresponding iodonium salt 3ea in 17% yield, likely due to increased steric congestion. 3-Methoxybenzyne reacted with complete regioselectivity, delivering iodonium 3fa in 95% yield, with the C–O and C–I bonds formed at the distal and proximal positions, respectively. This regioselectivity can be rationalized by the electronic bias of 3-methoxybenzyne, in which the distal position is more electrophilic,^32^ in conjunction with the complementary roles of the oxygen and iodine centers in reagent 2a. Similarly, 3,5-dimethoxybenzyne and 3-chlorobenzyne underwent annulation with high regioselectivity to furnish 3ga and 3ha as single regioisomers. The reaction of 1,2-naphthalyne also proceeded with exclusive regioselectivity, where C–O bond formation took place at the less hindered position. In contrast, electronically and sterically less biased benzynes such as 4-methylbenzyne and 4-chlorobenzyne exhibited diminished regioselectivity, affording 3ja and 3ka as mixtures of regioisomers. We also examined alternative aryne generation methods using the classical diazonium carboxylate system (generated *in situ* from anthranilic acid and *tert*-BuONO) and a cyclic diarylbromonium salt precursor recently developed by Wencel-Delord and co-workers.^33^ Under the conditions examined, neither system afforded the desired seven-membered cyclic iodonium products, and only complex reaction mixtures were obtained.

We next examined the scope of the iodonium component. The benzylic substituents could be varied flexibly, as demonstrated by products 3ab (Et, Et), 3ac (Me, Ph), 3ad (Me, *p*-tolyl), and 3ae (Me, CF_3_), all obtained in good yields. Furthermore, substitution on the aromatic ring of the benzyl alcohol skeleton was well tolerated, including methyl (3af), methoxy (3ag), and bromo (3ah) groups.

Having established the scope of the annulation, we next sought to improve the accessibility of the pseudocyclic diaryliodonium reagent while probing the structural requirements of the transformation. Whereas the pseudocyclic diaryliodonium chlorides employed above were prepared from the corresponding chlorobenziodoxoles and 2,4,6-trimethoxyphenyllithium, we devised a more convenient route to an analogous tosylate reagent (2a-OTs) through one-pot oxidation/arylation of 2-iodo-α,α-dimethylbenzyl alcohol (see the SI). Gratifyingly, this readily accessible reagent underwent smooth annulation with parent benzyne under the standard conditions to afford 3aa in 80% yield.

We then examined the structural requirements of the pseudocyclic diaryliodonium reagent. In contrast to 2a-OTs, analogous tosylate reagents lacking one or both *gem*-dimethyl substituents (2i and 2j) failed to furnish the desired annulation products, instead giving complex mixtures. Likewise, extending the tether by one methylene unit using a phenethyl alcohol-derived reagent (2k) did not afford the corresponding eight-membered cyclic iodonium salt. These observations indicate that the geminal substituents play a crucial role in preorganizing the hydroxy and iodonium functionalities into a favorable geometry for the annulation (Thorpe–Ingold effect), while also suggesting that simple extension of the tether is insufficient to enable larger-ring formation. Whether incorporation of geminal substituents into the phenethyl alcohol-derived scaffold can overcome this limitation and enable eight-membered ring formation is currently under investigation.

**Scheme 4:**
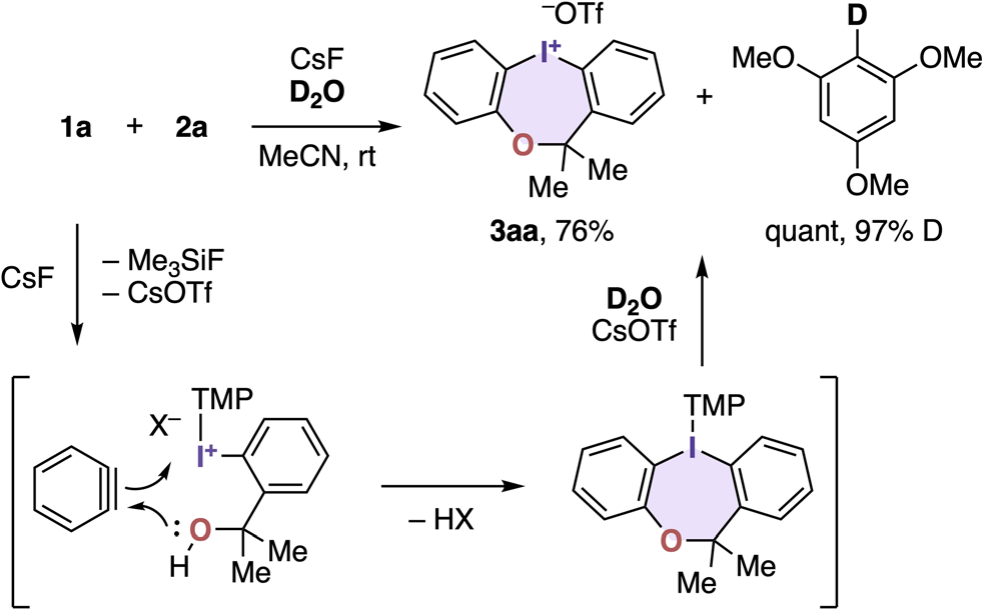
Deuterium-labeling experiments.

Mechanistic insight into the annulation was obtained from deuterium-labeling experiments (Scheme 4). When the reaction was conducted in the presence of D_2_O (5 equiv.), the formation of iodonium salt 3aa was accompanied by quantitative generation of 1,3,5-trimethoxybenzene with a high level of deuterium incorporation. This observation is consistent with, and provides indirect support for, the intermediacy of a cyclic triaryliodane species, followed by facile protonation (or deuteration) of the dummy ligand during the reaction.^27^

**Scheme 5:**
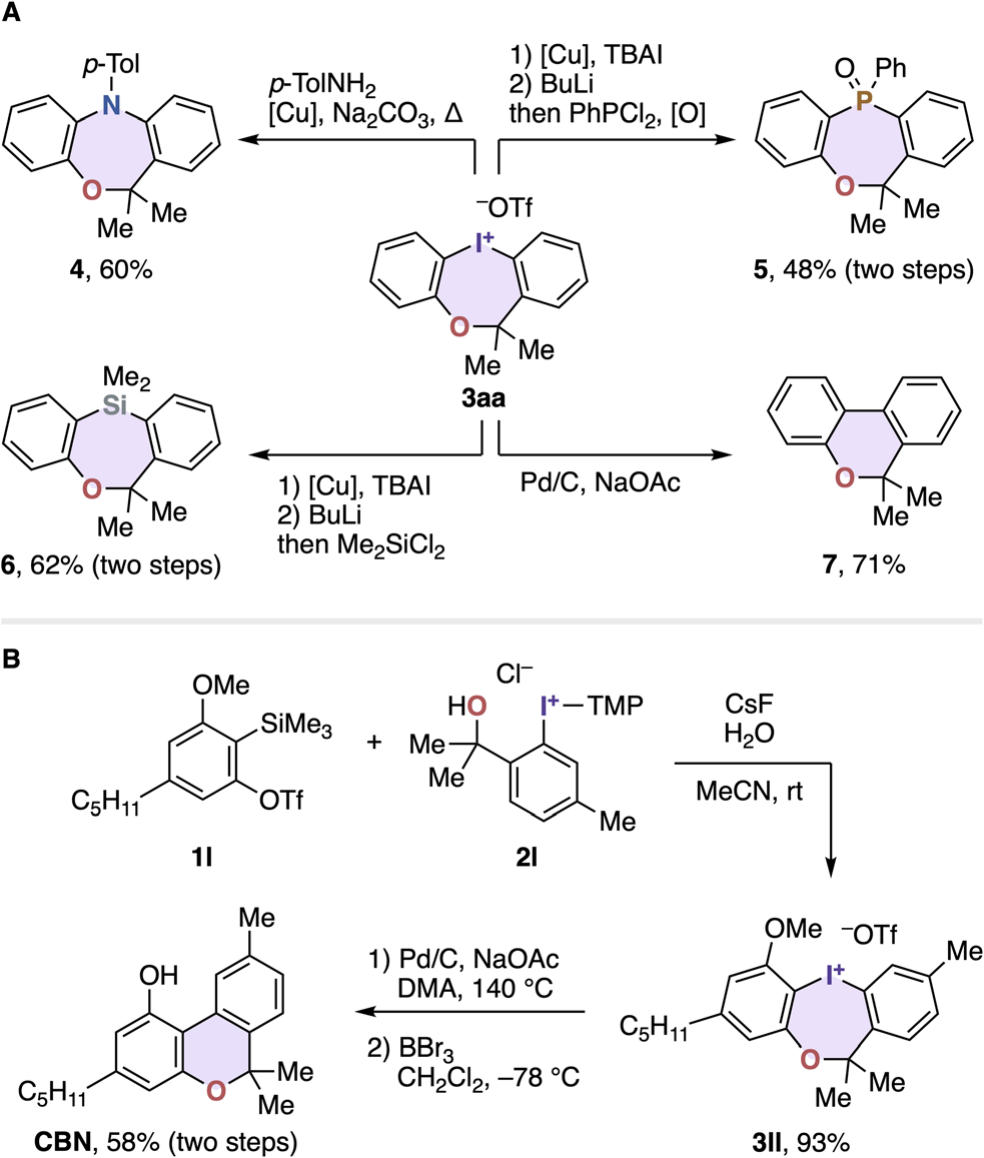
Synthetic applications (see the SI for the detailed reaction conditions): (A) product diversification *via* iodine replacement or deletion. (B) concise synthesis of cannabinol (CBN) *via* aryne/iodonium annulation and iodine deletion.

Scheme 5A illustrates representative transformations of the oxygen-bridged seven-membered iodonium salt 3aa. A copper-catalyzed double C–N coupling with *p*-toluidine^34^ furnished dihydrodibenzo[*b*,*e*][1,4]oxazepane derivative 4 in 60% yield. Alternatively, 3aa underwent copper-catalyzed iodinative ring-opening to give a linear ether bearing two iodoaryl moieties.^35^ Subsequent double iodine–lithium exchange, followed by electrophilic trapping with dichlorophenylphosphine or dichlorodimethylsilane, afforded the oxaphosphepine derivative 5 and the oxasilepine derivative 6, respectively. These iodine-to-heteroatom exchange processes highlight the utility of the present iodonium salts as versatile building blocks for medium-sized oxygen heterocycles that remain relatively underexplored. Furthermore, the iodine bridge could be removed by treatment of 3aa with Pd/C in the presence of NaOAc under heating conditions (DMA, 140 °C),^31,36^ furnishing the benzo[*c*]chromene derivative 7 in 71% yield. A one-pot annulation–deiodination sequence was also feasible, directly providing the benzo[*c*]chromene framework without isolation of the iodonium salt (see the SI for details).

The successful iodine deletion of 3aa and the resulting benzo[*c*]chromene framework highlight the potential of the present annulation as a platform for the convergent and modular synthesis of cannabinol (CBN) derivatives, related benzo[*c*]chromene-based natural products, and their structurally modified analogues.^37–39^ Importantly, the annulation proceeds with complete regioselectivity with 3-methoxyarynes (see 3fa and 3ga in Scheme 3), which is essential for establishing the correct substitution pattern of the CBN framework. To demonstrate this potential, we carried out a concise synthesis of the parent CBN (Scheme 5B).^40–44^ Aryne precursor 1l and iodonium salt 2l, each prepared in several steps from known precursors (see the SI), were subjected to the standard conditions to furnish the corresponding seven-membered iodonium salt 3ll in 93% yield. Subsequent Pd-catalyzed iodine deletion, followed by demethylation with BBr_3_, afforded CBN in 58% yield.

## Conclusions

In summary, we have developed a direct annulative strategy for the synthesis of oxygen-bridged seven-membered iodonium salts *via* the reaction of arynes with ambiphilic diaryliodonium reagents featuring hydroxy-appended aryl and dummy aryl ligands. The method enables efficient construction of structurally unique medium-sized iodonium frameworks under mild conditions with broad substrate scope. Mechanistic studies support the intermediacy of a cyclic triaryliodane species, followed by protonation of the dummy ligand. The resulting iodonium salts serve as versatile synthetic intermediates, undergoing diverse transformations including heteroatom incorporation and reductive iodine deletion. The utility of this strategy was further demonstrated by a concise synthesis of cannabinol (CBN). Importantly, the convergent nature of the annulation allows modular combination of aryne precursors and iodonium reagents, enabling rapid access to diverse medium-sized ring systems from readily accessible building blocks. Future studies will focus on extending the present annulative strategy to nitrogen- and sulfur-bridged cyclic iodoniums through the development of the corresponding ambiphilic iodine(iii) reagents.

## Author contributions

Shungo Kamioka: investigation; data curation. Shunya Morohashi: investigation. Jun Kikuchi: formal analysis; writing – review and editing. Eunsang Kwon: investigation; formal analysis. Naohiko Yoshikai: conceptualization; writing – original draft; writing – review and editing; supervision; project administration; funding acquisition.

## Conflicts of interest

There are no conflicts to declare.

## Supplementary Material

SC-OLF-D6SC05160J-s001

SC-OLF-D6SC05160J-s002

## Data Availability

CCDC 2541021 (2a), 2541032 (2e), and 2541035 (3aa) contain the supplementary crystallographic data for this paper.^45*a*–*c*^ The data supporting this article have been included as part of the supplementary information (SI). Supplementary information is available. See DOI: https://doi.org/10.1039/d6sc05160j.

## References

[cit1] Peng X., Rahim A., Peng W., Jiang F., Gu Z., Wen S. (2023). Chem. Rev..

[cit2] Singhal R., Choudhary S. P., Malik B., Pilania M. (2023). Org. Biomol. Chem..

[cit3] Wang Y., An G., Wang L., Han J. (2020). Curr. Org. Chem..

[cit4] Cheng H.-C., Ma J.-L., Guo P.-H. (2023). Adv. Synth. Catal..

[cit5] Singh P. R., Banerjee A., Simlandy A. K. (2025). ACS Catal..

[cit6] Robidas R., Reinhard D. L., Legault C. Y., Huber S. M. (2021). Chem. Rec..

[cit7] Jovanovic D., Mohanan M. P., Huber S. M. (2024). Angew. Chem., Int. Ed..

[cit8] Stang P. J., Zhdankin V. V. (1993). J. Am. Chem. Soc..

[cit9] Radhakrishnan U., Stang P. J. (2003). J. Org. Chem..

[cit10] Chen W. W., Artigues M., Font-Bardia M., Cuenca A. B., Shafir A. (2023). J. Am. Chem. Soc..

[cit11] Jiao S., Hamza K., Wang L., Han J. (2025). Chin. J. Org. Chem..

[cit12] Grushin V. V. (2000). Chem. Soc. Rev..

[cit13] Collette J., McGreer D., Crawford R., Chubb F., Sandin R. B. (1956). J. Am. Chem. Soc..

[cit14] Nesmeyanov A. N., Tolstaya T. P., Vanchikova L. N., Petrakov A. V. (1980). Bull. Acad. Sci. USSR.

[cit15] Tolstaya T. P., Sukhomlinova L. I., Vanchikov A. N., Bumagin N. A. (1999). Chem. Heterocycl. Compd..

[cit16] Luo B., Cui Q., Luo H., Hu Y., Huang P., Wen S. (2016). Adv. Synth. Catal..

[cit17] Seidl T. L., Sundalam S. K., McCullough B., Stuart D. R. (2016). J. Org. Chem..

[cit18] Lindstedt E., Reitti M., Olofsson B. (2017). J. Org. Chem..

[cit19] Arakawa C., Kanemoto K., Nakai K., Wang C., Morohashi S., Kwon E., Ito S., Yoshikai N. (2024). J. Am. Chem. Soc..

[cit20] Kanemoto K., Yoshimura K., Ono K., Ding W., Ito S., Yoshikai N. (2024). Chem. Eur. J..

[cit21] Otsuki S., Kanemoto K., Martos D. C., Kwon E., Wencel-Delord J., Yoshikai N. (2025). Chem. Sci..

[cit22] Morohashi S., Zhou L., Kanemoto K., Kwon E., Yoshikai N. (2025). Org. Lett..

[cit23] Merritt E. A., Olofsson B. (2009). Angew. Chem., Int. Ed..

[cit24] Chu L., Wang L., Han J. (2025). Adv. Synth. Catal..

[cit25] Shi J., Li L., Li Y. (2021). Chem. Rev..

[cit26] Kim N., Choi M., Suh S.-E., Chenoweth D. M. (2024). Chem. Rev..

[cit27] Beringer F. M., Chang L. L. (1971). J. Org. Chem..

[cit28] CCDC 2541021 (**2a**), 2541032 (**2e**), and 2541035 (**3aa**) contain the supplementary crystallographic data for this paper. These data are provided free of charge by the joint Cambridge Crystallographic Data Center and Fachinformationszentrum Karlsruhe Access Structures Service, https://www.ccdc.cam.ac.uk/structures

[cit29] Zhao K., Duan L., Xu S., Jiang J., Fu Y., Gu Z. (2018). Chem.

[cit30] Caspers L. D., Spils J., Damrath M., Lork E., Nachtsheim B. J. (2020). J. Org. Chem..

[cit31] Damrath M., Caspers L. D., Duvinage D., Nachtsheim B. J. (2022). Org. Lett..

[cit32] Medina J. M., Mackey J. L., Garg N. K., Houk K. N. (2014). J. Am. Chem. Soc..

[cit33] Lanzi M., Dherbassy Q., Wencel-Delord J. (2021). Angew. Chem., Int. Ed..

[cit34] Zhu D., Liu L. Q., Luo B., Chen M., Pi R., Huang P., Wen S. (2013). Adv. Synth. Catal..

[cit35] Wu B., Yoshikai N. (2015). Angew. Chem., Int. Ed..

[cit36] Panda N., Mattan I., Nayak D. K. (2015). J. Org. Chem..

[cit37] Maioli C., Mattoteia D., Amin H. I. M., Minassi A., Caprioglio D. (2022). Plants.

[cit38] Pratap R., Ram V. J. (2014). Chem. Rev..

[cit39] Kearney S. E., Gangano A. J., Barrus D. G., Rehrauer K. J., Reid T.-E. R., Navaratne P. V., Tracy E. K., Roitberg A., Ghiviriga I., Cunningham C. W., Gamage T., Grenning A. J. (2023). J. Am. Chem. Soc..

[cit40] Nandaluru P. R., Bodwell G. J. (2012). Org. Lett..

[cit41] Li Y., Ding Y. J., Wang J. Y., Su S. Y. M., Wang X. S. (2013). Org. Lett..

[cit42] Norseeda K., Tummatorn J., Krajangsri S., Thongsornkleeb C., Ruchirawat S. (2016). Asian J. Org. Chem..

[cit43] Caprioglio D., Mattoteia D., Minassi A., Pollastro F., Lopatriello A., Munoz E., Taglialatela-Scafati O., Appendino G. (2019). Org. Lett..

[cit44] Kumar S., Nunewar S., Kanchupalli V. (2022). Asian J. Org. Chem..

[cit45] (a) CCDC 2541021: Experimental Crystal Structure Determination, 2026, 10.5517/ccdc.csd.cc2r94fn

