# Supplementary material for: Aryne-mediated direct assembly of O-bridged seven-membered cyclic diaryliodoniums: access to oxaheteropines and cannabinols via skeletal modification
Source: Chem Sci. 2026 Jul 28. Online ahead of print. doi: 10.1039/d6sc05160j (PMC13430731; doi:10.1039/d6sc05160j)

Supplementary Information (SI) for Chemical Science.
This journal is © The Royal Society of Chemistry 2026

Supplementary Information

Aryne-mediated direct assembly of *O*-bridged seven-membered cyclic diaryliodoniums: Access to oxaheteropines and cannabinols via skeletal modification

Shungo Kamioka,[1] Shunya Morohashi,[1] Jun Kikuchi,[1] Eunsang Kwon,[2] and Naohiko Yoshikai[1,*]

[1]*Graduate School of Pharmaceutical Sciences, Tohoku University, 6-3 Aoba, Aramaki, Sendai 980-8578, Japan*

[2]*Research and Analytical Center for Giant Molecules, Graduate School of Science, Tohoku University, Sendai 980-8578, Japan*

E-mail: naohiko.yoshikai.c5@tohoku.ac.jp

Contents

1. Material and Methods S2

2. Preparation of Diaryliodonium Reagents S4

3. Aryne-Mediated Synthesis of Seven-Membered Cyclic Diaryliodoniums S17

4. Transformations of Cyclic Diaryliodonium Salts S29

5. Synthesis of Cannabinol S34

6. Deuterium-Labeling Experiments S39

7. X-Ray Crystallographic Analysis S40

8. References S46

9. NMR Spectra S48

1. Material and Methods

General. All reactions dealing with air- or moisture-sensitive compounds were performed by standard Schlenk techniques in oven-dried reaction vessels under argon. Analytical thin-layer chromatography (TLC) was performed on Merck 60 F254 silica gel plates. Column chromatography was performed using flash chromatography with 40–63 μm silica gel (Silica Gel 60N, Kanto Chemical Co., Inc.). Preparative thin layer chromatography was conducted using a 20×20 cm glass sheet coated with a 1 mm thick layer of silica gel (FUJIFILM Wako Pure Chemical Corporation, Wakogel® B-5F, Cat. No. 230-00043). ^{1}H, ^{13}C{^{1}H} and ^{19}F{^{1}H} nuclear magnetic resonance (NMR) spectra were recorded on a Varian Mercury (400 MHz), a JNM-ECZL400S (400 MHz), or a JEOL-ECA600 (600 MHz) spectrometer. ^{1}H and ^{13}C{^{1}H} NMR spectra were reported in parts per million (ppm) downfieldRfom an internal standard, tetramethylsilane (0.00 ppm for ^{1}H NMR in $CDCl_3$) and the solvent peak (3.31 ppm for ^{1}H NMR in CD_3OD, 77.16 ppm for ^{13}C{^{1}H} NMR in $CDCl_3$, 49.00 ppm for ^{13}C{^{1}H} NMR in CD_3OD), respectively. ^{19}F{^{1}H} NMR spectra are referenced to external standard (CF_3CO_2H, –76.6 ppm in $CDCl_3$). ^{31}P NMR spectra are referenced to external standard (PPh_3, –6.0 ppm in $CDCl_3$). The following abbreviations (or combinations thereof) indicate multiplicities: s = singlet, d = doublet, t = triplet, q = quartet, m = multiplet. Melting points were determined with an MPA100 OptiMelt apparatus. High-resolution mass spectra (HRMS) were recorded on a JEOL JMS-DX-303, a JEOL JMS-700, or a JEOL JMS-T100GC spectrometer with magnetic sector time-of-flight mass analyzer. Gel permeation chromatography (GPC) was conducted with a Recycling Preparative HPLC LaboACE LC-5060P (Japan Analytical Industry, Co. Ltd.).

Materials. Unless otherwise noted, commercial reagents were purchased from Tokyo Chemical Industry Co., Ltd., Kanto Chemical Co., Inc., Signa-Aldrich Japan, FUJIFILM Wako Pure Chemical Corporation, and other commercial suppliers and were used as received. Anhydrous MeCN and 1,4-dioxane (FUJIFILM Wako Pure Chemical Corporation) and anhydrous THF and Et_2O (KANTO Chemical Co., Inc., "Dehydrated Solvent System") were used as received. Figure S1 summarizes the aryne and iodonium substrates used in this study. Aryne precursors **1a**, **1b**, **1f**, **1i**, and **1j** were purchased from commercial suppliers and used without further purification, while precursors **1c**,[1] **1d**,[1] **1e**,[2] **1g**,[3] **1h**,[4] and **1k**[5] were synthesized according to literature procedures. The synthesis of aryne precursor **1l** is described in the section on cannabinol synthesis (Section 5). The synthesis of diaryliodonium or benziodoxole reagents **2a**–**2l** and **2a-OTs** is described in the next section (Section 2). Known benziodoxole

reagents bearing chloro,[6] bromo,[7] trifluoromethyl,[8] hydroxy,[9] and acetoxy[9] ligands were synthesized as described in the literature.

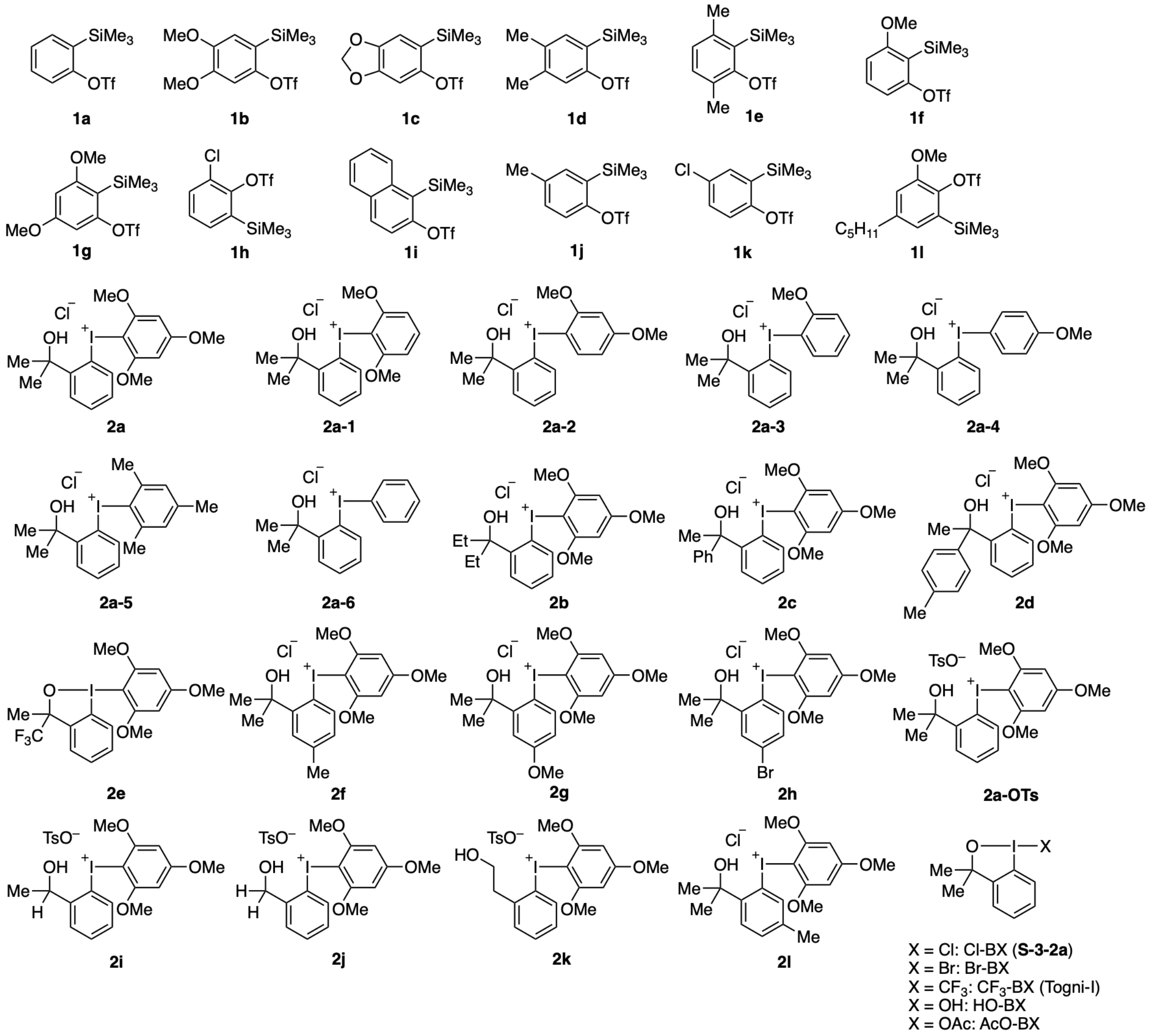

Figure S1. Substrates used in this study.

2. Preparation of Diaryliodonium Reagents

2-1. Synthesis of Diaryliodonium Chlorides

Diaryliodonium chlorides (**2a** and its analogues **2a-1**–**2a-6**, **2b**–**2d**, **2f**–**2h**, and **2l**) were synthesized via the reaction of chlorobenziodoxoles **S-3** with aryllithium reagents (Scheme S1). Chlorobenziodoxoles **S-3** were synthesized via the oxidation of the corresponding 2-iodobenzyl alcohol derivatives **S-2**, which were derived from appropriate precursors (esters **S-1** or ketones **S-1'**) and Grignard reagents. Known chlorobenziodoxoles **S-3-2a**,[6] **S-3-2b**,[10] **S-3-2c**,[11] **S-3-2e**,[10] and **S-3-2g**[12] were synthesized according to the literature procedure. Described below are specific synthetic procedures and characterization data for new chlorobenziodoxoles and diaryliodonium chlorides.

Scheme S1. General Synthetic Routes to Diaryliodonium Chlorides

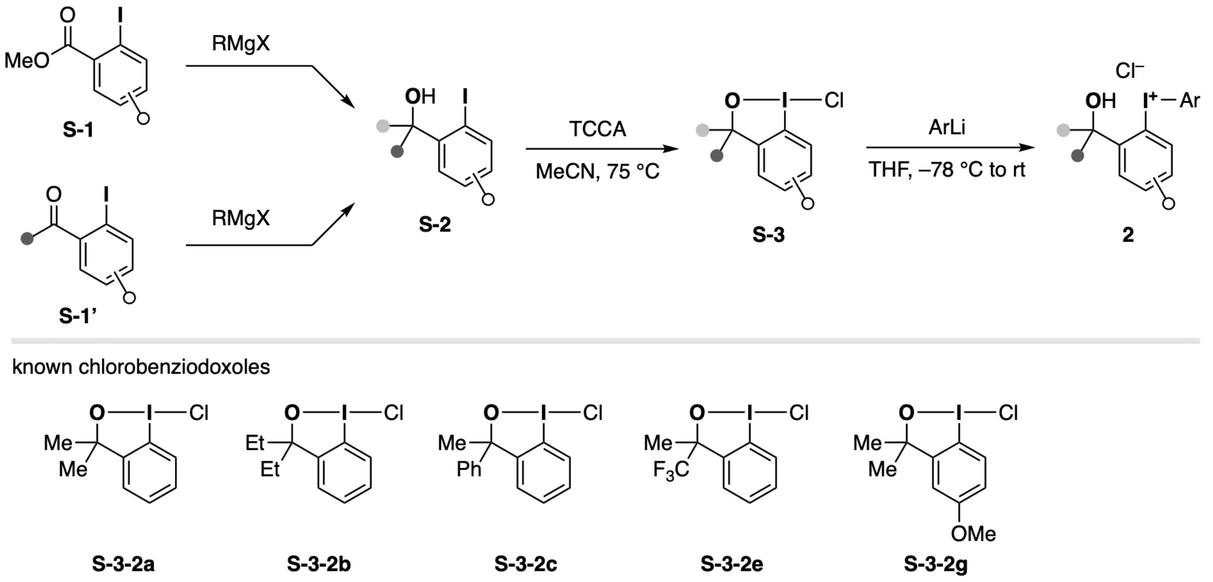

2-1-1. Synthesis of Chlorobenziodoxoles S-3-2f, S-3-2h, and S-3-2i

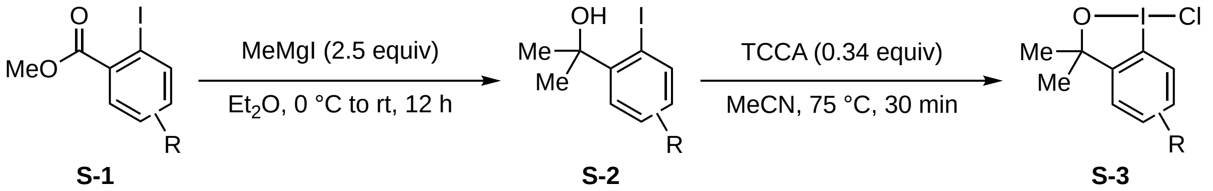

General Procedure (GP1): Under an argon atmosphere, a 30 mL two-necked flask equipped with a magnetic stir bar was charged with **S-1** (5.0 mmol, 1.0 equiv) and Et_2O (10 mL). To the solution was added MeMgI (3.0 M in Et_2O, 4.17 mL, 12.5 mmol, 2.5 equiv) dropwise at 0 °C. The resulting mixture was allowed to gradually warm to room temperature and stirred for 12

h. After quenching with sat. NH_4Cl aq., the mixture was extracted with EtOAc (50 mL × 3). The combined organic layer was dried over $MgSO_4$ and concentrated under reduced pressure to give dimethylbenzyl alcohol **S-2**, which was used directly without purification. In a separate 30 mL two-necked flask under an argon atmosphere, **S-2** was dissolved in MeCN (3 mL) and heated to 75 °C in an oil bath. A solution of trichloroisocyanuric acid (TCCA; 395 mg, 1.7 mmol, 0.34 equiv) in MeCN (2 mL) was slowly added via a dropping funnel to the solution of **S-2**, and the mixture was stirred for 30 min at the same temperature. The reaction mixture was filtered under reduced pressure, and the filter cake was washed with additional hot MeCN (2 mL). The filtrate was concentrated under reduced pressure, and the residue was purified by column chromatography on silica gel (eluent: CH_2Cl_2/MeOH = 30/1) to afford chlorobenziodoxole **S-3**.

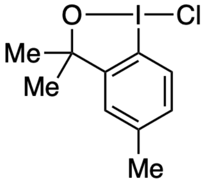

1-Chloro-3,3,5-trimethyl-1,3-dihydro-1λ³-benzo[*d*][1,2]iodaoxole (S-3-2f): Synthesized according to **GP1** from methyl 2-iodo-5-methylbenzoate[13] (**S-1-2f**; 1.38 g, 5.00 mmol). Yellow solid (1.26 g, 81%); m.p. 110−111 °C; R_f 0.29 (CH_2Cl_2/MeOH = 30/1); **^{1}H NMR** (400 MHz, $CDCl_3$) δ 7.85 (d, *J* = 8.4 Hz, 1H), 7.37 (d, *J* = 8.6 Hz, 1H), 6.96 (s, 1H), 2.47 (s, 3H), 1.53 (s, 6H); **^{13}C{^{1}H} NMR** (150 MHz, $CDCl_3$) δ 149.6, 141.7, 131.5, 128.2, 127.0, 111.2, 85.1, 29.3, 21.0; **HRMS** (FAB) *m*/*z*: $[M + H]^+$ calcd for $C_{10}H_{13}ClIO$ 310.9694, found 310.9692.

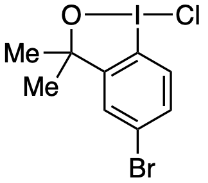

5-Bromo-1-chloro-3,3-dimethyl-1,3-dihydro-1λ³-benzo[*d*][1,2]iodaoxole (S-3-2h): Synthesized according to **GP1** from methyl 5-bromo-2-iodobenzoate[13] (**S-1-2h**; 1.70 g, 4.99 mmol). Yellow solid (294 mg, 16%); m.p. 164−165 °C; R_f 0.18 (CH_2Cl_2/MeOH = 30/1); **^{1}H NMR** (400 MHz, $CDCl_3$) δ 7.86 (d, *J* = 8.7 Hz, 1H), 7.66 (dd, *J* = 8.7, 2.2 Hz, 1H), 7.29 (d, *J* = 2.2 Hz, 1H), 1.55 (s, 6H); **^{13}C{^{1}H} NMR** (150 MHz, $CDCl_3$) δ 151.9, 133.4, 130.1, 129.3, 126.2, 113.1, 84.6, 29.2; **HRMS** (FAB) *m*/*z*: $[M + H]^+$ calcd for $C_9H_{10}BrClIO$ 374.8643, found 374.8663.

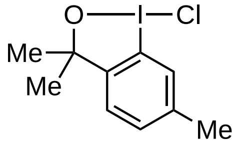

1-Chloro-3,3,6-trimethyl-1,3-dihydro-1λ^3-benzo[*d*][1,2]iodaoxole (S-3-2l): Synthesized according to **GP1** from methyl 2-iodo-4-methylbenzoate[13] (**S-1-2l**; 996 mg, 3.61 mmol). Yellow solid (586 mg, 52%); m.p. 141−142 °C; R_f 0.31 (CH_2Cl_2/MeOH = 30/1); **^{1}H NMR** (400 MHz, $CDCl_3$) δ 7.80 (s, 1H), 7.31 (d, *J* = 7.7 Hz, 1H), 7.02 (d, *J* = 7.6 Hz, 1H), 2.46 (s, 3H), 1.53 (s, 6H); **^{13}C{^{1}H} NMR** (101 MHz, $CDCl_3$) δ 146.8, 141.4, 132.0, 128.5, 125.8, 114.9, 85.2, 29.4, 21.5; **HRMS** (FAB) *m/z*: $[M + H]^+$ calcd for $C_{10}H_{13}ClIO$ 310.9694, found 310.9696.

2-1-2. Synthesis of Chlorobenziodoxole S-3-2d

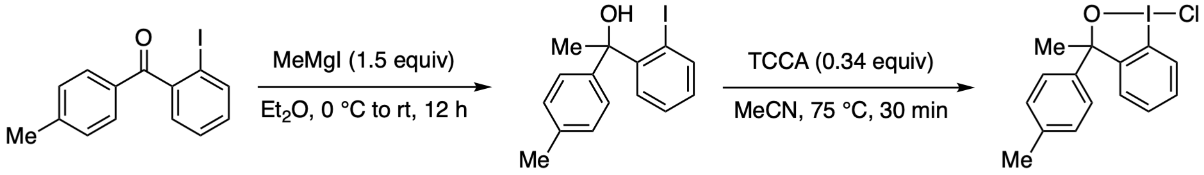

S-1-2d **S-2-2d** **S-3-2d**

1-Chloro-3-methyl-3-(*p*-tolyl)-1,3-dihydro-1λ^3-benzo[*d*][1,2]iodaoxole (S-3-2d): Under an argon atmosphere, a 50 mL two-necked flask equipped with a magnetic stir bar was charged with (2-iodophenyl)(*p*-tolyl)methanone[14] (**S-1-2d**; 1.61 g, 5.00 mmol, 1.0 equiv) and Et_2O (10 mL). To the solution was added MeMgI (3.0 M in Et_2O, 2.5 mL, 7.5 mmol, 1.5 equiv) dropwise at 0 °C. The resulting mixture was allowed to gradually warm to room temperature and stirred for 12 h. After quenching with sat. NH_4Cl aq., the mixture was extracted with EtOAc (50 mL × 3). The combined organic layer was dried over $MgSO_4$ and concentrated under reduced pressure to give 1-(2-iodophenyl)-1-(*p*-tolyl)ethan-1-ol (**S-2-2d**), which was used directly without purification. In a separate 30 mL two-necked flask under an argon atmosphere, **S-2-2d** was dissolved in MeCN (3 mL) and heated to 75 °C in an oil bath. To this solution was added a solution of trichloroisocyanuric acid (TCCA; 395 mg, 1.70 mmol, 0.34 equiv) in MeCN (2 mL) slowly via a dropping funnel, and the mixture was stirred for 30 min at the same temperature. The reaction mixture was filtered under reduced pressure, and the filter cake was washed with additional hot MeCN (2 mL). The filtrate was concentrated under reduced pressure, and the residue was purified by column chromatography on silica gel (eluent: CH_2Cl_2/MeOH = 30/1) to afford 1-chloro-3-methyl-3-(*p*-tolyl)-1,3-dihydro-1λ^3-

benzo[*d*][1,2]iodaoxole (**S-3-2d**) as a yellow solid (1.61 g, 86%).

R_f 0.14 (CH_2Cl_2/MeOH = 30/1); m.p. 131–132 °C; **^{1}H NMR** (400 MHz, $CDCl_3$) δ 8.06 (d, J = 8.0 Hz, 1H), 7.58 (t, 7.7 Hz, 1H), 7.54 (t, 7.3 Hz, 1H), 7.23 (dd, J = 7.4, 1.4 Hz, 1H), 7.19 (d, 8.1 Hz, 2H), 7.12 (d, 8.1 Hz, 2H), 2.32 (s, 3H), 1.89 (s, 3H); **^{13}C{^{1}H} NMR** (150 MHz, $CDCl_3$) δ 148.1, 141.5, 137.9, 130.7, 130.6, 129.3, 128.7, 128.1, 126.0, 116.6, 88.0, 29.4, 21.2; **HRMS** (FAB) *m/z*: $[M + H]^+$ calcd for $C_{15}H_{15}ClIO$ 372.9851, found 372.9852.

2-1-3. Synthesis of Diaryliodonium Chlorides

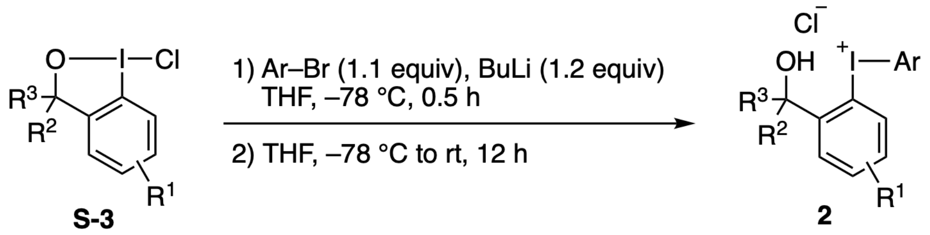

General Procedure (GP2): Under an argon atmosphere, a 30 mL two-necked flask equipped with a magnetic stir bar was charged with aryl bromide (2.2 mmol, 1.1 equiv) and THF (16 mL). To the solution was added slowly BuLi (1.6 M in hexane, 1.55 mL, 2.4 mmol, 1.2 equiv) at –78 °C, and the mixture was stirred for 0.5 h at the same temperature (referred to as suspension A). In a separate 50 mL two-necked flask under an argon atmosphere, chlorobenziodoxole **S-3** (2.0 mmol, 1.0 equiv) was dissolved in THF (4 mL) and cooled to –78 °C. Suspension A was added dropwise to this solution, and the resulting mixture was allowed to gradually warm to room temperature and stirred for 12 h. After quenching with sat. NH_4Cl aq., the mixture was concentrated under reduced pressure. The residue was extracted with CH_2Cl_2 (10 mL × 3), washed with brine (10 mL), dried over $MgSO_4$, and concentrated under reduced pressure. The resulting solid was triturated with THF (20 mL), washed with THF (ca. 5 mL × 3), and dried under vacuum to afford the diaryliodonium chloride **2**.

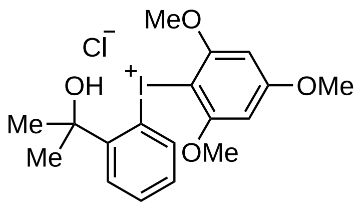

(2-(2-Hydroxypropan-2-yl)phenyl)(2,4,6-trimethoxyphenyl)iodonium chloride (2a): Synthesized according to **GP2** from **S-3-2a** (1.48 g, 4.99 mmol). Colorless solid (1.90 g, 82%); R_f 0.13 (CH_2Cl_2/MeOH = 10/1); m.p. 209−210 °C; **^{1}H NMR** (400 MHz, $CDCl_3$) δ 8.93 (brs,

1H), 7.46−7.39 (m, 2H), 7.14 (ddd, J = 8.6, 6.3, 2.2 Hz, 1H), 6.82 (dd, J = 8.4, 1.2 Hz, 1H), 6.28 (s, 2H), 3.96 (s, 3H), 3.86 (s, 6H), 1.81 (s, 6H); **^{13}C{^{1}H} NMR** (150 MHz, $CDCl_3$) δ 167.8, 161.9, 147.6, 130.6, 130.0, 128.4, 127.2, 109.0, 91.6, 83.6, 74.3, 57.1, 56.2, 30.5; **HRMS** (FAB) *m*/*z*: $[M–Cl]^+$ calcd for $C_{18}H_{22}IO_4$ 429.0557, found 429.0569.

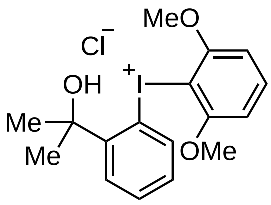

(2,6-Dimethoxyphenyl)(2-(2-hydroxypropan-2-yl)phenyl)iodonium chloride (2a-1): Synthesized according to **GP2** from **S-3-2a** (593 mg, 2.00 mmol). Colorless solid (433.0 mg, 50%); R_f 0.12 (CH_2Cl_2/MeOH = 10/1); m.p. 186−187 °C; **^{1}H NMR** (400 MHz, $CDCl_3$) δ 7.64 (t, J = 8.4 Hz, 1H), 7.45−7.40 (m, 2H), 7.13−7.08 (m, 1H), 6.78 (d, J = 8.0 Hz, 1H), 6.75 (d, J = 8.4 Hz, 2H), 3.88 (s, 6H), 1.81 (s, 6H); **^{13}C{^{1}H} NMR** (150 MHz, $CDCl_3$) δ 160.5, 147.7, 137.0, 130.5, 129.9, 128.2, 127.8, 109.4, 104.8, 94.6, 74.3, 57.2, 30.4; **HRMS** (FAB) *m*/*z*: $[M–Cl]^+$ calcd for $C_{17}H_{20}IO_3$ 399.0452, found 399.0450.

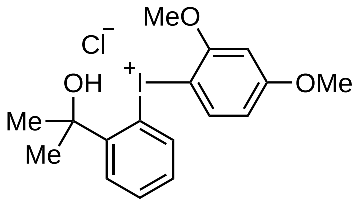

(2,4-Dimethoxyphenyl)(2-(2-hydroxypropan-2-yl)phenyl)iodonium chloride (2a-2): Synthesized according to **GP2** from **S-3-2a** (890 mg, 3.00 mmol). Colorless solid (996 mg, 76%); R_f 0.17 (CH_2Cl_2/MeOH = 10/1); m.p. 189−190 °C; **^{1}H NMR** (600 MHz, $CDCl_3$) δ 7.98 (d, J = 8.4 Hz, 1H), 7.36−7.39 (m, 2H), 7.11−7.05 (m, 1H), 6.82 (d, J = 8.2 Hz, 1H), 6.62−6.59 (m, 2H), 3.90 (s, 3H), 3.81 (s, 3H), 1.76 (s, 6H); **^{13}C{^{1}H} NMR** (150 MHz, $CDCl_3$) δ 165.6, 160.0, 147.8, 140.8, 130.1, 129.6, 128.5, 128.0, 111.9, 108.7, 99.7, 94.9, 74.6, 56.8, 56.0, 30.6; **HRMS** (FAB) *m*/*z*: $[M–Cl]^+$ calcd for $C_{17}H_{20}IO_3$ 399.0452, found 399.0451.

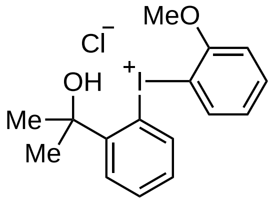

(2-(2-Hydroxypropan-2-yl)phenyl)(2-methoxyphenyl)iodonium chloride (2a-3): Synthesized according to **GP2** from **S-3-2a** (890 mg, 3.00 mmol). Colorless solid (824 mg, 68%); R_f 0.12 (CH_2Cl_2/MeOH = 10/1); m.p. 209−210 °C; ^{1}**H NMR** (400 MHz, $CDCl_3$) δ 8.12 (dd, J = 7.8, 1.4 Hz, 1H), 7.67−7.61 (m, 1H), 7.41−7.34 (m, 2H), 7.11−7.01 (m, 3H), 6.78 (d, J = 8.1 Hz, 1H), 3.83 (s, 3H), 1.78 (s, 6H); ^{13}**C{^{1}H} NMR** (150 MHz, $CDCl_3$) δ 158.2, 148.0, 139.8, 135.0, 130.1, 129.6, 129.0, 127.9, 123.7, 112.4, 112.2, 106.1, 74.7, 56.9, 30.5; **HRMS** (FAB) *m*/*z*: $[M–Cl]^+$ calcd for $C_{16}H_{18}IO_2$ 369.0346, found 369.0349.

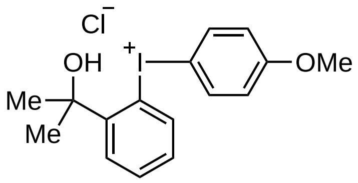

(2-(2-Hydroxypropan-2-yl)phenyl)(4-methoxyphenyl)iodonium chloride (2a-4): Synthesized according to **GP2** from **S-3-2a** (297 mg, 1.00 mmol). Colorless solid (307 mg, 76%); R_f 0.20 (CH_2Cl_2/MeOH = 10/1); m.p. 194−195 °C; ^{1}**H NMR** (600 MHz, $CDCl_3$) δ 8.11 (brs, 1H), 7.95 (d, J = 9.0 Hz, 2H), 7.40–7.36 (m, 2H), 7.11–7.05 (m, 1H), 6.99 (d, J = 8.9 Hz, 2H), 6.83 (d, J = 8.0 Hz, 1H), 3.88 (s, 3H), 1.76 (s, 6H); ^{13}**C{^{1}H} NMR** (150 MHz, $CDCl_3$) δ 162.8, 148.1, 139.6, 130.2, 129.7, 129.5, 128.2, 117.8, 113.5, 104.2, 74.9, 55.7, 30.8; **HRMS** (FAB) *m*/*z*: $[M–Cl]^+$ calcd for $C_{16}H_{18}IO_2$ 369.0346, found 369.0350.

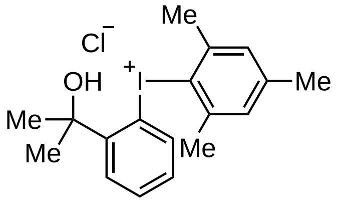

(2-(2-Hydroxypropan-2-yl)phenyl)(mesityl)iodonium chloride (2a-5): Synthesized according to **GP2** from **S-3-2a** (148 mg, 0.499 mmol). Colorless solid (107 mg, 51%); R_f 0.13 (CH_2Cl_2/MeOH = 10/1); m.p. 214−215 °C; ^{1}**H NMR** (600 MHz, $CDCl_3$) δ 7.38 (dd, J = 7.7, 1.7 Hz, 1H), 7.35 (t, J = 7.4 Hz, 1H), 7.08 (s, 2H), 7.01 (ddd, J = 8.52, 6.6, 1.9 Hz, 1H), 6.67 (dd, J = 8.3, 1.0 Hz, 1H) , 2.62 (s, 6H) , 2.36 (s, 3H), 1.76 (s, 6H); ^{13}**C{^{1}H} NMR** (150 MHz,

$CDCl_3$) δ 148.7, 143.2, 143.1, 130.1, 129.8, 129.7, 128.4, 128.2, 122.0, 112.4, 74.5, 30.7, 27.1, 21.4; **HRMS** (FAB) *m/z*: $[M–Cl]^+$ calcd for $C_{18}H_{22}IO$ 381.0710, found 381.0710.

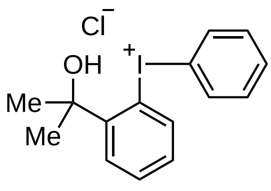

(2-(2-Hydroxypropan-2-yl)phenyl)(phenyl)iodonium chloride (2a-6): Synthesized according to **GP2** from **S-3-2a** (148 mg, 0.499 mmol). Colorless solid (93.2 mg, 50%); R_f 0.14 (CH_2Cl_2/MeOH = 10/1); m.p. 230−231 °C; **^{1}H NMR** (600 MHz, $CDCl_3$) δ 8.08 (dd, *J* = 8.2, 1.0 Hz, 2H), 7.67 (t, *J* = 7.5 Hz, 1H), 7.50 (t, *J* = 7.7 Hz, 2H), 7.40–7.34 (m, 2H), 7.07–7.03 (m, 1H), 6.78 (d, *J* = 8.4 Hz, 1H), 1.76 (s, 6H); **^{13}C{^{1}H} NMR** (150 MHz, $CDCl_3$) δ 148.2, 137.7, 132.2, 131.8, 130.2, 130.0, 129.7, 128.1, 115.8, 113.8, 75.0, 30.8; **HRMS** (FAB) *m/z*: $[M–Cl]^+$ calcd for $C_{15}H_{16}IO$ 339.0240, found 339.0250.

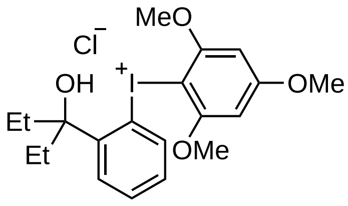

(2-(3-Hydroxypentan-3-yl)phenyl)(2,4,6-trimethoxyphenyl)iodonium chloride (2b): Synthesized according to **GP2** from **S-3-2b** (649 mg, 2.00 mmol). Colorless solid (716 mg, 73%); R_f 0.20 (CH_2Cl_2/MeOH = 10/1); m.p. 218−219 °C; **^{1}H NMR** (600 MHz, $CDCl_3$) δ 7.46 (t, *J* = 6.9 Hz, 1H), 7.27 (dd, *J* = 7.9, 1.7 Hz, 1H), 7.17−7.13 (m, 1H), 6.79 (d, *J* = 7.6 Hz, 1H), 6.28 (s, 2H), 3.97 (s, 3H), 3.85 (s, 6H), 2.28−2.21 (m, 2H), 2.03−1.95 (m, 2H), 0.90 (t, *J* = 7.2 Hz, 6H); **^{13}C{^{1}H} NMR** (150 MHz, $CDCl_3$) δ 167.8, 162.0, 143.9, 130.4, 129.8, 129.3, 126.7, 110.2, 91.6, 83.0, 80.0, 57.0, 56.2, 35.0, 8.2; **HRMS** (FAB) *m/z*: $[M–Cl]^+$ calcd for $C_{20}H_{26}IO_4$ 457.0870, found 457.0887.

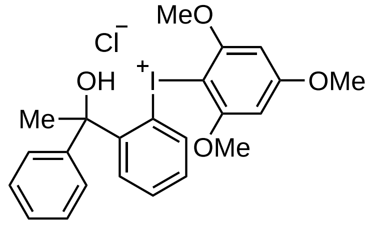

(2-(1-Hydroxy-1-phenylethyl)phenyl)(2,4,6-trimethoxyphenyl)iodonium chloride (2c):

Synthesized according to **GP2** from **S-3-2c** (359 mg, 1.00 mmol). Colorless solid (225 mg, 43%); R_f 0.14 (CH_2Cl_2/MeOH = 10/1); m.p. 155−156 °C; **^{1}H NMR** (400 MHz, $CDCl_3$) δ 7.50−7.41 (m, 4H), 7.35−7.24 (m, 3H), 7.28−7.12 (m, 1H), 6.89 (d, J = 8.2 Hz, 1H), 6.20 (s, 2H), 3.91 (s, 3H), 3.72 (s, 6H), 2.22 (s, 3H); **^{13}C{^{1}H} NMR** (150 MHz, $CDCl_3$) δ 167.6, 161.6, 146.7, 145.3, 130.2, 130.1, 129.9, 128.2, 128.0, 127.4, 126.6, 112.8, 91.6, 84.1, 77.6, 56.9, 56.2, 30.0; **HRMS** (FAB) *m*/*z*: $[M–Cl]^+$ calcd for $C_{23}H_{24}IO_4$ 491.0714, found 491.0735.

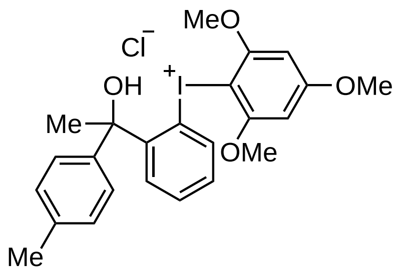

(2-(1-Hydroxy-1-(p-tolyl)ethyl)phenyl)(2,4,6-trimethoxyphenyl)iodonium chloride (2d): Synthesized according to **GP2** from **S-3-2d** (745 mg, 2.00 mmol). Colorless solid (575 mg, 53%); R_f 0.14 (CH_2Cl_2/MeOH = 10/1); m.p. 153–154 °C; **^{1}H NMR** (400 MHz, $CDCl_3$) δ 7.44–7.38 (m, 2H), 7.34 (d, J = 8.3 Hz, 2H), 7.17–7.10 (m, 3H), 6.87 (d, J = 8.6 Hz, 1H), 6.21 (s, 2H), 3.92 (s, 3H), 3.75 (s, 6H), 2.33 (s, 3H), 2.20 (s, 3H); **^{13}C{^{1}H} NMR** (150 MHz, $CDCl_3$) δ 167.6, 161.6, 146.8, 142.4, 137.1, 130.2, 130.1, 129.9, 128.9, 127.8, 126.6, 112.3, 91.6, 83.9, 77.5, 56.9, 56.2, 29.9, 21.1; **HRMS** (FAB) *m*/*z*: $[M–Cl]^+$ calcd for $C_{24}H_{26}IO_4$ 505.0870, found 505.0885.

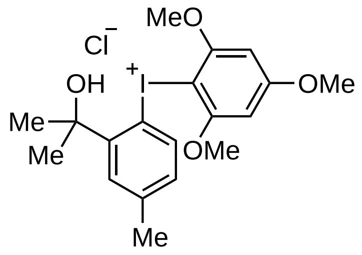

(2-(2-Hydroxypropan-2-yl)-4-methylphenyl)(2,4,6-trimethoxyphenyl)iodonium chloride (2f): Synthesized according to **GP2** from **S-3-2f** (621 mg, 2.00 mmol). Colorless solid (639 mg, 67%); R_f 0.13 (CH_2Cl_2/MeOH = 10/1); m.p. 205−206 °C; **^{1}H NMR** (400 MHz, $CDCl_3$) δ 9.09 (brs, 1H), 7.21 (d, J = 2.0 Hz, 1H), 6.94 (dd, J = 8.4, 2.0 Hz, 1H), 6.65 (d, J = 8.5 Hz, 1H), 6.25 (s, 2H), 3.94 (s, 3H), 3.85 (s, 6H), 2.36 (s, 3H), 1.80 (s, 6H); **^{13}C{^{1}H} NMR** (150 MHz, $CDCl_3$) δ 167.8, 162.0, 147.4, 141.1, 130.9, 129.2, 126.7, 104.8, 91.5, 83.2, 74.2, 57.0, 56.2, 30.6, 21.0; **HRMS** (FAB) *m*/*z*: $[M–Cl]^+$ calcd for $C_{19}H_{24}IO_4$ 443.0714, found 443.0704.

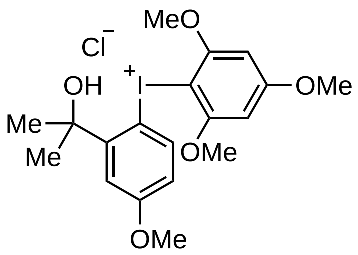

(2-(2-Hydroxypropan-2-yl)-4-methoxyphenyl)(2,4,6-trimethoxyphenyl)iodonium chloride (2g): Synthesized according to **GP2** from **S-3-2g** (327 mg, 1.00 mmol). Colorless solid (314 mg, 63%); R_f 0.07 (CH_2Cl_2/MeOH = 10/1); m.p. 144–145 °C; **^{1}H NMR** (600 MHz, $CDCl_3$) δ 7.31(s, 1H), 6.91 (d, *J* = 1.9 Hz, 1H), 6.71–6.69 (m, 2H), 6.28 (s, 2H) 3.96 (s, 3H), 3.87 (s, 6H), 3.80 (s, 3H), 1.78 (s, 6H); **^{13}C{^{1}H} NMR** (150 MHz, $CDCl_3$) δ 167.7, 161.7, 161.6, 148.8, 128.4, 115.1, 114.5, 97.9, 91.7, 83.5, 74.2, 57.1, 56.3, 55.8, 30.4; **HRMS** (FAB) *m/z*: $[M–Cl]^+$ calcd for $C_{19}H_{24}IO_5$ 459.0663, found 459.0653.

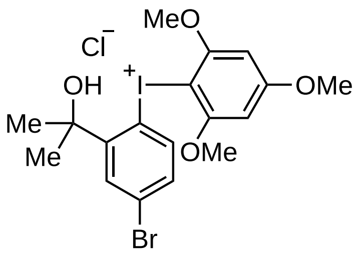

(4-Bromo-2-(2-hydroxypropan-2-yl)phenyl)(2,4,6-trimethoxyphenyl)iodonium chloride (2h): Synthesized according to **GP2** from **S-3-2h** (189 mg, 0.503 mmol). Colorless solid (115 mg, 42%); R_f 0.14 (CH_2Cl_2/MeOH = 10/1); m.p. 148–149 °C; **^{1}H NMR** (600 MHz, $CDCl_3$) δ 7.47 (d, *J* = 2.0 Hz, 1H), 7.19 (dd, *J* = 8.9, 2.0 Hz, 1H), 6.69 (d, *J* = 8.9 Hz, 1H), 6.25 (s, 2H), 3.92 (s, 3H), 3.86 (s, 6H), 1.77 (s, 6H); **^{13}C{^{1}H} NMR** (150 MHz, $CDCl_3$) δ 167.4, 161.5, 150.1, 132.5, 130.9, 129.6, 124.9, 109.6, 91.7, 85.9, 74.1, 57.1, 56.1, 30.3; **HRMS** (FAB) *m/z*: $[M–Cl]^+$ calcd for $C_{18}H_{21}BrIO_4$ 506.9662, found 506.9651.

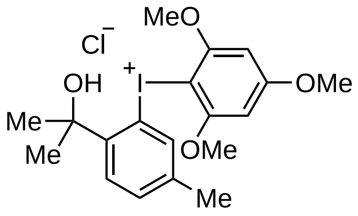

(2-(2-Hydroxypropan-2-yl)-5-methylphenyl)(2,4,6-trimethoxyphenyl)iodonium chloride

(2l): Synthesized according to **GP2** from **S-3-2l** (311 mg, 1.00 mmol). Colorless solid (144 mg, 30%); R_f 0.14 (CH_2Cl_2/MeOH = 10/1); m.p. 165−166 °C; **^{1}H NMR** (600 MHz, $CDCl_3$) δ 8.99 (brs, 1H), 7.29 (dd, J = 8.3, 2.1 Hz, 1H), 7.24 (d, J = 8.2 Hz, 1H), 6.56 (s, 1H), 6.27 (s, 2H), 3.96 (s, 3H), 3.86 (s, 6H), 2.20 (s, 3H), 1.79 (s, 6H); **^{13}C{^{1}H} NMR** (150 MHz, $CDCl_3$) δ 167.8, 162.0, 144.6, 140.3, 131.6, 128.1, 127.0, 108.5, 91.5, 83.1, 74.2, 57.1, 56.2, 30.6, 21.2; **HRMS** (FAB) m/z: $[M–Cl]^+$ calcd for $C_{19}H_{24}IO_4$ 443.0714, found 443.0719.

2-2. Synthesis of Benziodoxole Reagent 2e

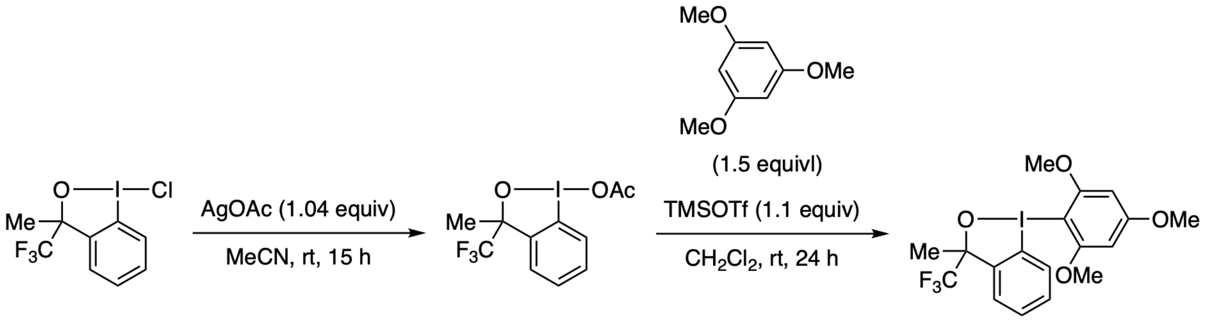

S-3-2e **S-4-2e** **2e**

3-Methyl-3-(trifluoromethyl)-1-(2,4,6-trimethoxyphenyl)-1,3-dihydro-1λ^3-benzo[*d*][1,2]iodaoxole (2e): Under an argon atmosphere, a 30 mL two-necked flask equipped with a magnetic stir bar was charged with **S-3-2e** (701 mg, 2.00 mmol, 1.0 equiv), AgOAc (347 mg, 2.08 mmol, 1.04 equiv) and MeCN (6 mL). The reaction mixture was stirred for 15 h at the room temperature in the dark. The mixture was filtered under reduced pressure, and the filter cake was washed with additional MeCN (1 mL). The filtrate was concentrated under reduced pressure to afford 3-methyl-3-(trifluoromethyl)-1λ^3-benzo[*d*][1,2]iodaoxol-1(3*H*)-yl acetate **S-4-2e** (748 mg, 100%), which was used in the next step without further purification. Under an argon atmosphere, a 30 mL two-necked flask equipped with a magnetic stir bar was charged with **S-4-2e** (374 mg, 1.0 mmol, 1.0 equiv) and CH_2Cl_2 (8 mL). To the solution was added TMSOTf (0.20 mL, 1.1 mmol, 1.1 equiv) and the mixture was stirred for 30 min at the room temperature. To the mixture was added 1,3,5-trimethoxybenzene (252 mg, 1.5 mmol, 1.5 equiv) and the mixture was stirred for 24 h at the same temperature. After quenching with sat. Na_2CO_3 aq., the mixture was extracted with CH_2Cl_2 (5 mL × 3), washed with brine (5 mL), dried over Na_2SO_4, and concentrated under reduced pressure. The residue was purified by column chromatography (eluent: CH_2Cl_2/MeOH = 10/1) on silica gel to afford the desired product **2e** as a colorless solid (428 mg, 89%).

R_f 0.17 (CH_2Cl_2/MeOH = 10/1); m.p. 187–188 °C; **^{1}H NMR** (600 MHz, $CDCl_3$) δ 7.56 (d, J = 7.6 Hz, 1H), 7.44 (t, J = 7.3 Hz, 1H), 7.20 (ddd, J = 8.3, 7.1, 1.3 Hz, 1H), 6.79 (d, J = 8.1 Hz, 1H), 6.18 (s, 2H), 3.90 (s, 3H), 3.77 (s, 6H), 1.71 (s, 3H); **^{13}C{^{1}H} NMR** (150 MHz, $CDCl_3$) δ 165.4, 162.2, 162.0, 139.7, 129.9, 129.3, 128.9, 127.3 (q, J_{C-F} = 289.5 Hz), 125.9, 111.8, 92.1, 90.4, 76.7 (q, J_{C-FF} = 27.1 Hz), 56.0, 55.5, 25.0; **^{19}F NMR** (376 MHz, CD_3OD) δ –80.8; **HRMS** (FAB) *m/z*: $[M + H]^+$ calcd for $C_{18}H_{19}F_3IO_4$ 483.0275, found 483.0288.

2-3. Synthesis of Diaryliodonium Tosylates

Diaryliodonium tosylates (**2a-OTs** and **2i**–**2k**) were synthesized in a one-pot manner via oxidation of 2-iodobenzyl alcohol derivative (or 2-iodophenethyl alcohol; **S-2**) followed by arylation with 1,3,5-trimethoxybenzene according to the following general procedure (**GP3**).

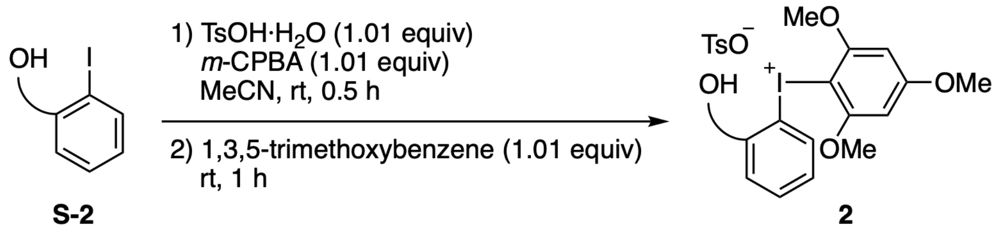

General Procedure (GP3): Under an argon atmosphere, a 4 mL vial equipped with a magnetic stir bar was charged with aryl iodide **S-2** (1.0 mmol, 1 equiv), TsOH·H_2O (192 mg, 1.01 mmol, 1.01 equiv), and MeCN (1 mL). To the solution was added *m*CPBA (249 mg, 1.01 mmol, 1.01 equiv), and the mixture was stirred for 0.5 h at room temperature. To the solution was added 1,3,5-trimethoxybenzene (167 mg, 1.01 mmol, 1.01 equiv) at room temperature, and stirring was continued for 1 h. The mixture was concentrated under reduced pressure, and the residue was purified by column chromatography on silica gel (eluent: CH_2Cl_2/MeOH = 40/1 to 10/1) to afford the desired iodonium tosylate **2**.

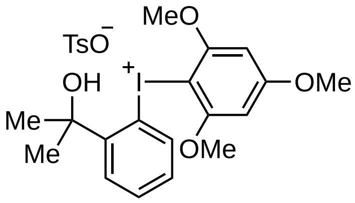

(2-(2-Hydroxypropan-2-yl)phenyl)(2,4,6-trimethoxyphenyl)iodonium 4-methylbenzenesulfonate (2a-OTs): Synthesized according to **GP3** from **S-2-2a** (446 mg, 1.7 mmol). Off-white solid (775 mg, 76%); R_f 0.11 (CH_2Cl_2/MeOH = 10/1); m.p. 141−142 °C; **^{1}H NMR** (400 MHz, $CDCl_3$) δ 7.82 (d, J = 8.2 Hz, 2H), 7.46−7.38 (m, 2H), 7.16−7.11 (m, 3H), 6.82 (d, J = 8.4 Hz, 1H), 6.24 (s, 2H), 3.94 (s, 3H), 3.85 (s, 6H), 2.32 (s, 3H), 1.74 (s, 6H);

$^{13}C\{^1H\}$ **NMR** (150 MHz, $CDCl_3$) δ 167.8, 162.1, 147.4, 142.5, 139.9, 130.6, 130.0, 128.7, 128.4, 127.2, 126.4, 108.0, 91.5, 83.3, 74.0, 57.0, 56.2, 30.6, 21.5; **HRMS** (FAB) *m*/*z*: [M–OTs]$^+$ calcd for $C_{18}H_{22}IO_4$ 429.0557, found 429.0548.

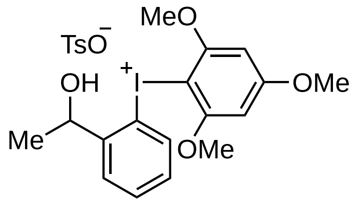

(2-(1-Hydroxyethyl)phenyl)(2,4,6-trimethoxyphenyl)iodonium 4-methylbenzenesulfonate (2i): Synthesized according to **GP3** from 1-(2-iodophenyl)ethan-1-ol (**S-2-2i**; 248 mg, 1.0 mmol). Off-white amorphous (381 mg, 65%); R_f 0.13 (CH_2Cl_2/MeOH = 10/1); ^{1}**H NMR** (400 MHz, $CDCl_3$) δ 7.85–7.82 (m, 2H), 7.42 (t, *J* = 7.4 Hz, 1H), 7.31 (d, *J* = 7.4 Hz, 1H), 7.18−7.11 (m, 3H), 6.82 (d, *J* = 8.3 Hz, 1H), 6.24 (s, 2H), 5.30 (q, *J* = 6.5 Hz, 1H), 3.94 (s, 3H), 3.85 (s, 6H), 2.32 (s, 3H), 1.65 (d, *J* = 6.4 Hz, 3H); $^{13}C\{^1H\}$ **NMR** (150 MHz, $CDCl_3$) δ 167.9, 161.9, 144.0, 143.2, 139.4, 130.5, 130.2, 128.9, 128.5, 127.2, 126.3, 108.8, 91.5, 81.7, 69.1, 57.0, 56.2, 23.9, 21.5; **HRMS** (FAB) *m*/*z*: [M–OTs]$^+$ calcd for $C_{17}H_{20}IO_4$ 415.0401, found 415.0397.

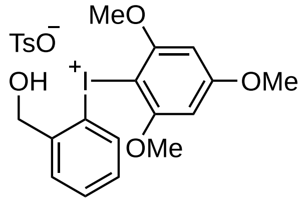

(2-(Hydroxymethyl)phenyl)(2,4,6-trimethoxyphenyl)iodonium 4-methylbenzenesulfonate (2j): Synthesized according to **GP3** from (2-iodophenyl)methanol (**S-2-2j**; 46.8 mg, 0.2 mmol). Colorless solid (91.4 mg, 80%); R_f 0.13 (CH_2Cl_2/MeOH = 10/1); m.p. 161−162 °C; ^{1}**H NMR** (600 MHz, $CDCl_3$) δ 8.46–8.31 (brs, 1H), 7.80 (d, *J* = 8.1 Hz, 2H), 7.40 (t, *J* = 7.2 Hz, 1H), 7.31–7.26 (m, 1H), 7.16 (t, *J* = 7.4 Hz, 1H), 7.12 (d, *J* = 7.9 Hz, 2H), 6.84 (d, *J* = 8.3 Hz, 1H), 6.27 (s, 2H), 5.05 (s, 2H), 3.94 (s, 3H), 3.85 (s, 6H), 2.31 (s, 3H); $^{13}C\{^1H\}$ **NMR** (150 MHz, $CDCl_3$) δ 168.0, 161.8, 143.2, 139.7, 139.4, 130.52, 130.48, 129.8, 128.7, 127.5, 126.2, 109.8, 91.7, 80.5, 63.5, 57.1, 56.3, 21.4; **HRMS** (FAB) *m*/*z*: [M–OTs]$^+$ calcd for $C_{16}H_{18}IO_4$ 401.0245, found 401.0225.

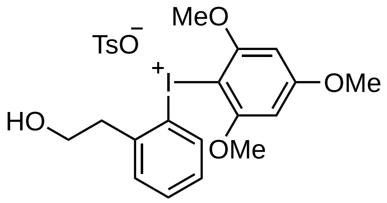

(2-(2-Hydroxyethyl)phenyl)(2,4,6-trimethoxyphenyl)iodonium 4-methylbenzenesulfonate (2k): Synthesized according to **GP3** from 2-(2-iodophenyl)ethan-1-ol (**S-2-2k**; 248 mg, 1.0 mmol). Off-white solid (444 mg, 76%); R_f 0.13 (CH_2Cl_2/MeOH = 10/1); m.p. 142 °C; **^{1}H NMR** (600 MHz, $CDCl_3$) δ 7.78 (d, J = 8.1 Hz, 2H), 7.45–7.39 (m, 2H), 7.36 (d, J = 7.6 Hz, 1H), 7.15–7.08 (m, 3H), 6.75 (s, 1H), 6.19 (s, 2H), 3.90–3.82 (overlapping two s and one t, 11H), 3.10 (t, J = 5.3 Hz, 2H), 2.30 (s, 3H); **^{13}C{^{1}H} NMR** (150 MHz, $CDCl_3$) δ 167.2, 160.6, 143.4, 142.2, 139.3, 132.9, 131.9, 131.2, 130.0, 128.6, 126.2, 121.7, 91.7, 85.7, 62.1, 57.1, 56.2, 41.3, 21.3; **HRMS** (FAB) m/z: $[M–OTs]^+$ calcd for $C_{17}H_{20}IO_4$ 415.0401, found 415.0409.

3. Aryne-Mediated Synthesis of Seven-Membered Cyclic Diaryliodoniums

Table S1. Reaction of benzyne with trivalent iodine reagents bearing various dummy ligand[a]

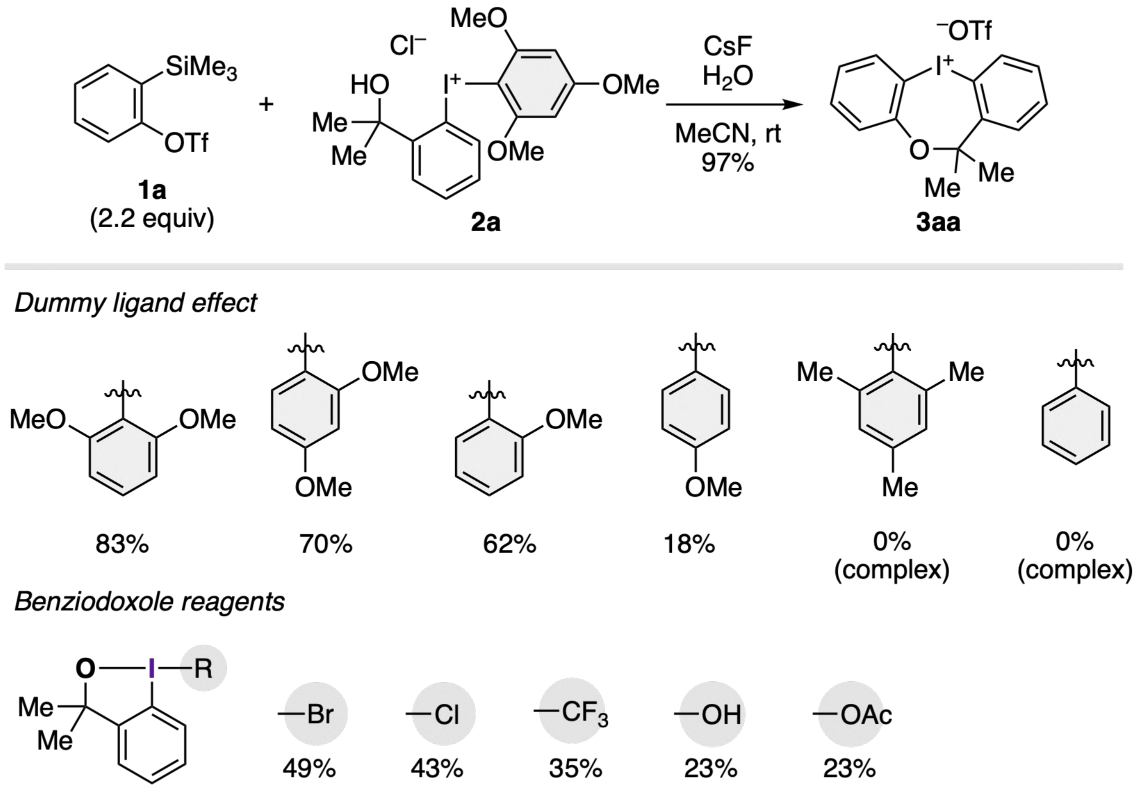

[a]Determined by ^{1}H NMR analysis using 1,3,5-trimethoxybenzene or 1,1,2,2-tetrachloroethane as an internal standard.

Table S2. Investigation of reaction conditions.

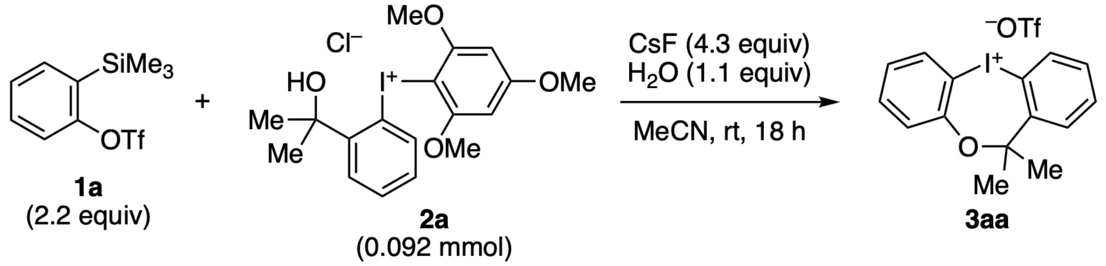

entry	deviation from standard conditions	yield (%)[a]
1	–	quant (97)[b]
2	H_2O (none)	31
3	H_2O (0.5 equiv)	quant
4	H_2O (2.7 equiv)	100
5	H_2O (5.5 equiv)	96
6	H_2O (11 equiv)	58
7	H_2O (1 equiv) was added after the reaction	38
8	**1a**:**2a** = 1:2	61[c]
9	Solvent: CH_2Cl_2	45

[a]Determined by ^{1}H NMR analysis using 1,1,2,2-tetrachloroethane as an internal standard. [b]The isolated yield is shown in the parentheses. [c]The yield is based on **1a**.

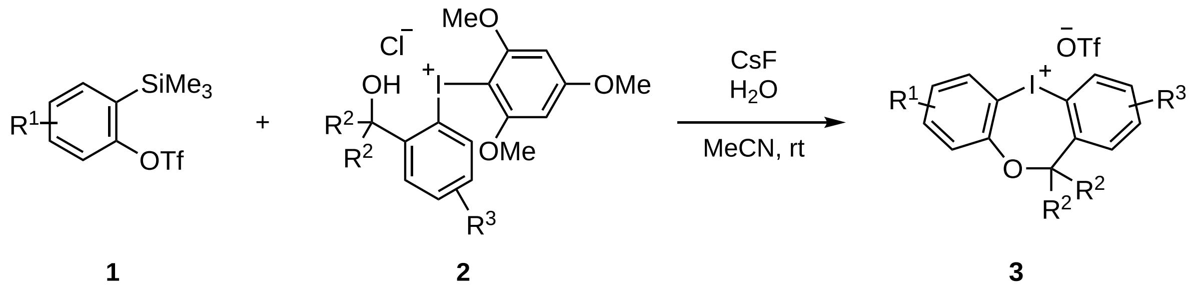

General Procedure (GP4): In a 4 mL vial equipped with a magnetic stir bar, organobenziodoxole **2** (0.092 mmol, 1.0 equiv) and cesium fluoride (60.8 mg, 0.4 mmol, 4.3 equiv) was dissolved in MeCN (0.5 mL). To the solution were added H_2O (0.1 mmol, 1.1 equiv) and *o*-silylaryl triflate **1** (0.20 mmol, 2.2 equiv) at room temperature, and the resulting mixture was stirred for 18 h. The reaction mixture was filtered through a pad of silica gel (eluent: CH_2Cl_2/MeOH = 5/1) and concentrated under reduced pressure. The residue was purified by column chromatography on silica gel (eluent: CH_2Cl_2/MeOH = 40/1) to afford the desired product **3**.

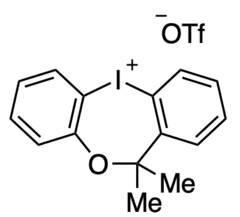

11,11-Dimethyl-11*H*-dibenzo[*b*,*f*][1,4]iodaoxepin-5-ium trifluoromethanesulfonate (3aa): Synthesized according to **GP4** from **1a** (59.7 mg, 0.20 mmol) and **2a** (42.8 mg, 0.092 mmol). Colorless solid (43.2 mg, 97%); R_f 0.20 (CH_2Cl_2/MeOH = 10/1); m.p. 147−148 °C (decomp.); **^{1}H NMR** (400 MHz, CD_3OD) δ 8.12 (d, J = 7.6 Hz, 1H), 8.04 (dd, J = 8.1, 1.5 Hz, 1H), 7.68−7.62 (m, 3H), 7.56 (dd, J = 8.2, 1.5 Hz, 1H), 7.35−7.28 (m, 2H), 1.95 (s, 6H); **^{13}C{^{1}H} NMR** (150 MHz, CD_3OD) δ 153.9, 145.4, 138.9, 135.5, 134.9, 134.3, 132.0, 131.1, 128.6, 126.5, 121.8 (q, $J_{C–F}$ = 316.7 Hz), 120.2, 111.2, 85.6, 30.5; **^{19}F NMR** (376 MHz, CD_3OD) δ –80.0; **HRMS** (FAB) *m*/*z*: $[M–OTf]^+$ calcd for $C_{15}H_{14}IO$ 337.0084, found 337.0092.

The same compound was also obtained from **1a** (59.7 mg, 0.20 mmol) and the tosylate salt **2a-OTs** (60.0 mg, 0.10 mmol) according to **GP4** (38.9 mg, 80%).

Gram-scale synthesis of 3aa: Under an argon atmosphere, a 100 mL flask equipped with a magnetic stir bar was charged with **2a** (2.14 g, 4.6 mmol, 1 equiv), CsF (3.04 g, 20 mmol, 4.3

equiv), and MeCN (25 mL). To the solution were added H_2O (108 μL, 6 mmol, 1.3 equiv) and 2-(trimethylsilyl)phenyl trifluoromethanesulfonate **1a** (3.01 g, 10.1 mmol, 2.2 equiv) at room temperature, and the resulting mixture was stirred for 18 h. The reaction mixture was filtered through a pad of silica gel (eluent: CH_2Cl_2/MeOH = 5/1) and concentrated under reduced pressure. The residue was recrystallized from $CHCl_3$ to afford **3aa** as a colorless solid (1.79 g, 80%).

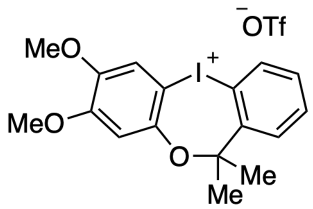

7,8-Dimethoxy-11,11-dimethyl-11*H*-dibenzo[*b*,*f*][1,4]iodaoxepin-5-ium trifluoromethanesulfonate (3ba): Synthesized according to **GP3** from **1b** (71.7 mg, 0.20 mmol) and **2a** (42.8 mg, 0.092 mmol). Colorless solid (45.3 mg, 90%); R_f 0.25 (CH_2Cl_2/MeOH = 10/1); m.p. 115−116 °C (decomp.); **^{1}H NMR** (400 MHz, CD_3OD) δ 8.09 (d, J = 8.3 Hz, 1H), 7.65−7.59 (m, 2H), 7.51 (s, 1H), 7.30 (ddd, J = 8.0, 6.4, 2.5 Hz, 1H), 7.12 (s, 1H), 3.88 (s, 3H), 3.83 (s, 3H), 1.94 (s, 6H); **^{13}C{^{1}H} NMR** (150 MHz, CD_3OD) δ 155.3, 149.2, 148.9, 145.8, 138.6, 134.2, 132.2, 130.8, 120.5 (q, $J_{C–F}$ = 316.7 Hz), 115.9, 111.6, 109.4, 107.5, 85.1, 57.2, 57.0, 30.7; **^{19}F NMR** (376 MHz, CD_3OD) δ –80.1; **HRMS** (FAB) *m*/*z*: [M–OTf]$^+$ calcd for $C_{17}H_{18}IO_3$ 397.0295, found 397.0295.

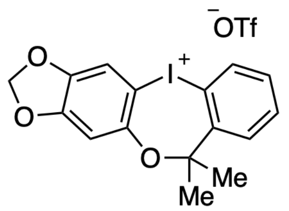

10,10-Dimethyl-10*H*-[1,3]dioxolo[4',5':4,5]benzo[1,2-*b*]benzo[*f*][1,4]iodaoxepin-5-ium trifluoromethanesulfonate (3ca): Synthesized according to **GP3** from **1c** (68.4 mg, 0.20 mmol) and **2a** (42.8 mg, 0.092 mmol). Brown solid (41.3 mg, 85%); R_f 0.20 (CH_2Cl_2/MeOH = 10/1); m.p. 95−96 °C (decomp.); **^{1}H NMR** (400 MHz, CD_3OD) δ 8.09 (d, J = 7.7 Hz, 1H), 7.64−7.61 (m, 2H), 7.43 (s, 1H), 7.31 (ddd, J = 8.9, 4.6, 3.4 Hz, 1H), 7.10 (s, 1H), 6.09 (s, 2H), 1.91 (s, 6H); **^{13}C{^{1}H} NMR** (150 MHz, CD_3OD) δ 154.1, 149.9, 147.5, 145.4, 138.6, 134.3, 132.0, 130.9, 121.8 (q, $J_{C–F}$ = 316.3 Hz), 112.6, 112.1, 108.9, 107.0, 104.8, 85.3, 30.5; **^{19}F**

NMR (376 MHz, CD_3OD) δ –80.1; **HRMS** (FAB) *m/z*: $[M–OTf]^+$ calcd for $C_{16}H_{14}IO_3$ 380.9982, found 380.9971.

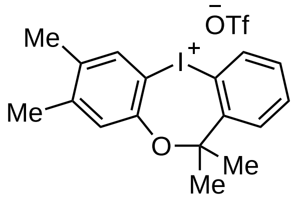

7,8,11,11-Tetramethyl-11*H*-dibenzo[*b*,*f*][1,4]iodaoxepin-5-ium trifluoromethanesulfonate (3da): Synthesized according to **GP3** from **1d** (65.3 mg, 0.20 mmol) and **2a** (42.8 mg, 0.092 mmol). Colorless solid (46.8 mg, 99%); R_f 0.15 (CH_2Cl_2/MeOH = 10/1); m.p. 133–134 °C (decomp.); **^{1}H NMR** (600 MHz, CD_3OD) δ 8.10 (d, *J* = 8.5 Hz, 1H), 7.77 (s, 1H), 7.62–7.59 (m, 2H), 7.33 (s, 1H), 7.31–7.28 (m, 1H), 2.32 (s, 3H), 2.27 (s, 3H), 1.92 (s, 6H); **$^{13}C\{^1H\}$ NMR** (150 MHz, CD_3OD) δ 151.9, 145.7, 145.5, 138.8, 138.1, 134.5, 134.2, 132.1, 130.9, 127.0, 121.8 (q, $J_{C–F}$ = 317.0 Hz), 116.0, 111.3, 85.1, 30.6, 20.1, 19.2; **^{19}F NMR** (376 MHz, CD_3OD) δ –79.9; **HRMS** (FAB) *m/z*: $[M–OTf]^+$ calcd for $C_{17}H_{18}IO$ 365.0397, found 365.0398.

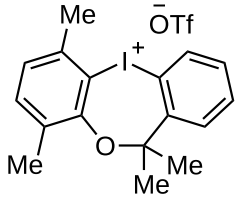

6,9,11,11-Tetramethyl-11*H*-dibenzo[*b*,*f*][1,4]iodaoxepin-5-ium trifluoromethanesulfonate (3ea): Synthesized according to **GP3** from **1e** (65.3 mg, 0.20 mmol) and **2a** (42.8 mg, 0.092 mmol). The crude residue was purified by column chromatography on silica gel (eluent: CH_2Cl_2/MeOH = 40/1), followed by recrystallization from $CHCl_3$ to afford the title compound as a colorless solid (7.9 mg, 17%); R_f 0.21 (CH_2Cl_2/MeOH = 10/1); m.p. 140–141 °C (decomp.); **^{1}H NMR** (400 MHz, CD_3OD) δ 8.18 (d, *J* = 8.0 Hz, 1H), 7.70–7.60 (m, 2H), 7.40 (d, *J* = 7.7 Hz, 1H), 7.35 (t, *J* = 7.4 Hz, 1H), 7.19 (d, *J* = 7.8 Hz, 1H), 2.58 (s, 3H), 2.45 (s, 3H), 1.94 (s, 6H); **$^{13}C\{^1H\}$ NMR** (150 MHz, CD_3OD) δ 153.5, 145.6, 140.1, 138.4, 137.3, 134.2, 132.8, 132.0, 131.3, 128.4, 123.8, 121.8 (q, $J_{C–F}$ = 316.6 Hz), 110.1, 88.0, 30.9, 24.5, 18.8; **^{19}F NMR** (376 MHz, CD_3OD) δ –80.1; **HRMS** (FAB) *m/z*: $[M–OTf]^+$ calcd for $C_{17}H_{18}IO$, 365.0397, found 365.0414.

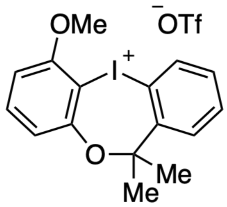

6-Methoxy-11,11-dimethyl-11*H*-dibenzo[*b*,*f*][1,4]iodaoxepin-5-ium trifluoromethanesulfonate (3fa): Synthesized according to **GP3** from **1f** (65.7 mg, 0.20 mmol) and **2a** (42.8 mg, 0.092 mmol). Colorless solid (45.2 mg, 95%); R_f 0.29 (CH_2Cl_2/MeOH = 10/1); m.p. 120−121 °C (decomp.); **^{1}H NMR** (400 MHz, CD_3OD) δ 8.09 (d, *J* = 7.8 Hz, 1H), 7.62−7.55 (m, 3H), 7.32−7.26 (m, 1H), 7.10 (dd, *J* = 8.1, 0.9 Hz, 1H), 7.04 (dd, *J* = 8.5 Hz, 1H), 3.97 (s, 3H), 1.94 (s, 6H); **^{13}C{^{1}H} NMR** (150 MHz, CD_3OD) δ 159.4, 155.3, 145.5, 138.8, 136.3, 134.3, 132.0, 131.0, 121.8 (q, $J_{C–F}$ = 316.7 Hz), 118.3, 111.5, 110.3, 109.1, 85.5, 57.6, 30.6; **^{19}F NMR** (376 MHz, CD_3OD) δ –80.1; **HRMS** (FAB) *m/z*: [M–OTf]$^+$ calcd for $C_{16}H_{16}IO_2$ 367.0190, found 367.0200.

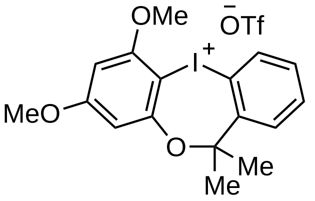

6,8-Dimethoxy-11,11-dimethyl-11*H*-dibenzo[*b*,*f*][1,4]iodaoxepin-5-ium trifluoromethanesulfonate (3ga): Synthesized according to **GP3** from **1g** (71.7 mg, 0.20 mmol) and **2a** (42.8 mg, 0.092 mmol). Colorless solid (41.5 mg, 82%); R_f 0.14 (CH_2Cl_2/MeOH = 10/1); m.p. 94–95 °C (decomp.); **^{1}H NMR** (600 MHz, CD_3OD) δ 8.06 (d, *J* = 8.2 Hz, 1H), 7.65–7.58 (m, 2H), 7.31–7.27 (m, 1H), 6.66 (d, *J* = 2.4 Hz, 1H), 6.58 (d, *J* = 2.3 Hz, 1H), 3.94 (s, 3H), 3.86 (s, 3H), 1.94 (s, 6H); **^{13}C{^{1}H} NMR** (150 MHz, CD_3OD) δ 167.4, 160.1, 156.4, 145.5, 138.5, 134.1, 132.0, 130.8, 121.8 (q, $J_{C–F}$ = 316.9 Hz), 112.0, 104.4, 98.5, 97.3, 85.6, 57.5, 56.8, 30.7; **^{19}F NMR** (565 MHz, CD_3OD) δ –80.0; **HRMS** (FAB) *m/z*: [M–OTf]$^+$ calcd for $C_{17}H_{18}IO_3$ 397.0295, found 397.0293.

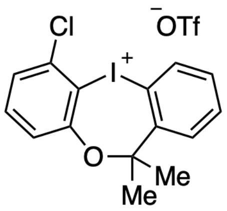

6-Chloro-11,11-dimethyl-11*H*-dibenzo[*b*,*f*][1,4]iodaoxepin-5-ium trifluoromethanesulfonate (3ha): Synthesized according to **GP3** from **1h** (66.6 mg, 0.20 mmol) and **2a** (42.8 mg, 0.092 mmol). Colorless solid (19.7 mg, 41%); R_f 0.14 (CH_2Cl_2/MeOH = 10/1); m.p. 157−158 °C (decomp.); ^{1}**H NMR** (600 MHz, CD_3OD) δ 8.18 (d, *J* = 7.7 Hz, 1H), 7.68−7.64 (m, 2H), 7.62 (t, *J* = 8.2 Hz, 1H), 7.51 (dd, *J* = 8.2, 1.1 Hz, 1H), 7.48 (dd, *J* = 8.1, 1.2 Hz, 1H), 7.38−7.33 (m, 1H), 1.97 (s, 6H); ^{13}**C{^{1}H} NMR** (150 MHz, CD_3OD) δ 155.8, 144.8, 139.0, 137.0, 136.2, 134.7, 132.1, 131.5, 128.0, 124.7, 123.7, 121.8 (q, $J_{C\text{–}F}$ = 316.7 Hz), 112.5, 86.4, 30.4; ^{19}**F NMR** (565 MHz, CD_3OD) δ –80.0; **HRMS** (FAB) *m*/*z*: $[M\text{–}OTf]^+$ calcd for $C_{16}H_{16}IO_2$ 370.9694, found 370.9705.

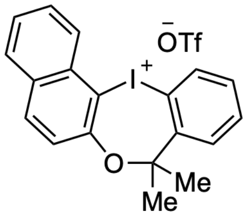

8,8-Dimethyl-8*H*-benzo[*f*]naphtho[1,2-*b*][1,4]iodaoxepin-13-ium trifluoromethanesulfonate (3ia): Synthesized according to **GP3** from **1i** (69.7 mg, 0.20 mmol) and **2a** (42.8 mg, 0.092 mmol). Colorless solid (40.0 mg, 81%); m.p. 107−108 °C (decomp.); R_f 0.19 (CH_2Cl_2/MeOH = 10/1); ^{1}**H NMR** (400 MHz, CD_3OD) δ 8.23−8.16 (m, 2H), 8.00 (d, *J* = 8.3 Hz, 1H), 7.97 (d, *J* = 8.4 Hz, 1H), 7.79−7.75 (m, 1H), 7.69 (d, *J* = 8.7 Hz, 1H), 7.66−7.57 (m, 3H), 7.30−7.24 (m, 1H), 2.04 (s, 6H); ^{13}**C{^{1}H} NMR** (150 MHz, CD_3OD) δ 153.8, 145.3, 138.7, 135.9, 134.5, 133.5, 132.9, 132.0, 131.0, 130.8, 130.0, 129.1, 128.2, 124.2, 121.8 (q, $J_{C\text{–}F}$ = 316.3 Hz), 120.5, 111.7, 86.0, 30.8; ^{19}**F NMR** (376 MHz, CD_3OD) δ –80.1; **HRMS** (FAB) *m*/*z*: $[M\text{–}OTf]^+$ calcd for $C_{19}H_{16}IO$ 387.0240, found 387.0240.

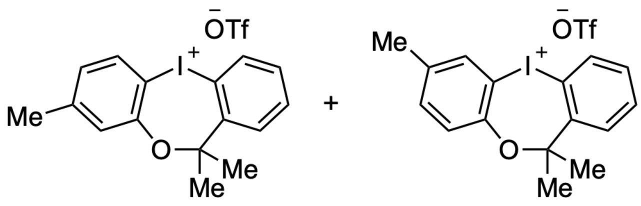

3ja **3ja'**

8,11,11-Trimethyl-11*H*-dibenzo[*b*,*f*][1,4]iodaoxepin-5-ium trifluoromethanesulfonate (3ja) and 7,11,11-trimethyl-11*H*-dibenzo[*b*,*f*][1,4]iodaoxepin-5-ium trifluoromethanesulfonate (3ja'): Synthesized according to **GP3** from **1j** (62.5 mg, 0.20 mmol) and **2a** (42.8 mg, 0.092 mmol). Colorless solid (45.8 mg, 99%, ratio = 1.4:1 as determined by ^{1}H NMR); R_f 0.16 (CH_2Cl_2/MeOH = 10/1); m.p. 98–99 °C (decomp.); **^{1}H NMR** (600 MHz, $CDCl_3$, **3ja**) δ 8.19 (d, J = 8.1 Hz, 1H), 7.95 (d, J = 8.5 Hz, 1H), 7.48 (t, J = 7.7 Hz, 1H), 7.38–7.35 (m, 1H), 7.23–7.17 (m, 1H), 7.12 (d, J = 1.2 Hz, 1H), 7.01 (d, J = 7.2 Hz, 1H), 2.38 (s, 3H), 1.90 (s, 6H); **^{1}H NMR** (600 MHz, $CDCl_3$, **3ja'**) δ 8.19 (d, J = 8.1 Hz, 1H), 7.90 (s, 1H), 7.48 (t, J = 7.7 Hz, 1H), 7.38–7.35 (m, 1H), 7.30–7.26 (m, 1H), 7.23–7.17 (m, 2H), 2.33 (s, 3H), 1.88 (s, 6H); **^{13}C{^{1}H} NMR** (150 MHz, $CDCl_3$, mixture of **3ja** and **3ja'**) δ 152.5, 150.2, 145.3, 144.3, 144.2, 138.6, 138.5, 138.0, 134.64, 134.57, 134.1, 132.78, 132.77, 130.24, 130.19, 130.00, 129.99, 128.3, 125.7, 124.4, 120.4 (q, $J_{C\text{–}F}$ = 318.0 Hz), 118.7, 115.2, 110.6, 110.3, 83.9, 83.7, 30.6, 21.7, 20.8, one carbon could not be found due to overlapping; **^{19}F NMR** (565 MHz, $CDCl_3$, mixture of **3ja** and **3ja'**) δ –78.0; **HRMS** (FAB) *m/z*: [M–OTf]$^+$ calcd for $C_{16}H_{16}IO$ 351.0240, found 351.0257.

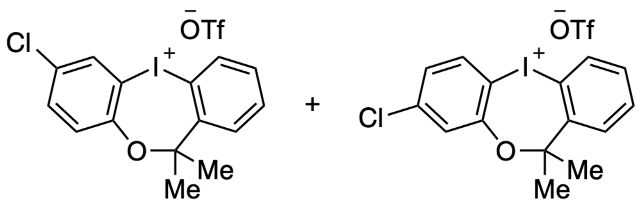

3ka **3ka'**

7-Chloro-11,11-dimethyl-11*H*-dibenzo[*b*,*f*][1,4]iodaoxepin-5-ium trifluoromethanesulfonate (3ka) and 8-chloro-11,11-dimethyl-11*H*-dibenzo[*b*,*f*][1,4]iodaoxepin-5-ium trifluoromethanesulfonate (3ka'): Synthesized according to **GP3** from **1k** (66.6 mg, 0.20 mmol) and **2a** (42.8 mg, 0.092 mmol). Colorless solid (42.1 mg, 88%, ratio = 1.9:1 as determined by ^{1}H NMR); R_f 0.20 (CH_2Cl_2/MeOH = 10/1); m.p. 117–118 °C (decomp.); **^{1}H NMR** (600 MHz, CD_3OD, **3ka**) δ 8.16–8.12 (m, 1H), 8.10 (d, J = 2.4 Hz, 1H), 7.67–7.62 (m, 3H), 7.54 (d, J = 8.8 Hz, 1H), 7.38–7.31 (m, 1H), 1.95 (s, 6H);

^{1}**H NMR** (600 MHz, CD_3OD, **3ka'**) δ 8.16–8.12 (m, 1H), 8.03 (d, J = 8.8 Hz, 1H), 7.67–7.62 (m, 3H), 7.38–7.31 (m, 2H), 1.96 (s, 6H); ^{13}**C{^{1}H} NMR** (150 MHz, CD_3OD, mixture of **3ka** and **3ka'**) δ 154.9, 152.9, 145.10, 145.07, 141.1, 139.0, 138.9, 135.6, 135.4, 134.5, 134.4, 134.2, 132.8, 132.0, 131.3, 128.8, 127.1, 126.8, 121.8 (q, $J_{C–F}$ = 316.7 Hz), 120.7, 118.4, 111.9, 111.7, 86.6, 86.1, 30.4, some carbon signals could not be found due to overlapping; ^{19}**F NMR** (565 MHz, CD_3OD, mixture of **3ka** and **3ka'**) δ –79.9; **HRMS** (FAB) $[M–OTf]^+$ calcd for $C_{15}H_{13}ClIO$ 370.9694, found 370.9707.

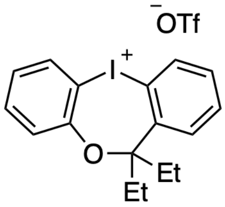

11,11-Diethyl-11*H*-dibenzo[*b*,*f*][1,4]iodaoxepin-5-ium trifluoromethanesulfonate (3ab): Synthesized according to **GP3** from **1a** (59.7 mg, 0.20 mmol) and **2b** (45.5 mg, 0.092 mmol). Colorless solid (37.6 mg, 79%); R_f 0.30 (CH_2Cl_2/MeOH = 10/1); m.p. 148−149 °C (decomp.); ^{1}**H NMR** (600 MHz, CD_3OD) δ 8.16 (d, J = 8.3 Hz, 1H), 8.04 (d, J = 8.2 Hz, 1H), 7.66−7.61 (m, 2H), 7.56 (d, J = 7.6 Hz, 1H), 7.49 (d, J = 8.3 Hz, 1H), 7.33 (t, J = 7.6 Hz, 1H), 7.28 (t, J = 7.6 Hz, 1H), 2.48−2.40 (m, 2H), 2.18−2.10 (m, 2H), 1.00 (t, J = 7.5 Hz, 1H); ^{13}**C{^{1}H} NMR** (150 MHz, CD_3OD) δ 154.1, 142.8, 139.1, 135.7, 134.9, 133.7, 132.9, 131.2, 128.4, 126.0, 121.8 (q, $J_{C–F}$ = 318.0 Hz), 120.2, 113.6, 91.4, 31.5, 8.6; ^{19}**F NMR** (376 MHz, CD_3OD) δ –80.1; **HRMS** (FAB) m/z: $[M–OTf]^+$ calcd for $C_{17}H_{18}IO$ 365.0397, found 365.0400.

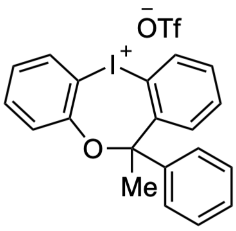

11-Methyl-11-phenyl-11*H*-dibenzo[*b*,*f*][1,4]iodaoxepin-5-ium trifluoromethanesulfonate (3ac): Synthesized according to **GP3** from **1a** (59.7 mg, 0.20 mmol) and **2c** (49.0 mg, 0.093 mmol). Colorless solid (42.8 mg, 84%); R_f 0.23 (CH_2Cl_2/MeOH = 10/1); m.p. 102–103 °C (decomp.); ^{1}**H NMR** (400 MHz, CD_3OD) δ 8.15 (dd, J = 8.1, 1.2 Hz, 1H), 8.06 (dd, J = 8.1, 1.4 Hz, 1H), 7.65–7.59 (m, 1H), 7.55–7.46 (m, 2H), 7.40–7.33 (m, 6H), 7.31–7.25 (m, 1H), 6.92 (d, J = 8.0 Hz, 1H), 2.35 (s, 3H); ^{13}**C{^{1}H} NMR** (150 MHz, CD_3OD) δ 153.9, 144.3, 143.6, 138.7, 135.1, 134.8, 134.1, 133.7, 131.4, 129.7, 129.5, 128.7, 128.6, 126.9, 121.8 (q, $J_{C–F}$ =

316.9 Hz), 119.6, 112.5, 89.0, 30.2; **^{19}F NMR** (376 MHz, CD_3OD) δ –80.0; **HRMS** (FAB) *m/z*: $[M–OTf]^+$ calcd for $C_{20}H_{16}IO$ 399.0240, found 399.0237.

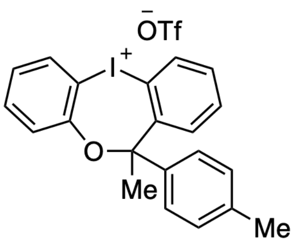

11-Methyl-11-(*p*-tolyl)-11*H*-dibenzo[*b*,*f*][1,4]iodaoxepin-5-ium trifluoromethanesulfonate (3ad): Synthesized according to **GP3** from **1a** (59.7 mg, 0.20 mmol) and **2d** (50.4 mg, 0.093 mmol). Colorless solid (50.5 mg, 96%); R_f 0.17 (CH_2Cl_2/MeOH = 10/1); m.p. 110–111 °C (decomp.); **^{1}H NMR** (400 MHz, CD_3OD) δ 8.14 (d, *J* = 8.0 Hz, 1H), 8.05 (d, *J* = 8.0 Hz, 1H), 7.63–7.57 (m, 1H), 7.51–7.44 (m, 2H), 7.38–7.33 (m, 1H), 7.30–7.25 (m, 1H), 7.24–7.16 (m, 4H), 6.82 (d, *J* = 8.0 Hz, 1H), 2.35 (s, 3H), 2.34 (s, 3H); **^{13}C{^{1}H} NMR** (101 MHz, CD_3OD) δ 154.0, 144.8, 140.5, 140.0, 138.6, 135.1, 134.8, 134.2, 133.6, 131.4, 130.0, 128.7, 128.6, 127.0, 120.5 (q, $J_{C–F}$ = 318.9 Hz), 119.4, 112.1, 89.0, 30.4, 21.0; **^{19}F NMR** (376 MHz, CD_3OD) δ –80.0; **HRMS** (FAB) *m/z*: $[M–OTf]^+$ calcd for $C_{21}H_{18}IO$ 413.0397, found 413.0411.

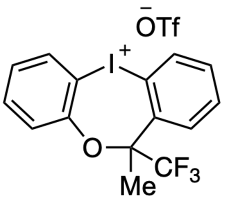

11-Methyl-11-(trifluoromethyl)-11*H*-dibenzo[*b*,*f*][1,4]iodaoxepin-5-ium trifluoromethanesulfonate (3ae): Synthesized according to **GP3** from **1a** (59.7 mg, 0.20 mmol) and **2e** (48.2 mg, 0.10 mmol). Colorless solid (39.6 mg, 73%); m.p. 180−181 °C (decomp.); R_f 0.20 (CH_2Cl_2/MeOH = 10/1); **^{1}H NMR** (600 MHz, CD_3OD) δ 8.26 (dd, *J* = 8.0, 1.3 Hz, 1H), 8.11 (dd, *J* = 8.1, 1.4 Hz, 1H), 7.79 (d, *J* = 7.7 Hz, 1H), 7.76−7.67 (m, 3H), 7.52−7.48 (m, 1H), 7.43−7.39 (m, 1H), 2.22 (s, 3H); **^{13}C{^{1}H} NMR** (150 MHz, CD_3OD) δ 151.2, 139.6, 135.9, 135.4, 135.2, 134.6, 134.3, 133.3, 129.9, 126.5, 125.3 (q, $J_{C–F}$ = 282.7 Hz), 121.8 (q, $J_{C–F}$ = 316.4 Hz), 119.3, 113.8, 85.8 (q, $J_{C–F}$ = 29.2 Hz), 21.3; **^{19}F NMR** (376 MHz, CD_3OD) δ –80.0, –80.8; **HRMS** (FAB) *m/z*: $[M–OTf]^+$ calcd for $C_{15}H_{11}F_3IO$ 390.9801, found 390.9805.

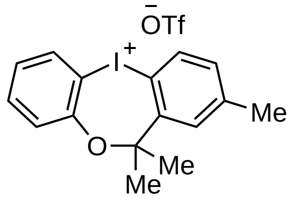

2,11,11-Trimethyl-11*H*-dibenzo[*b*,*f*][1,4]iodaoxepin-5-ium trifluoromethanesulfonate (3af): Synthesized according to **GP3** from **1a** (59.7 mg, 0.20 mmol) and **2f** (44.2 mg, 0.092 mmol). Colorless solid (44.0 mg, 95%); R_f 0.21 (CH_2Cl_2/MeOH = 10/1); m.p. 149–150 °C (decomp.); **^{1}H NMR** (600 MHz, CD_3OD) δ 8.03 (dd, J = 8.1, 1.4 Hz, 1H), 7.98 (d, J = 8.1 Hz, 1H), 7.66–7.62 (m, 1H), 7.53 (dd, J = 8.2, 1.4 Hz, 1H), 7.43 (d, J = 1.9 Hz, 1H), 7.31–7.27 (m, 1H), 7.13 (dd, J = 7.9, 1.7 Hz, 1H), 2.37 (s, 3H), 1.94 (s, 6H); **^{13}C{^{1}H} NMR** (101 MHz, CD_3OD) δ 154.0, 145.5, 145.2, 138.7, 135.4, 134.7, 132.5, 131.8, 128.5, 126.5, 121.8 (q, $J_{C\text{–}F}$ = 318.9 Hz), 120.3, 107.6, 85.6, 30.5, 21.2; **^{19}F NMR** (376 MHz, CD_3OD) δ –80.0; **HRMS** (FAB) *m*/*z*: [M–OTf]$^+$ calcd for $C_{16}H_{16}IO$, 351.0240, found 351.0261.

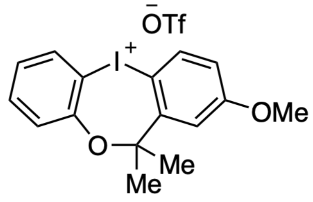

2-Methoxy-11,11-dimethyl-11*H*-dibenzo[*b*,*f*][1,4]iodaoxepin-5-ium trifluoromethanesulfonate (3ag): Synthesized according to **GP3** from **1a** (59.7 mg, 0.20 mmol) and **2g** (45.8 mg, 0.093 mmol). Colorless solid (45.6 mg, 95%); m.p. 143−144 °C (decomp.); R_f 0.10 (CH_2Cl_2/MeOH = 10/1); **^{1}H NMR** (400 MHz, CD_3OD) δ 8.02 (dd, J = 8.1, 1.4 Hz, 1H), 8.01 (d, J = 8.9 Hz, 1H), 7.67−7.61 (m, 1H), 7.54 (dd, J = 8.1, 1.4 Hz, 1H), 7.31−7.26 (m, 1H), 7.08 (d, J = 3.0 Hz, 1H), 6.90 (dd, J = 8.9, 3.0 Hz, 1H), 3.82 (s, 3H), 1.94 (s, 6H); **^{13}C{^{1}H} NMR** (150 MHz, CD_3OD) δ 164.6, 154.0, 147.3, 140.3, 135.4, 134.6, 128.4, 126.5, 121.8 (q, $J_{C\text{–}F}$ = 318.0 Hz), 120.7, 118.4, 115.8, 100.0, 85.7, 56.4, 30.5; **^{19}F NMR** (376 MHz, CD_3OD) δ –80.0; **HRMS** (FAB) *m*/*z*: [M–OTf]$^+$ calcd for $C_{16}H_{16}IO_2$ 367.0190, found 367.0211.

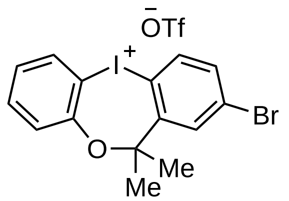

2-Bromo-11,11-dimethyl-11*H*-dibenzo[*b*,*f*][1,4]iodaoxepin-5-ium trifluoromethanesulfonate (3ah): Synthesized according to **GP3** from **1a** (59.7 mg, 0.20 mmol) and **2h** (50.6 mg, 0.093 mmol). Colorless solid (45.2 mg, 86%); R_f 0.13 (CH_2Cl_2/MeOH = 10/1); m.p. 114–115 °C (decomp.); **^{1}H NMR** (400 MHz, CD_3OD) δ 8.07–8.00 (m, 2H), 7.79 (d, J = 2.3 Hz, 1H), 7.70–7.64 (m, 1H), 7.56 (dd, J = 8.2, 1.4 Hz, 1H), 7.51 (dd, J = 8.5, 2.3 Hz, 1H), 7.35–7.29 (m, 1H), 1.94 (s, 6H); **^{13}C{^{1}H} NMR** (150 MHz, CD_3OD) δ 153.8, 147.7, 140.2, 135.7, 135.1, 134.9, 134.7, 134.2, 128.8, 126.6, 121.8 (q, $J_{C–F}$ = 316.8 Hz), 120.2, 109.7, 85.3, 30.4 **^{19}F NMR** (376 MHz, CD_3OD) δ –80.1; **HRMS** (FAB) *m*/*z*: $[M–OTf]^+$ calcd for $C_{15}H_{13}BrIO$, 414.9189, found 414.9187.

4. Transformations of Cyclic Diaryliodonium Salts

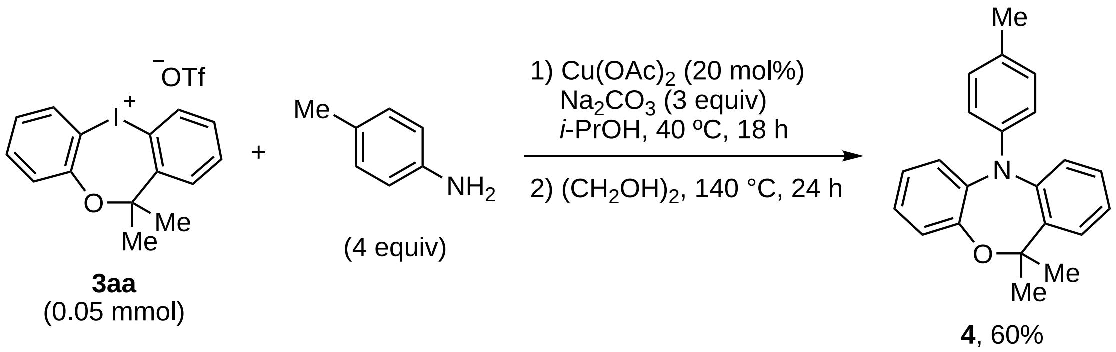

3aa (0.05 mmol) **4**, 60%

11,11-Dimethyl-5-(*p*-tolyl)-5,11-dihydrodibenzo[*b*,*e*][1,4]oxazepine (4): Under an argon atmosphere, an oven-dried Schlenk tube equipped with a magnetic stir bar was charged with cyclic diaryliodonium triflate **3aa** (24.3 mg, 0.05 mmol, 1.0 equiv) and *i*-PrOH (0.9 mL). To the solution was added $Cu(OAc)_2$ (1.8 mg, 10.0 μmol, 20 mol%), *p*-toluidine (21.4 mg, 0.2 mmol, 4 equiv), and Na_2CO_3 (15.9 mg, 0.15 mmol, 3 equiv). The mixture was warmed to 40 °C and stirred for 18 h, then ethylene glycol (0.1 mL) was added. The reaction mixture was then heated to 140 °C and stirred for 24 h. After cooling to room temperature, the mixture was concentrated under reduced pressure. The residue was extracted with EtOAc (5 mL × 3), washed with brine, dried over Na_2SO_4, and concentrated under reduced pressure. The residue was purified by flash chromatography on silica gel (eluent: hexane/EtOAc = 100/1), followed by PTLC, to afford the desired product **4** as a colorless solid (9.4 mg, 60%).

R_f 0.49 (hexane/EtOAc= 10/1); m.p. 154−155 °C; **^{1}H NMR** (400 MHz, $CDCl_3$) δ 7.41 (dd, *J* = 7.6, 1.6 Hz, 1H), 7.39−7.35 (m, 1H), 7.25−7.16 (m, 4H), 7.12−7.06 (m, 2H), 6.93 (d, *J* = 8.0 Hz, 2H), 6.61 (d, *J* = 8.7 Hz, 2H), 2.23 (s, 3H), 1.65 (s, 6H); **^{13}C{^{1}H} NMR** (150 MHz, $CDCl_3$) δ 152.6, 146.8, 141.8, 141.0, 140.2, 130.3, 129.63, 129.57, 128.9, 128.0, 127.9, 127.5, 126.3, 124.6, 123.8, 114.3, 81.9, 30.9, 20.5; **HRMS** (EI) *m*/*z*: $[M]^+$ calcd for $C_{22}H_{21}NO$ 315.1623, found 315.1619.

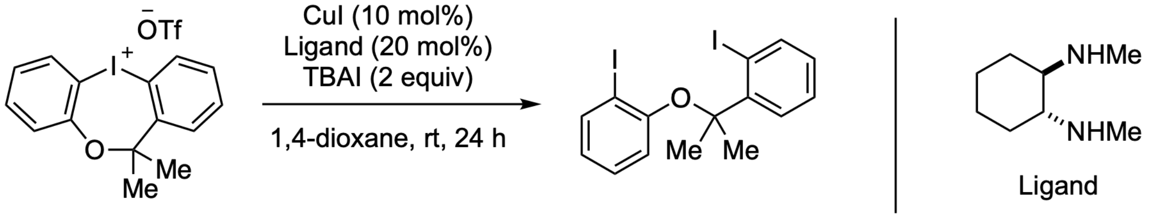

3aa (0.2 mmol) **I-5**, 86%

1-Iodo-2-(2-(2-iodophenoxy)propan-2-yl)benzene (I-5): Under an argon atmosphere, an oven-dried Schlenk tube was charged with cyclic diaryliodonium triflate **3aa** (97.2 mg, 0.2

mmol, 1.0 equiv), CuI (3.8 mg, 20.0 μmol, 10 mol%), and tetrabutylammonium iodide (147 mg, 0.4 mmol, 2 equiv). The Schlenk tube was evacuated and backfilled with argon, followed by the addition of 1,4-dioxane (2.0 mL) and *trans*-*N*,*N'*-dimethylcyclohexane-1,2-diamine (5.69 mg, 40.0 μmol, 20 mol%). The reaction mixture was stirred at room temperature for 24 h. The mixture was diluted with EtOAc (5 mL) and H_2O (5 mL), and the aqueous layer was extracted with EtOAc (5 × 3 mL). The combined organic layer was dried over $MgSO_4$ and concentrated under reduced pressure. The residue was purified by flash chromatography on silica gel (eluent: hexane) to afford the desired product **I-5** as a colorless solid (80.2 mg, 86%). R_f 0.14 (hexane); m.p. 101−102 °C; **^{1}H NMR** (600 MHz, $CDCl_3$) δ 8.01 (dd, *J* = 8.3, 1.4 Hz, 1H), 7.77 (dd, *J* = 7.9, 1.7 Hz, 1H), 7.45 (dd, *J* = 7.9, 1.7 Hz, 1H), 7.39−7.35 (m, 1H), 6.97−6.91 (m, 2H), 6.62 (td, *J* = 6.9, 1.4 Hz, 1H), 6.21 (dd, *J* = 8.3, 1.4 Hz, 1H), 1.91 (s, 6H); **^{13}C{^{1}H} NMR** (150 MHz, $CDCl_3$) δ 154.8, 146.0, 143.7, 139.6, 129.1 128.7, 128.4, 127.3, 122.9, 117.0, 93.1, 91.0, 82.2, 28.5; **HRMS** (EI) *m*/*z*: $[M]^+$ calcd for $C_{15}H_{14}I_2O$ 463.9134, found 463.9135.

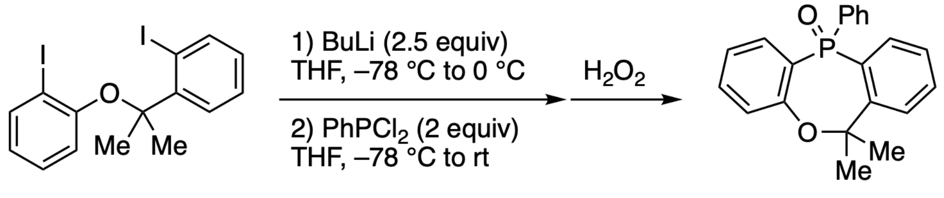

I-5
(0.10 mmol)

5, 56%

11,11-Dimethyl-5-phenyl-11*H*-dibenzo[*b*,*e*][1,4]oxaphosphepine 5-oxide (5): Under an argon atmosphere, an oven-dried Schlenk tube equipped with a magnetic stir bar was charged with 1-iodo-2-(2-(2-iodophenoxy)propan-2-yl)benzene (**I-5**; 46.4 mg, 0.10 mmol, 1.0 equiv) and THF (1.0 mL). To the solution was slowly added BuLi (1.54 M in hexane, 0.16 mL, 0.25 mmol, 2.5 equiv) at –78 °C, and stirred for 30 min. The mixture was warmed to 0 °C, stirred for 1 h, and then cooled again to –78 °C. Dichlorophenylphosphine (27.1 μL, 0.20 mmol, 2 equiv) was added, and the reaction mixture was warmed to room temperature and stirred for 12 h. An aqueous solution of H_2O_2 (ca. 30%, a few drops) was added at 0 °C, and the resulting mixture was stirred at room temperature for 0.5 h. After quenching with sat. NH_4Cl aq., the mixture was extracted with EtOAc (5 mL × 3). The combined organic layer was washed with brine, dried over $MgSO_4$, and concentrated under reduced pressure. The residue was purified by flash chromatography on silica gel (eluent: hexane/EtOAc/CH_2Cl_2 = 2/1/2), followed by GPC, to afford the desired product **5** as a colorless oil (18.8 mg, 56%).

m.p. 192−193 °C; R_f 0.12 (hexane/EtOAc = 2/1); **^{1}H NMR** (400 MHz, $CDCl_3$) δ 8.51 (ddd, *J* = 13.1, 7.7, 1.4 Hz, 1H), 8.12 (ddd, *J* = 11.6, 7.5, 1.7 Hz, 1H), 7.58−7.31 (m, 9H), 7.24−7.19 (m, 1H), 7.06 (dd, *J* = 8.0, 4.8 Hz, 1H), 1.59 (s, 6H); **^{13}C{^{1}H} NMR** (150 MHz, $CDCl_3$) δ 155.7 (d, J_{C-P} = 5.0 Hz), 149.5 (d, J_{C-P} = 7.9 Hz), 136.3 (d, J_{C-P} = 109.9 Hz), 133.8 (d, J_{C-P} = 2.1 Hz), 133.4 (d, J_{C-P} = 6.1 Hz), 132.8 (d, J_{C-P} = 5.4 Hz), 132.5 (d, J_{C-P} = 2.5 Hz), 131.3 (d, J_{C-P} = 2.9 Hz), 130.3 (d, J_{C-P} = 108.1 Hz), 130.2 (d, J_{C-P} = 11.1 Hz), 128.3 (d, J_{C-P} = 12.9 Hz), 127.7 (d, J_{C-P} = 10.8 Hz), 127.6 (d, J_{C-P} = 99.1 Hz), 127.1 (d, J_{C-P} = 10.8 Hz), 125.4 (d, J_{C-P} = 11.1 Hz), 125.0 (d, J_{C-P} = 7.2 Hz), 83.6 (d, J_{C-P} = 1.4 Hz), 33.5, 29.5; **^{31}P NMR** (162 MHz, $CDCl_3$) δ 24.9; **HRMS** (EI) *m*/*z*: $[M]^+$ calcd for $C_{21}H_{19}O_2P$ 334.1123, found 334.1109.

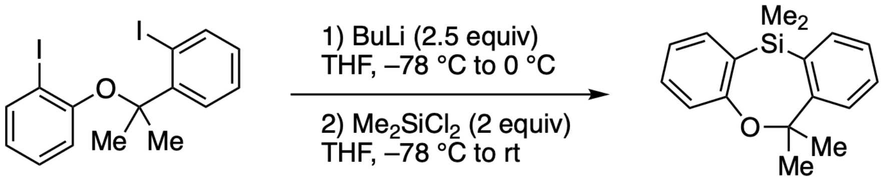

I-5
(0.10 mmol)

6, 72%

5,5,11,11-Tetramethyl-5,11-dihydrodibenzo[*b*,*e*][1,4]oxasilepine (6): Under an argon atmosphere, an oven-dried Schlenk tube equipped with a magnetic stir bar was charged with 1-iodo-2-(2-(2-iodophenoxy)propan-2-yl)benzene (**I-5**; 46.4 mg, 0.10 mmol, 1.0 equiv) and THF (1.0 mL). To the solution was slowly added BuLi (1.54 M in hexane, 0.16 mL, 0.25 mmol, 2.5 equiv) at –78 °C, and stirred for 30 min. The mixture was warmed to 0 °C and stirred for 1 h, and then cooled again to –78 °C. Dichlorodimethylsilane (23.9 μL, 0.2 mmol, 2 equiv) was added, and the reaction mixture was warmed to room temperature and stirred for 12 h. After quenching with sat. NH_4Cl aq., the resulting mixture was extracted with EtOAc (5 mL × 3). The combined organic layer was washed with brine, dried over $MgSO_4$, and concentrated under reduced pressure. The residue was purified by flash chromatography on silica gel (eluent: hexane/EtOAc = 100/1) to afford the desired product **6** as a colorless oil (19.2 mg, 72%).

R_f 0.30 (hexane/EtOAc = 100/1); **^{1}H NMR** (600 MHz, $CDCl_3$) δ 7.60 (dd, *J* = 7.4, 1.4 Hz, 1H), 7.42 (dd, *J* = 7.1, 1.6 Hz, 1H), 7.34−7.29 (m, 2H), 7.42 (td, *J* = 7.4, 1.1 Hz, 1H), 7.17 (d, *J* = 7.9 Hz, 1H), 7.11 (t, *J* = 6.9 Hz, 1H), 7.04 (d, *J* = 7.9 Hz, 1H), 1.69 (s, 6H), 0.58 (s, 6H); **^{13}C{^{1}H} NMR** (150 MHz, $CDCl_3$) δ 160.6, 153.7, 136.4, 134.3, 134.0, 133.2, 130.6, 129.5, 126.7, 125.9, 124.3, 123.8, 83.7, 31.9, –1.2; **HRMS** (EI) *m*/*z*: $[M]^+$ calcd for $C_{17}H_{20}OSi$ 268.1283, found 268.1274.

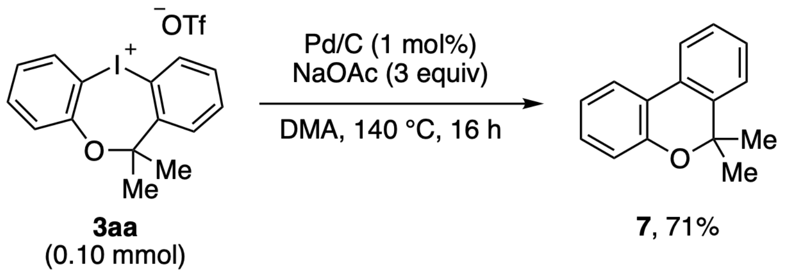

6,6-Dimethyl-6*H*-benzo[*c*]chromene (7): [15] Under an argon atmosphere, an oven-dried Schlenk tube equipped with a magnetic stir bar was charged with cyclic diaryliodonium triflate **3aa** (48.6 mg, 0.10 mmol, 1.0 equiv) and *N*,*N*-dimethylacetamide (DMA, 2 mL). To the solution was added palladium on carbon (10 wt% Pd/C, 1.1 mg, 1.0 µmol of Pd, 1 mol%), and NaOAc (24.6 mg, 0.30 mmol, 3 equiv). The mixture was warmed to 140 °C and stirred for 16 h. After quenching with H_2O (5 mL), the mixture was extracted with Et_2O (10 mL × 3). The combined organic layer was washed with brine, dried over $MgSO_4$, and concentrated under reduced pressure. The residue was purified by flash chromatography on silica gel (eluent: hexane/EtOAc= 200/1) to afford the desired product **7** as a colorless oil (14.9 mg, 71%).

R_f 0.57 (hexane/EtOAc= 10/1); **^{1}H NMR** (600 MHz, $CDCl_3$) δ 7.73 (d, *J* = 7.7 Hz, 2H), 7.36–7.32 (m, 1H), 7.22 (td, *J* = 7.5, 1.2 Hz, 1H), 7.25−7.20 (m, 2H), 7.03−7.00 (m, 1H), 6.95 (dd, *J* = 8.1, 0.9 Hz, 1H), 1.64 (s, 6H); **^{13}C{^{1}H} NMR** (150 MHz, $CDCl_3$) δ 152.8, 139.6, 129.5, 128.7, 128.0, 127.8, 123.3, 122.9, 122.5, 122.3, 121.6, 118.1, 77.6, 27.6; **HRMS** (EI) *m*/*z*: $[M]^+$ calcd for $C_{15}H_{14}O$ 210.1045, found 210.1048.

One-pot annulation–iodine deletion process

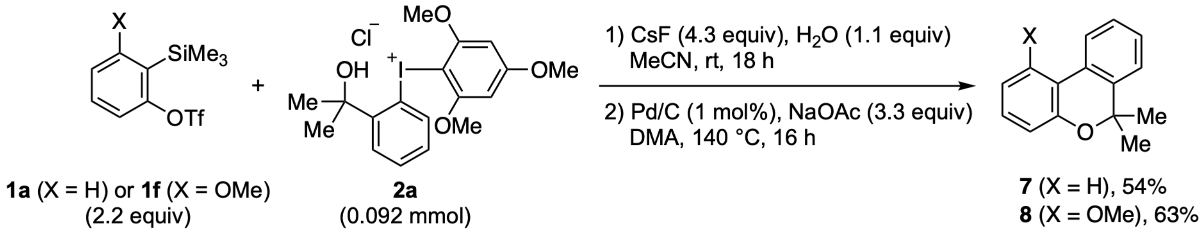

6,6-Dimethyl-6*H*-benzo[*c*]chromene (7): Under an argon atmosphere, an oven-dried Schlenk tube equipped with a magnetic stir bar was charged with **2a** (42.8 mg, 0.092 mmol, 1.0 equiv), CsF (60.8 mg, 0.4 mmol, 4.3 equiv), and MeCN (0.5 mL). To the solution were added H_2O (1.8 µL, 0.1 mmol, 1.1 equiv) and 2-(trimethylsilyl)phenyl trifluoromethanesulfonate (**1a**; 59.7 mg, 0.2 mmol, 2.2 equiv) at room temperature, and the resulting mixture was stirred for 18 h. To the mixture was added palladium on carbon (10 wt% Pd/C, 1.1 mg, 1.0 µmol of Pd, 1 mol%), NaOAc (24.6 mg, 0.3 mmol, 3.3 equiv), and *N*,*N*-dimethylacetamide (DMA, 2 mL). The

mixture was heated to 140 °C and stirred for 16 h. After quenching with H_2O (5 mL), the mixture was extracted with Et_2O (10 mL × 3). The combined organic layer was washed with brine, dried over $MgSO_4$, and concentrated under reduced pressure. The residue was purified by flash chromatography on silica gel (eluent: hexane/EtOAc= 200/1) to afford the desired product **7** (10.5 mg, 54%).

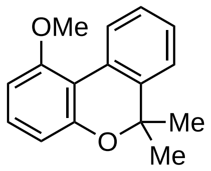

1-Methoxy-6,6-dimethyl-6*H*-benzo[*c*]chromene (8):[16] The one-pot synthesis was performed following the procedure described above, using the aryne precursor **1f**. Colorless oil (14.0 mg, 63%); R_f 0.51 (hexane/EtOAc= 10/1); **^{1}H NMR** (400 MHz, $CDCl_3$) δ 8.43 (dd, J = 7.6, 1.2 Hz, 1H), 7.34–7.22 (m, 3H), 7.15 (t, J = 8.3 Hz, 1H), 6.66–6.60 (m, 2H), 3.94 (s, 3H), 1.61 (s, 6H); **^{13}C{^{1}H} NMR** (101 MHz, $CDCl_3$) δ 157.5, 154.7, 140.0, 129.0, 127.7, 127.4, 127.3, 127.0, 122.5, 112.6, 111.3, 104.8, 77.5, 55.8, 27.1; **HRMS** (EI) *m*/*z*: $[M]^+$ calcd for $C_{16}H_{16}O_2$ 240.1150, found 240.1146.

5. Synthesis of Cannabinol

Synthesis of Aryne Fragment

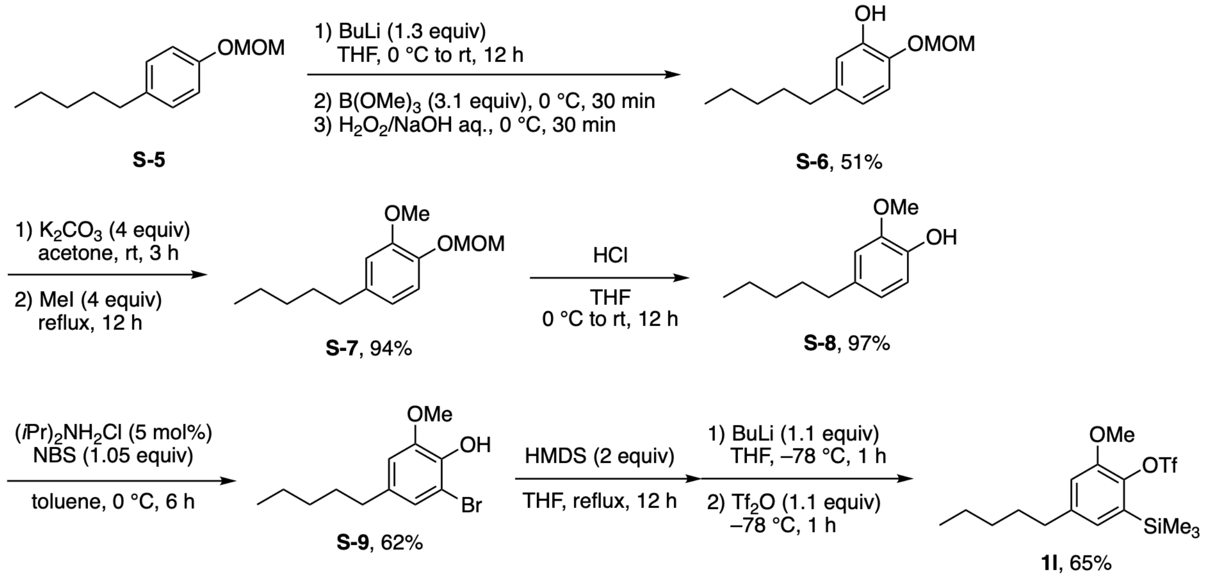

2-(Methoxymethoxy)-5-pentylphenol (S-6): Under an argon atmosphere, a 200 mL three-necked flask equipped with a magnetic stir bar was charged with 1-(methoxymethoxy)-4-pentylbenzene[17] (**S-5**; 2.08 g, 9.99 mmol, 1.0 equiv) and THF (30 mL). To the solution was added BuLi (1.53 M in hexane, 8.5 mL, 13 mmol, 1.3 equiv) dropwise over 3 h at 0 °C, and the resulting mixture was allowed to gradually warm to room temperature and stirred for 12 h. To the mixture was added $B(OMe)_3$ (3.46 mL, 31 mmol, 3.1 equiv) dropwise at 0 °C, and the mixture was stirred for 30 min at the same temperature. To the mixture was added H_2O_2/3 M NaOH aq. (1/1, 20 mL) dropwise at 0 °C, and the mixture was stirred for 30 min at the same temperature. After the mixture was treated with sat. $NaHSO_3$ aq. (20 mL) dropwise at 0 °C, the mixture was extracted with EtOAc (20 mL × 3), washed with brine (10 mL), dried over Na_2SO_4, and concentrated under reduced pressure. The residue was purified by column chromatography (eluent: hexane/EtOAc = 30/1) on silica gel to afford the desired product **S-6** as a brown oil (1.15 g, 51%).

R_f 0.17 (Hexane/EtOAc = 10/1); **^{1}H NMR** (400 MHz, $CDCl_3$) δ 6.97 (d, J = 8.2 Hz, 1H), 6.78 (d, J = 1.8 Hz, 1H), 6.62 (dd, J = 8.2, 1.8 Hz, 1H), 5.90 (s, 1H), 5.16 (s, 2H), 3.52 (s, 3H), 2.51 (t, J = 7.7 Hz, 2H), 1.63−1.52 (m, 2H), 1.38−1.24 (m, 4H), 0.88 (t, J = 6.8 Hz, 3H); **^{13}C{^{1}H} NMR** (150 MHz, $CDCl_3$) δ 146.3, 142.7, 138.5, 120.1, 115.8, 115.5, 96.5, 56.5, 35.5, 31.6, 31.3, 22.7, 14.2; **HRMS** (EI) *m/z*: $[M]^+$ calcd for $C_{13}H_{20}O_3$ 224.1412, found 224.1414.

2-Methoxy-1-(methoxymethoxy)-4-pentylbenzene (S-7): Under an argon atmosphere, a 200

mL three-necked flask equipped with a magnetic stir bar was charged with 2-(methoxymethoxy)-5-pentylphenol **S-6** (1.12 g, 4.99 mmol, 1.0 equiv), K_2CO_3 (2.76 g, 20 mmol, 4 equiv) and acetone (50 mL). The reaction mixture was stirred for 3 h at room temperature. To the solution was added MeI (1.25 mL, 20 mmol, 4 equiv), and the resulting mixture was stirred at reflux for 12 h. After cooling to room temperature, the reaction mixture was filtered, and the filtrate was concentrated under reduced pressure. The residue was purified by column chromatography (eluent: hexane/EtOAc = 10/1) on silica gel to afford the desired product **S-7** as a colorless oil (1.12 g, 94%).

R_f 0.20 (hexane/EtOAc = 10/1); **^{1}H NMR** (600 MHz, $CDCl_3$) δ 7.05 (d, J = 8.1 Hz, 1H), 6.72 (d, J = 1.9 Hz, 1H), 6.70 (dd, J = 8.1, 2.0 Hz, 1H), 5.19 (s, 2H), 3.87 (s, 3H), 3.52 (s, 3H), 2.55 (t, J = 7.8 Hz, 2H), 1.63−1.56 (m, 2H), 1.38−1.28 (m, 4H), 0.90 (t, J = 7.0 Hz, 3H); **^{13}C{^{1}H} NMR** (150 MHz, $CDCl_3$) δ 149.7, 144.5, 137.7, 120.5, 116.7, 112.3, 95.9, 56.3, 56.0, 35.8, 31.7, 31.5, 22.7, 14.2; **HRMS** (EI) *m*/*z*: $[M]^+$ calcd for $C_{14}H_{22}O_3$ 238.1569, found 238.1571.

2-Methoxy-4-pentylphenol (S-8): A 200 mL flask equipped with a magnetic stir bar was charged with 2-methoxy-1-(methoxymethoxy)-4-pentylbenzene **S-7** (1.07 g, 4.49 mmol, 1.0 equiv) and THF (18 mL). To the solution was added 3 M HCl aq. (18 mL) at 0 °C, and the resulting mixture was allowed to warm to room temperature and stirred for 12 h. After quenching with sat. $NaHCO_3$ aq., the mixture was extracted with EtOAc (10 mL × 3), washed with brine (10 mL), dried over Na_2SO_4, and concentrated under reduced pressure. The residue was purified by column chromatography (eluent: hexane/EtOAc = 10/1) on silica gel to afford the desired product **S-8** as a colorless oil (846 mg, 97%).

R_f 0.20 (hexane/EtOAc = 10/1); **^{1}H NMR** (600 MHz, $CDCl_3$) δ 6.82 (dd, J = 7.2, 1.2 Hz, 1H), 6.70–6.64 (m, 2H), 5.44 (s, 1H), 3.88 (s, 3H), 2.53 (t, J = 7.8 Hz, 2H), 1.63–1.52 (m, 2H), 1.38–1.28 (m, 2H), 0.89 (t, J = 6.9 Hz, 3H); **^{13}C{^{1}H} NMR** (150 MHz, $CDCl_3$) δ 146.4, 143.6, 135.1, 121.0, 114.2, 111.1, 56.0, 35.8, 31.7, 22.7, 14.2, one carbon could not be found due to overlapping; **HRMS** (EI) *m*/*z*: $[M]^+$ calcd for $C_{12}H_{18}O_2$ 194.1307, found 194.1305.

2-Bromo-6-methoxy-4-pentylphenol (S-9): A 200 mL flask equipped with a magnetic stir bar was charged with 2-methoxy-4-pentylphenol **S-8** (486 mg, 2.50 mmol, 1.0 equiv), *i*Pr_2NH_2Cl (17.2 mg, 0.125 mmol, 5 mol%) and toluene (50 mL). To the solution was added NBS (467 mg, 2.63 mmol, 1.05 equiv) slowly at 0 °C, and the resulting mixture was stirred for 6 h at the same temperature. After quenching with sat. $NaHCO_3$ aq., the mixture was extracted with

EtOAc (10 mL × 3), and the combined organic layers were washed with brine (10 mL), dried over Na_2SO_4, and concentrated under reduced pressure. The residue was purified by column chromatography (eluent: hexane/CH_2Cl_2 = 2/1) on silica gel to afford the desired product **S-9** as a colorless oil (420 mg, 62%).

R_f 0.34 (hexane/CH_2Cl_2 = 2/1); **^{1}H NMR** (400 MHz, $CDCl_3$) δ 6.91 (d, J = 1.6 Hz, 1H), 6.62 (d, J = 1.6 Hz, 1H), 5.73 (s, 1H), 3.89 (s, 3H), 2.50 (t, J = 7.7 Hz, 2H), 1.62−1.52 (m, 2H), 1.37−1.27 (m, 4H), 0.90 (t, J = 6.9 Hz, 3H); **^{13}C{^{1}H} NMR** (101 MHz, $CDCl_3$) δ 147.0, 141.0, 135.8, 124.2, 110.4, 108.0, 56.3, 35.5, 31.5, 31.3, 22.6, 14.1; **HRMS** (EI) *m/z*: $[M]^+$ calcd for $C_{12}H_{17}BrO_2$ 272.0412, found 272.0416.

2-Methoxy-4-pentyl-6-(trimethylsilyl)phenyl trifluoromethanesulfonate (1l): A 30 mL flask equipped with a magnetic stir bar was charged with 2-bromo-6-methoxy-4-pentylphenol **S-9** (410 mg, 1.50 mmol, 1.0 equiv) and THF (3 mL). To the solution was added HMDS (0.63 mL, 3.0 mmol, 2.0 equiv), and the resulting mixture was refluxed for 12 h. After cooling to room temperature, the solvent was removed under vacuum to afford (2-bromo-6-methoxy-4-pentylphenoxy)trimethylsilane **S-10**, which was used in the next step without further purification. A 30 mL flask equipped with a magnetic stir bar was charged with **S-10** and THF (9 mL). To the solution was added slowly BuLi (1.53 M in hexane, 1.08 mL, 1.65 mmol, 1.1 equiv) at –78 °C, and the mixture was stirred for 1 h at the same temperature. To the solution was added Tf_2O (0.27 mL, 1.65 mmol, 1.1 equiv) dropwise at the same temperature, and the resulting mixture was allowed to warm to room temperature. After quenching with sat. $NaHCO_3$ aq., the mixture was extracted with EtOAc (10 mL × 3), washed with brine (10 mL), dried over Na_2SO_4, and concentrated under reduced pressure. The residue was purified by column chromatography (eluent: hexane/EtOAc = 100/1) on silica gel to afford the desired product **1l** as a colorless oil (391 mg, 65%).

R_f 0.43 (Hexane/EtOAc = 10/1); **^{1}H NMR** (600 MHz, $CDCl_3$) δ 6.83 (d, J = 2.0 Hz, 1H), 6.82 (d, J = 2.0 Hz, 1H), 3.85 (s, 3H), 2.59 (t, J = 7.9 Hz, 2H), 1.64−1.57 (m, 2H), 1.39−1.32 (m, 4H), 0.91 (t, J = 7.2 Hz, 3H), 0.37 (s, 9H); **^{13}C{^{1}H} NMR** (150 MHz, $CDCl_3$) δ 150.0, 143.9, 141.5, 134.6, 126.6, 119.1 (q, $J_{C–F}$ = 319.4 Hz), 114.2, 55.6, 36.0, 31.7, 31.3, 22.6, 14.1, –0.3; **HRMS** (EI) *m/z*: $[M]^+$ calcd for $C_{16}H_{25}F_3O_4SSi$ 398.1195, found 398.1197.

Assembly of Aryne and Iodonium Fragments

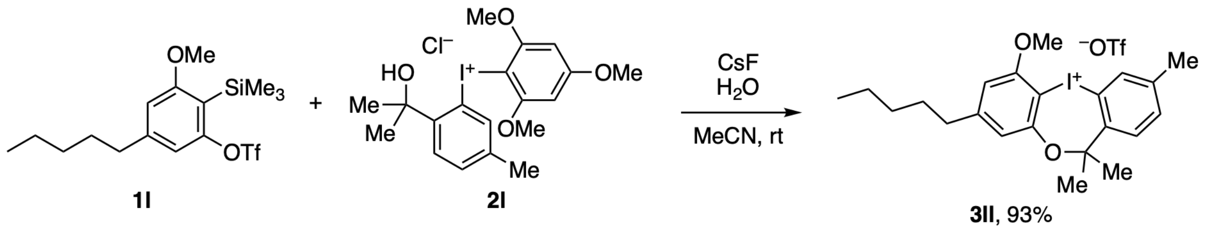

6-Methoxy-3,11,11-trimethyl-8-pentyl-11*H*-dibenzo[*b*,*f*][1,4]iodaoxepin-5-ium trifluoromethanesulfonate (3li): The reaction between aryne precursor **1l** (79.7 mg, 0.20 mmol) and iodonium salt **2l** (see Section 2; 44.2 mg, 0.092 mmol) was carried out according to **GP3** (see Section 3). Colorless solid (51.5 mg, 93%); R_f 0.13 (CH_2Cl_2/MeOH = 10/1); m.p. 115–116 °C (decomp.); ^{1}**H NMR** (400 MHz, CD_3OD) δ 7.90 (s, 1H), 7.46 (d, J = 8.2 Hz, 1H), 7.41 (d, J = 8.2 Hz, 1H), 6.91 (d, J = 1.1 Hz, 1H), 6.86 (d, J = 1.1 Hz, 1H), 3.95 (s, 3H), 2.67 (t, J = 7.8 Hz, 2H), 2.34 (s, 3H), 1.90 (s, 6H), 1.68–1.56 (m, 2H), 1.38–1.28 (m, 4H), 0.89 (t, J = 7.0 Hz, 3H); ^{13}**C{^{1}H} NMR** (150 MHz, CD_3OD) δ 159.1, 155.2, 153.1, 142.4, 141.7, 138.8, 134.7, 131.6, 121.8 (q, $J_{C–F}$ = 318.4 Hz), 118.4, 111.7, 110.4, 105.6, 85.2, 57.5, 37.2, 32.5, 32.1, 30.7, 23.5, 20.3, 14.3; ^{19}**F NMR** (376 MHz, CD_3OD) δ –80.0; **HRMS** (FAB) *m/z*: $[M–OTf]^+$ calcd for $C_{22}H_{28}IO_2$ 451.1129, found 451.1127.

Conversion to Cannabinol

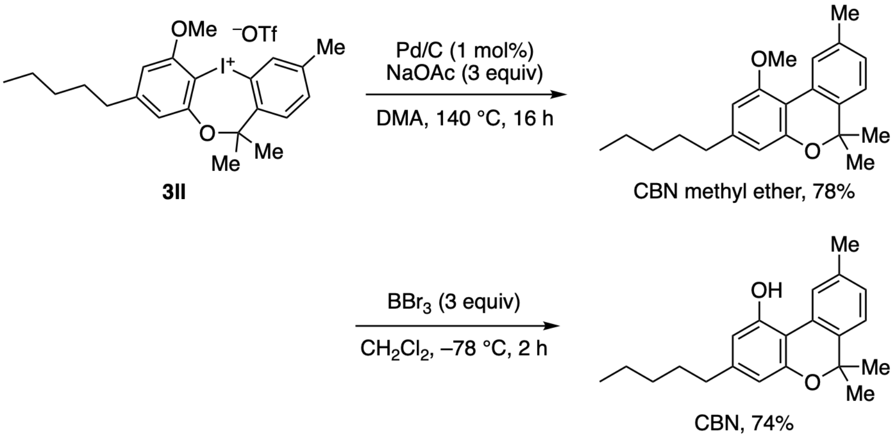

1-Methoxy-6,6,9-trimethyl-3-pentyl-6*H*-benzo[*c*]chromene (Cannabinol methyl ether):[18] Synthesized according to the iodine-deletion procedure described above from **3ll** (30.0 mg, 0.050 mmol). Colorless oil (12.6 mg, 78%); R_f 0.23 (hexane/CH_2Cl_2 = 3/1); ^{1}**H NMR** (400 MHz, $CDCl_3$) δ 8.21 (s, 1H), 7.11 (d, J = 7.8 Hz, 1H), 7.05 (d, J = 7.9 Hz, 1H), 6.47 (d, J = 1.5 Hz, 1H), 6.43 (d, J = 1.4 Hz, 1H), 3.93 (s, 3H), 2.56 (t, J = 7.8 Hz, 2H), 2.38 (s, 3H), 1.68–1.57 (m, 8H), 1.37–1.30 (m, 4H), 0.90 (t, J = 6.9 Hz, 3H); ^{13}**C{^{1}H} NMR** (150 MHz, $CDCl_3$) δ

157.5, 154.5, 144.6, 137.0, 136.6, 127.8, 127.6, 127.2, 122.4, 111.1, 110.0, 105.2, 77.2, 55.7, 36.3, 31.7, 30.8, 27.2, 22.7, 21.8, 14.2; **HRMS** (EI) *m*/*z*: $[M]^+$ calcd for $C_{22}H_{28}O_2$ 324.2089, found 324.2098.

6,6,9-Trimethyl-3-pentyl-6*H*-benzo[*c*]chromen-1-ol (Cannabinol):[18] Under an argon atmosphere, an oven-dried Schlenk tube equipped with a magnetic stir bar was charged with cannabinol methyl ether (16.2 mg, 0.05 mmol, 1.0 equiv) and CH_2Cl_2 (2 mL). To the solution was added BBr_3 (14 μL, 0.15 mmol, 3.0 equiv) dropwise at –78 °C. The mixture was stirred at the same temperature for 2 h. After quenching with H_2O (2 mL), the mixture was extracted with CH_2Cl_2 (5 mL × 3). The combined organic layer was washed with brine, dried over Na_2SO_4, and concentrated under reduced pressure. The residue was purified by flash chromatography on silica gel (eluent: hexane/EtOAc= 15/1) to afford Cannabinol as a colorless oil (11.5 mg, 74%).

R_f 0.14 (hexane/EtOAc = 10/1); **^{1}H NMR** (400 MHz, $CDCl_3$) δ 8.15 (s, 1H), 7.14 (d, *J* = 7.9 Hz, 1H), 7.07 (d, *J* = 7.9 Hz, 1H), 6.44 (s, 1H), 6.29 (s, 1H), 5.11 (s, 1H), 2.50 (t, *J* = 7.8 Hz, 2H), 2.38 (s, 3H), 1.65–1.57 (m, 8H), 1.38–1.29 (m, 4H), 0.89 (t, *J* = 7.0 Hz, 3H); **^{13}C{^{1}H} NMR** (150 MHz, $CDCl_3$) δ 154.8, 153.1, 144.7, 137.06, 137.05, 127.8, 127.7, 126.5, 122.8, 111.0, 110.0, 108.8, 35.8, 31.6, 30.6, 27.3, 22.7, 21.7, 14.2, one carbon resonance (for tertiary ether moiety) could not be resolved due to overlap with the solvent peaks (δ ~77.0); **HRMS** (EI) *m*/*z*: $[M]^+$ calcd for $C_{21}H_{26}O_2$ 310.1933, found 310.1935.

6. Deuterium-Labeling Experiments

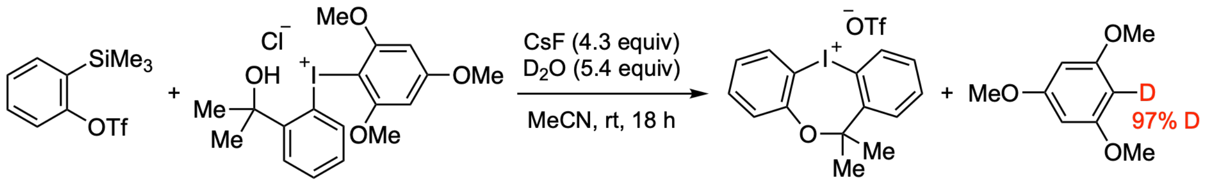

Deuterium-Labeling Experiment: Under an argon atmosphere, a 4 mL vial equipped with a magnetic stir bar was charged with **2a** (42.8 mg, 0.092 mmol, 1.0 equiv) and CsF (60.8 mg, 0.40 mmol, 4.3 equiv), and MeCN (0.5 mL). To the mixture were added D_2O (9.0 µL, 0.50 mmol, 5.4 equiv) and 2-(trimethylsilyl)phenyl trifluoromethanesulfonate (**1a**; 59.7 mg, 0.20 mmol, 2.2 equiv) at room temperature. The resulting mixture was stirred at the same temperature for 18 h and then filtered through a pad of silica gel (eluent: CH_2Cl_2/MeOH = 4/1). The filtrate was concentrated under reduced pressure, and the residue was purified by column chromatography on silica gel (eluent: CH_2Cl_2/MeOH = 100/1 to 4/1) to afford **3aa** (34.1 mg, 76%) and 2,4,6-trimethoxybenzene (TMP-D; 15.6 mg, quant). The deuterium content in the aromatic protons of TMP-D was determined to be 97% by ^{1}H NMR analysis (Figure S2).

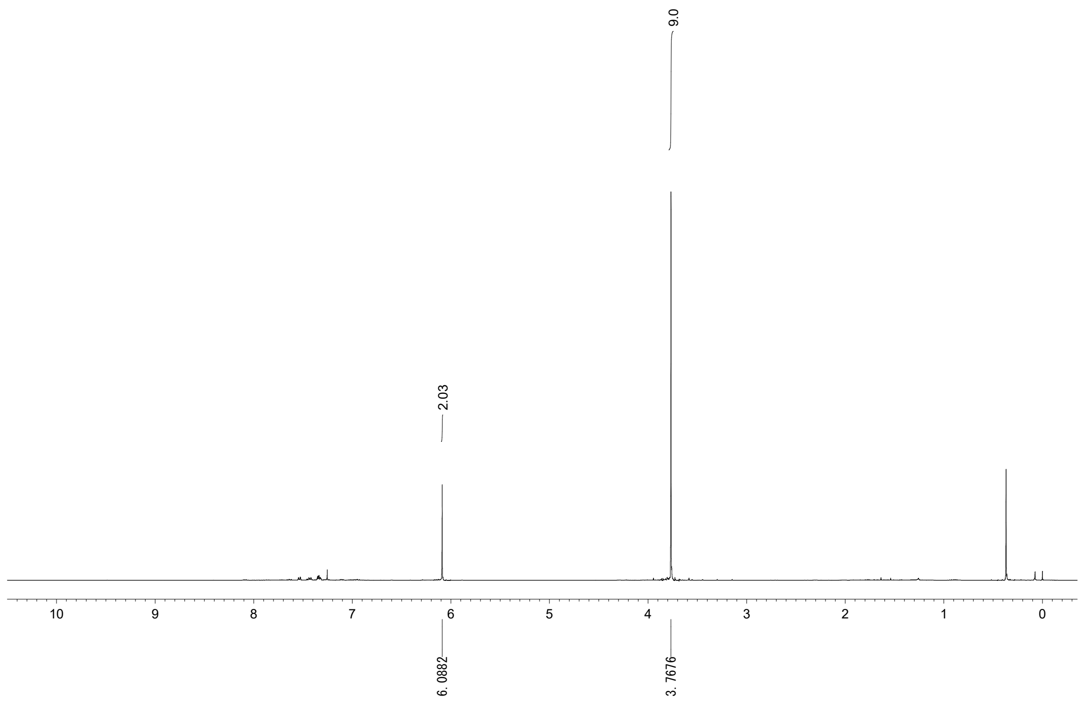

Figure S2. **^{1}H NMR** spectrum (400 MHz, $CDCl_3$) of the deuterium-labeling experiment.

7. X-Ray Crystallographic Analysis

X-ray crystal structure analysis of 2a

Single crystals of **2a** were obtained by recrystallization from $CHCl_3$/EtOAc. A suitable crystal was selected and loop on a XtaLAB Synergy R, DW system, HyPix diffractometer. The crystal was kept at 139(1) K during data collection. Using Olex2,[19] the structure was solved with the SHELXT[20] structure solution program using Intrinsic Phasing and refined with the SHELXL[21] refinement package using Least Squares minimisation. Crystallographic data of **2a** has been deposited on Cambridge Crystallographic Data Center, deposition no. CCDC 2541021.

Table S3. Crystal data and structure refinements for (2-(2-hydroxypropan-2-yl)phenyl)(2,4,6-trimethoxyphenyl)iodonium chloride (**2a**):

Empirical formula	$C_{18}H_{22}ClIO_4$
Formula weight	464.70
Temperature/K	139(1)
Crystal system	orthorhombic
Space group	Pccn
a/Å	12.0406(2)
b/Å	17.7037(4)
c/Å	17.8618(4)
α/°	90
β/°	90
γ/°	90
Volume/Å^3	3807.49(14)
Z	8
ρ_{calc}g/cm^3	1.621
μ/mm^{-1}	1.840
F(000)	1856.0
Crystal size/mm^3	0.2 × 0.2 × 0.2
Radiation	Mo Kα (λ = 0.71073)
2Θ range for data collection/°	4.684 to 61.968
Index ranges	$-17 \leq h \leq 16$, $-25 \leq k \leq 24$, $-24 \leq l \leq 25$
Reflections collected	50358
Independent reflections	5388 [R_{int} = 0.0662, R_{sigma} = 0.0322]
Data/restraints/parameters	5388/0/223
Goodness-of-fit on F^2	1.025
Final R indexes [I>=2σ (I)]	R_1 = 0.0259, wR_2 = 0.0591
Final R indexes [all data]	R_1 = 0.0375, wR_2 = 0.0641
Largest diff. peak/hole / e Å^{-3}	0.72/-1.02

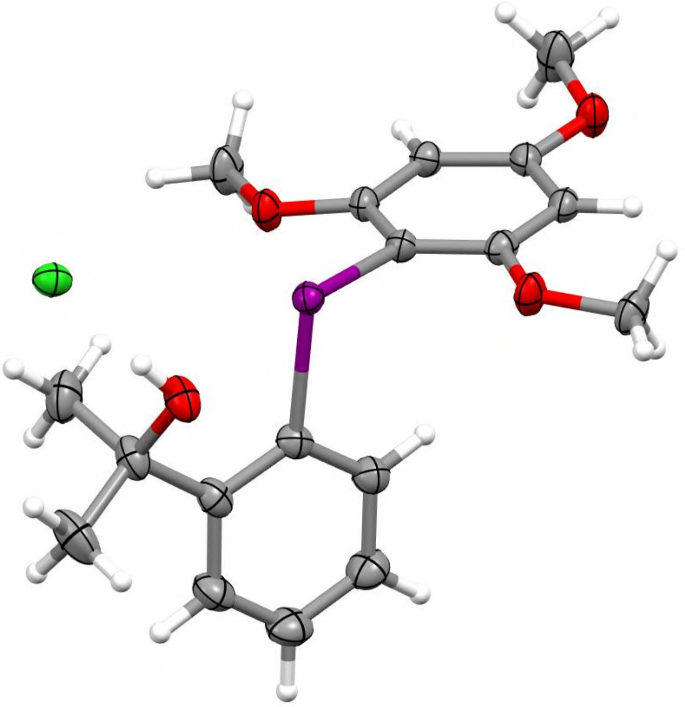

Figure S3. Thermal ellipsoid (50 % probability) plot of **2a**, CCDC No. 2541021. Color code of atoms: hydrogen, white; carbon, gray; chlorine, green; iodine, purple; oxygen, red

X-ray crystal structure analysis of 2e

Single crystals of **2e** were obtained by recrystallization from CH_2Cl_2/Hexane. A suitable crystal was selected and loop on a XtaLAB Synergy R, DW system, HyPix diffractometer. The crystal was kept at 104.0(8) K during data collection. Using Olex2,[19] the structure was solved with the SHELXT[20] structure solution program using Intrinsic Phasing and refined with the SHELXL[21] refinement package using Least Squares minimisation.

Crystallographic data of **2e** has been deposited on Cambridge Crystallographic Data Center, deposition no. CCDC 2541032.

Table S4. Crystal data and structure refinements for 3-methyl-3-(trifluoromethyl)-1-(2,4,6-trimethoxyphenyl)-1,3-dihydro-1λ^3-benzo[*d*][1,2]iodaoxole (**2e**):

Empirical formula	$2(C_{18}H_{18}F_3IO_4)$
Formula weight	482.22
Temperature/K	104.0(8)
Crystal system	triclinic
Space group	P-1
a/Å	7.79727(8)
b/Å	13.69121(13)
c/Å	17.66933(14)
α/°	109.3465(8)
β/°	90.2850(7)
γ/°	91.8636(8)
Volume/Å^3	1778.57(3)
Z	2
ρ_{calc}g/cm^3	1.801
μ/mm^{-1}	14.627
F(000)	952.0
Crystal size/mm^3	0.15 × 0.15 × 0.1
Radiation	Cu Kα (λ = 1.54184)
2Θ range for data collection/°	5.302 to 150.896
Index ranges	$-8 \leq h \leq 9$, $-17 \leq k \leq 17$, $-22 \leq l \leq 21$
Reflections collected	66094
Independent reflections	7129 [R_{int} = 0.1102, R_{sigma} = 0.0388]
Data/restraints/parameters	7129/0/477
Goodness-of-fit on F^2	1.045
Final R indexes [I>=2σ (I)]	R_1 = 0.0453, wR_2 = 0.1253
Final R indexes [all data]	R_1 = 0.0468, wR_2 = 0.1272

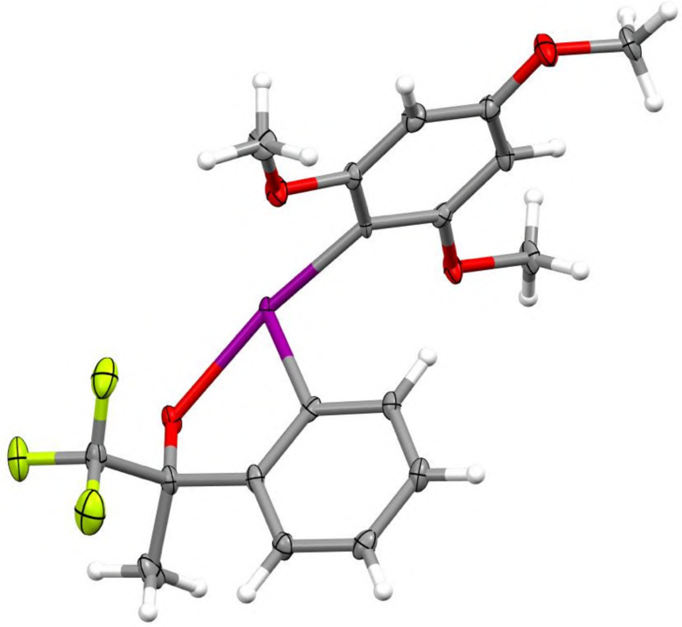

Figure S4. Thermal ellipsoid (50 % probability) plot of **2e**, CCDC No. 2541032. Color code of atoms: hydrogen, white; carbon, gray; fluorine, green; iodine, purple; oxygen, red

X-ray crystal structure analysis of 3aa

Single crystals of **3aa** were obtained by recrystallization from CH_2Cl_2/pentane. A suitable crystal was selected and loop on a XtaLAB Synergy R, DW system, HyPix diffractometer. The crystal was kept at 100(1) K during data collection. Using Olex2,[19] the structure was solved with the SHELXT[20] structure solution program using Intrinsic Phasing and refined with the SHELXL[21] refinement package using Least Squares minimisation.

Crystallographic data of **3aa** has been deposited on Cambridge Crystallographic Data Center, deposition no. CCDC 2541035.

Table S5. Crystal data and structure refinements for 11,11-dimethyl-11*H*-dibenzo[*b,f*][1,4]iodaoxepin-5-ium trifluoromethanesulfonate (**3aa**):

Empirical formula	$C_{16}H_{14}F_3IO_4S$
Formula weight	486.23
Temperature/K	100(1)
Crystal system	orthorhombic
Space group	$P2_12_12_1$
a/Å	7.71880(10)
b/Å	13.09150(10)
c/Å	16.9575(2)
α/°	90
β/°	90
γ/°	90
Volume/Å^3	1713.57(3)
Z	4
ρ_{calc}g/cm^3	1.885
μ/mm^{-1}	2.040
F(000)	952.0
Crystal size/mm^3	0.2 × 0.2 × 0.1
Radiation	Mo Kα (λ = 0.71073)
2Θ range for data collection/°	4.804 to 61.72
Index ranges	$-11 \leq h \leq 10$, $-17 \leq k \leq 18$, $-23 \leq l \leq 24$
Reflections collected	59158
Independent reflections	4862 [R_{int} = 0.0415, R_{sigma} = 0.0166]
Data/restraints/parameters	4862/0/228
Goodness-of-fit on F^2	1.029
Final R indexes [I>=2σ (I)]	R_1 = 0.0139, wR_2 = 0.0316
Final R indexes [all data]	R_1 = 0.0143, wR_2 = 0.0317
Largest diff. peak/hole / e Å^{-3}	0.30/-0.34
Flack parameter	-0.026(4)

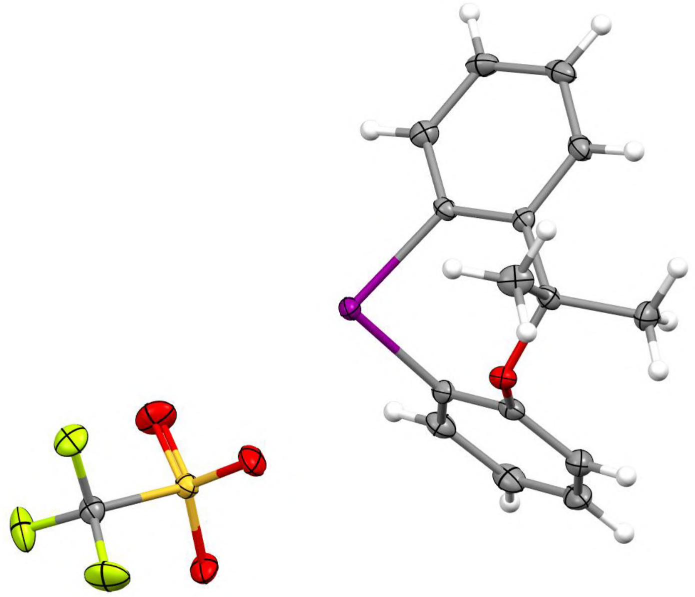

Figure S5. Thermal ellipsoid (50 % probability) plot of **3aa**, CCDC No. 2541035. Color code of atoms: hydrogen, white; carbon, gray; fluorine, green; iodine, purple; oxygen, red; sulfur, yellow

8. References

(1) Jiang, H.; Zhang, Y.; Xiong, W.; Cen, J.; Wang, L.; Cheng, R.; Qi, C.; Wu, W. A Three-Phase Four-Component Coupling Reaction: Selective Synthesis of *o*-Chloro Benzoates by KCl, Arynes, CO_2, and Chloroalkanes. *Org. Lett.* **2019**, *21*, 345–349.

(2) Cui, D.; Feng, Y.; Gan, Y.; Yin, J.; Wang, W.; Fan, Y.; Gao, L.; Ke, B.; Song, Z. (3 + 2)-Annulation of 1,3-N,Si-Tetraorganosilane Reagents $TsHNCH_2SiBnR^1R^2$ with Arynes for Efficient Synthesis of 3-Silaindolines. *Org. Chem. Front.* **2022**, *9*, 5453–5462.

(3) Tadross, P. M.; Gilmore, C. D.; Bugga, P.; Virgil, S. C.; Stoltz, B. M. Regioselective Reactions of Highly Substituted Arynes. *Org. Lett.* **2010**, *12*, 1224–1227.

(4) Medina, J. M.; MacKey, J. L.; Garg, N. K.; Houk, K. N. The Role of Aryne Distortions, Steric Effects, and Charges in Regioselectivities of Aryne Reactions. *J. Am. Chem. Soc.* **2014**, *136*, 15798–15805.

(5) Fang, Z.; Shou, J. Y.; Khan, M. N.; He, X. B.; Jia, X.; Qing, F. L. Insertion of Arynes into the C–C σ-Bond of α-Pentafluorosulfanyl Ketones: Access to 2-(Pentafluorosulfanyl)Methyl Benzophenones. *J. Org. Chem.* **2025**, *90*, 7841–7848.

(6) Matoušek, V.; Pietrasiak, E.; Schwenk, R.; Togni, A. One-Pot Synthesis of Hypervalent Iodine Reagents for Electrophilic Trifluoromethylation. *J. Org. Chem.* **2013**, *78*, 6763–6768.

(7) Geary, G. C.; Hope, E. G.; Singh, K.; Stuart, A. M. Electrophilic Fluorination Using a Hypervalent Iodine Reagent Derived from Fluoride. *Chem. Commun.* **2013**, *49*, 9263–9265.

(8) Powers, D. C.; Lee, E.; Ariafard, A.; Sanford, M. S.; Yates, B. F.; Canty, A. J.; Ritter, T. Connecting Binuclear Pd(III) and Mononuclear Pd(IV) Chemistry by Pd–Pd Bond Cleavage. *J. Am. Chem. Soc.* **2012**, *134*, 12002–12009.

(9) Ge, Y.; Shao, Y.; Wu, S.; Liu, P.; Li, J.; Qin, H.; Zhang, Y.; Xue, X. S.; Chen, Y. Distal Amidoketone Synthesis Enabled by Dimethyl Benziodoxoles via Dual Copper/Photoredox Catalysis. *ACS Catal.* **2023**, *13*, 3749–3756.

(10) Dolenc, D.; Plesničar, B. Abstraction of Iodine from Aromatic Iodides by Alkyl Radicals: Steric and Electronic Effects. *J. Org. Chem.* **2006**, *71*, 8028–8036.

(11) Niedermann, K.; Welch, J. M.; Koller, R.; Cvengroš, J.; Santschi, N.; Battaglia, P.; Togni, A. New Hypervalent Iodine Reagents for Electrophilic Trifluoromethylation and Their Precursors: Synthesis, Structure, and Reactivity. *Tetrahedron* **2010**, *66*, 5753–5761.

(12) Souilah, C.; Jannuzzi, S. A. V.; Demirbas, D.; Ivlev, S.; Swart, M.; DeBeer, S.; Casitas, A. Synthesis of Fe^{III} and Fe^{IV} Cyanide Complexes Using Hypervalent Iodine Reagents as Cyano-Transfer One-Electron Oxidants. *Angew. Chem. Int. Ed.* **2022**, *61*, e202201699.

(13) Ren, J.; Jia, M. C.; Du, F. H.; Zhang, C. A General Method for One-Step Synthesis of Monofluoroiodane(III) Reagents Using Silver Difluoride. *Chin. Chem. Lett.* **2022**, *33*, 4834–4837.

(14) Shibasaki, K.; Togo, H. Novel Transformation of Aryl 2-Iodophenyl Ketones into 1,3-Diarylisoquinolines with $(TMS)_2NH$, Styrenes, NIS, and ^{t}BuOK. *Tetrahedron* **2021**, *79*,

131864.
(15) Killander, D.; Sterner, O. Reagent-Controlled Cyclization–Deprotection Reaction to Yield either Fluorenes or Benzochromenes. *Eur. J. Org. Chem.* **2014**, *2014*, 6507–6512.
(16) Liang, Z.; Liu, C.; Fan, J.; Wang, M.; Yan, X.; Huang, M.; Cai, S. Photocatalytic Dehydrogenated Etherification of 2-Aryl Benzylic Alcohols. *Green Chem.* **2022**, *24*, 7442–7447.
(17) Abe, H.; Aoyagi, Y.; Inouye, M. A Rigid C_{3v}-Symmetrical Host for Saccharide Recognition: 1,3,5-Tris(2-Hydroxyaryl)-2,4,6-Trimethylbenzenes. *Org. Lett.* **2005**, *7*, 59–61.
(18) Teske, J. A.; Deiters, A. A Cyclotrimerization Route to Cannabinoids. *Org. Lett.* **2008**, *10*, 2195–2198.
(19) Dolomanov, O. V.; Bourhis, L. J.; Gildea, R. J.; Howard, J. A. K.; Puschmann, H. OLEX2: A Complete Structure Solution, Refinement and Analysis Program. *J. Appl. Crystallogr.* 2009, *42*, 339–341.
(20) Sheldrick, G. M. SHELXT-Integrated Space-Group and Crystal-Structure Determination. *Acta Cryst.* **2015**, *A71*, 3–8.
(21) Sheldrick, G. M. Crystal Structure Refinement with SHELXL. *Acta Cryst.* **2015**, *C71*, 3–8.

1-Chloro-3,3,5-trimethyl-1,3-dihydro-1λ^3-benzo[*d*][1,2]iodaoxole (S-3-2f)

^{1}H NMR (400 MHz, $CDCl_3$)

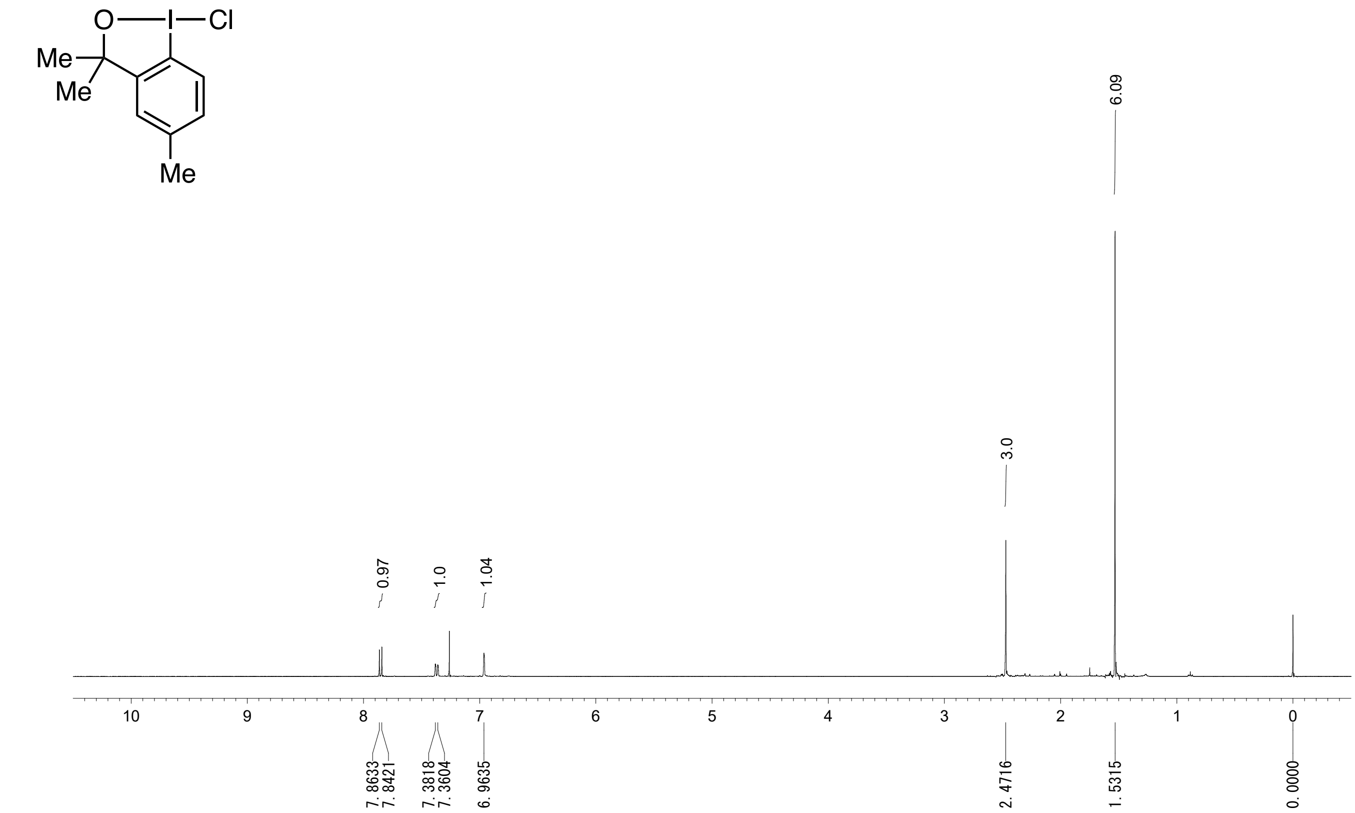

^{13}C{^{1}H} NMR (150 MHz, $CDCl_3$)

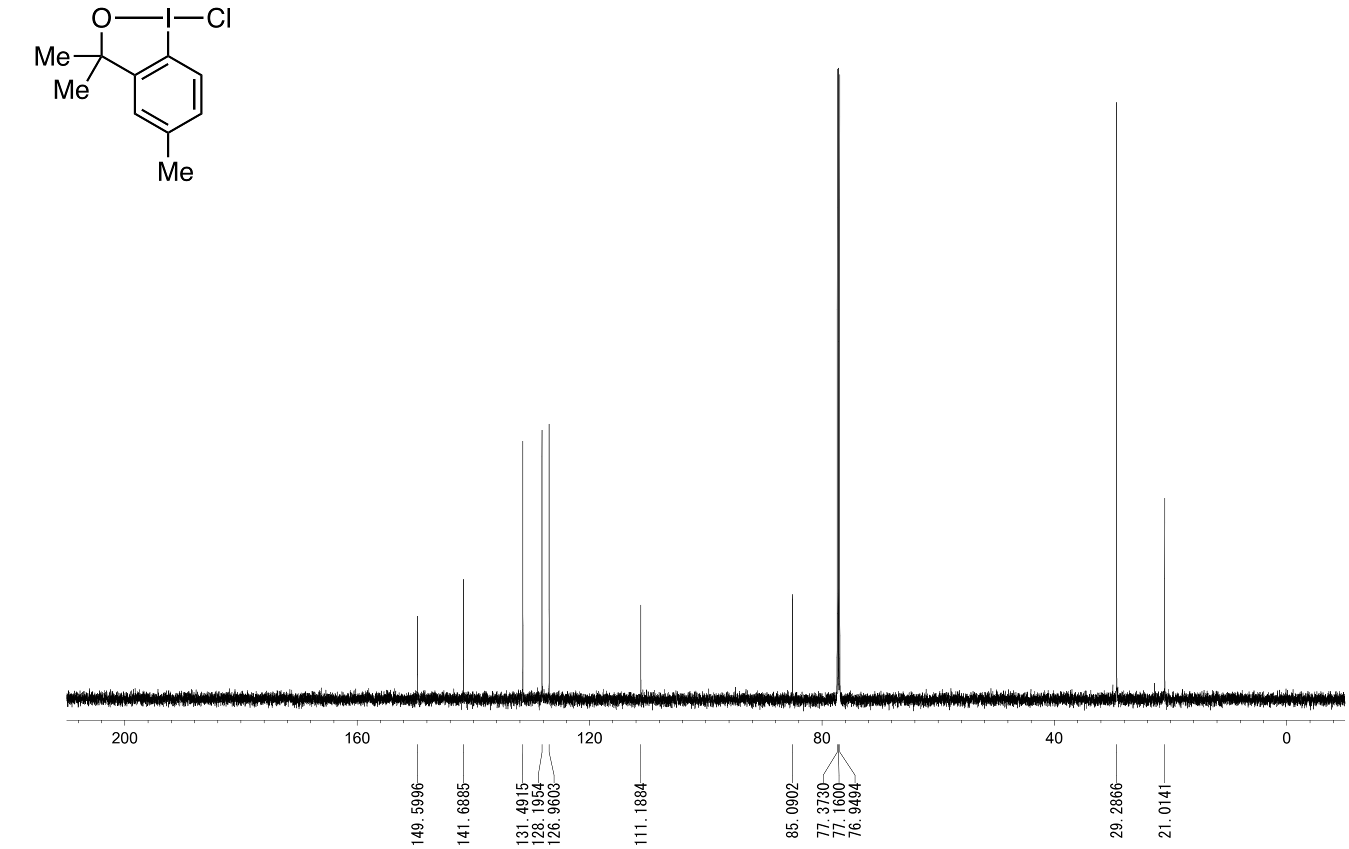

5-Bromo-1-chloro-3,3-dimethyl-1,3-dihydro-1λ^3-benzo[*d*][1,2]iodaoxole (S-3-2h)

^{1}H NMR (400 MHz, $CDCl_3$)

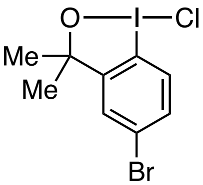

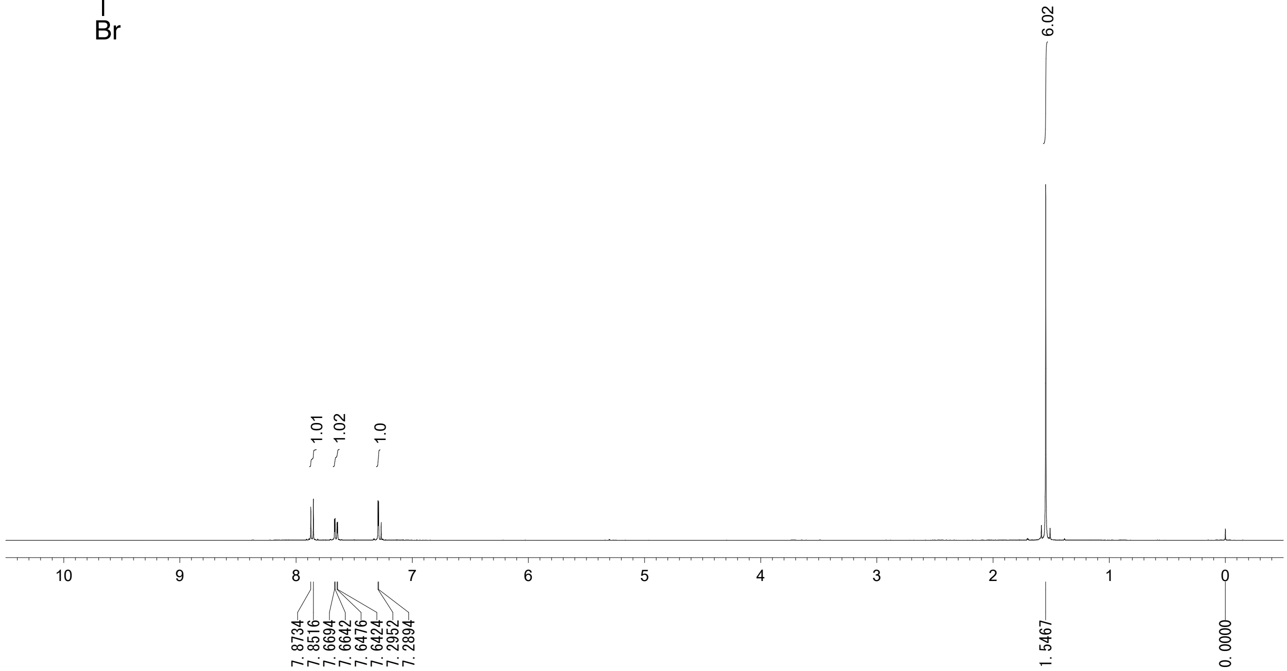

$^{13}C\{^1H\}$ NMR (150 MHz, $CDCl_3$)

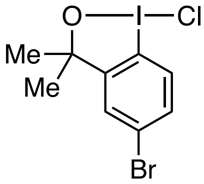

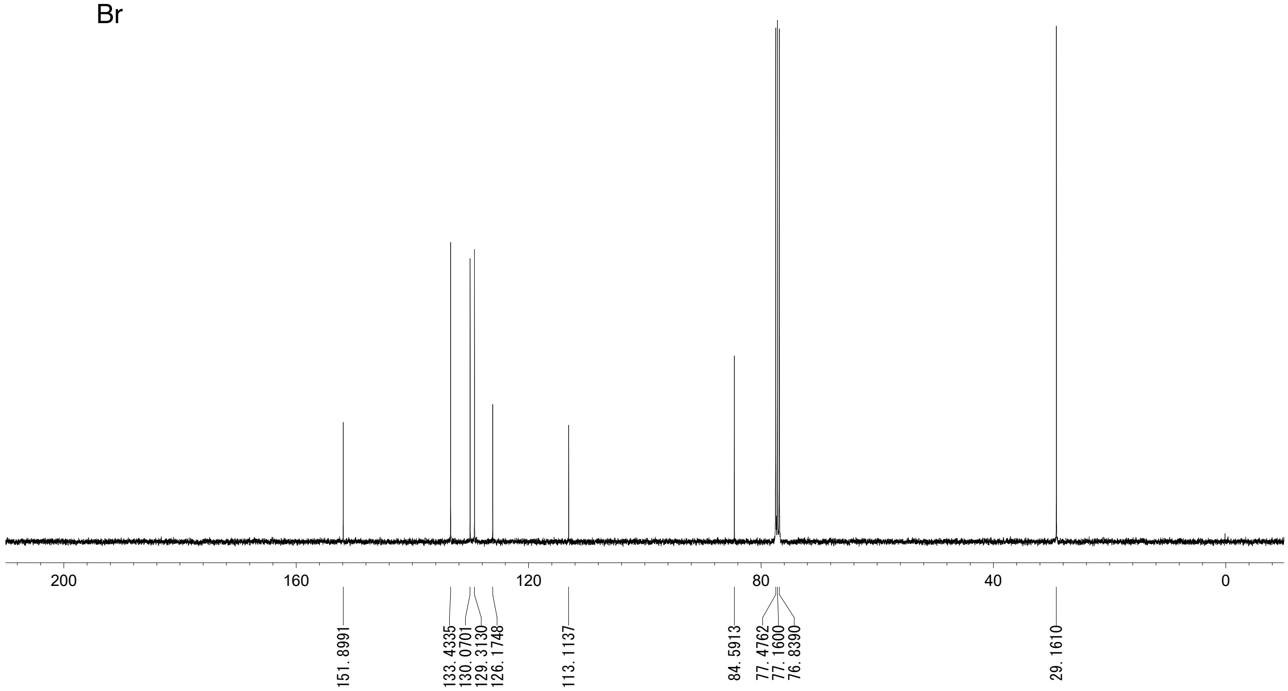

1-Chloro-3,3,6-trimethyl-1,3-dihydro-1λ^3-benzo[*d*][1,2]iodaoxole (S-3-2l)

^{1}H NMR (400 MHz, $CDCl_3$)

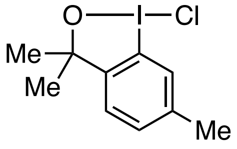

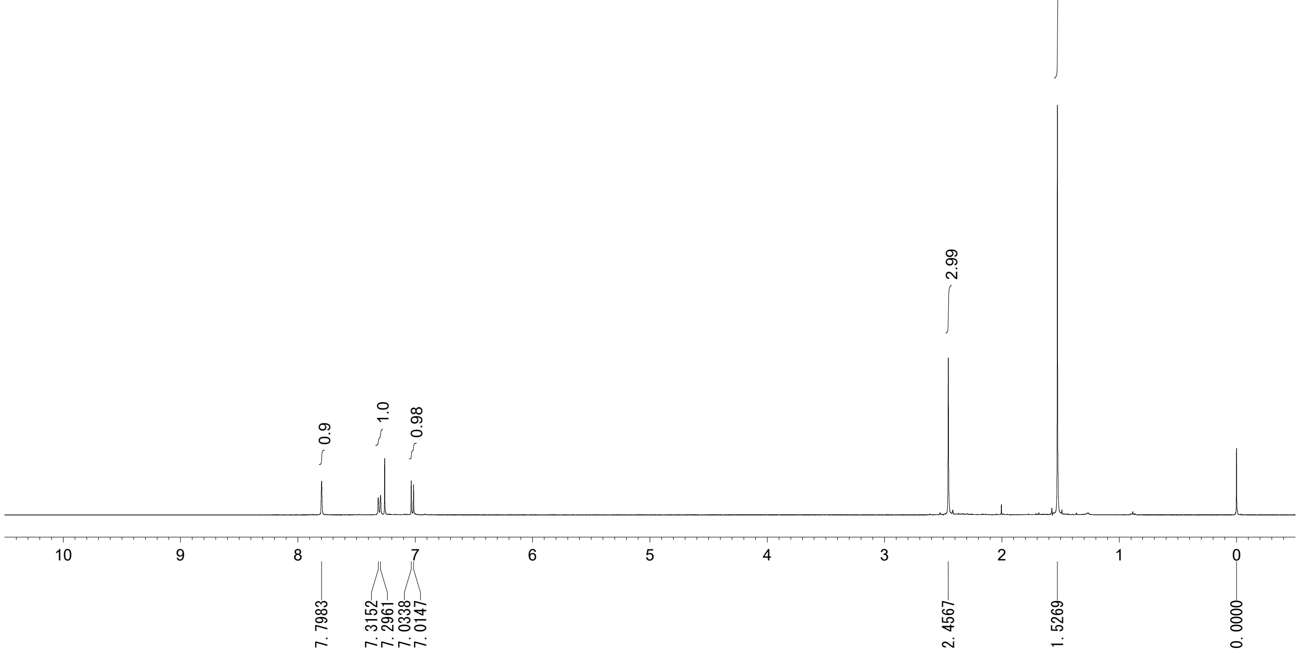

^{13}C{^{1}H} NMR (101 MHz, $CDCl_3$)

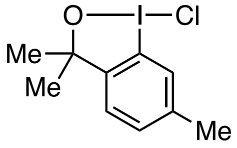

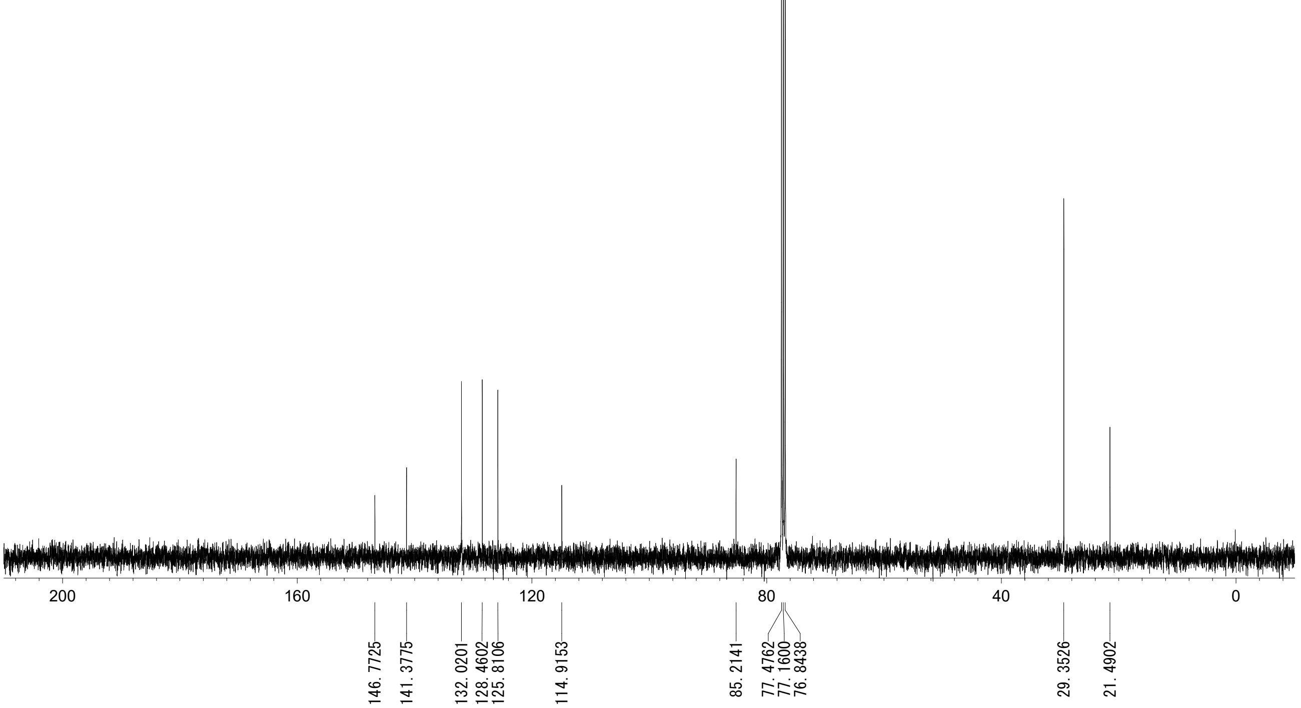

1-Chloro-3-methyl-3-(*p*-tolyl)-1,3-dihydro-1λ[3]-benzo[*d*][1,2]iodaoxole (S-3-2d)
^{1}H NMR (400 MHz, $CDCl_3$)

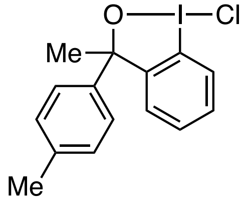

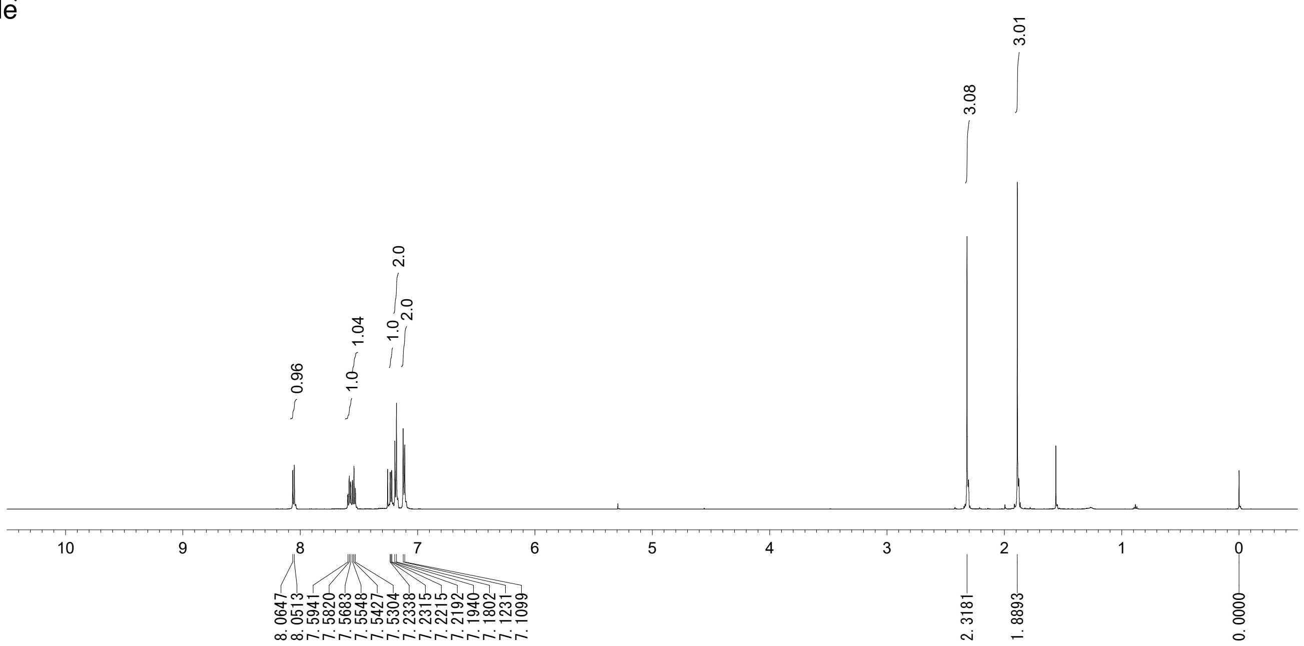

^{13}C{^{1}H} NMR (150 MHz, $CDCl_3$)

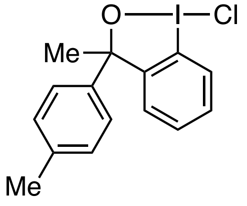

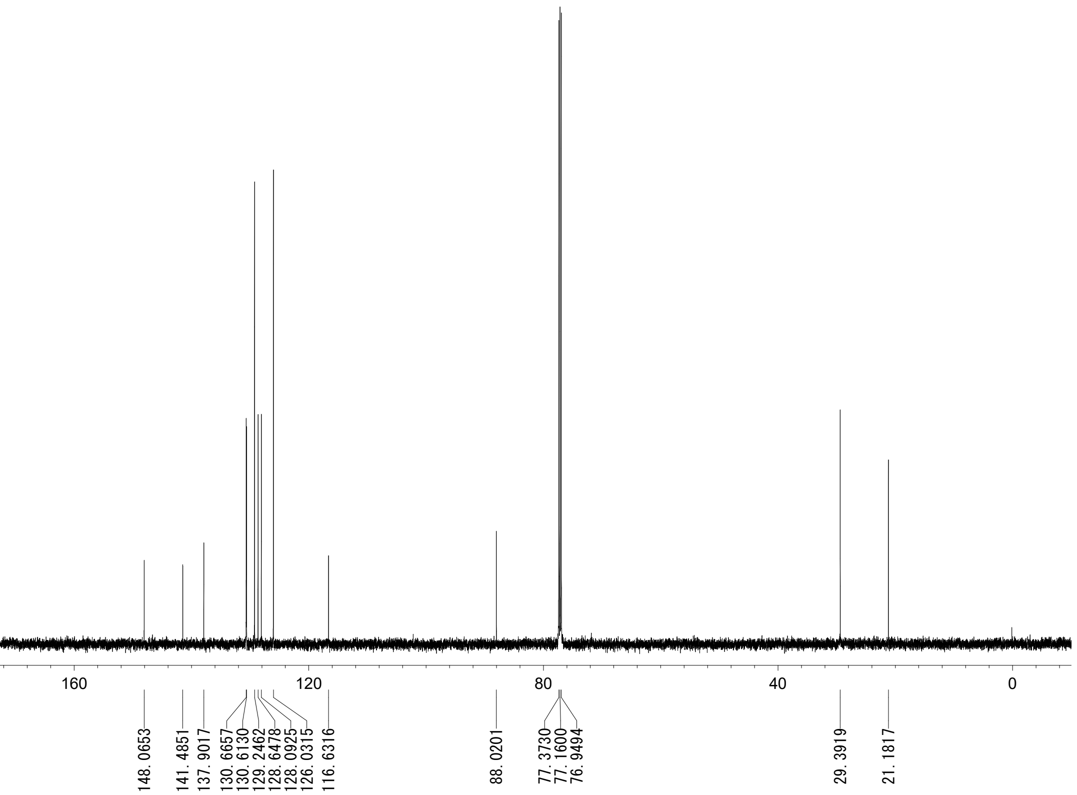

(2-(2-Hydroxypropan-2-yl)phenyl)(2,4,6-trimethoxyphenyl)iodonium chloride (2a)

^{1}H NMR (400 MHz, $CDCl_3$)

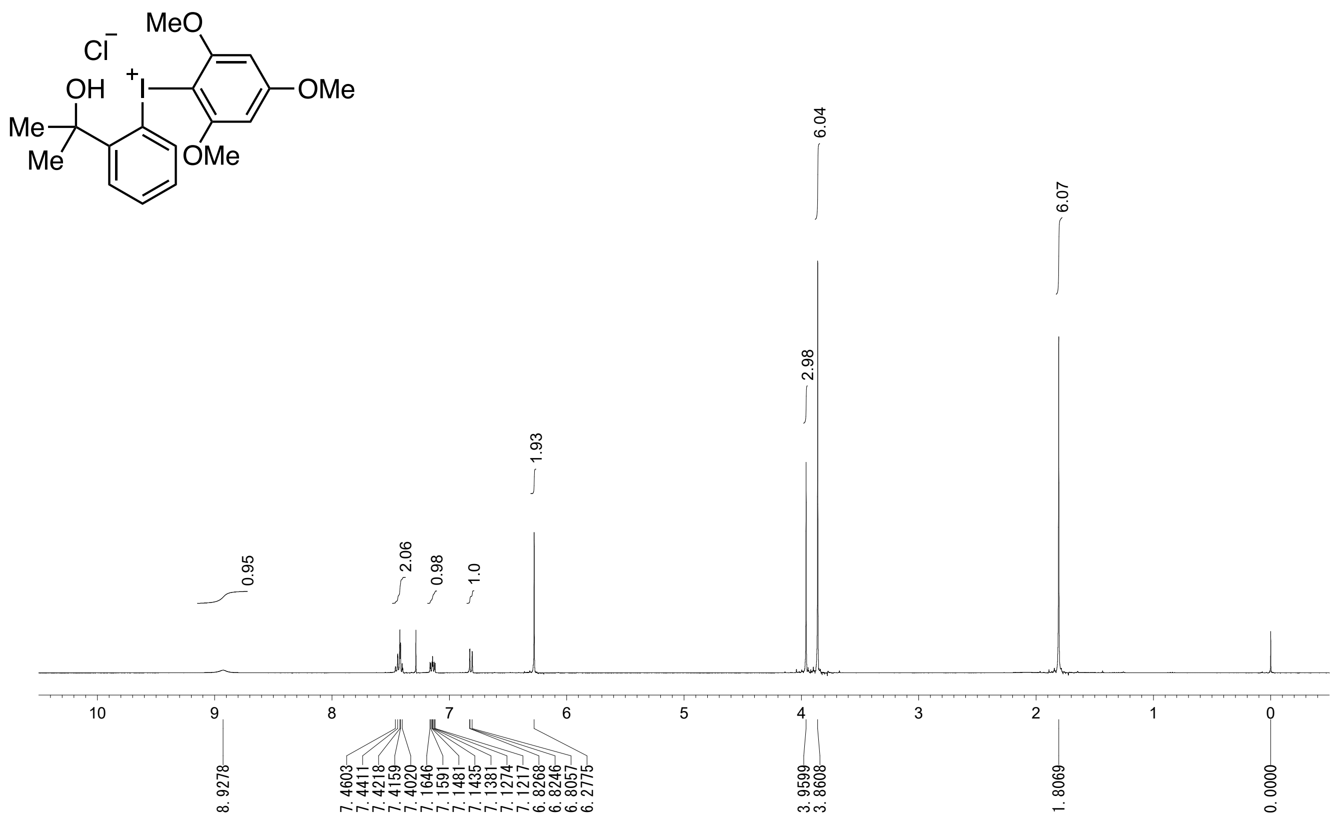

^{13}C{^{1}H} NMR (150 MHz, $CDCl_3$)

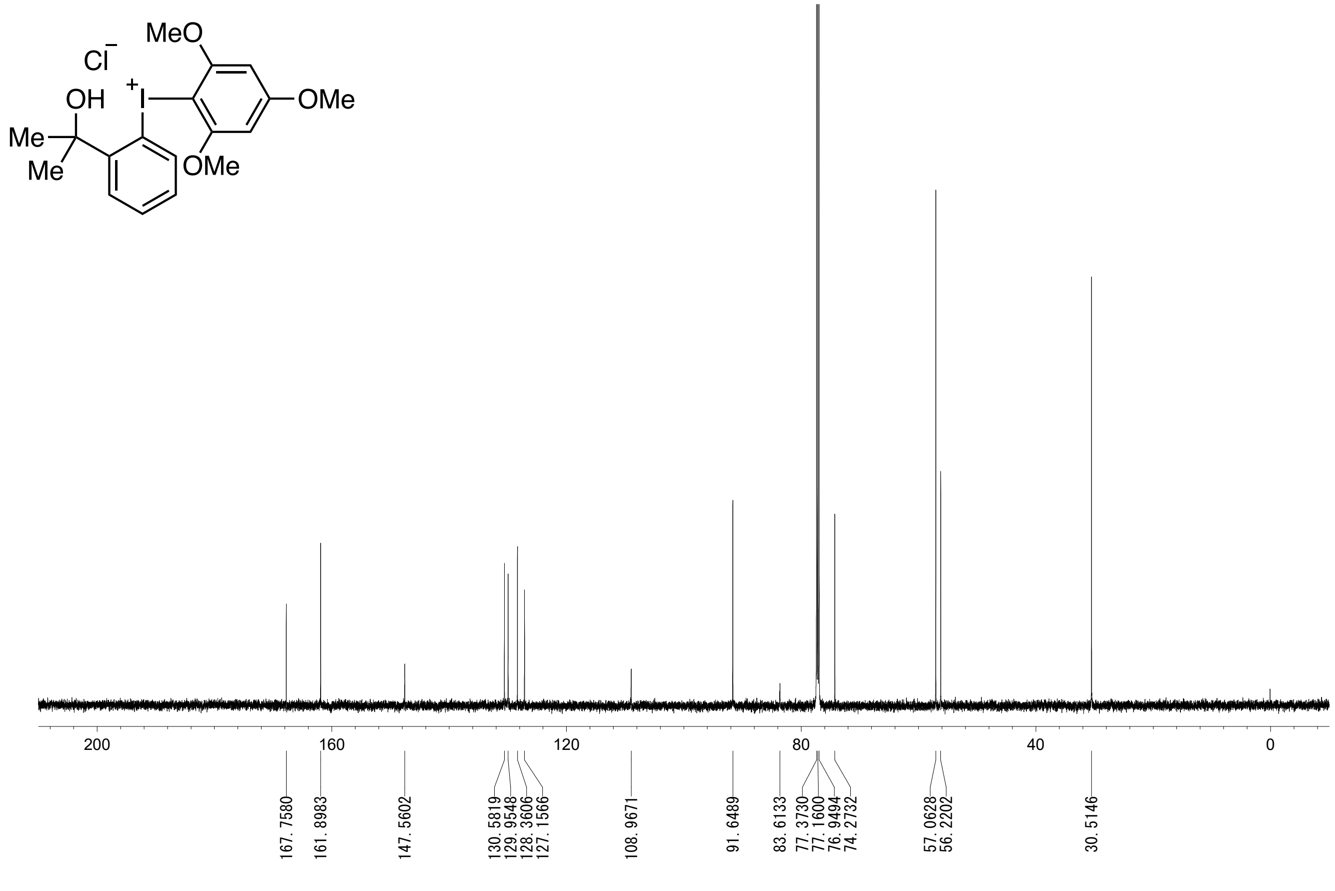

(2,6-Dimethoxyphenyl)(2-(2-hydroxypropan-2-yl)phenyl)iodonium chloride (2a-1)
^{1}H NMR (400 MHz, $CDCl_3$)

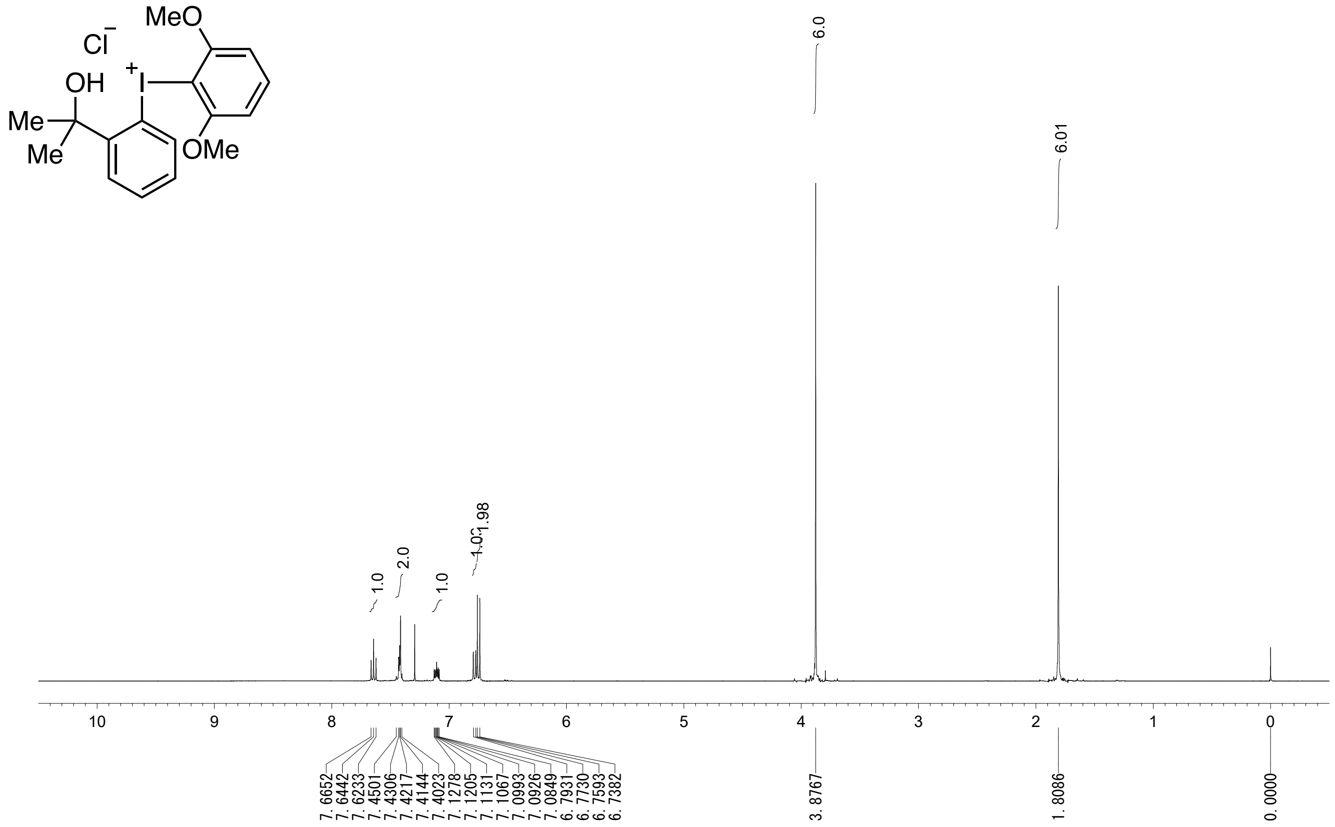

^{13}C{^{1}H} NMR (150 MHz, $CDCl_3$)

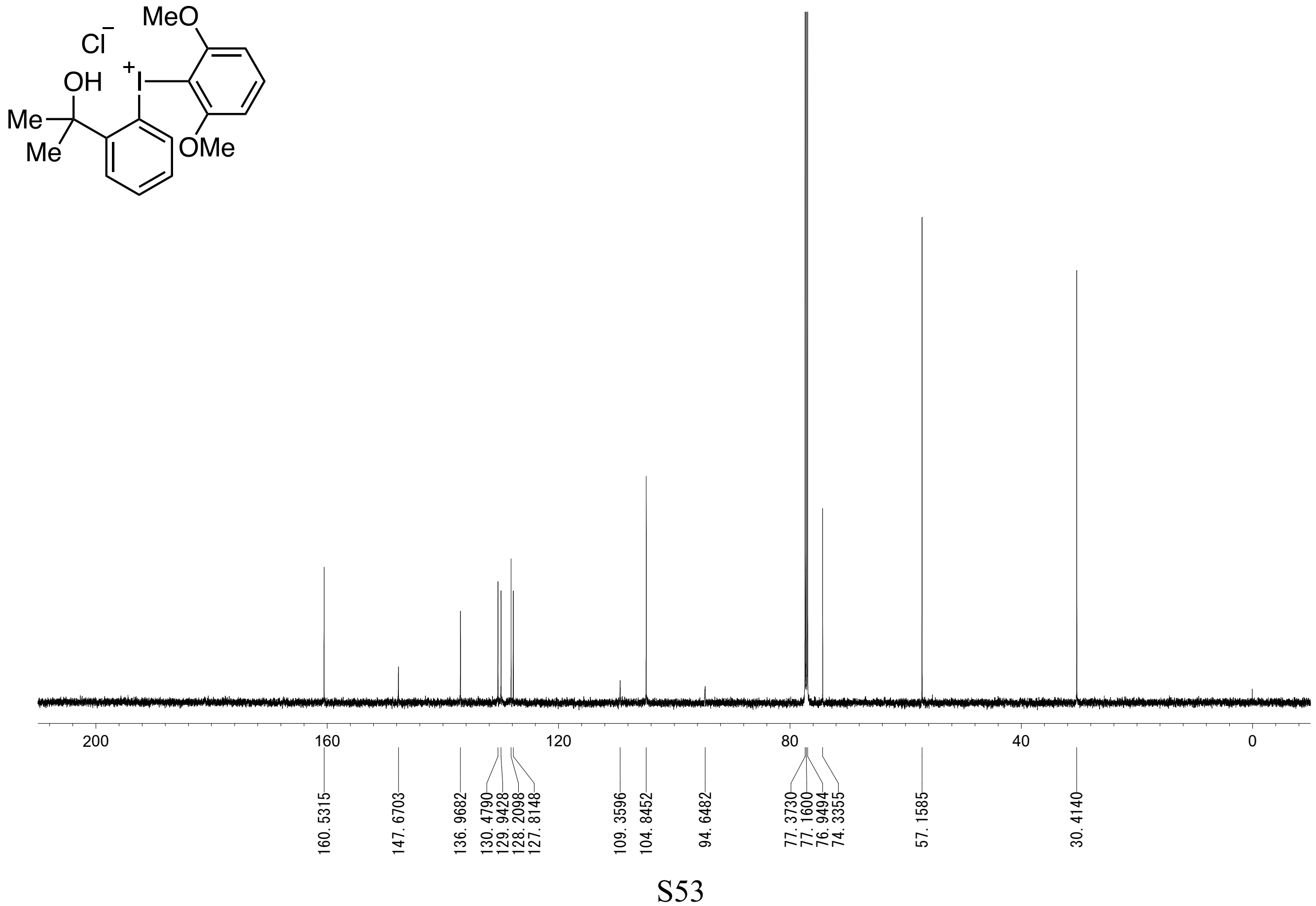

(2,4-Dimethoxyphenyl)(2-(2-hydroxypropan-2-yl)phenyl)iodonium chloride (2a-2)

^{1}H NMR (600 MHz, $CDCl_3$)

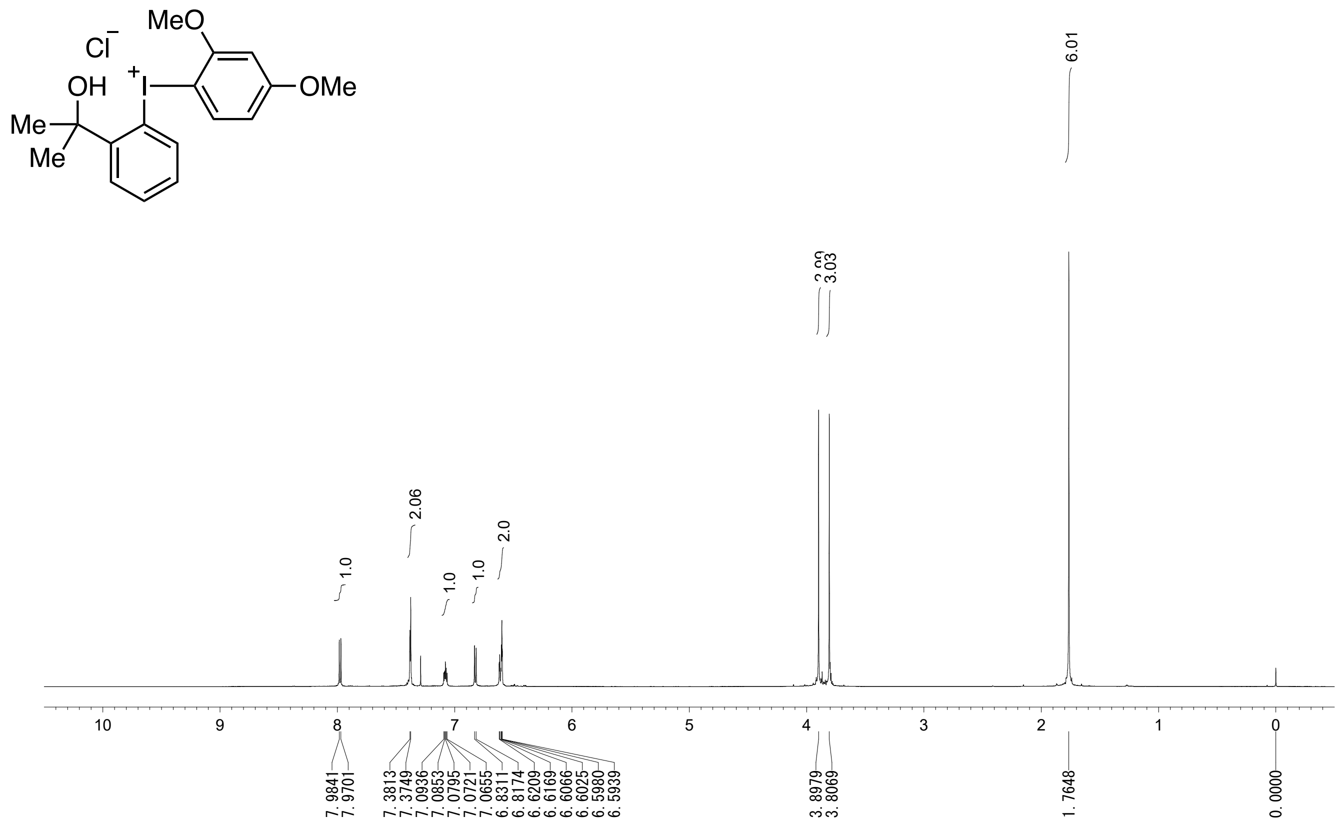

$^{13}C\{^{1}H\}$ NMR (150 MHz, $CDCl_3$)

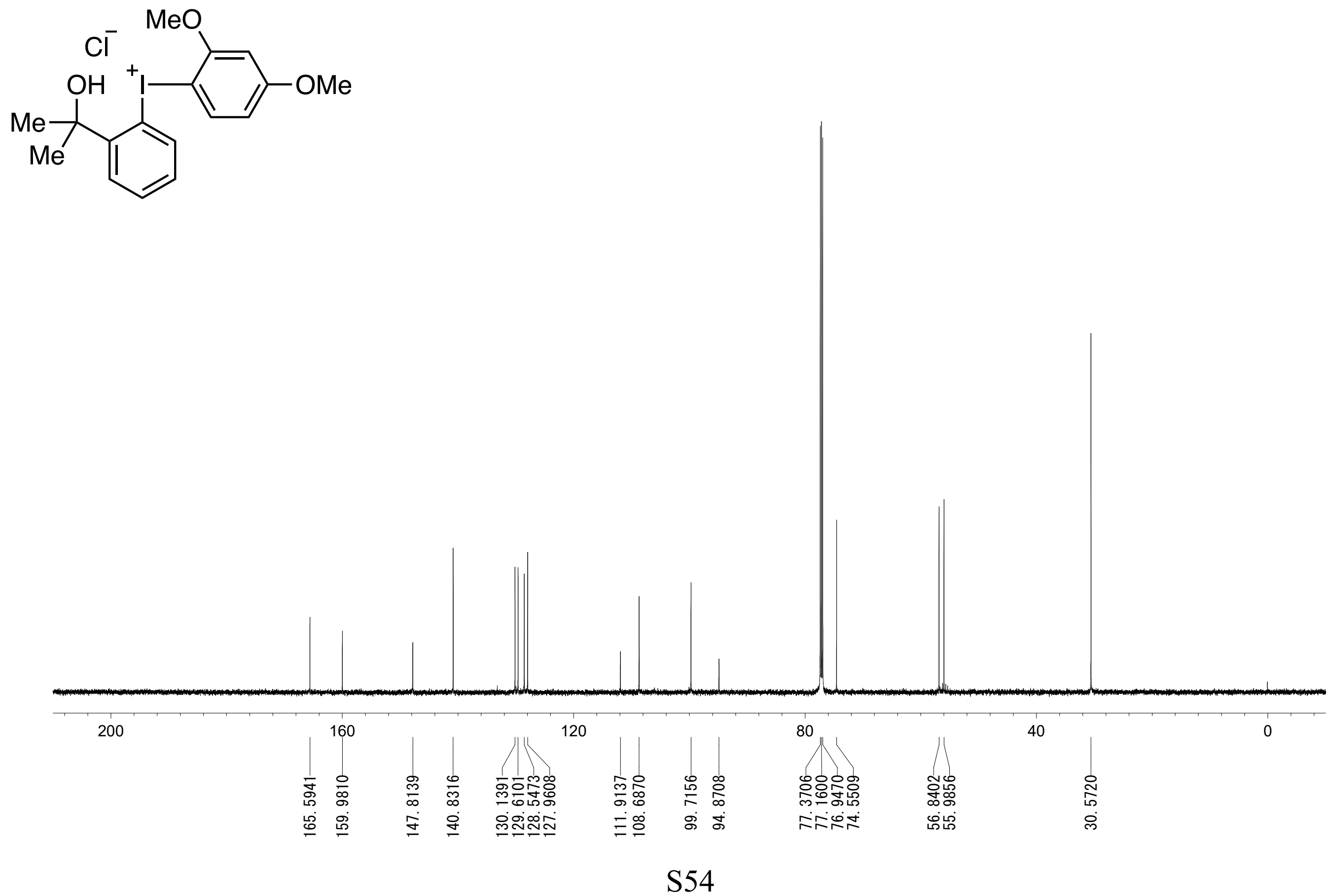

(2-(2-Hydroxypropan-2-yl)phenyl)(2-methoxyphenyl)iodonium chloride (2a-3)
^{1}H NMR (400 MHz, $CDCl_3$)

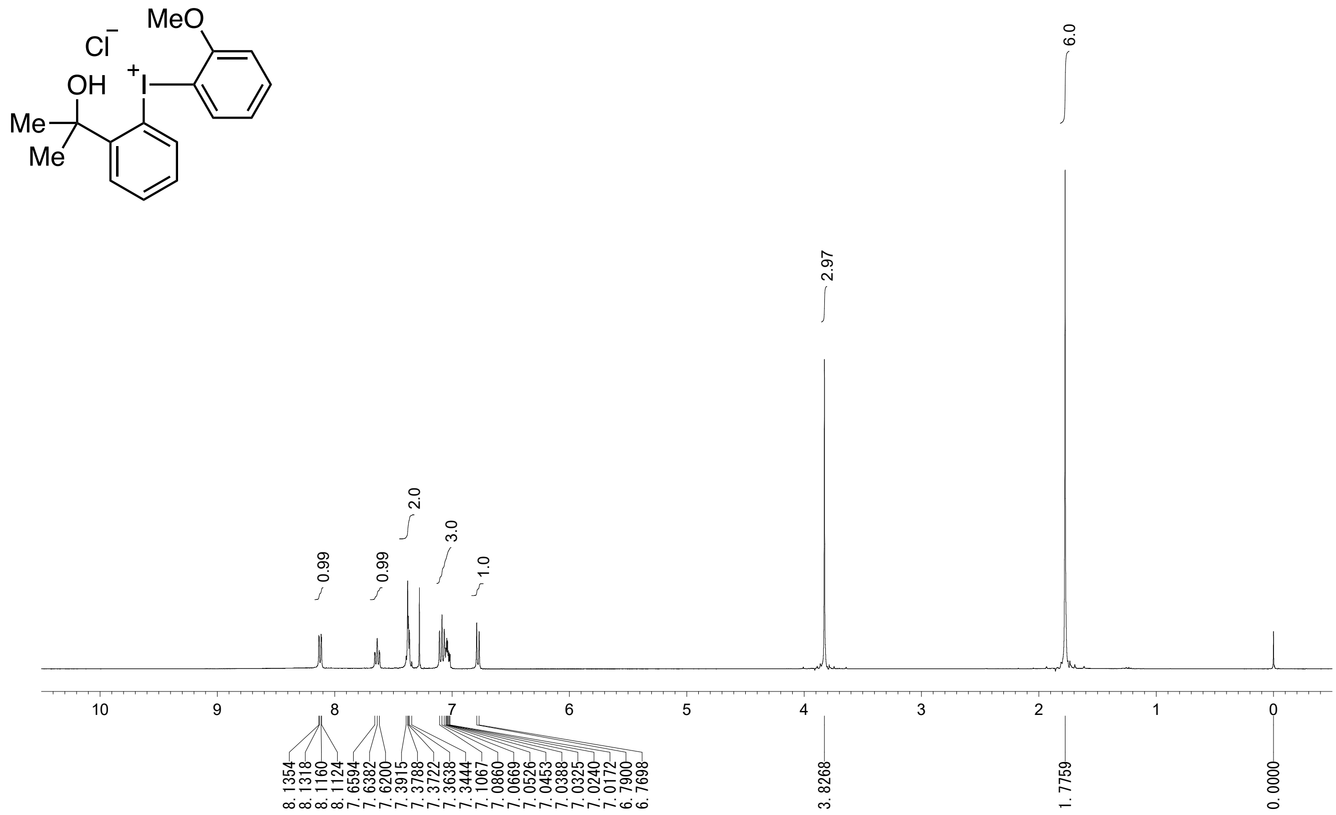

$^{13}C\{^{1}H\}$ NMR (150 MHz, $CDCl_3$)

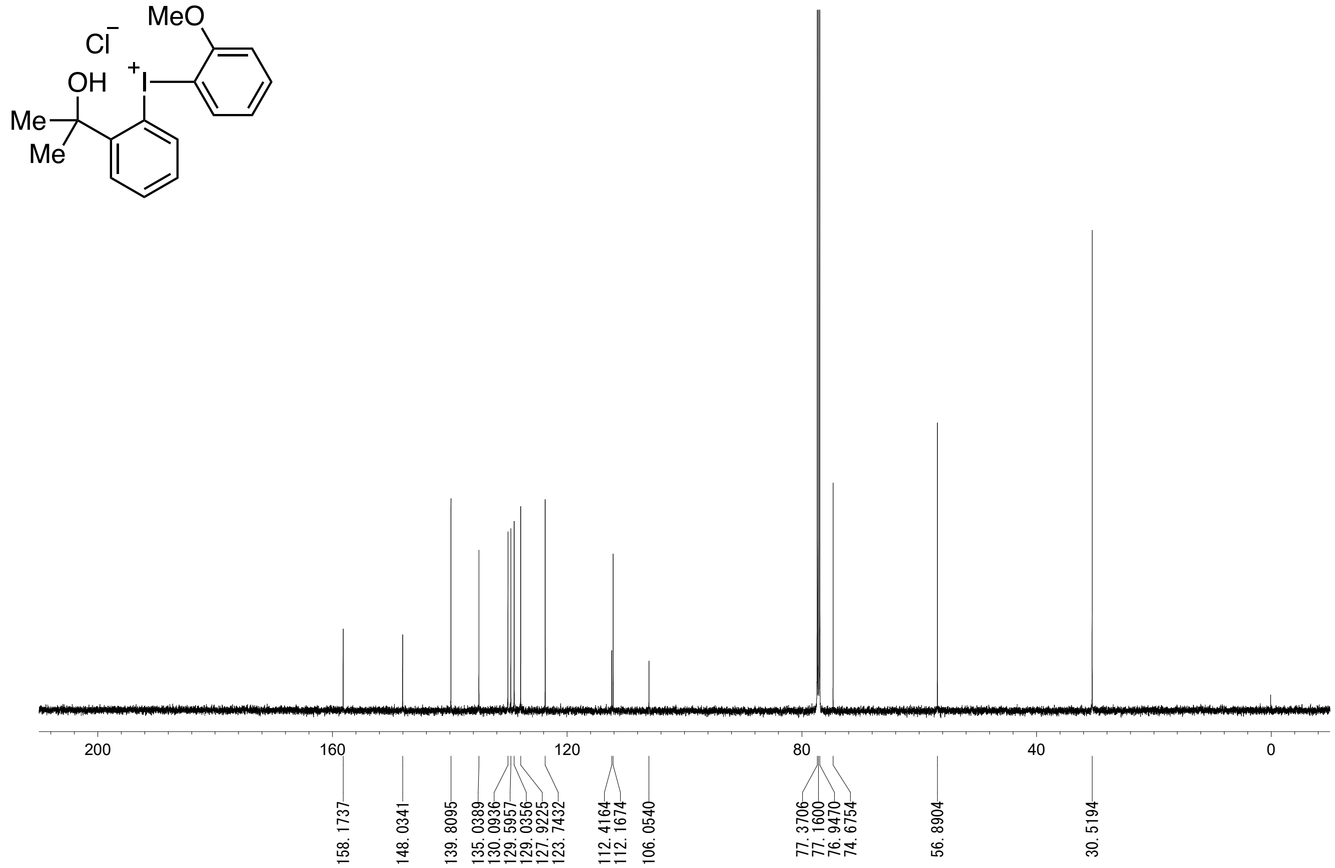

(2-(2-Hydroxypropan-2-yl)phenyl)(4-methoxyphenyl)iodonium chloride (2a-4)
^{1}H NMR (400 MHz, $CDCl_3$)

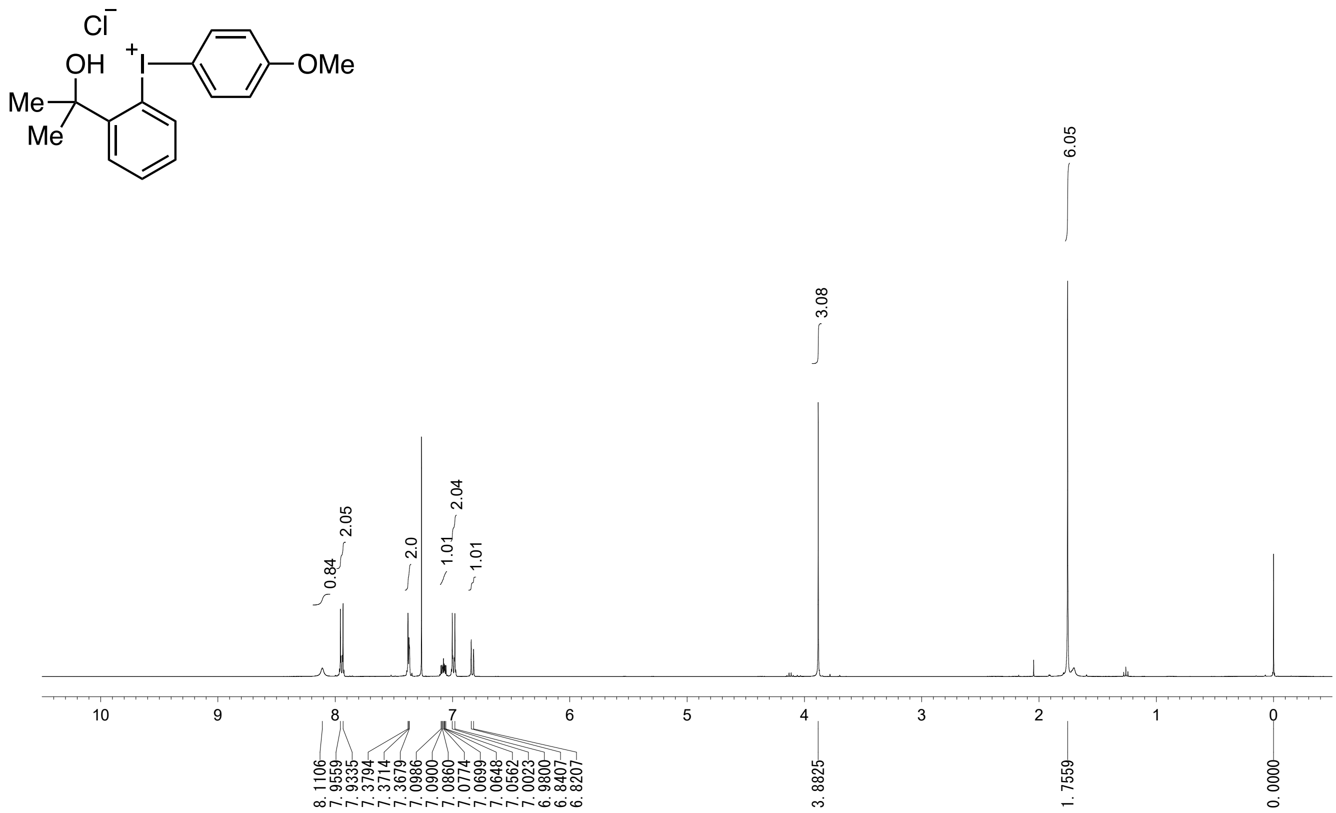

^{13}C{^{1}H} NMR (150 MHz, $CDCl_3$)

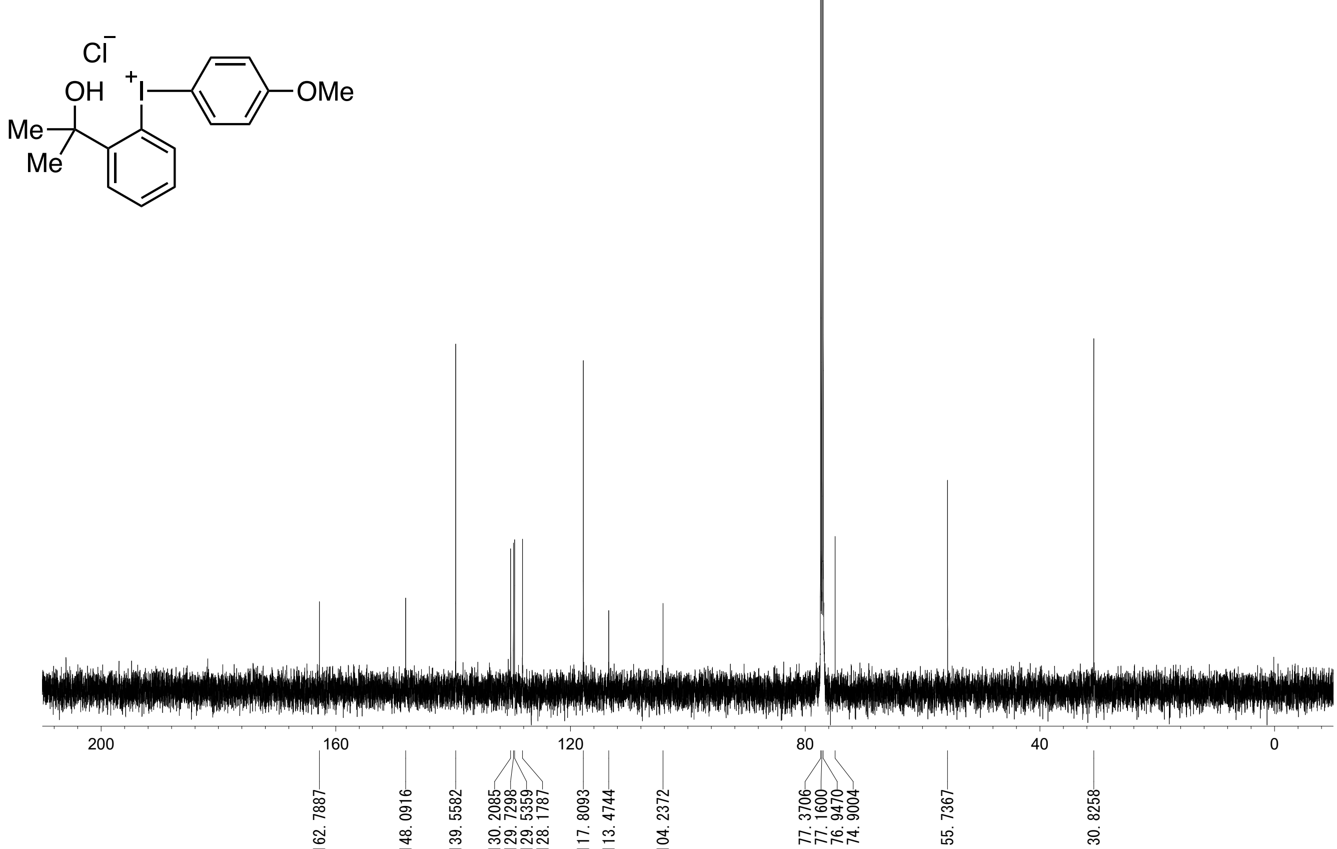

(2-(2-Hydroxypropan-2-yl)phenyl)(mesityl)iodonium chloride (2a-5)
^{1}H NMR (600 MHz, $CDCl_3$)

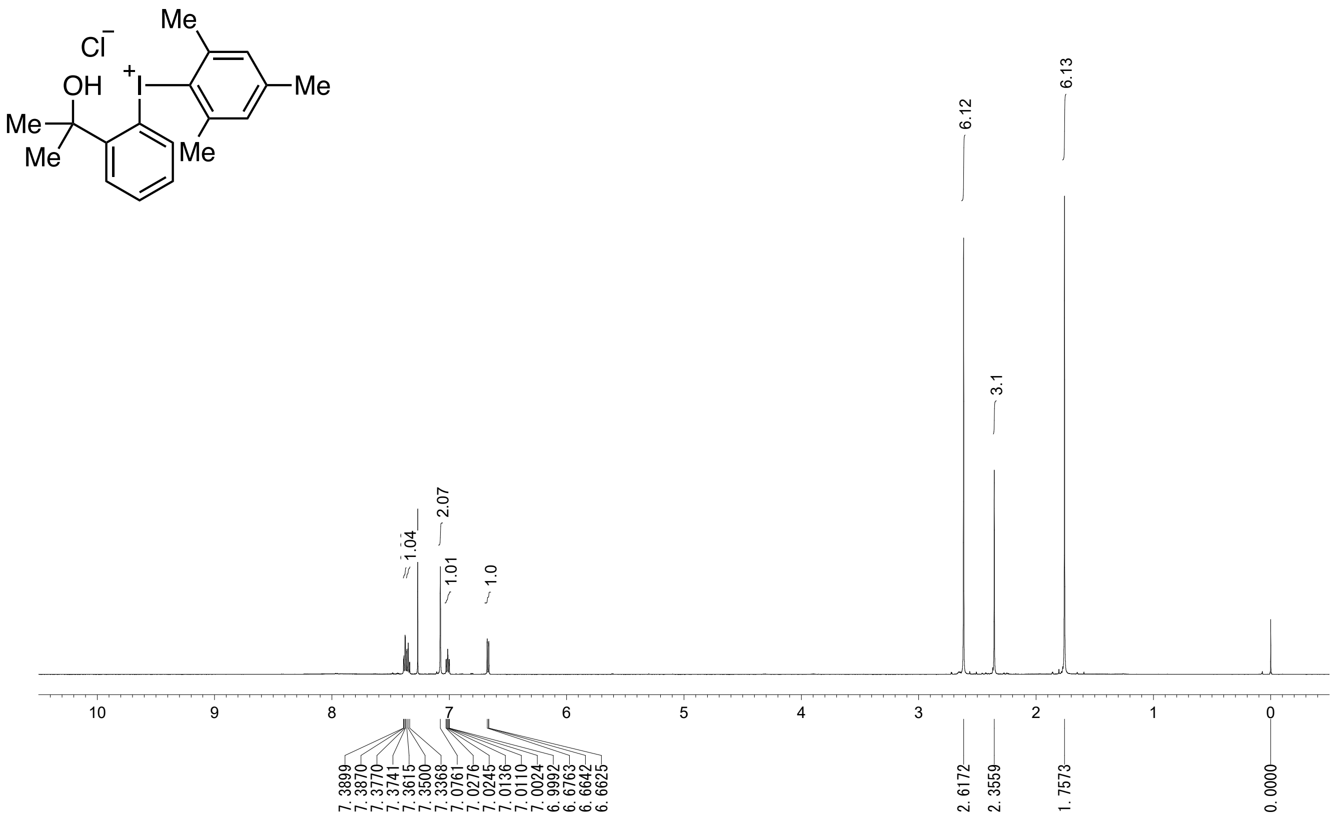

^{13}C{^{1}H} NMR (150 MHz, $CDCl_3$)

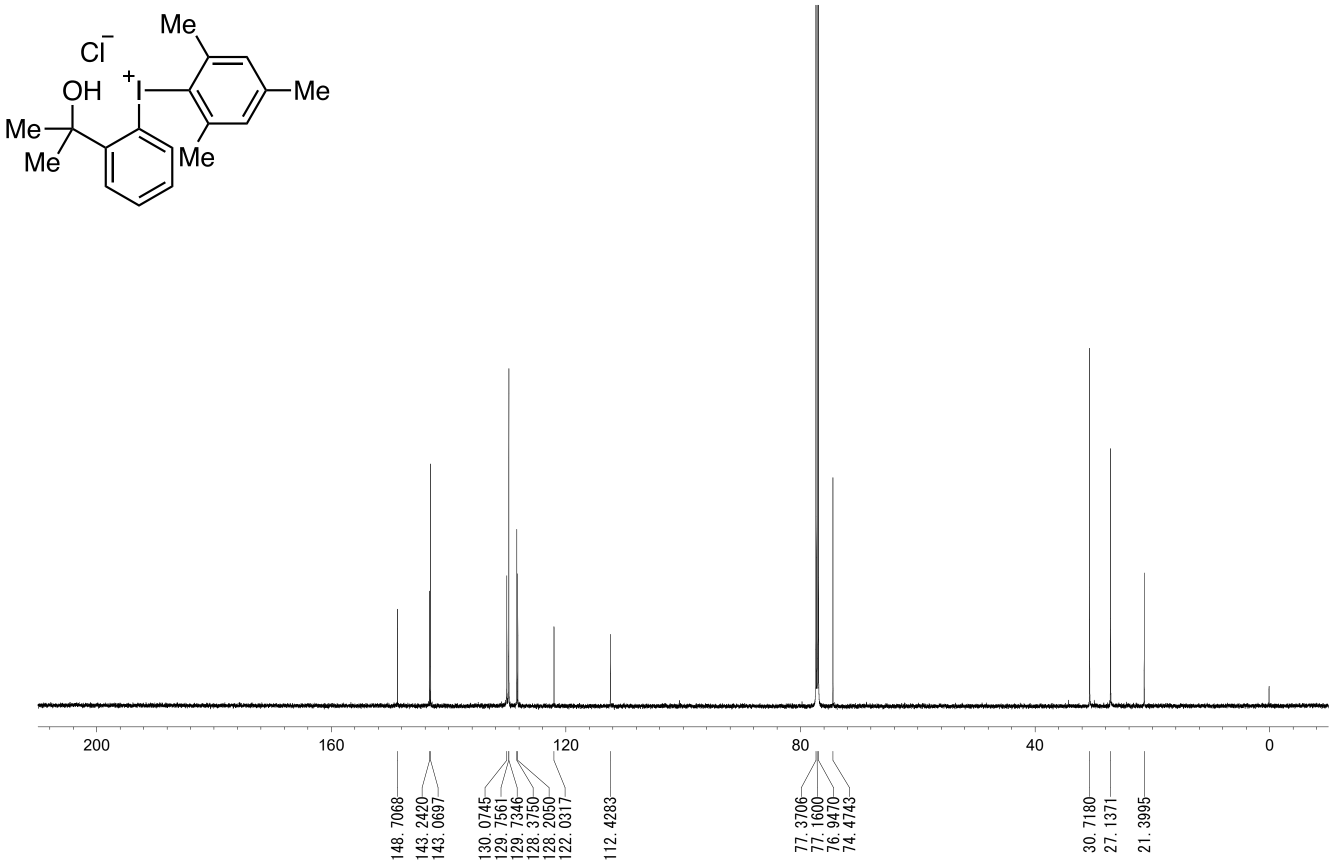

(2-(2-Hydroxypropan-2-yl)phenyl)(phenyl)iodonium chloride (2a-6)
^{1}H NMR (600 MHz, $CDCl_3$)

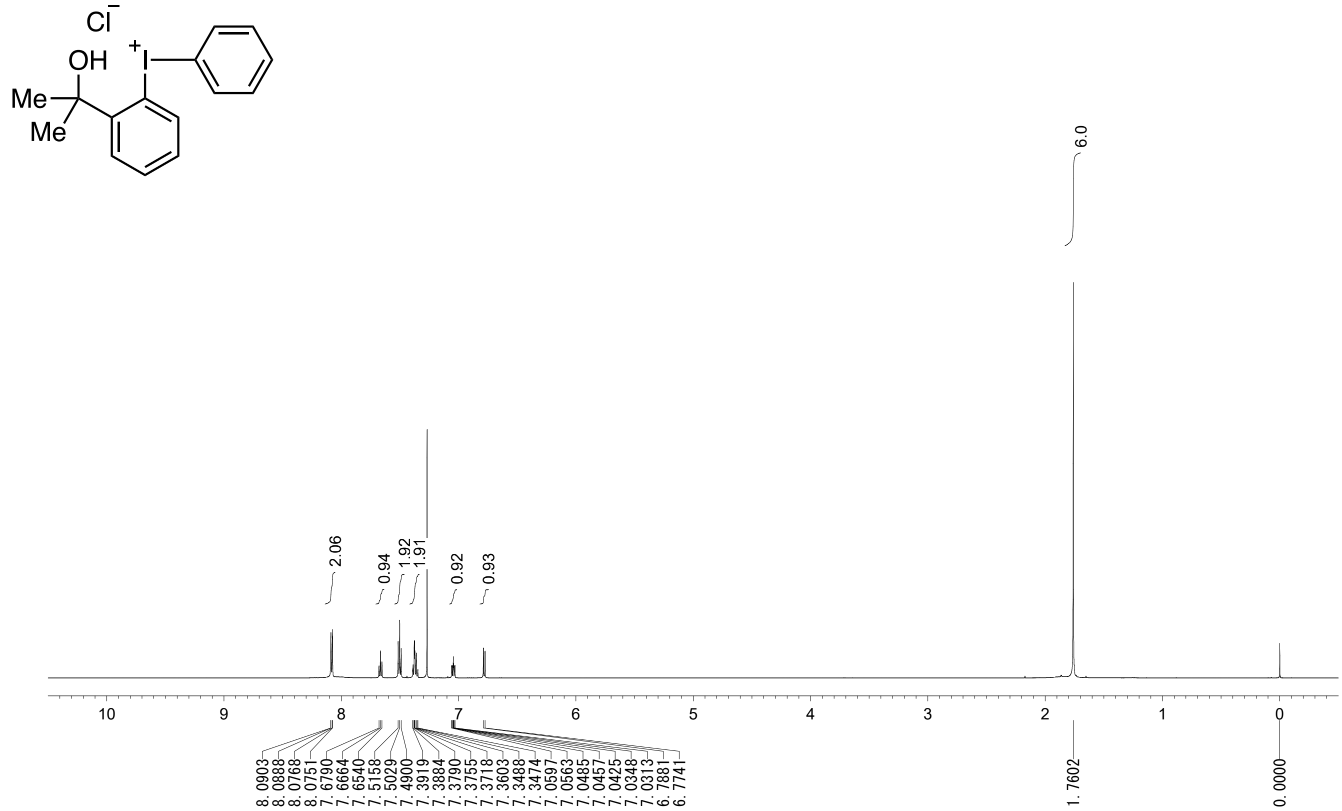

^{13}C{^{1}H} NMR (150 MHz, $CDCl_3$)

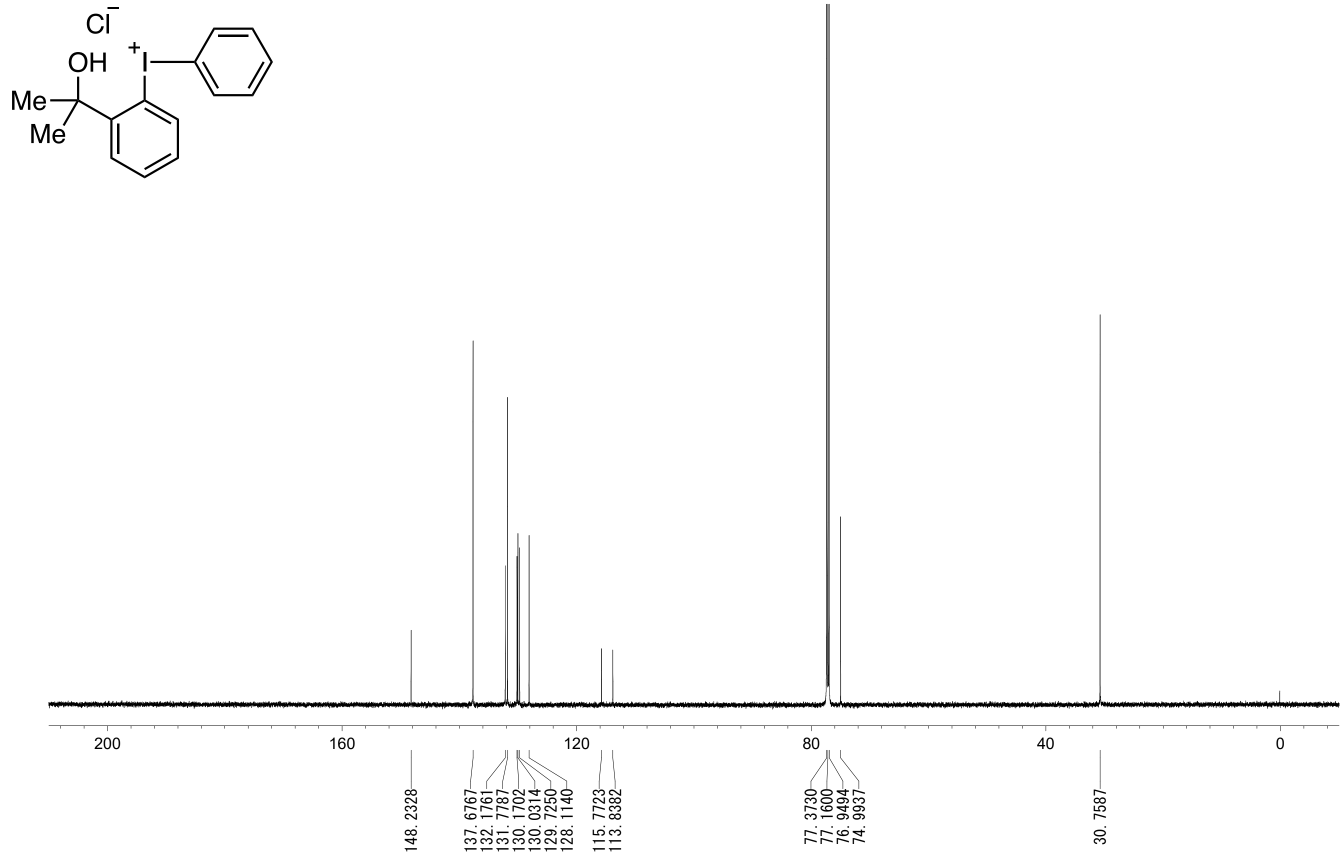

(2-(3-Hydroxypentan-3-yl)phenyl)(2,4,6-trimethoxyphenyl)iodonium chloride (2b)
^{1}H NMR (600 MHz, $CDCl_3$)

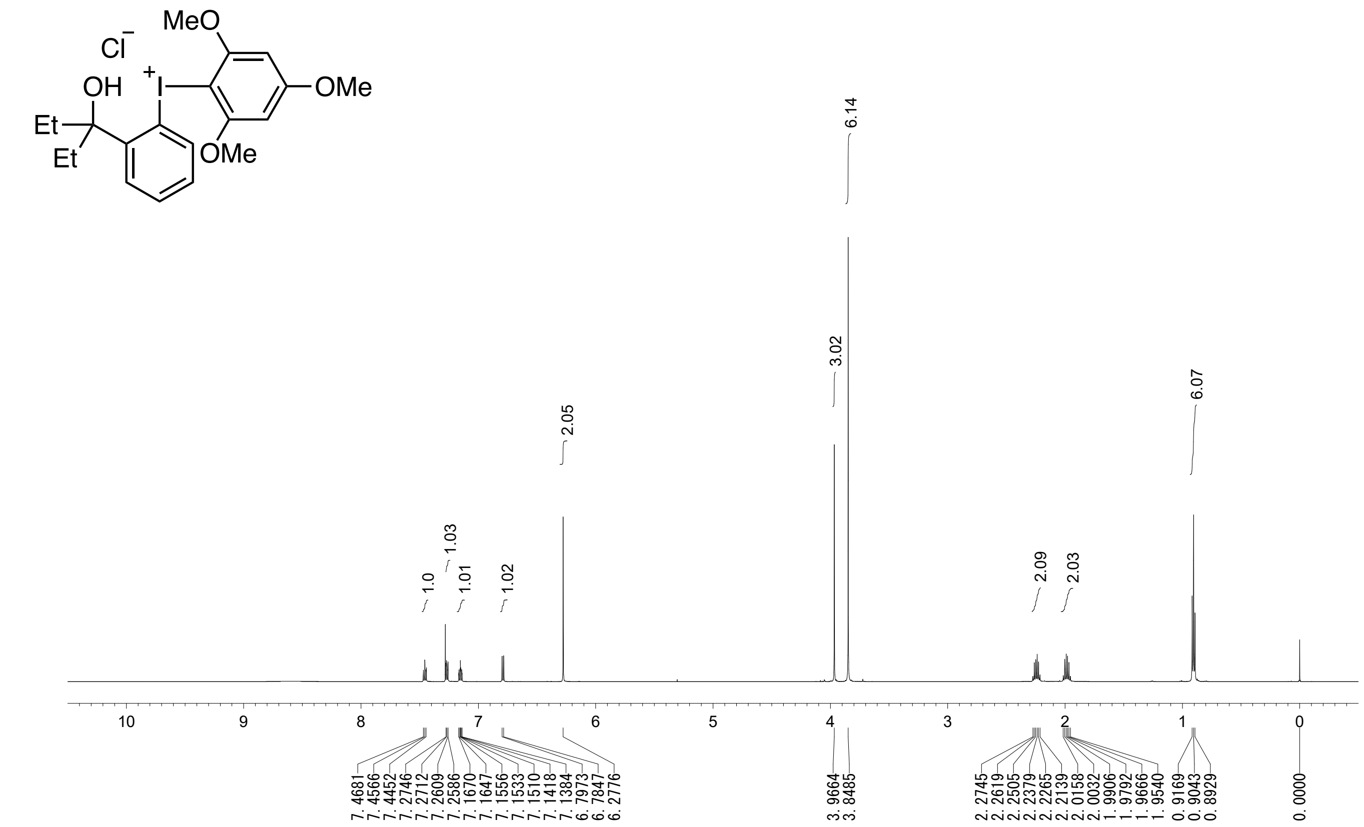

^{13}C{^{1}H} NMR (150 MHz, $CDCl_3$)

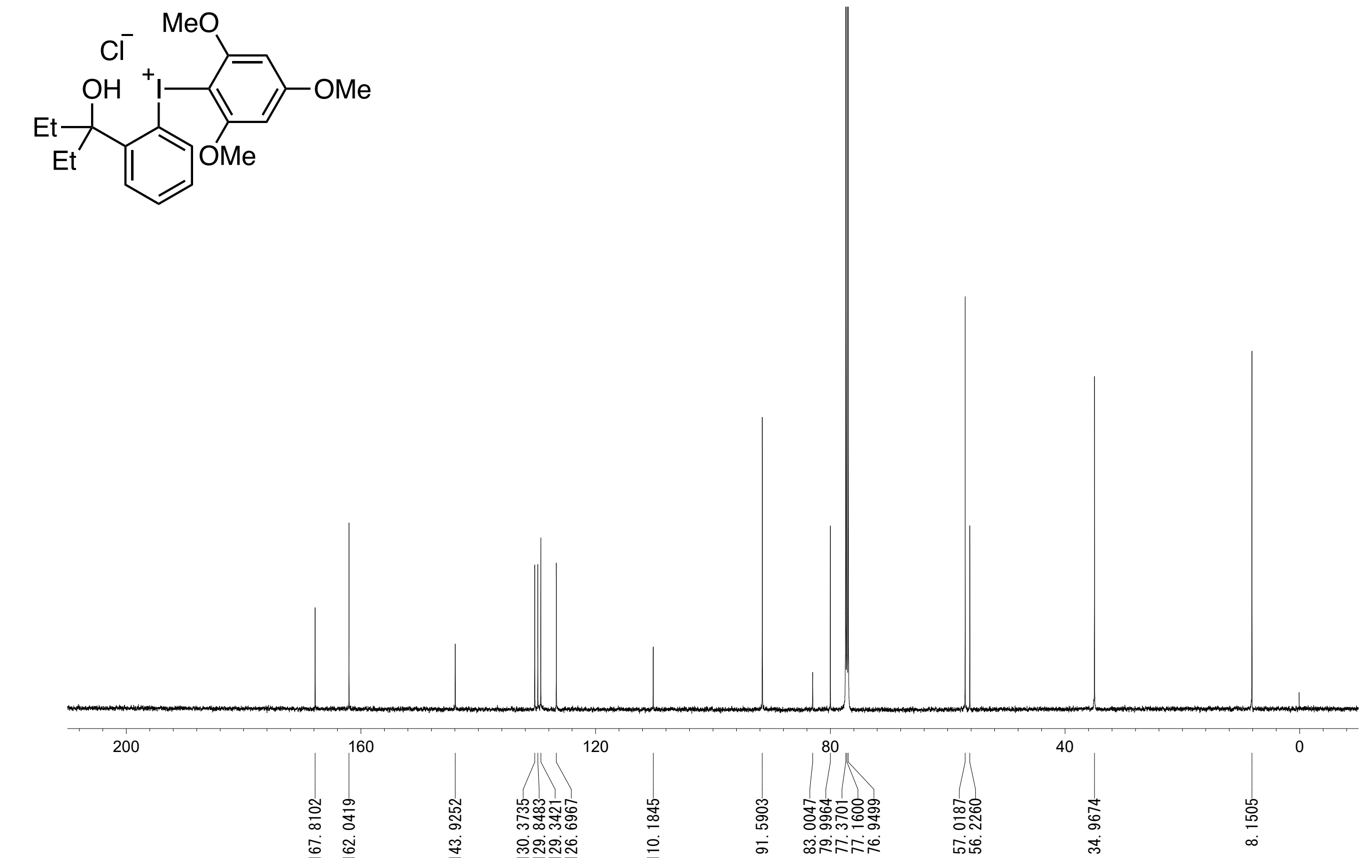

(2-(1-Hydroxy-1-phenylethyl)phenyl)(2,4,6-trimethoxyphenyl)iodonium chloride (2c)
^{1}H NMR (400 MHz, $CDCl_3$)

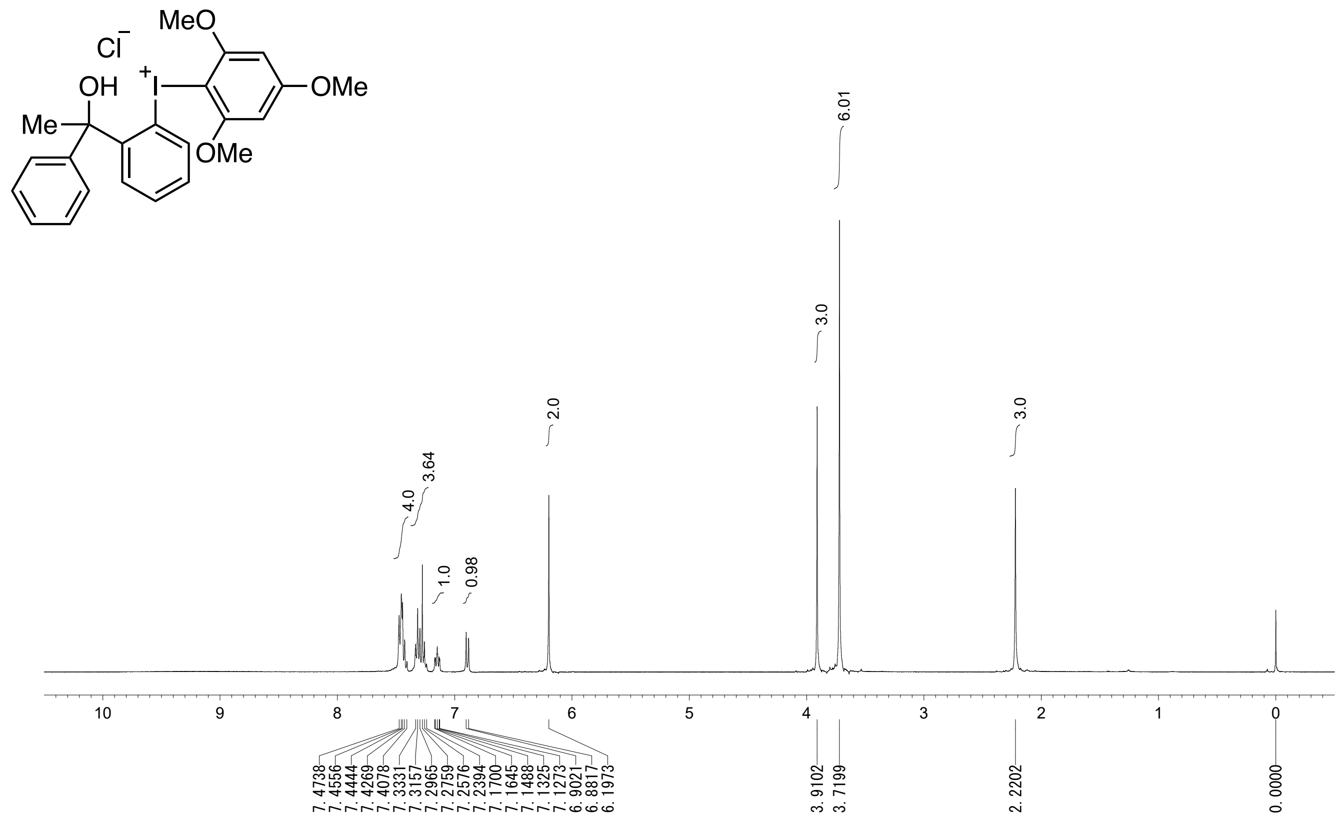

^{13}C{^{1}H} NMR (150 MHz, $CDCl_3$)

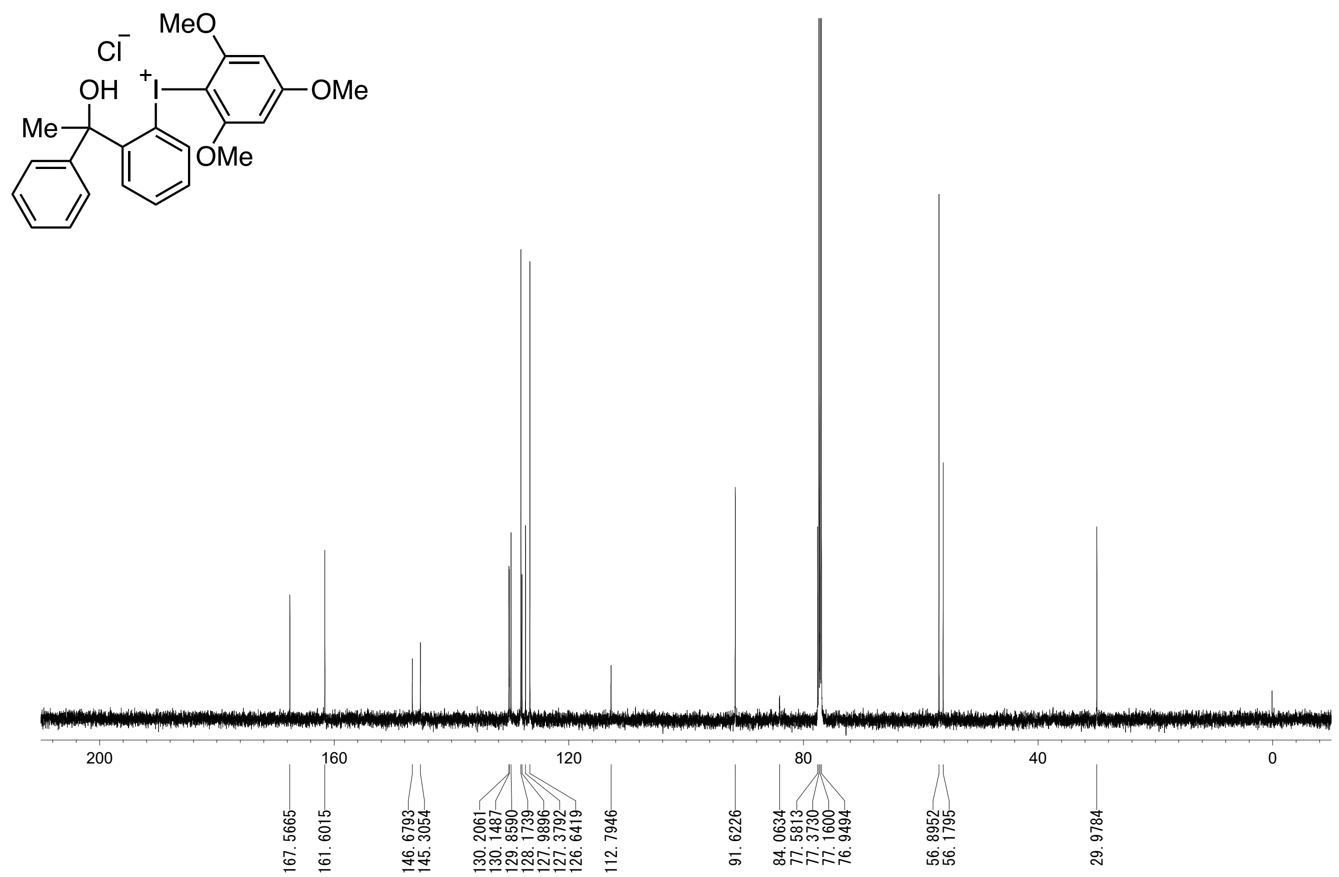

(2-(1-Hydroxy-1-(p-tolyl)ethyl)phenyl)(2,4,6-trimethoxyphenyl)iodonium chloride (2d)
^{1}H NMR (400 MHz, $CDCl_3$)

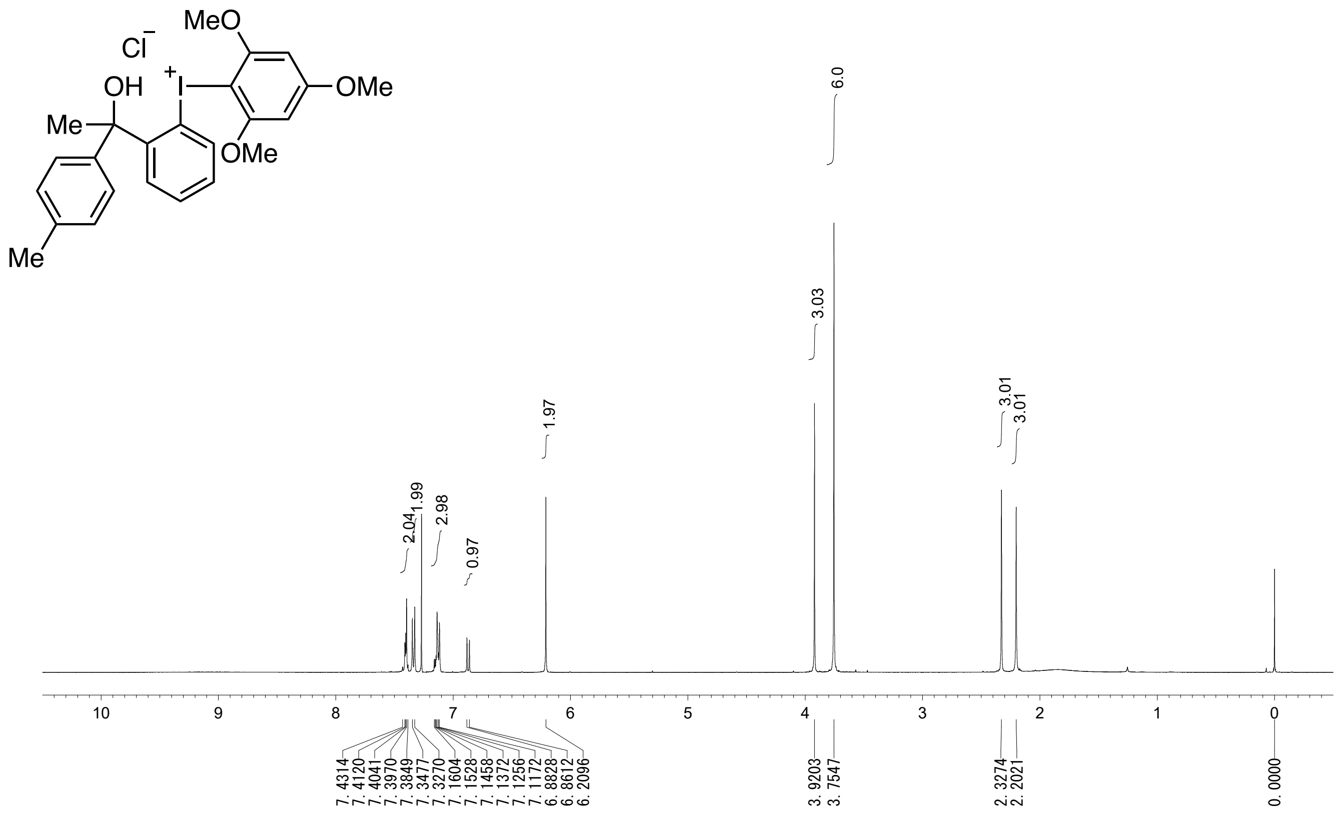

^{13}C{^{1}H} NMR (150 MHz, $CDCl_3$)

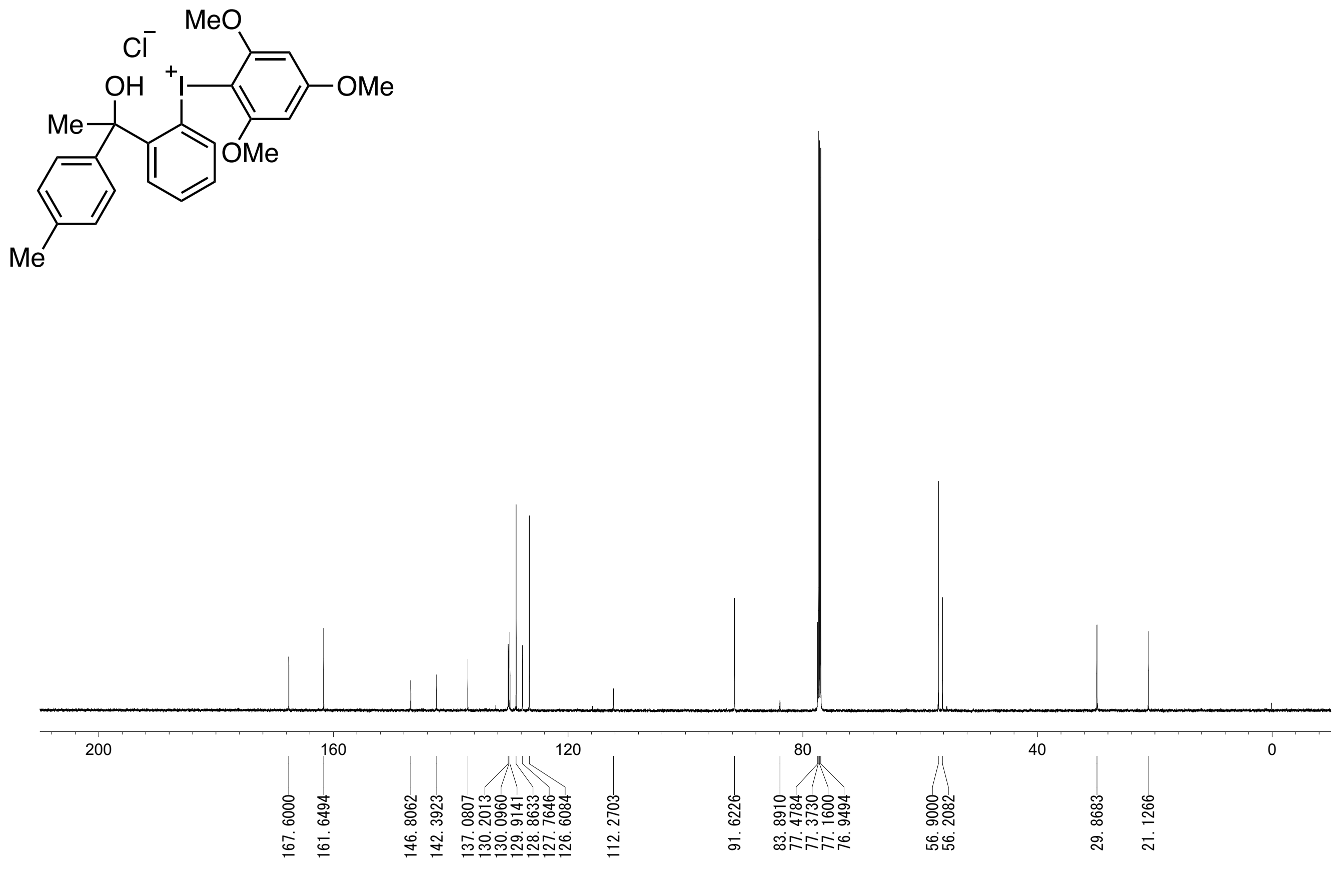

(2-(2-Hydroxypropan-2-yl)-4-methylphenyl)(2,4,6-trimethoxyphenyl)iodonium chloride (2f)
^{1}H NMR (400 MHz, $CDCl_3$)

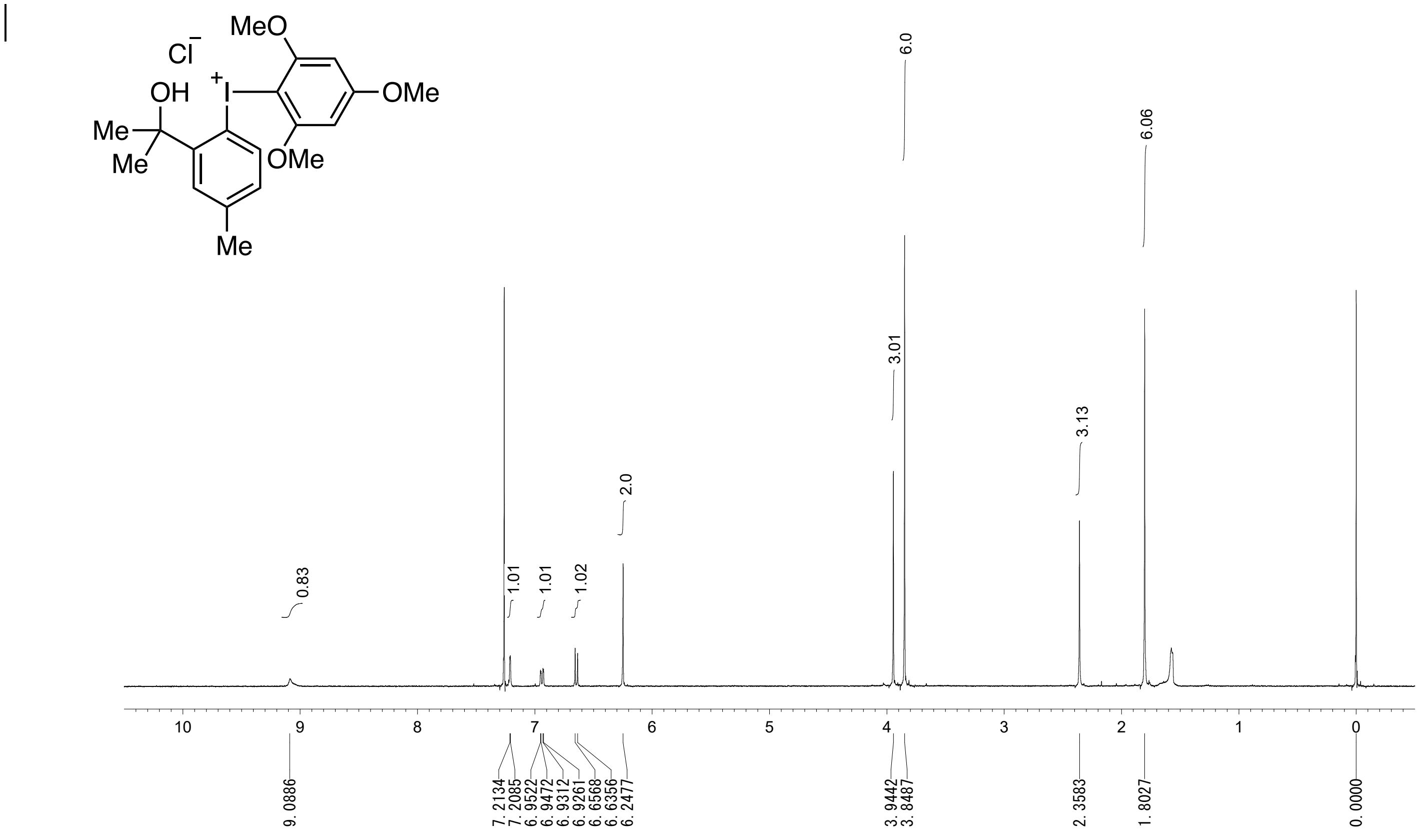

^{13}C{^{1}H} NMR (150 MHz, $CDCl_3$)

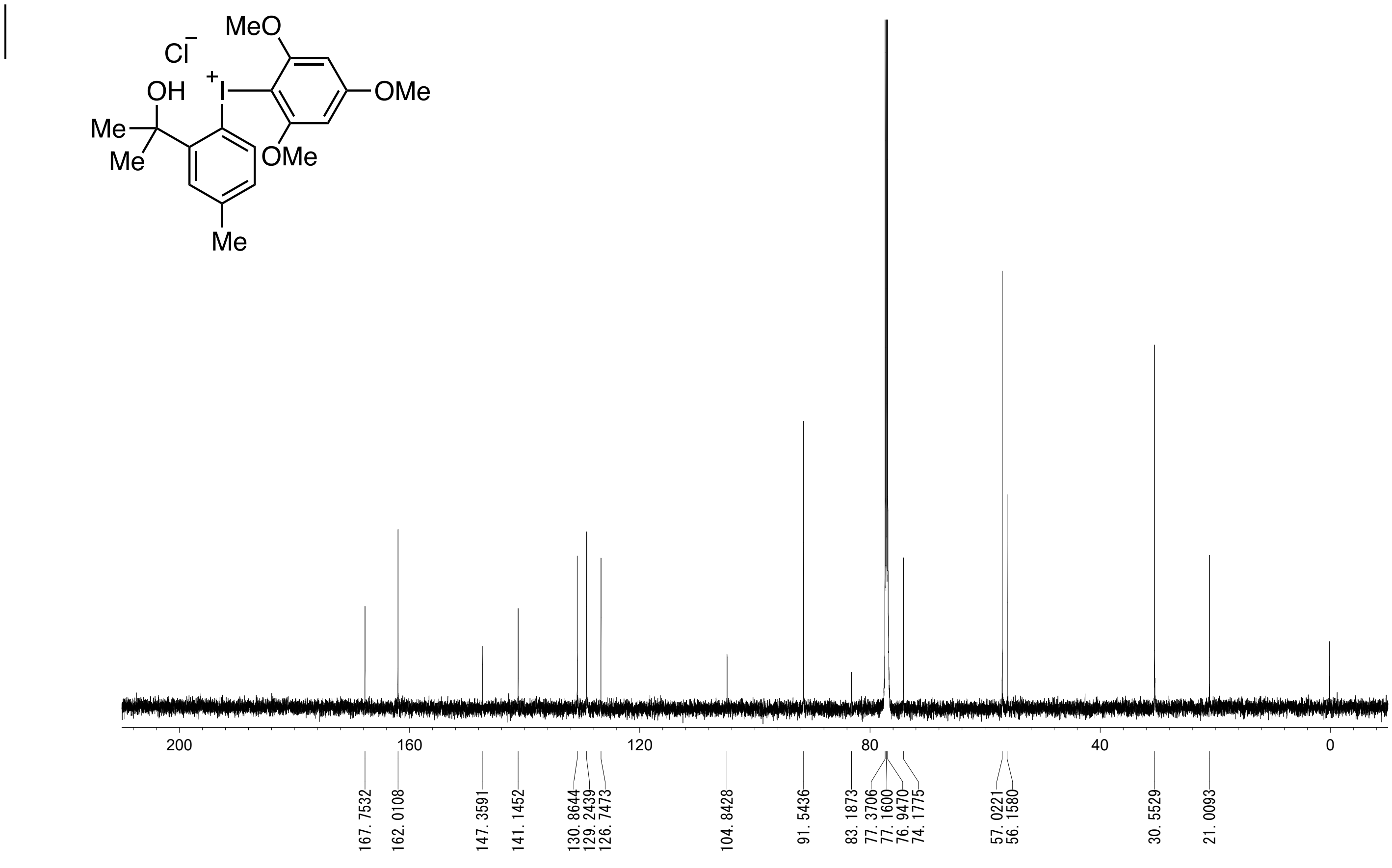

(2-(2-Hydroxypropan-2-yl)-4-methoxyphenyl)(2,4,6-trimethoxyphenyl)iodonium chloride (2g)
^{1}H NMR (600 MHz, $CDCl_3$)

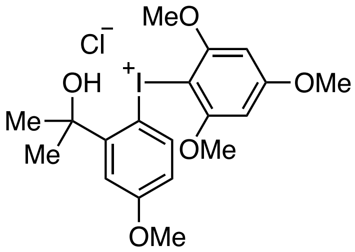

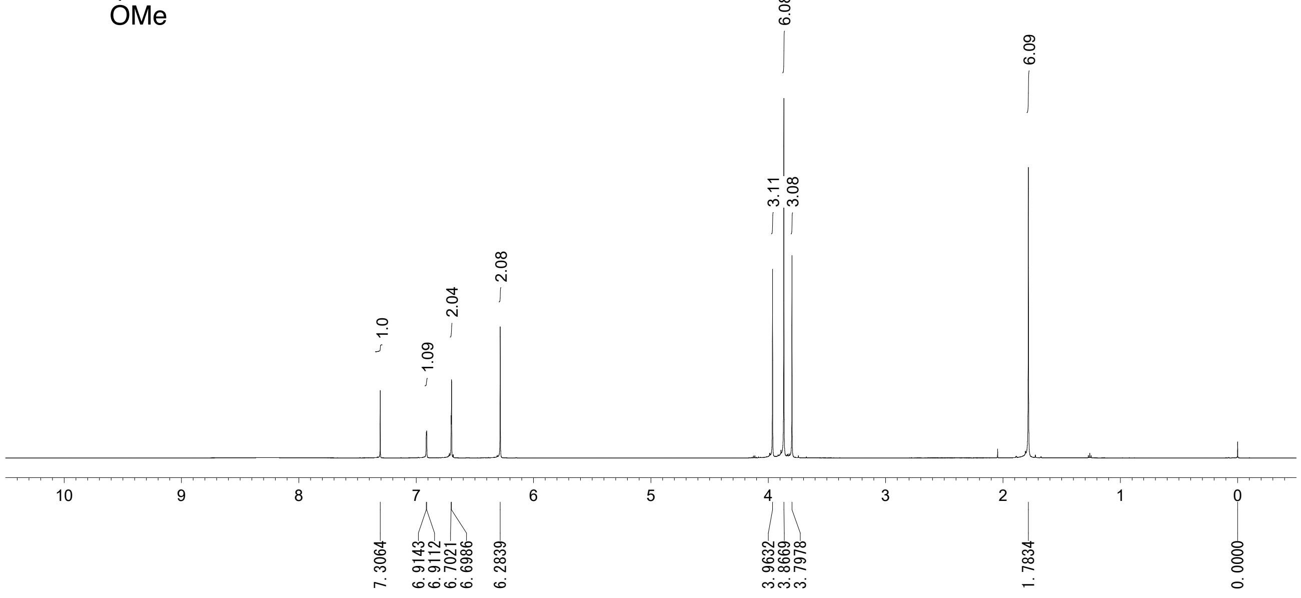

$^{13}C\{^{1}H\}$ NMR (150 MHz, $CDCl_3$)

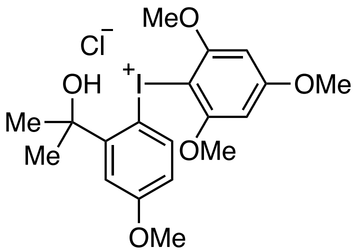

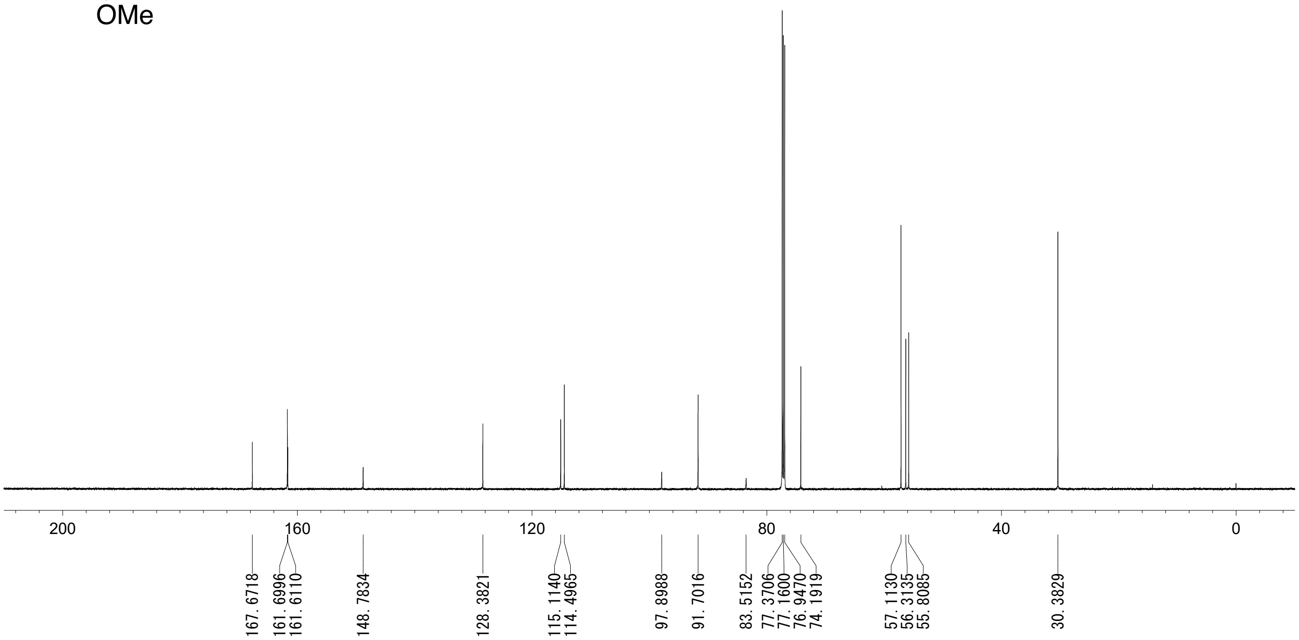

(4-Bromo-2-(2-hydroxypropan-2-yl)phenyl)(2,4,6-trimethoxyphenyl)iodonium chloride (2h)
^{1}H NMR (600 MHz, $CDCl_3$)

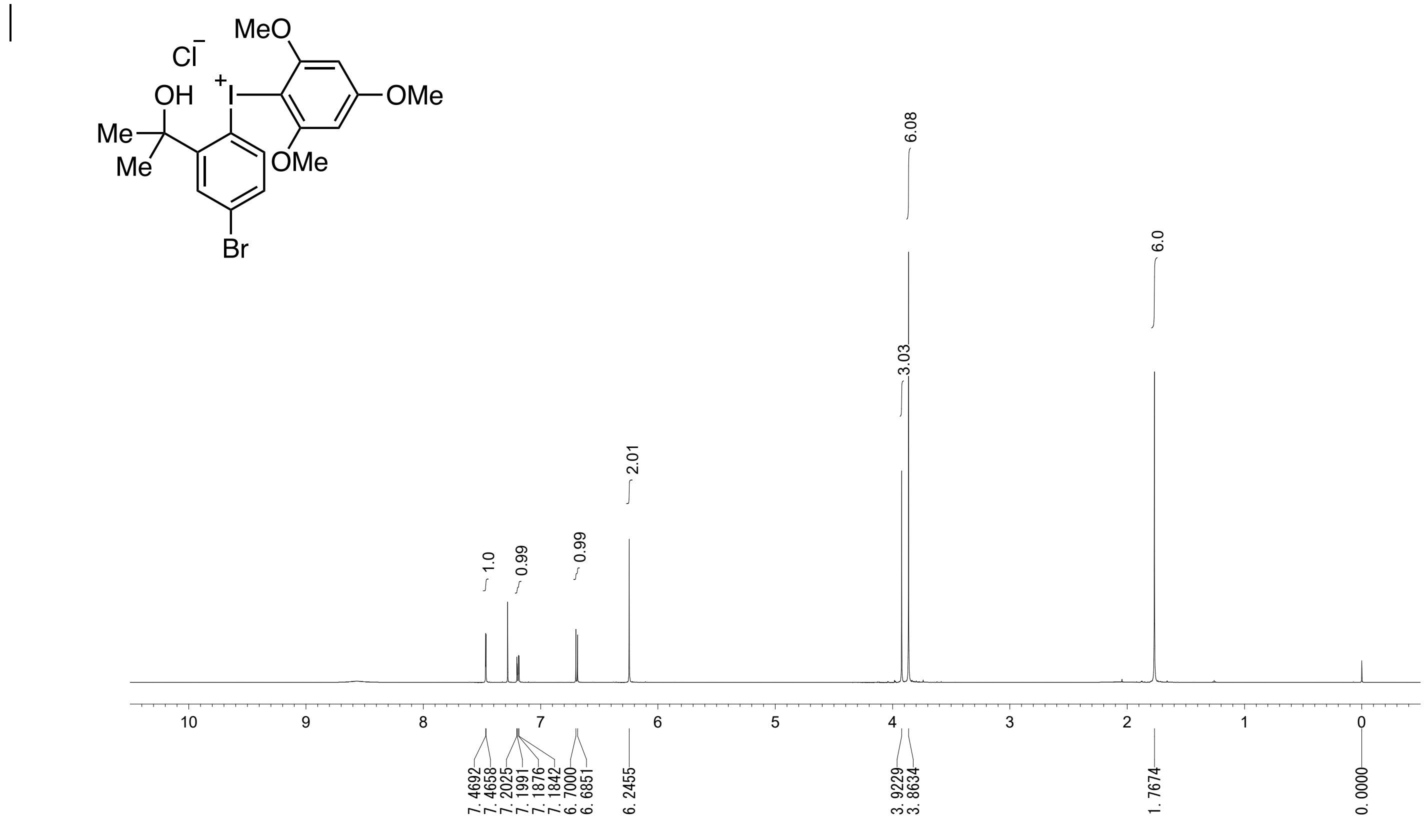

^{13}C{^{1}H} NMR (150 MHz, $CDCl_3$)

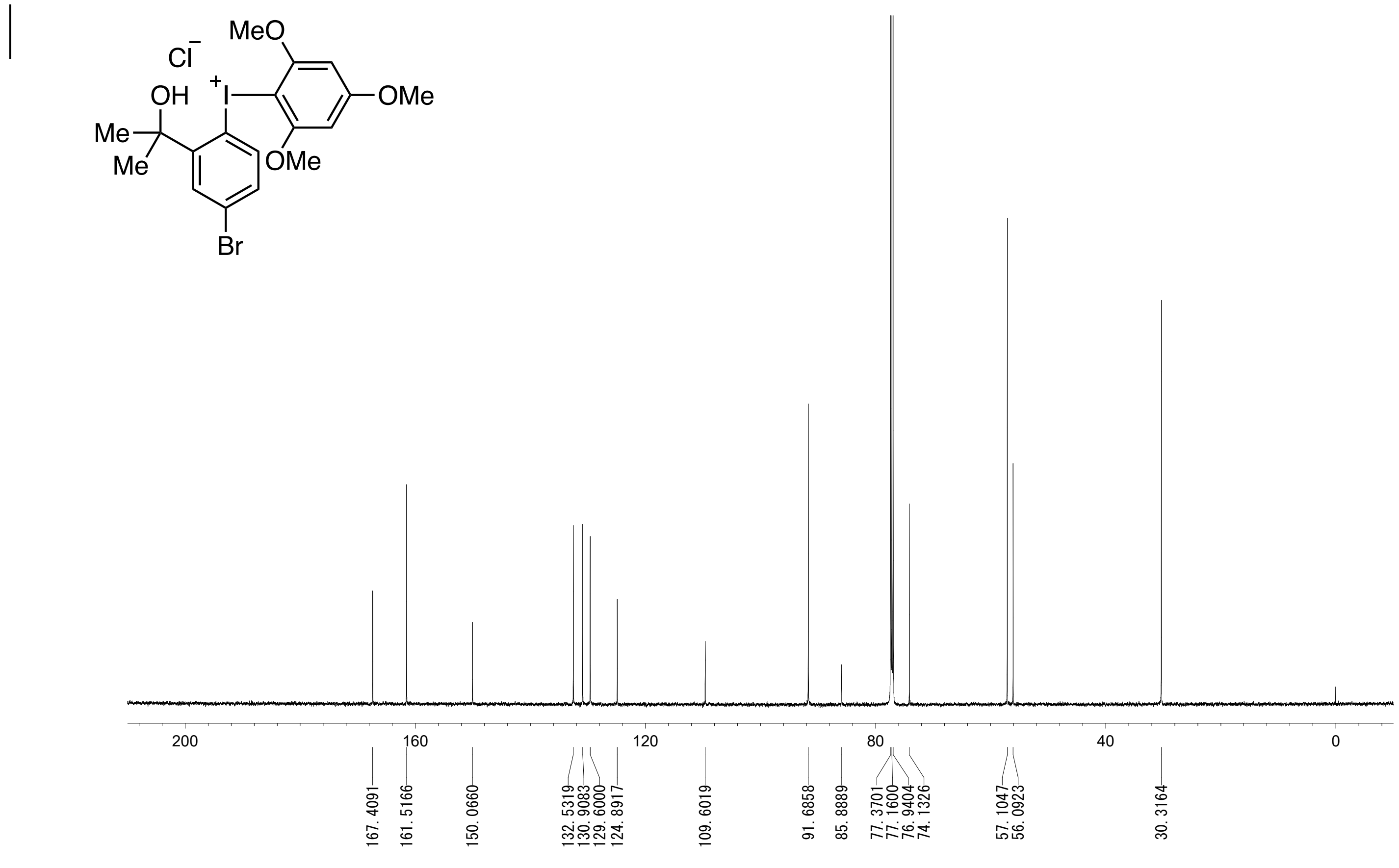

(2-(2-Hydroxypropan-2-yl)-5-methylphenyl)(2,4,6-trimethoxyphenyl)iodonium chloride (2l)

^{1}H NMR (600 MHz, $CDCl_3$)

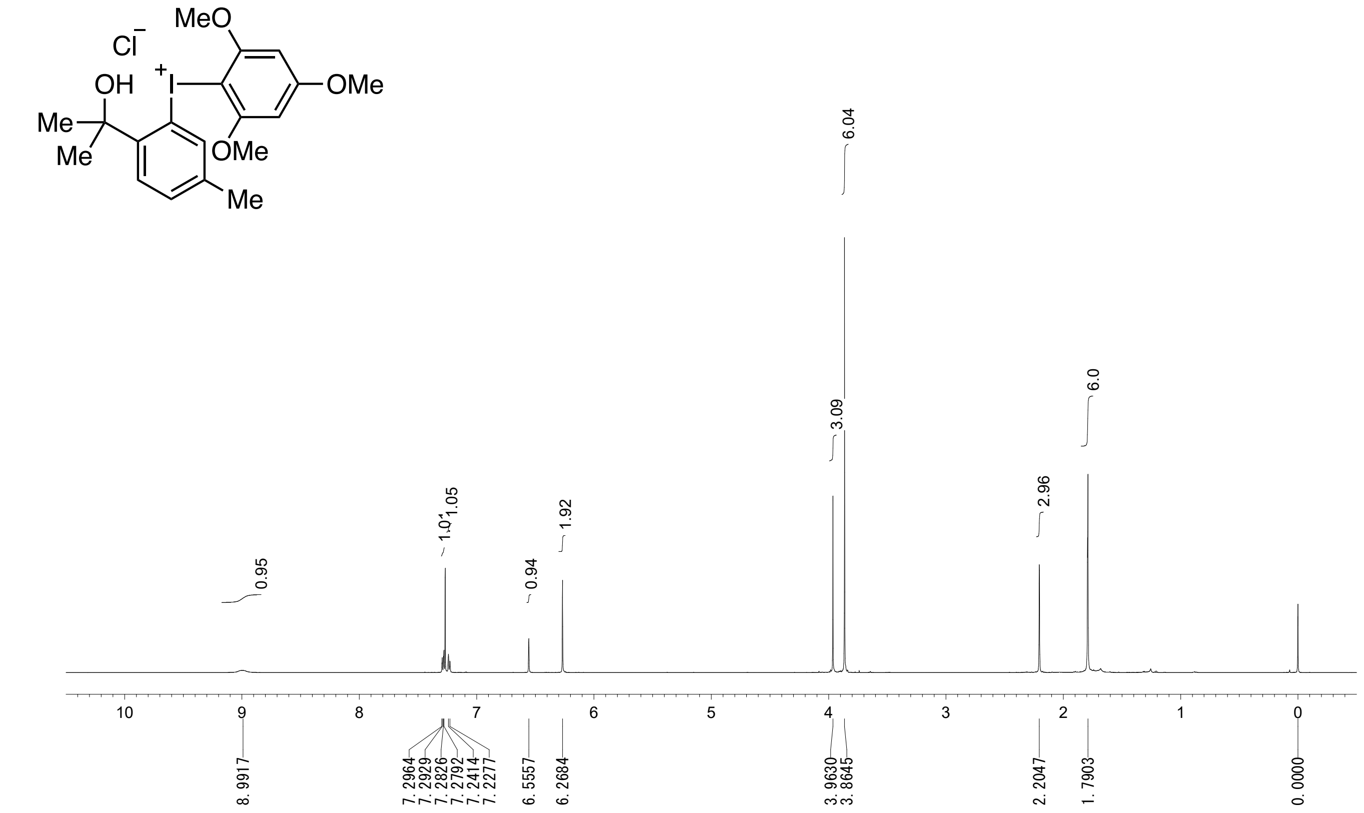

^{13}C{^{1}H} NMR (150 MHz, $CDCl_3$)

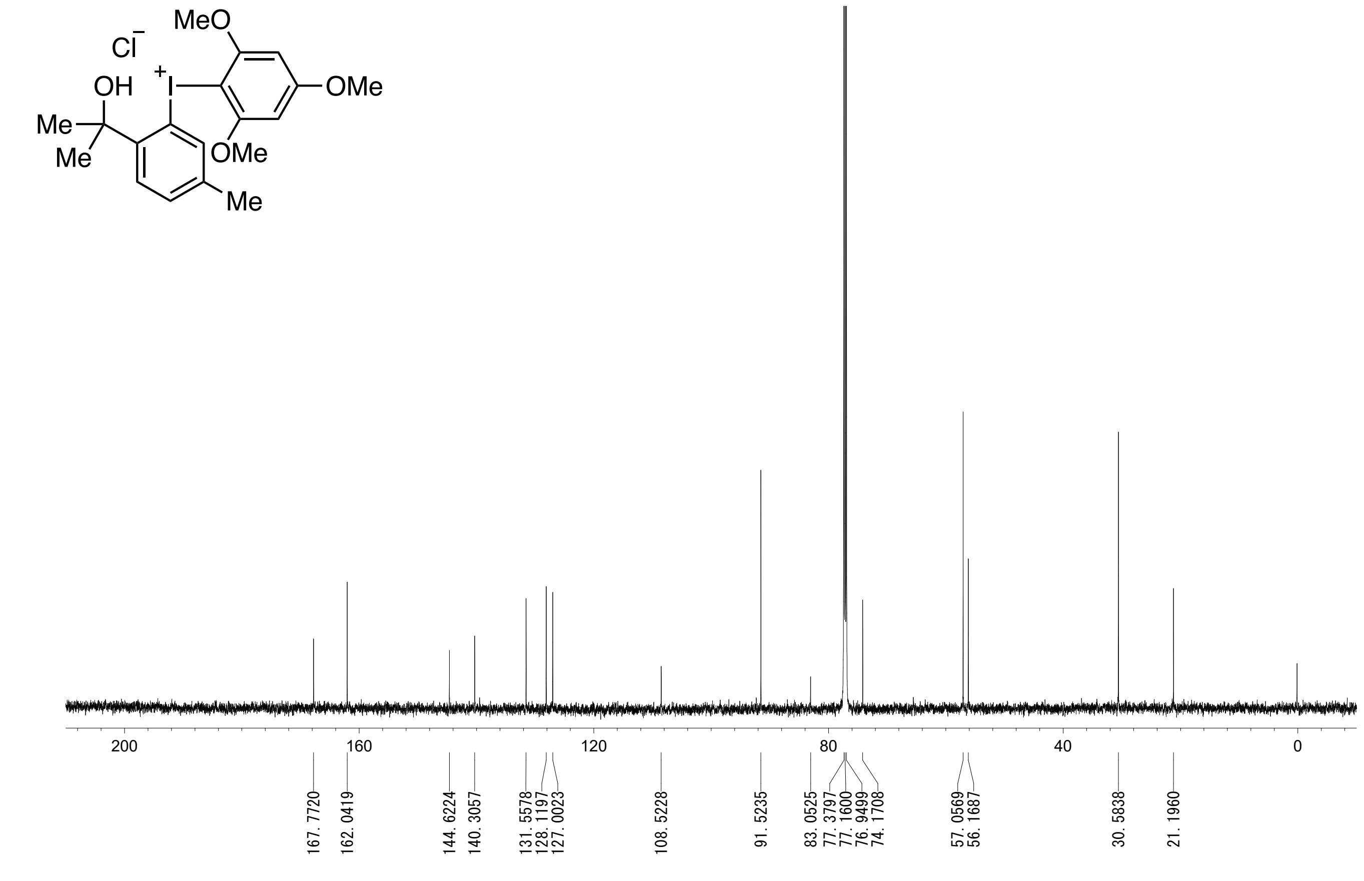

3-Methyl-3-(trifluoromethyl)-1-(2,4,6-trimethoxyphenyl)-1,3-dihydro-1λ³-benzo[*d*][1,2]iodaoxole (2e)
¹H NMR (600 MHz, $CDCl_3$)

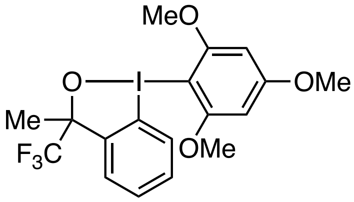

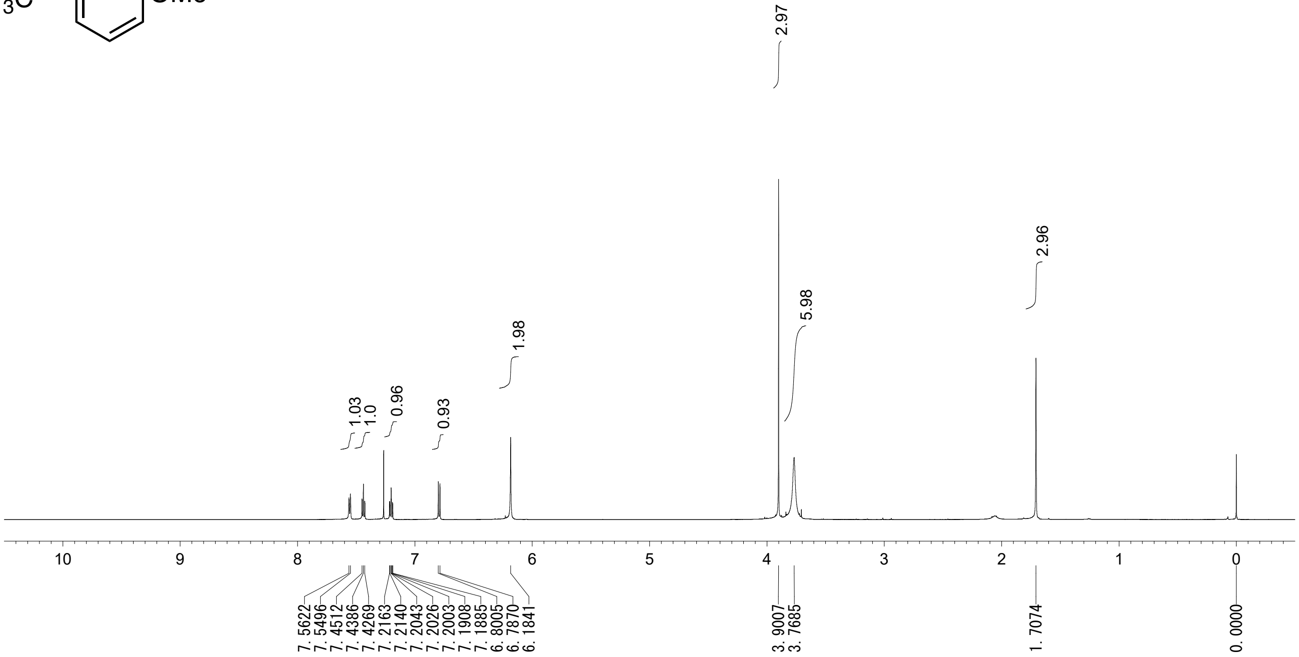

¹³C{¹H} NMR (150 MHz, $CDCl_3$)

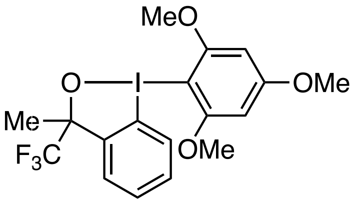

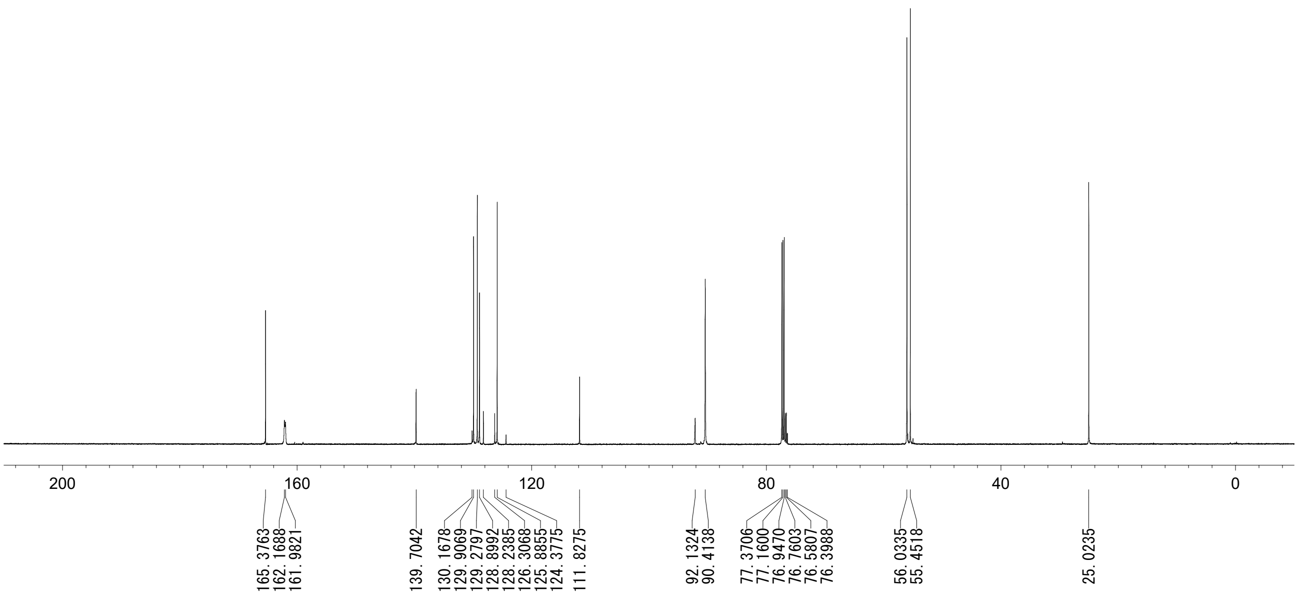

^{19}F NMR (376 MHz, $CDCl_3$)

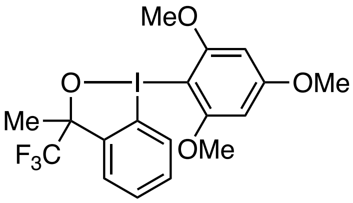

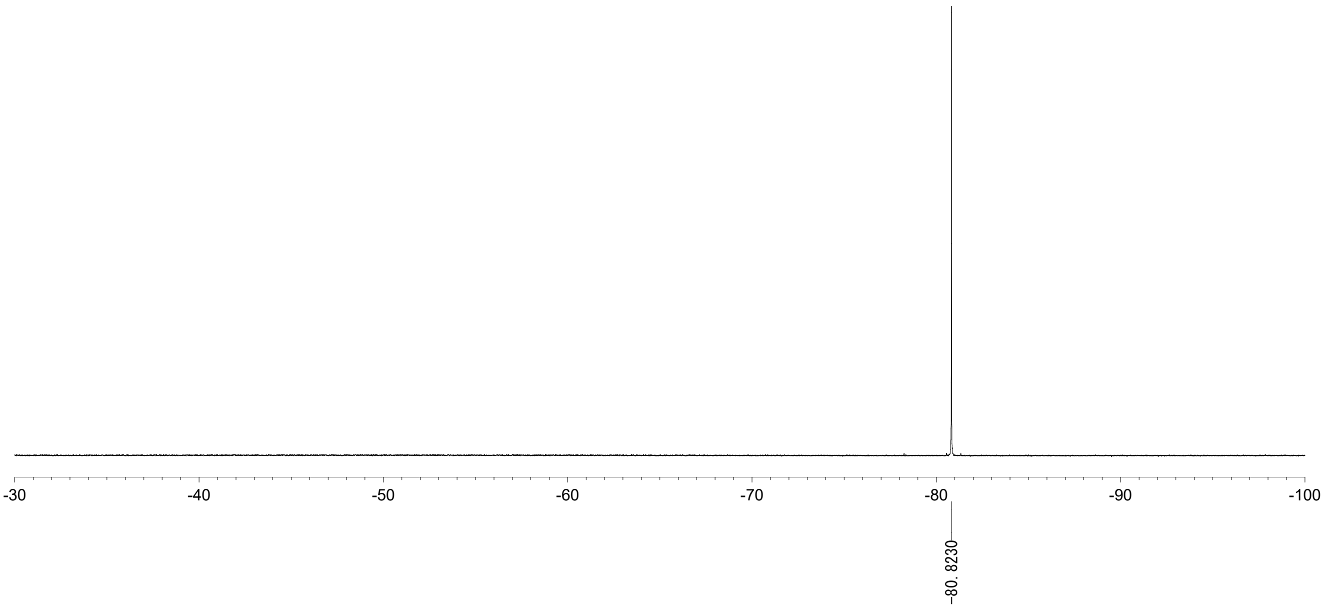

(2-(2-Hydroxypropan-2-yl)phenyl)(2,4,6-trimethoxyphenyl)iodonium 4-methylbenzenesulfonate (2a-OTs)
^{1}H NMR (400 MHz, $CDCl_3$)

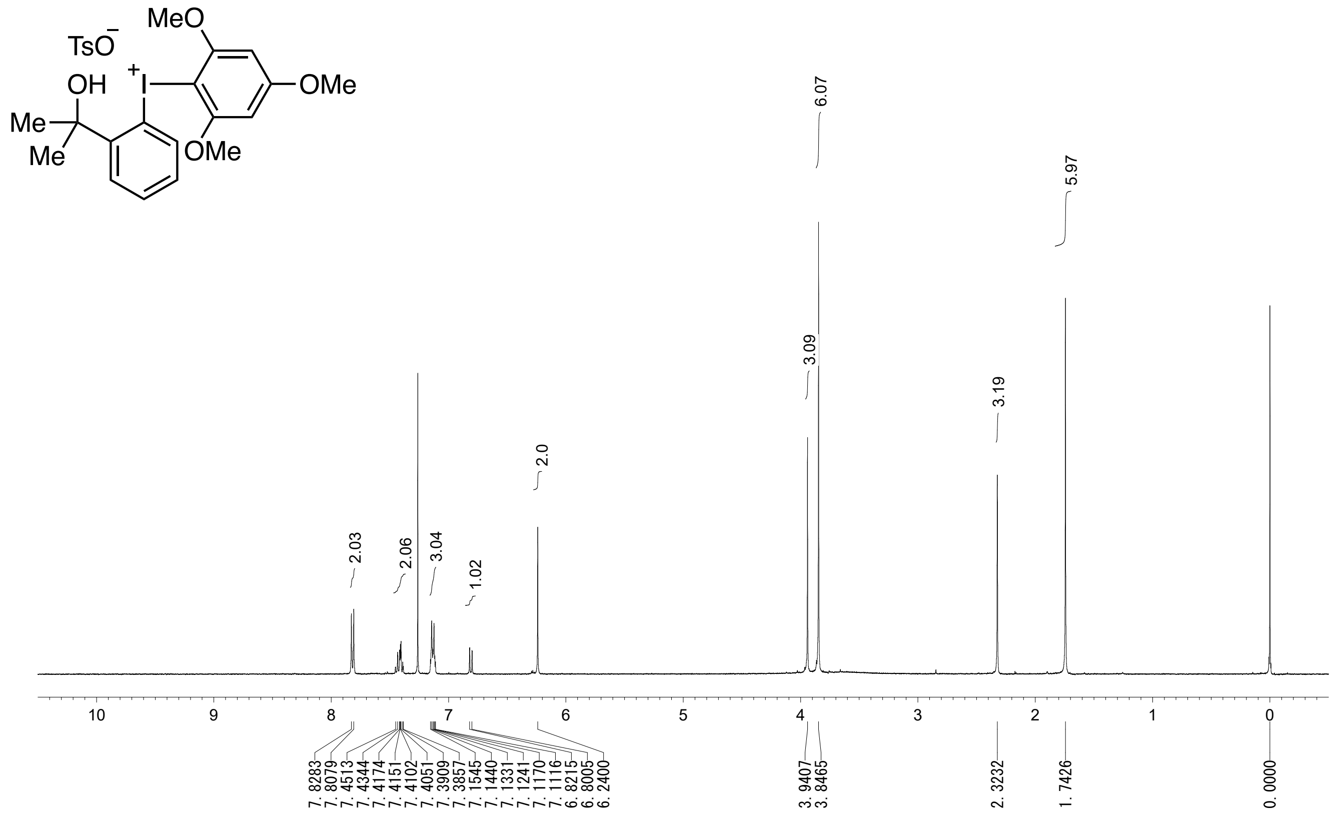

^{13}C{^{1}H} NMR (150 MHz, $CDCl_3$)

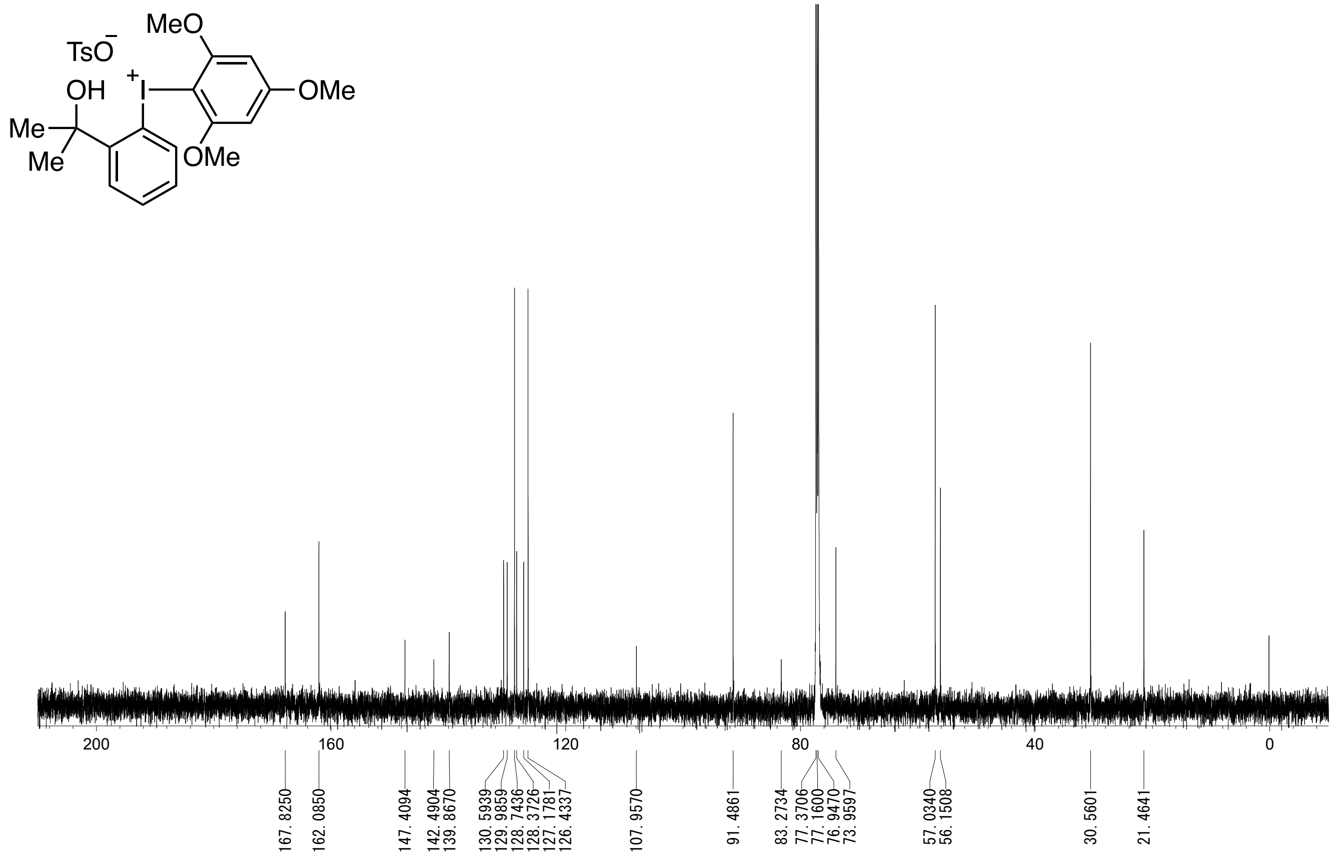

(2-(1-Hydroxyethyl)phenyl)(2,4,6-trimethoxyphenyl)iodonium 4-methylbenzenesulfonate (2i)
^{1}H NMR (400 MHz, $CDCl_3$)

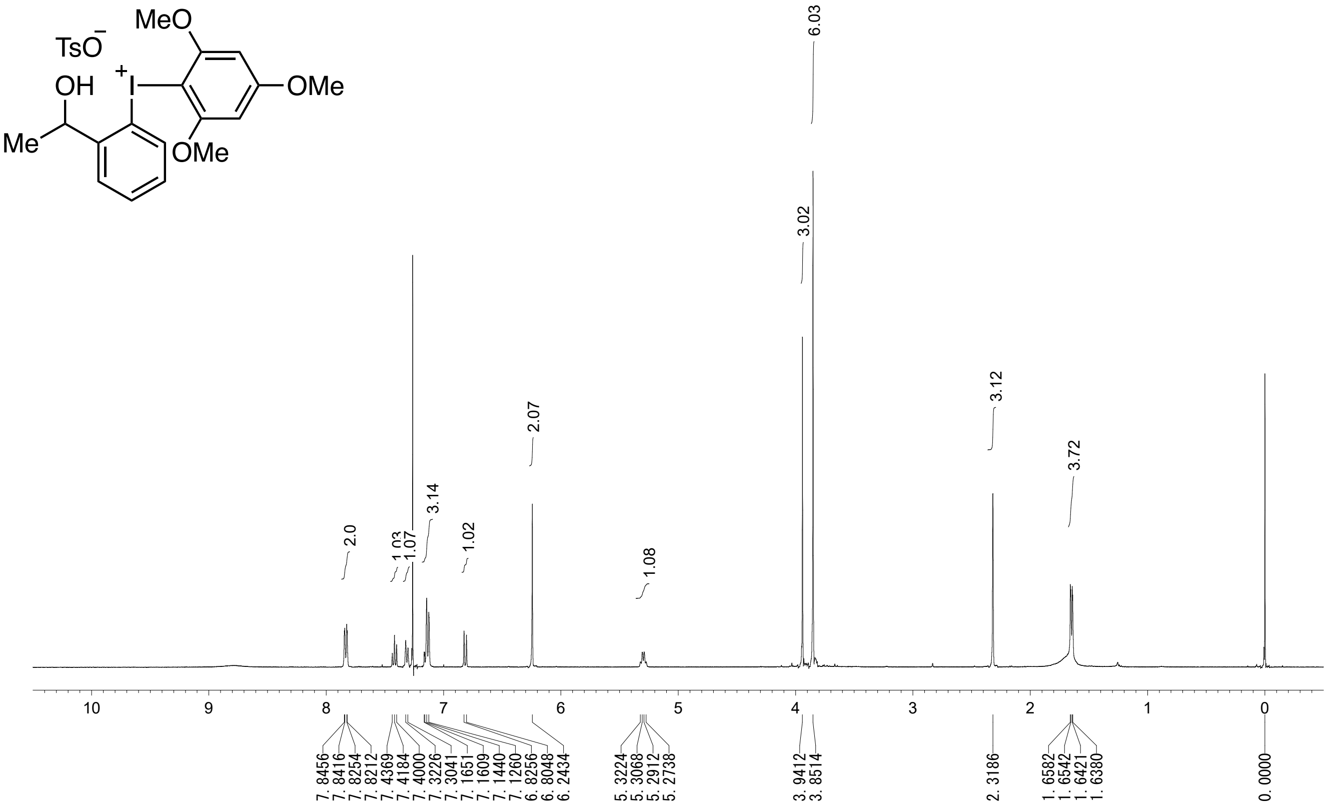

^{13}C{^{1}H} NMR (150 MHz, $CDCl_3$)

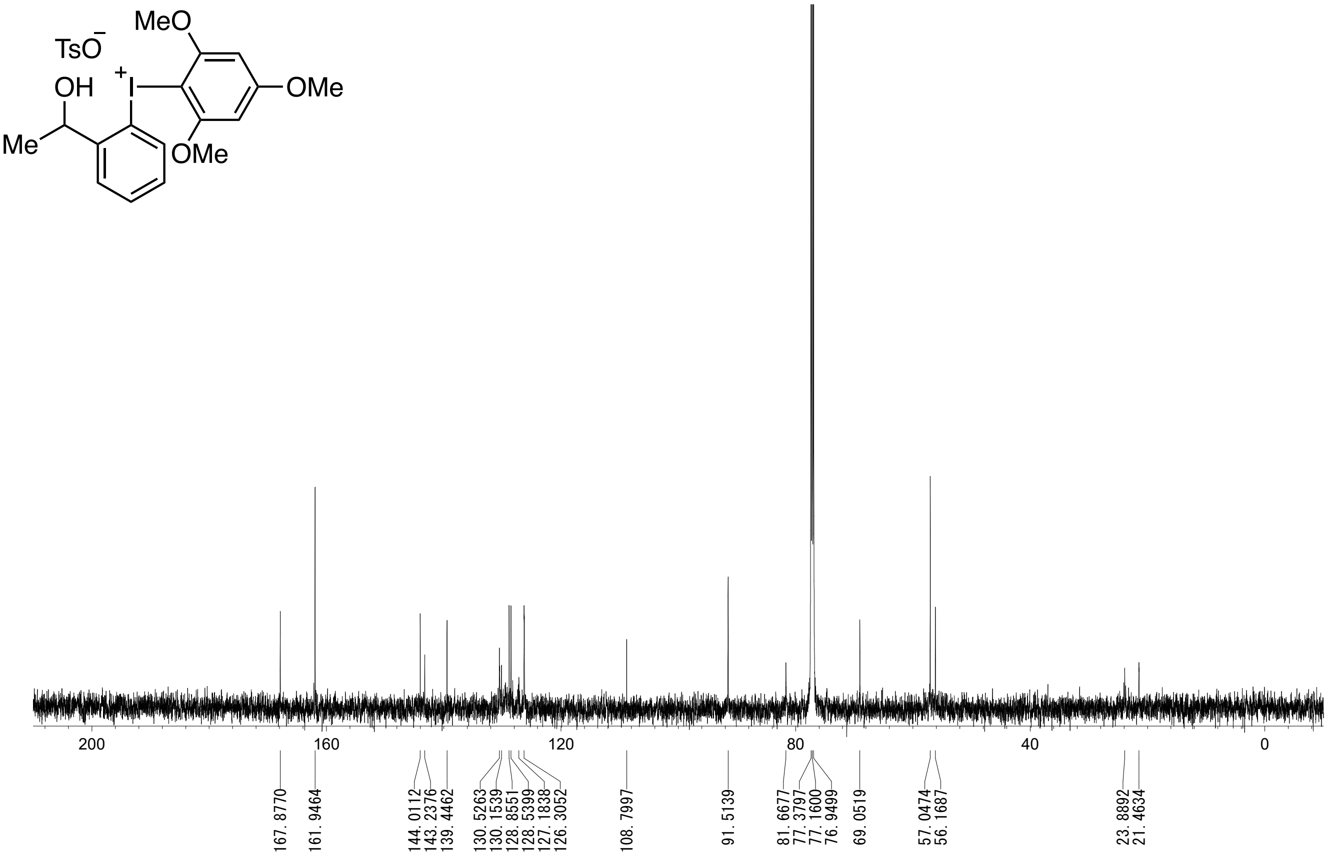

(2-(Hydroxymethyl)phenyl)(2,4,6-trimethoxyphenyl)iodonium 4-methylbenzenesulfonate (2j)
^{1}H NMR (600 MHz, $CDCl_3$)

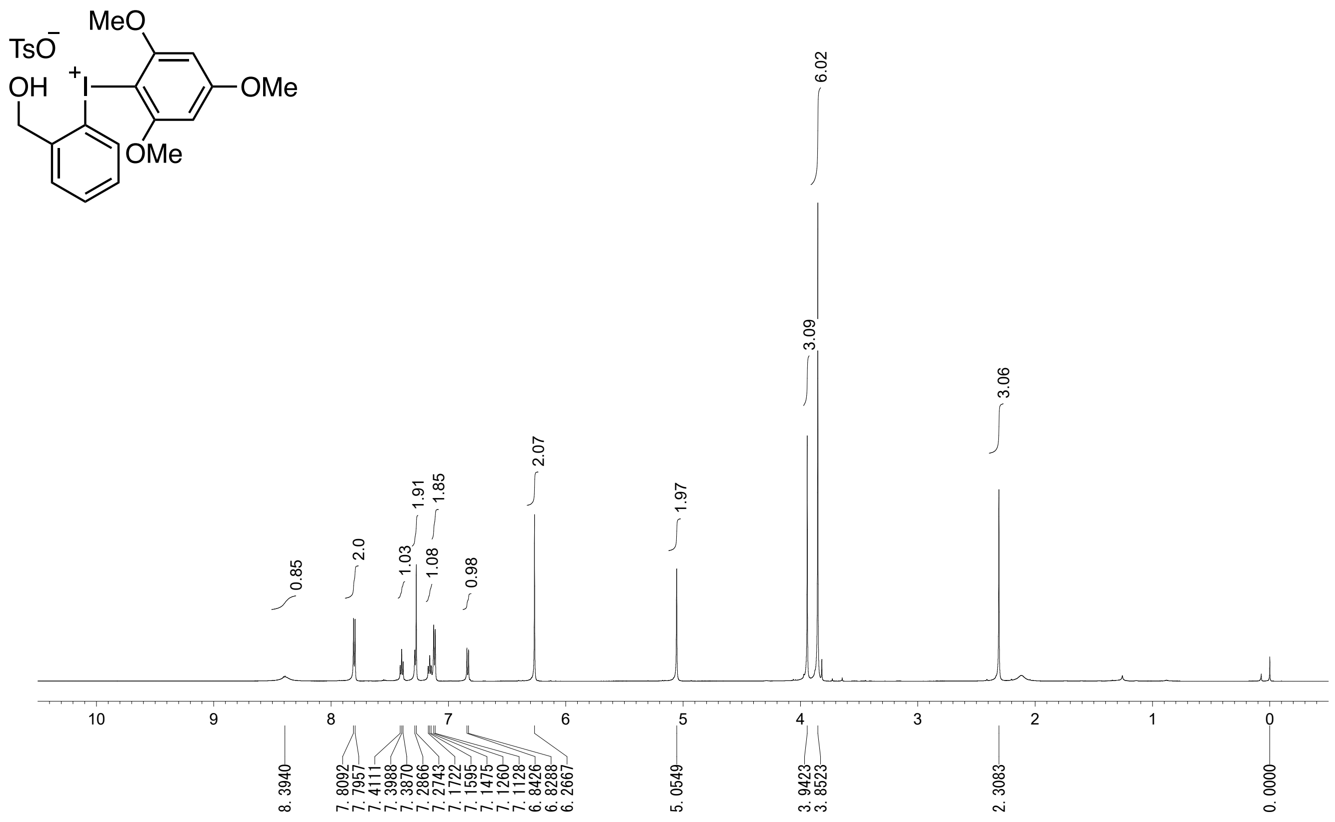

^{13}C{^{1}H} NMR (150 MHz, $CDCl_3$)

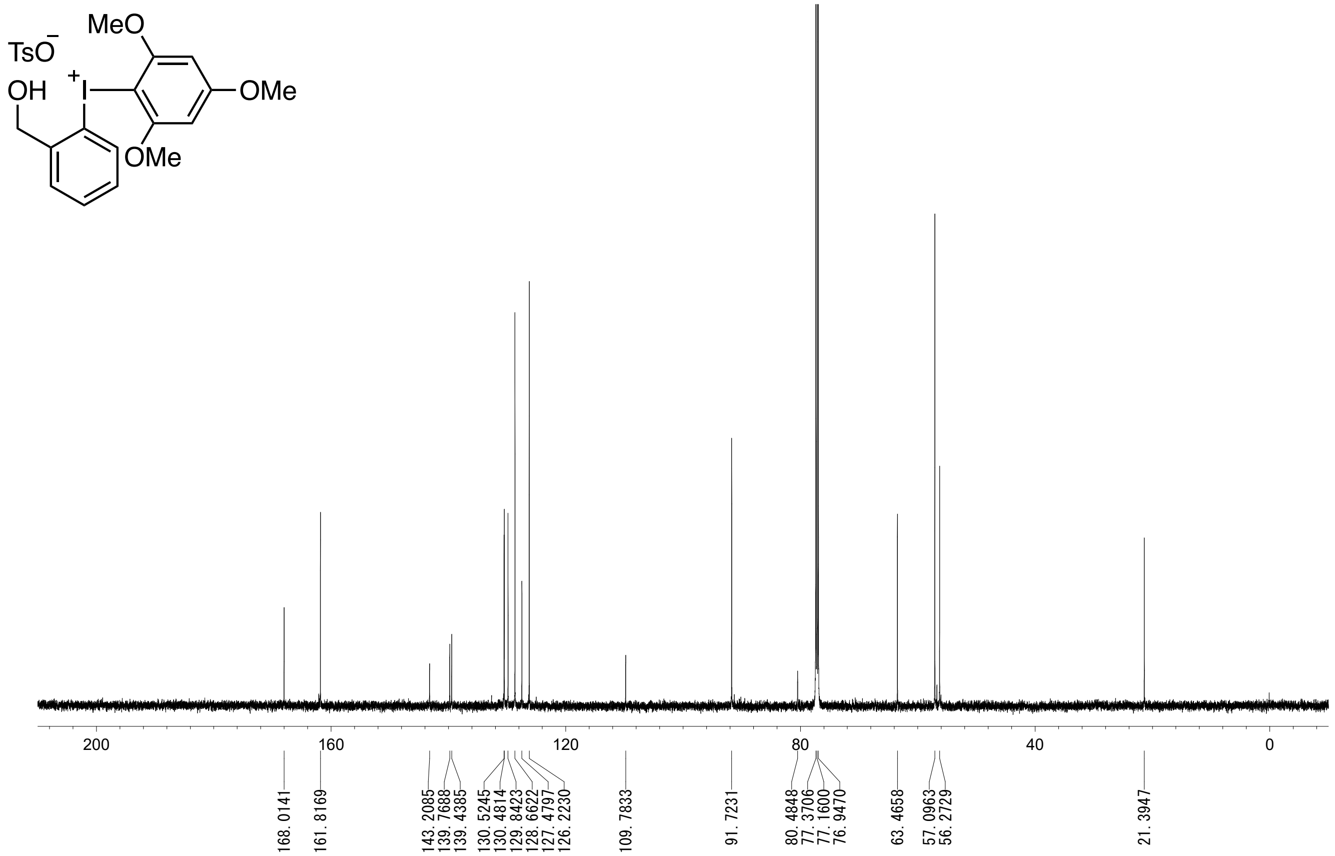

(2-(2-Hydroxyethyl)phenyl)(2,4,6-trimethoxyphenyl)iodonium 4-methylbenzenesulfonate (2k)
^{1}H NMR (400 MHz, $CDCl_3$)

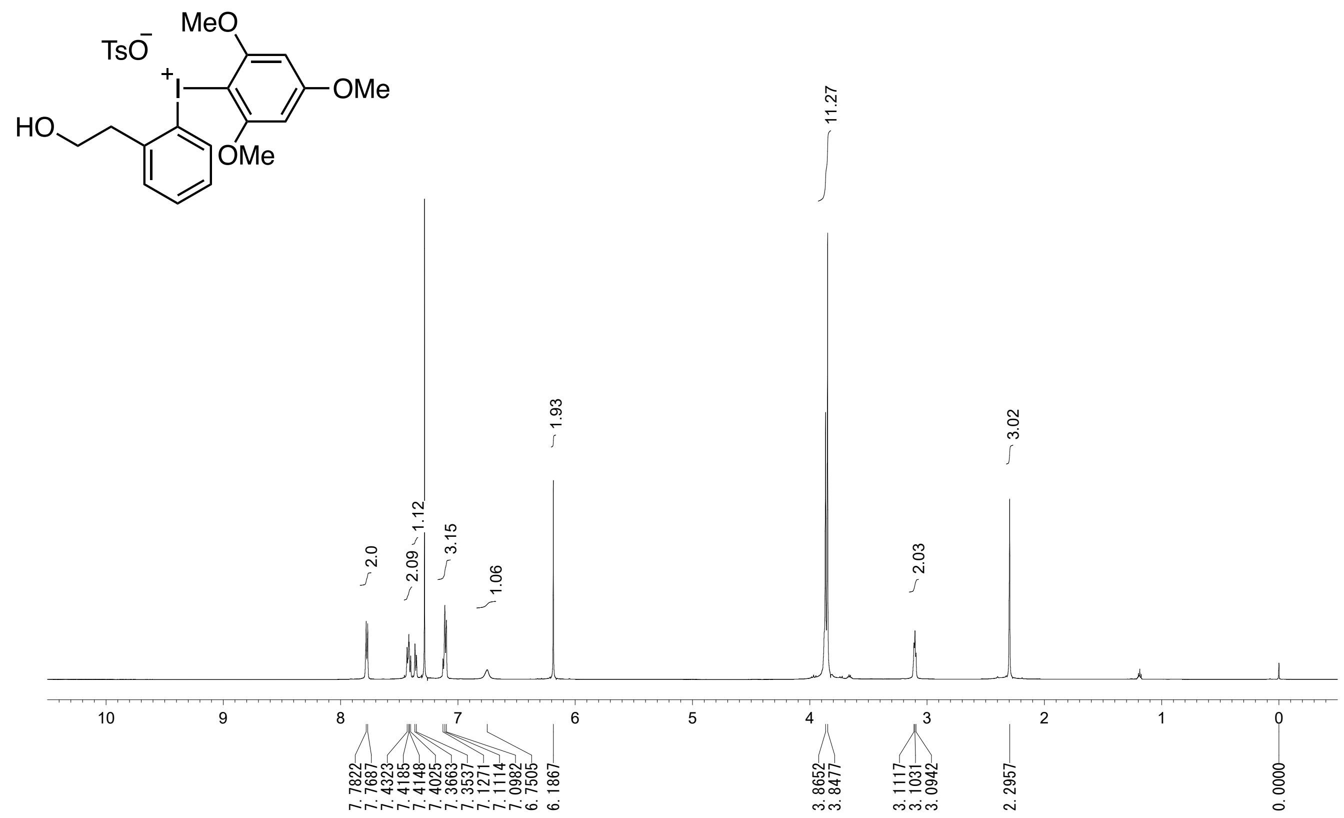

^{13}C{^{1}H} NMR (150 MHz, $CDCl_3$)

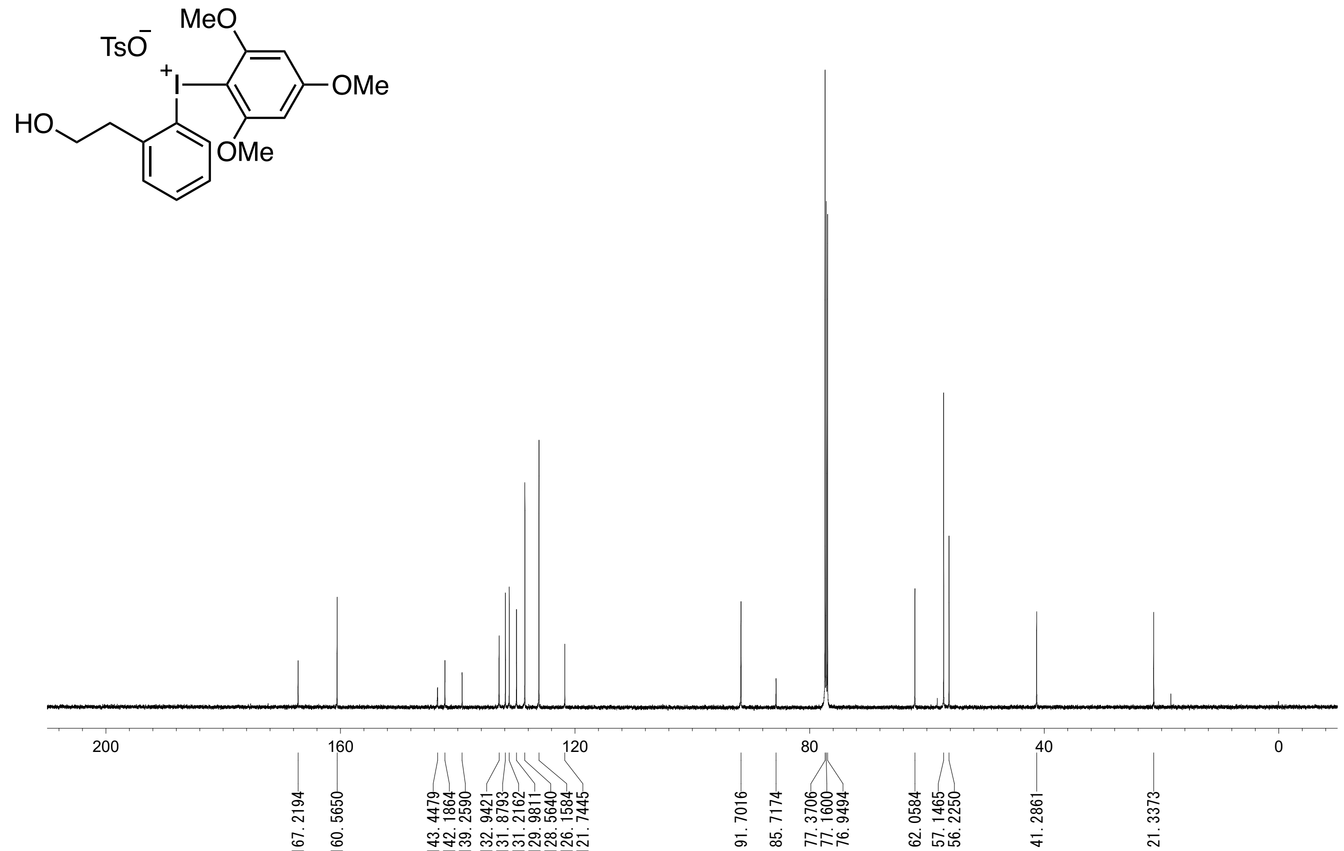

11,11-Dimethyl-11*H*-dibenzo[*b*,*f*][1,4]iodaoxepin-5-ium trifluoromethanesulfonate (3aa)

^{1}H NMR (400 MHz, CD_3OD)

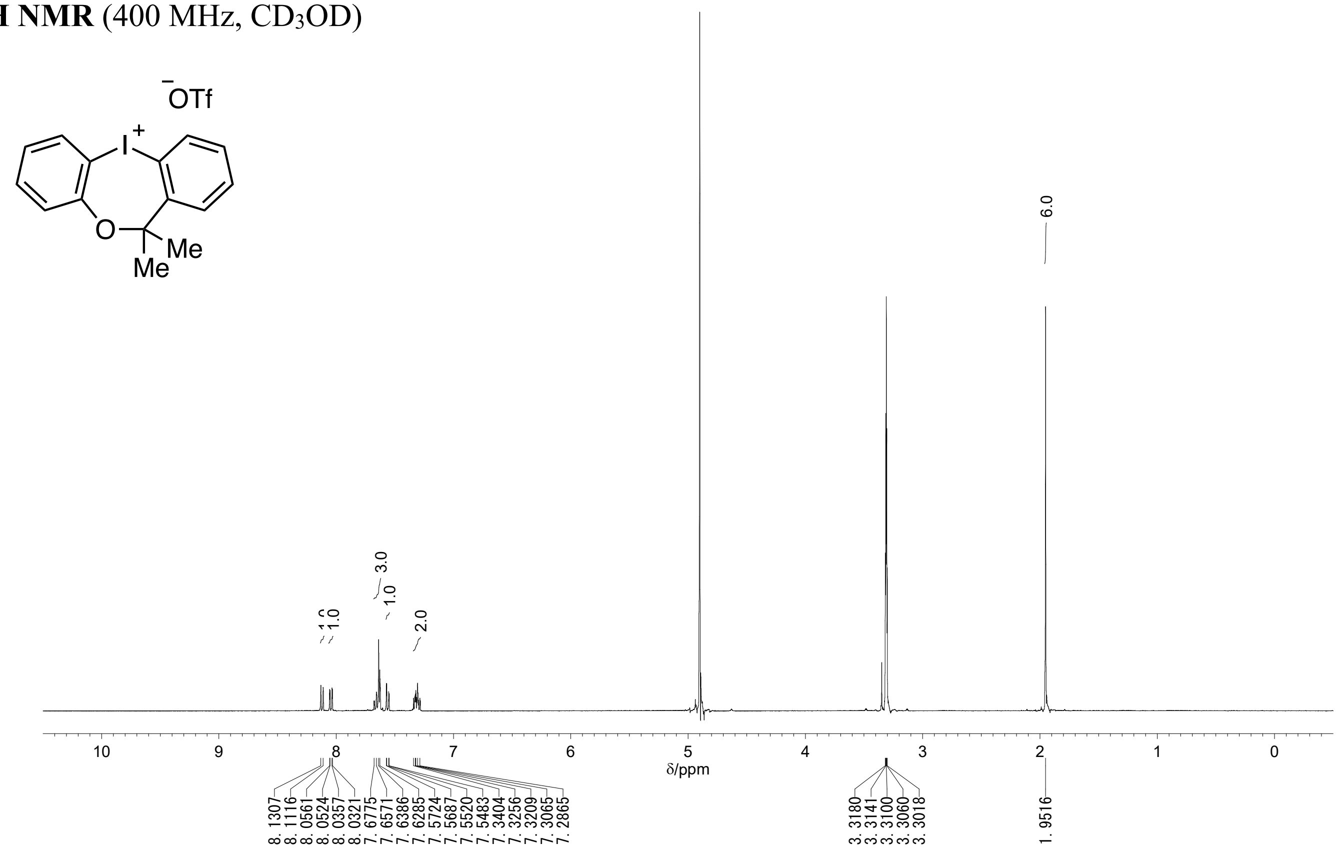

^{13}C{^{1}H} NMR (150 MHz, CD_3OD)

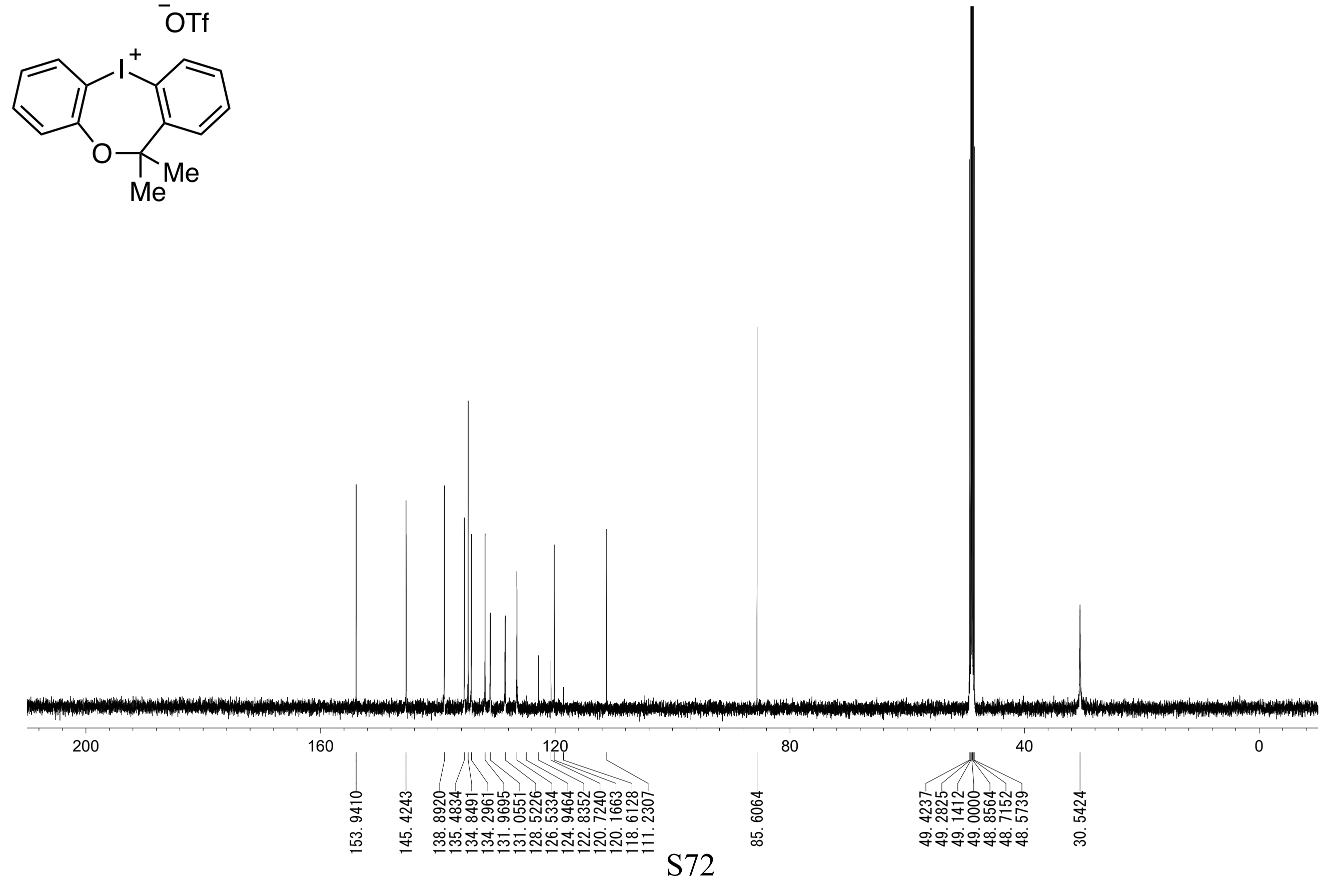

^{19}F NMR (376 MHz, CD_3OD)

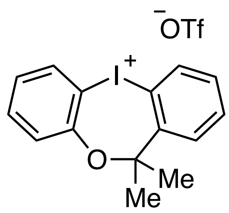

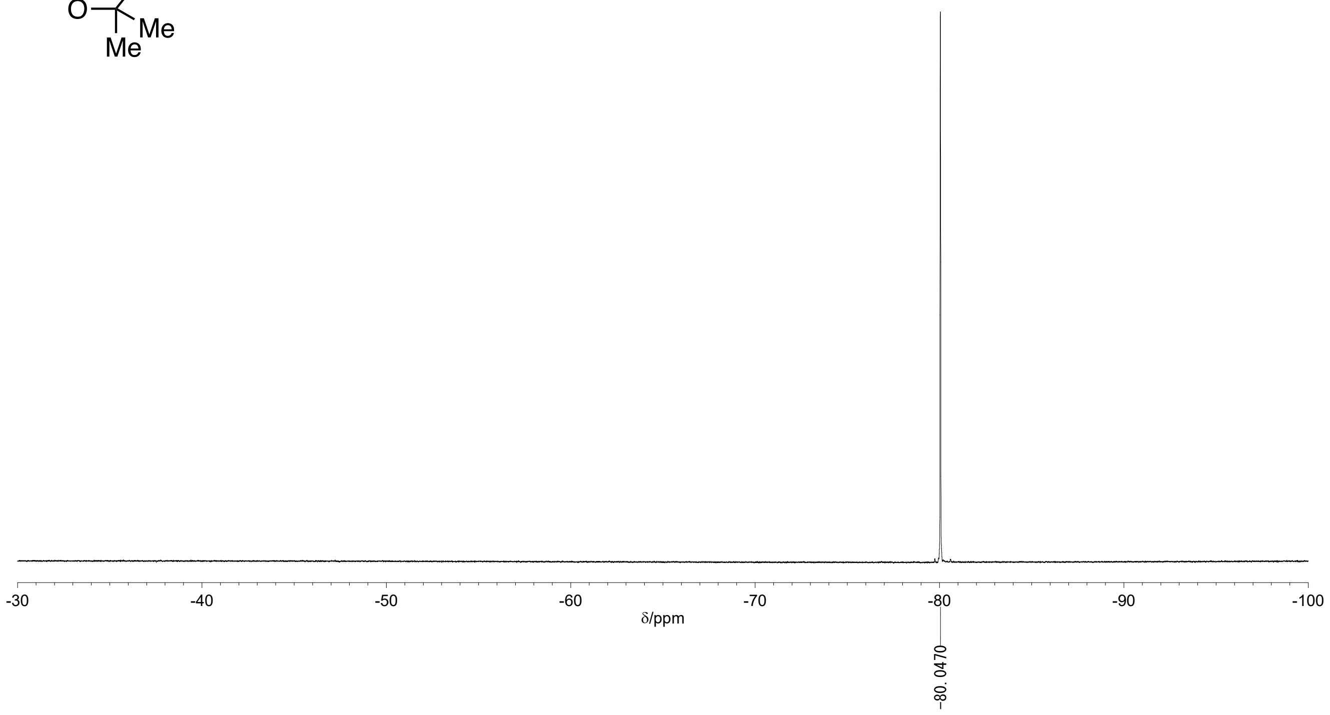

7,8-Dimethoxy-11,11-dimethyl-11*H*-dibenzo[*b*,*f*][1,4]iodaoxepin-5-ium trifluoromethanesulfonate (3ba)
^{1}H NMR (400 MHz, CD_3OD)

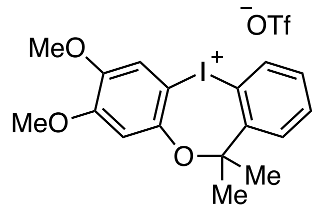

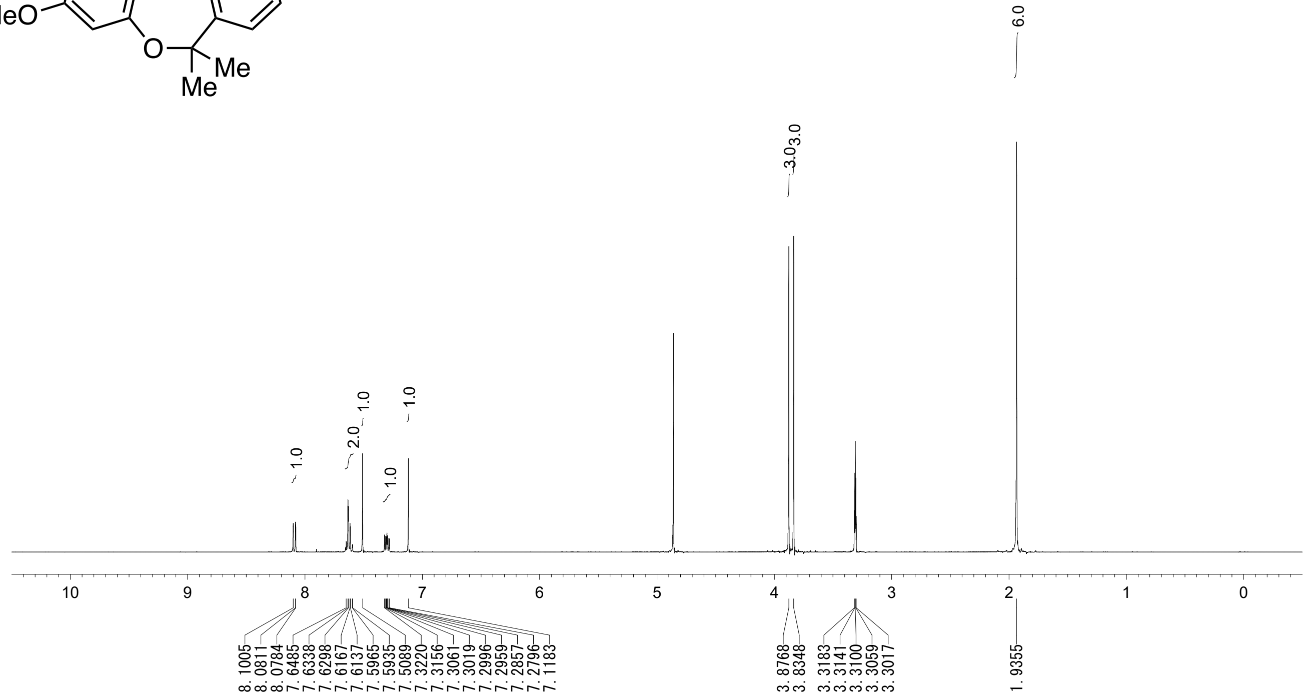

^{13}C{^{1}H} NMR (150 MHz, CD_3OD)

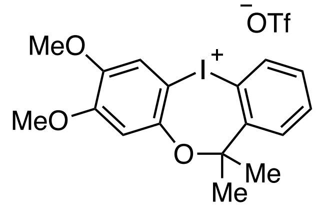

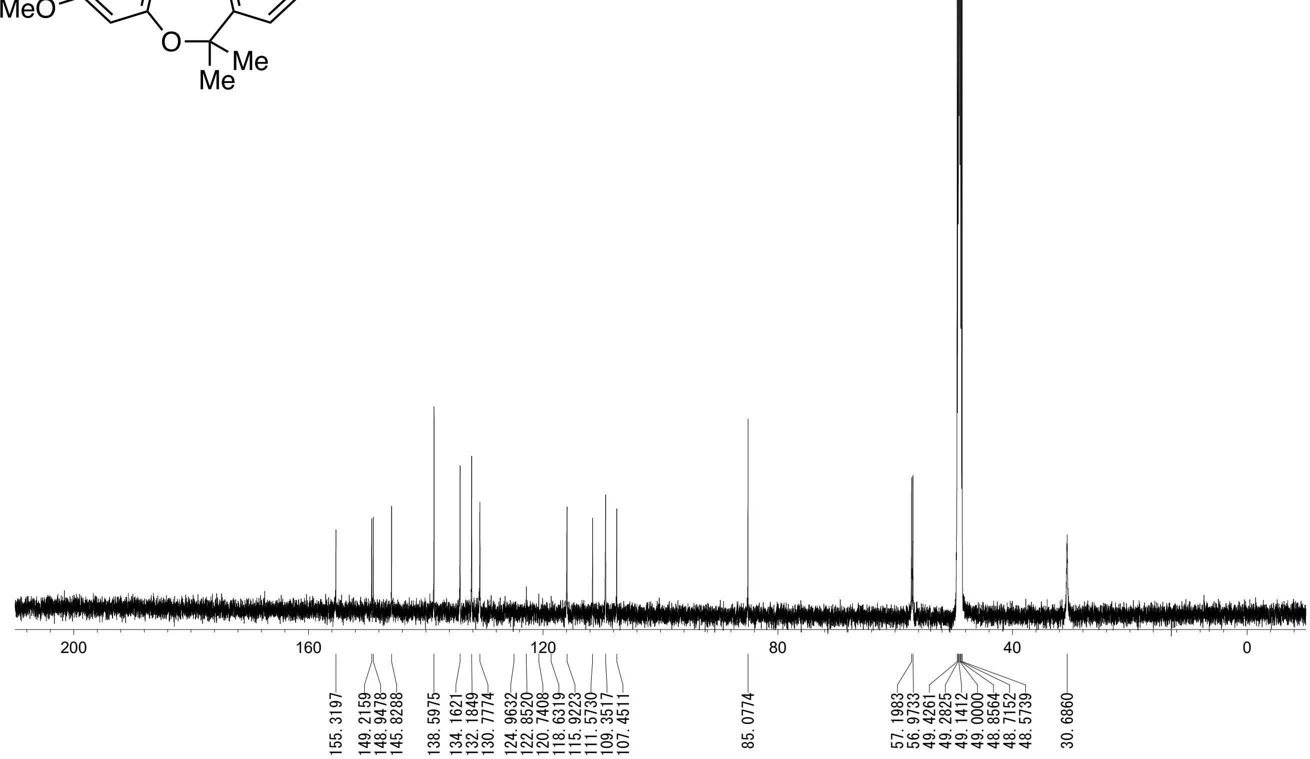

^{19}F NMR (376 MHz, CD_3OD)

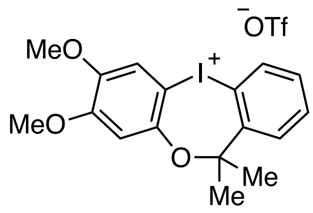

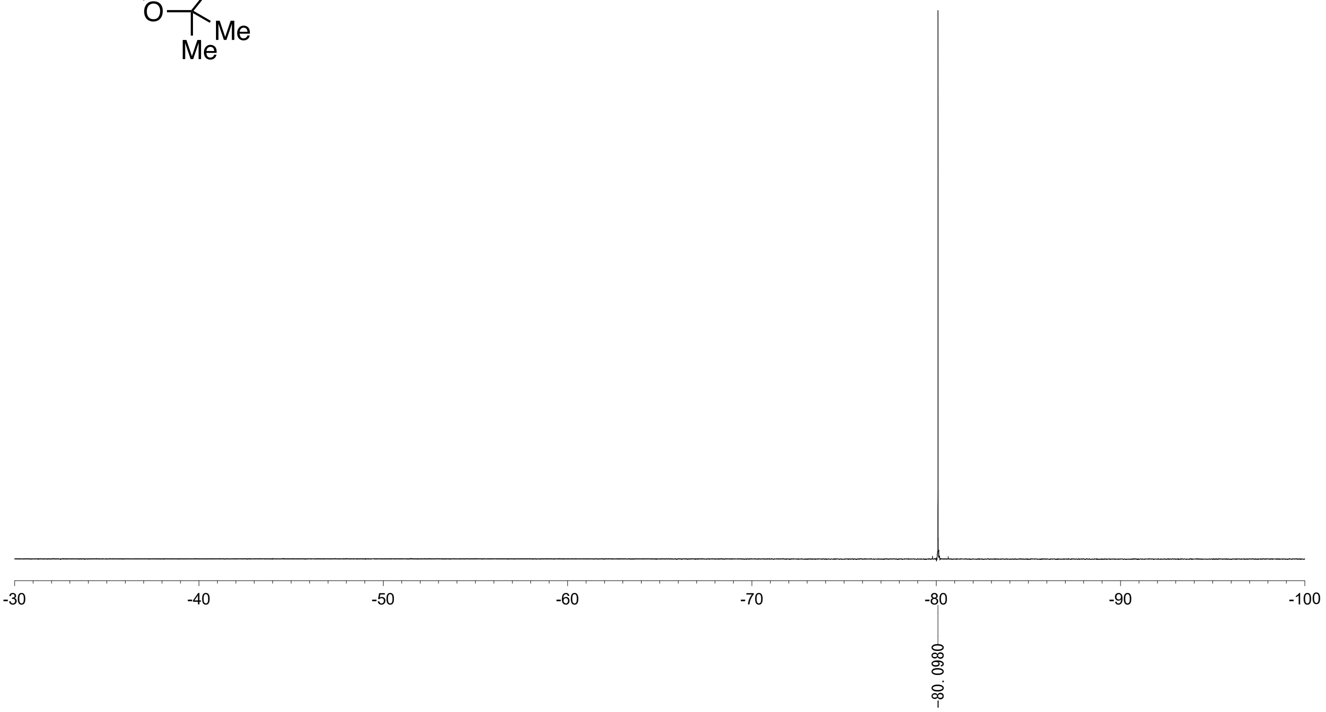

10,10-Dimethyl-10*H*-[1,3]dioxolo[4',5':4,5]benzo[1,2-*b*]benzo[*f*][1,4]iodaoxepin-5-ium trifluoromethanesulfonate (3ca)

^{1}H NMR (400 MHz, CD_3OD)

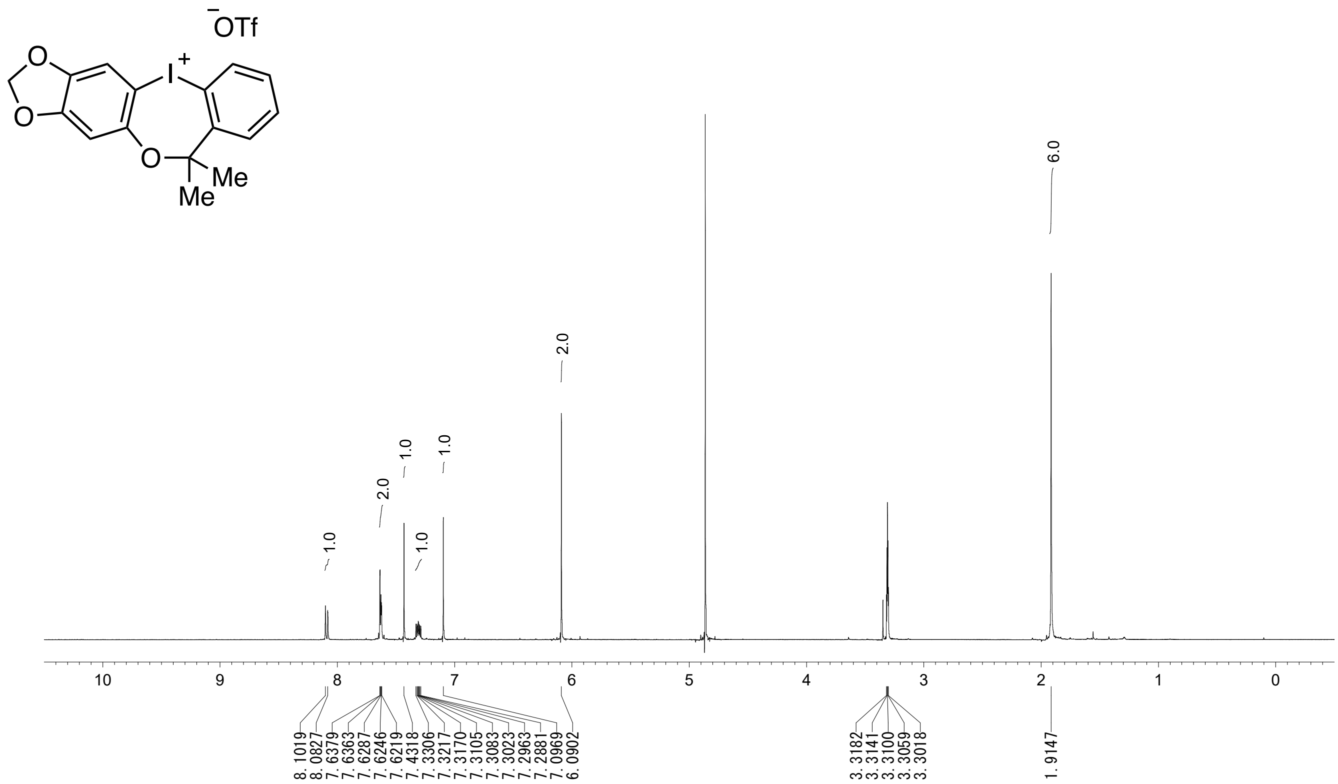

^{13}C{^{1}H} NMR (150 MHz, CD_3OD)

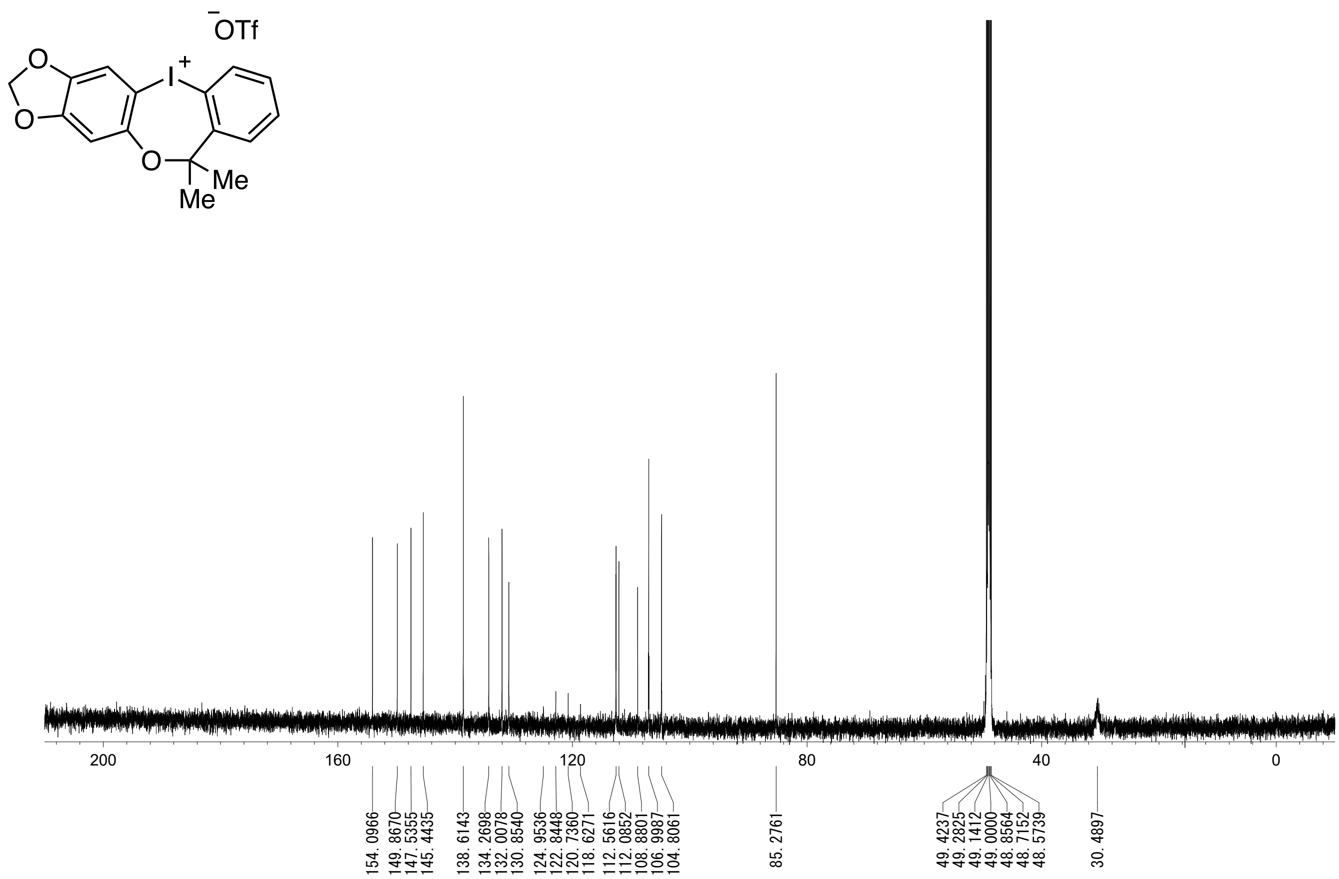

^{19}F NMR (376 MHz, CD_3OD)

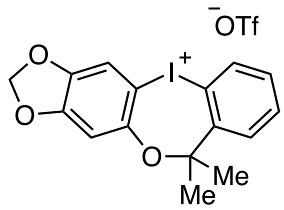

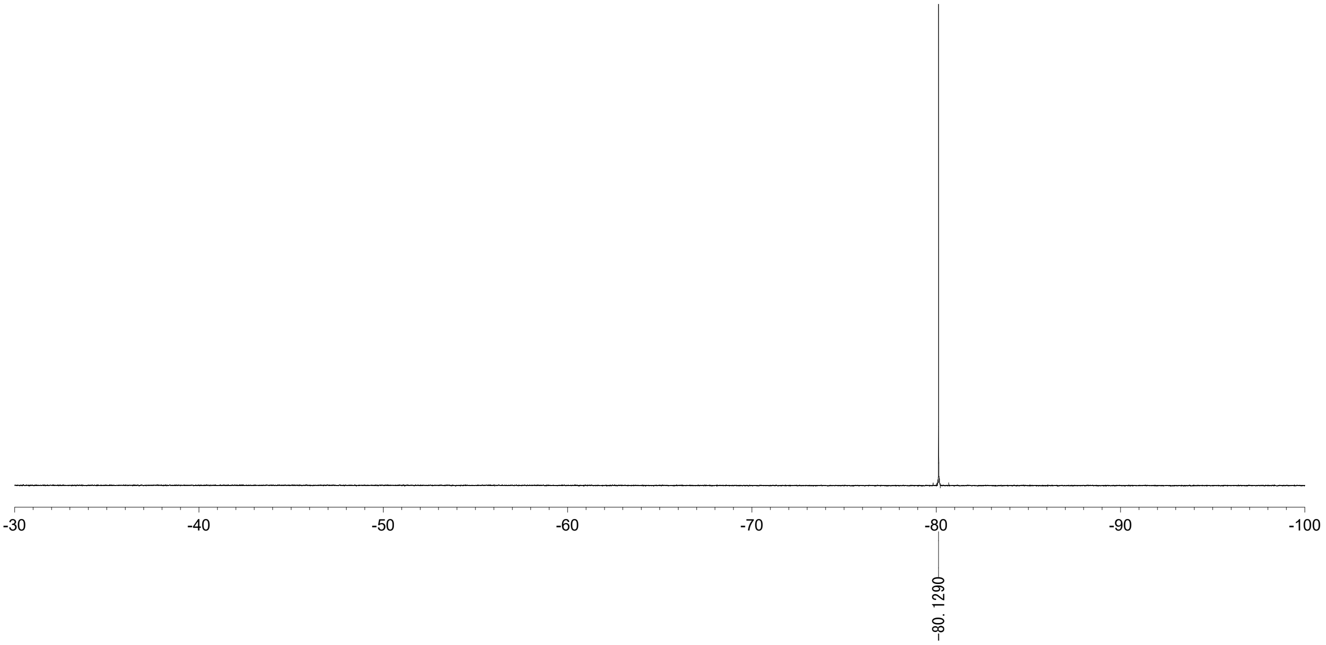

7,8,11,11-Tetramethyl-11*H*-dibenzo[*b*,*f*][1,4]iodaoxepin-5-ium trifluoromethanesulfonate (3da)
^{1}H NMR (600 MHz, CD_3OD)

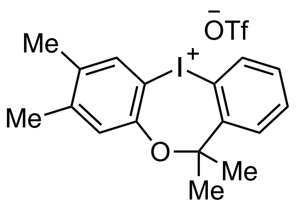

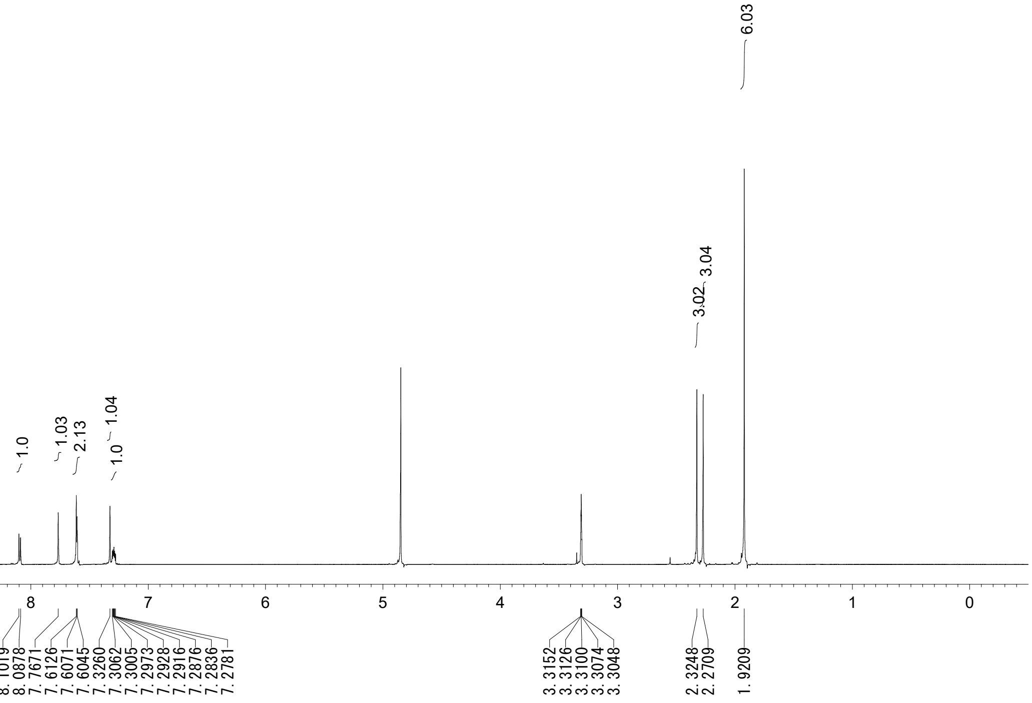

^{13}C{^{1}H} NMR (150 MHz, CD_3OD)

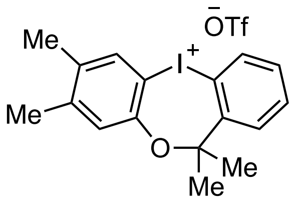

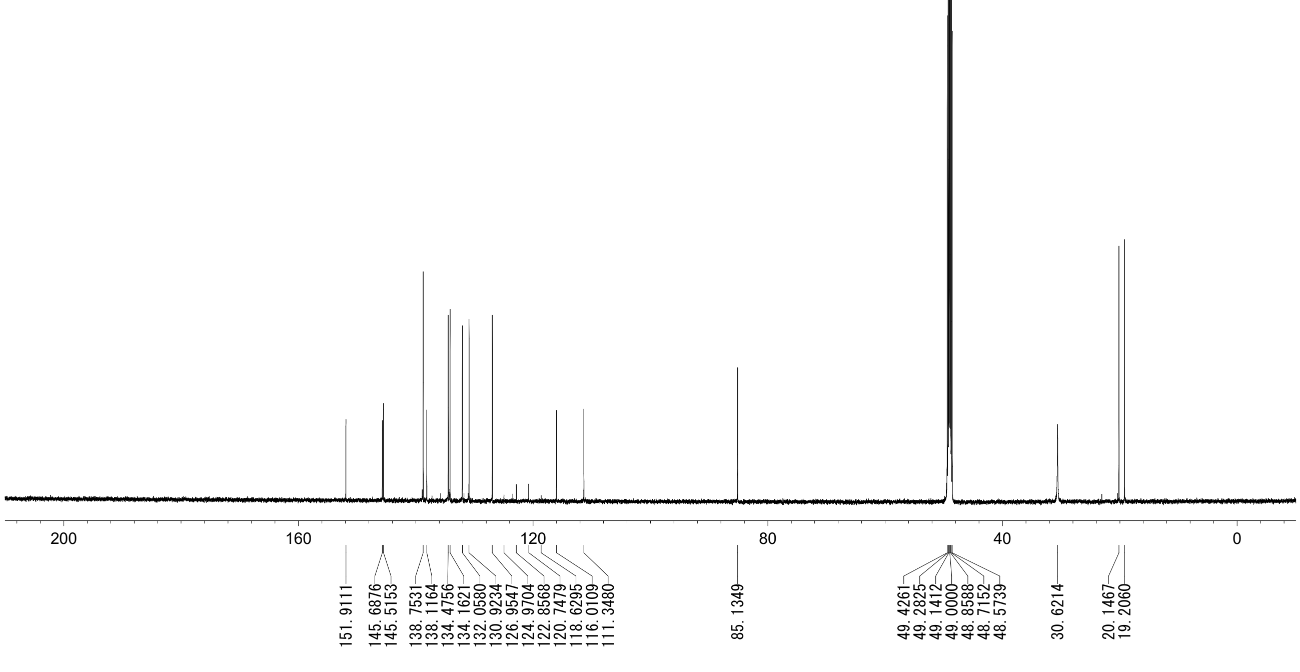

^{19}F NMR (376 MHz, CD_3OD)

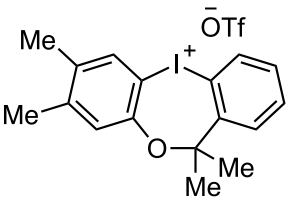

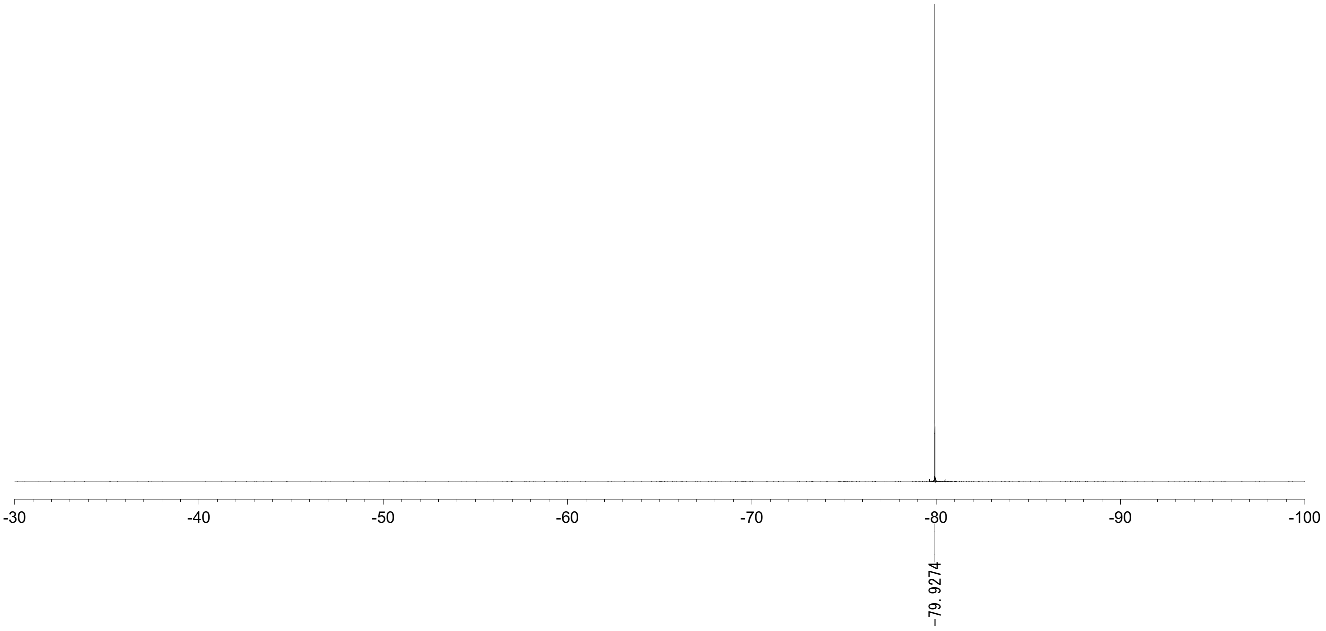

6,9,11,11-Tetramethyl-11*H*-dibenzo[*b*,*f*][1,4]iodaoxepin-5-ium trifluoromethanesulfonate (3ea)
^{1}H NMR (400 MHz, CD_3OD)

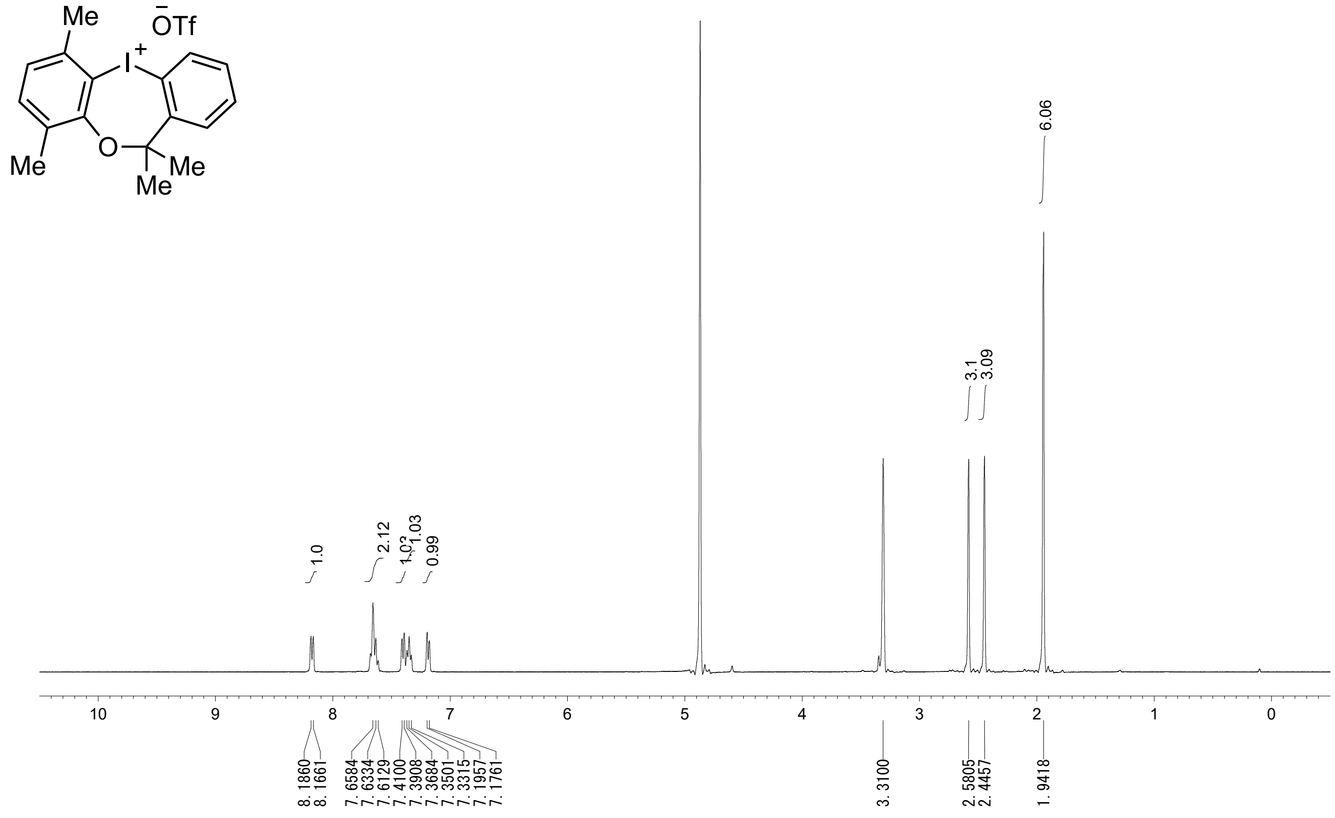

^{13}C{^{1}H} NMR (150 MHz, CD_3OD)

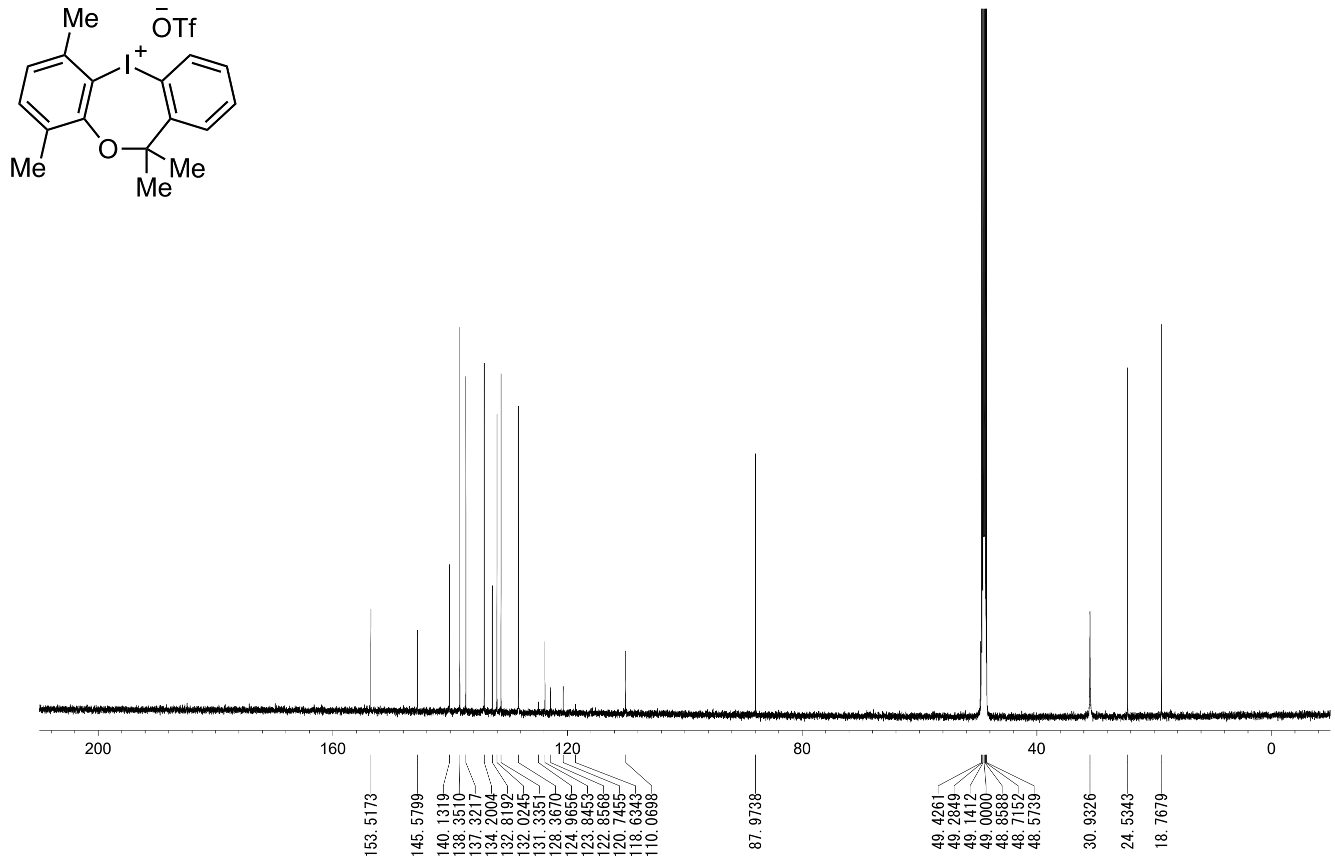

^{19}F NMR (376 MHz, CD_3OD)

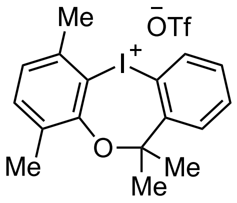

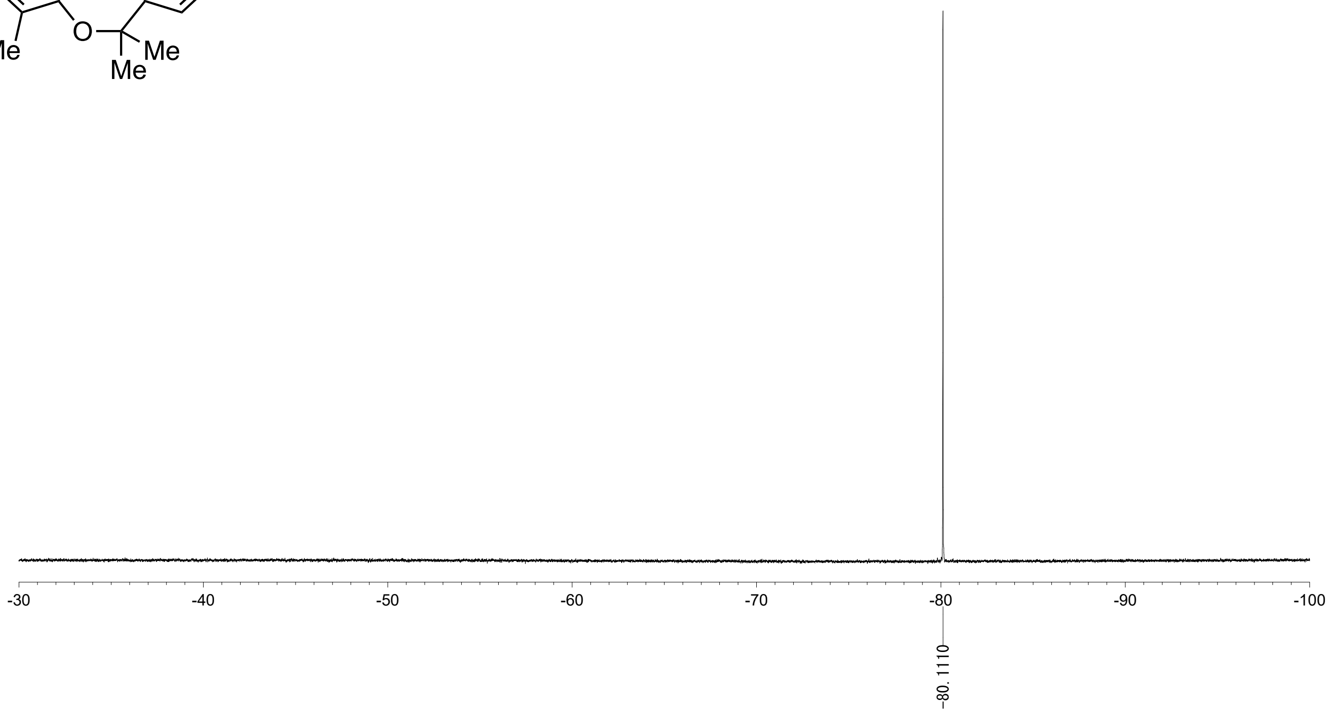

6-Methoxy-11,11-dimethyl-11*H*-dibenzo[b,*f*][1,4]iodaoxepin-5-ium trifluoromethanesulfonate (3fa)
^{1}H NMR (400 MHz, CD_3OD)

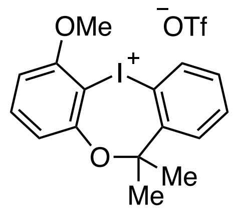

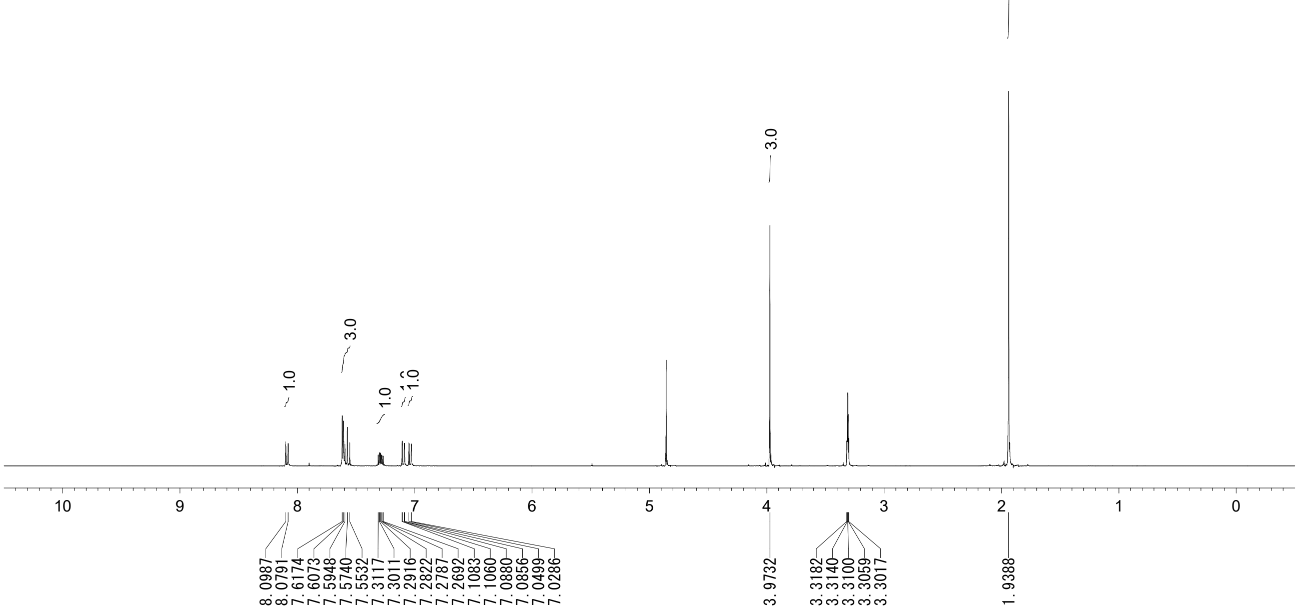

^{13}C{^{1}H} NMR (150 MHz, CD_3OD)

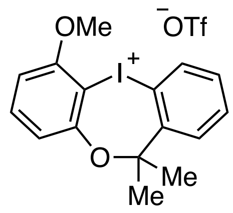

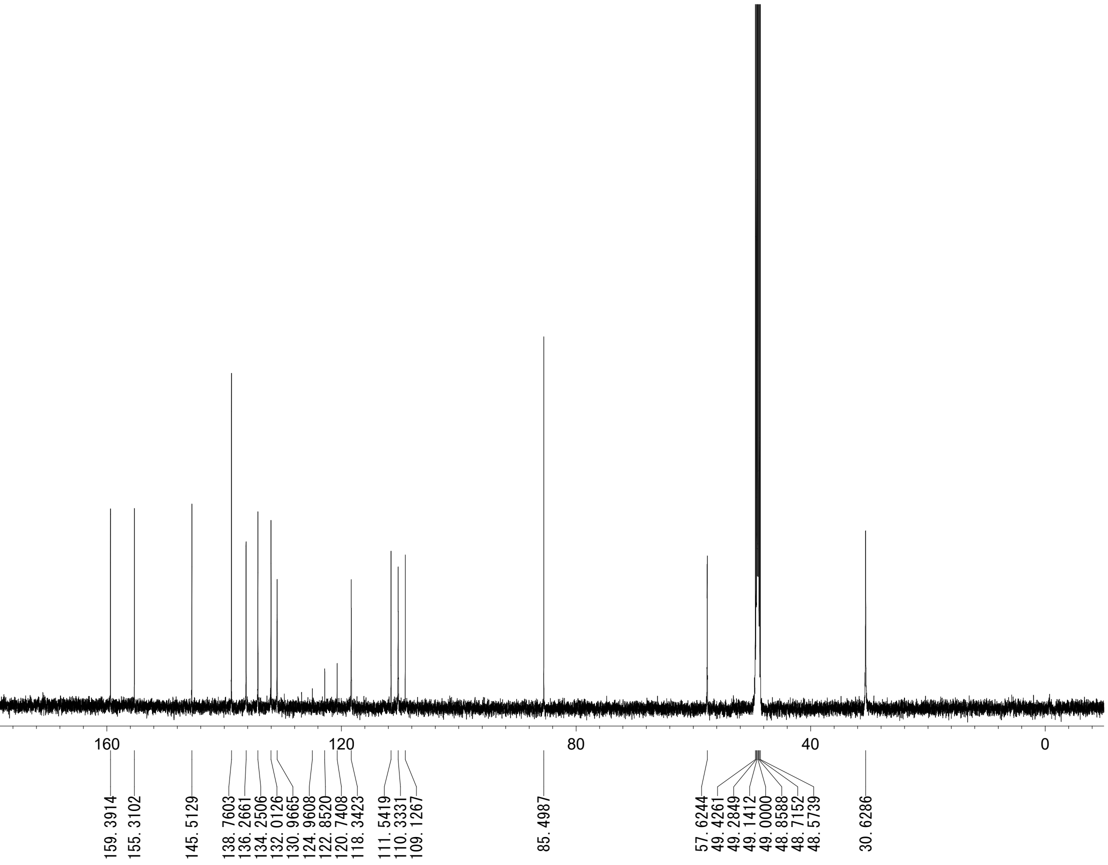

^{19}F NMR (376 MHz, CD_3OD)

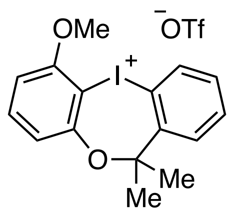

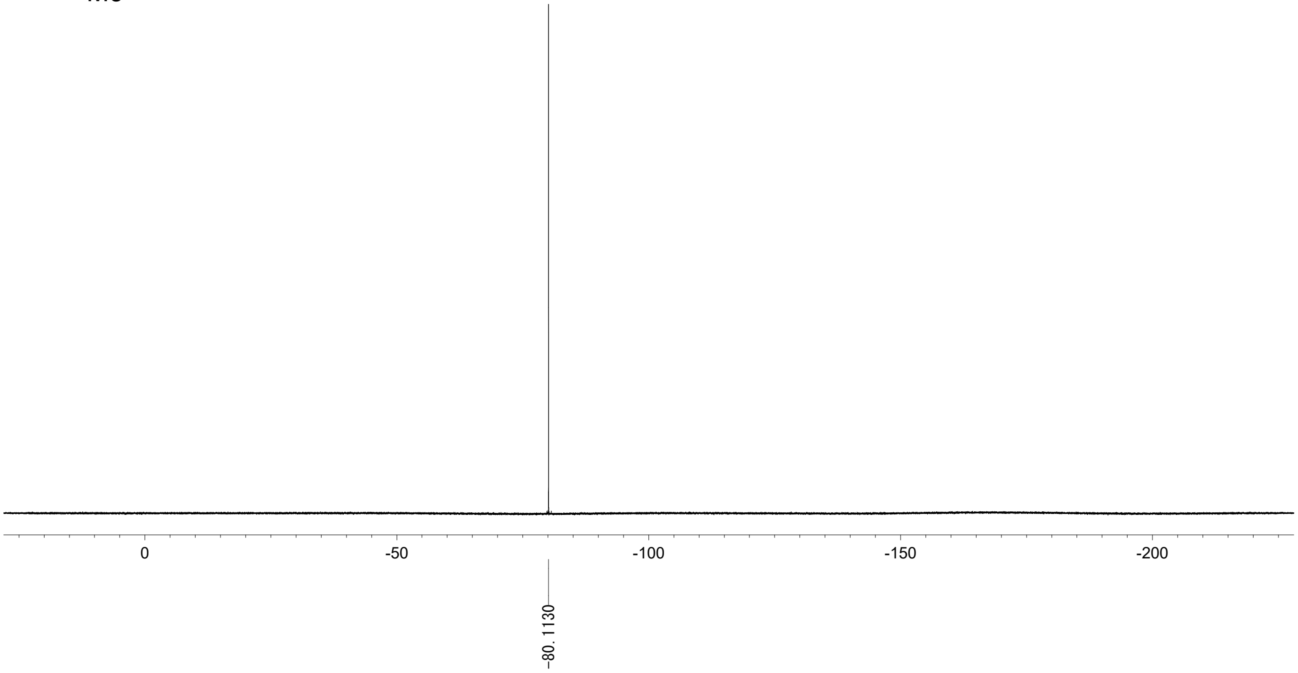

6,8-Dimethoxy-11,11-dimethyl-11*H*-dibenzo[*b*,*f*][1,4]iodaoxepin-5-ium trifluoromethanesulfonate (3ga)
^{1}H NMR (600 MHz, CD_3OD)

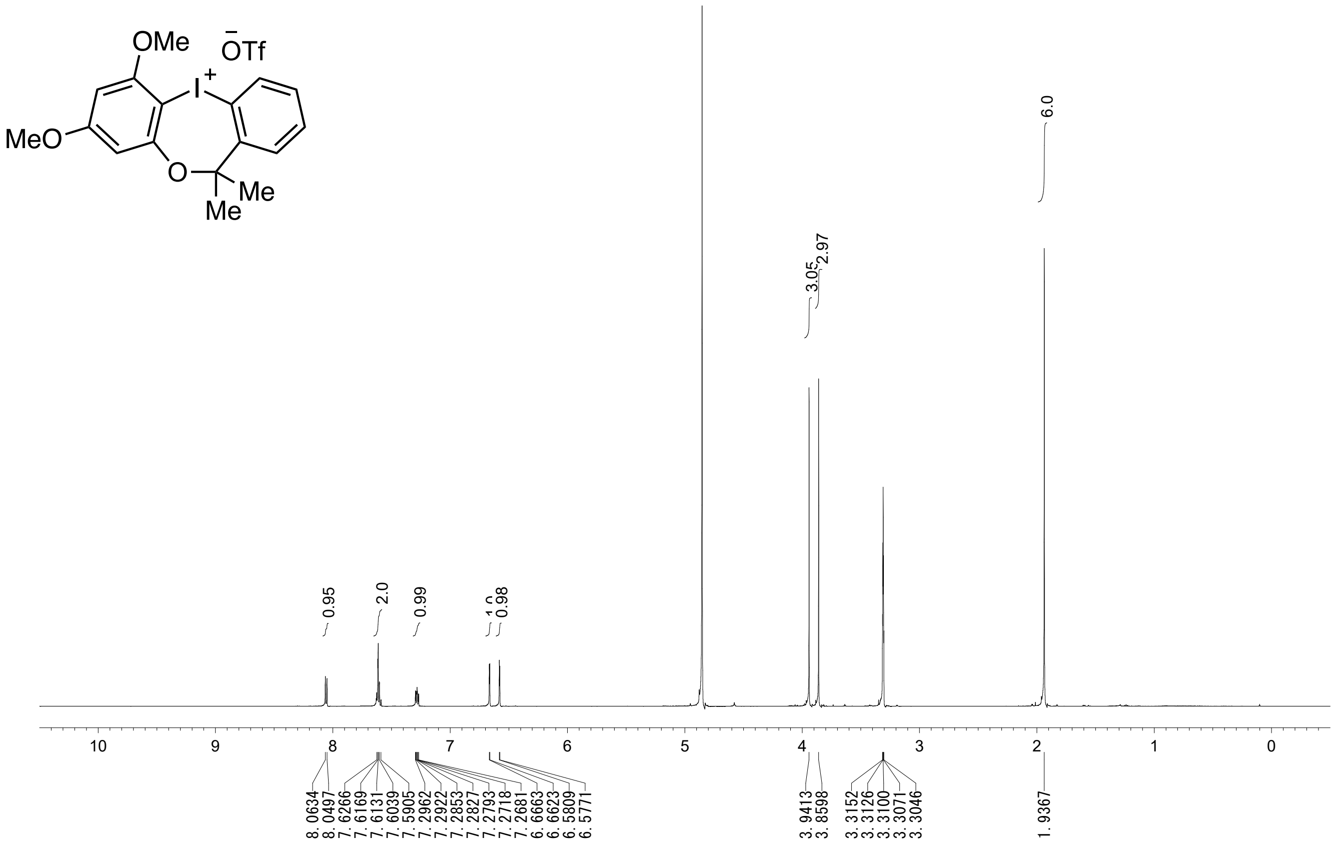

^{13}C{^{1}H} NMR (150 MHz, CD_3OD)

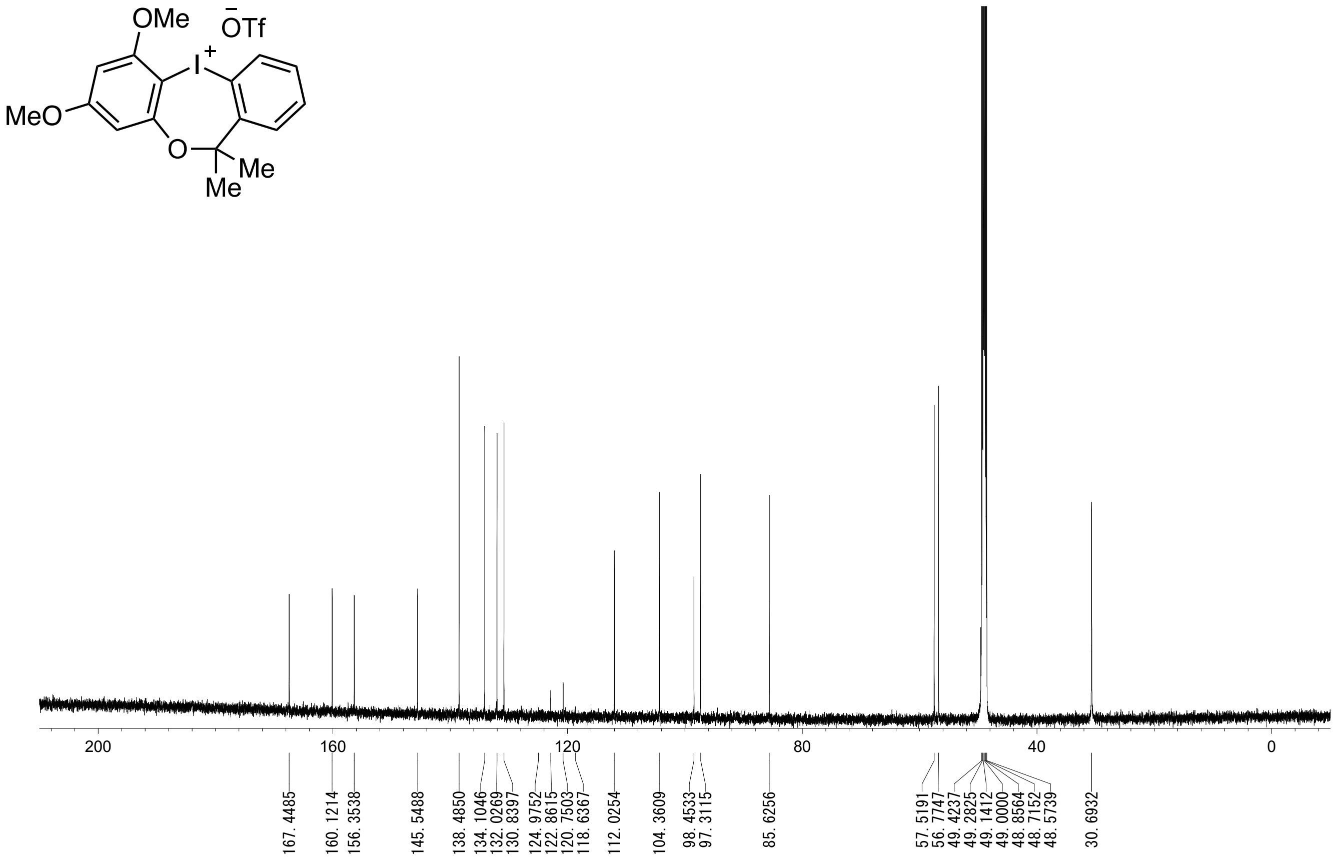

^{19}F NMR (565 MHz, CD_3OD)

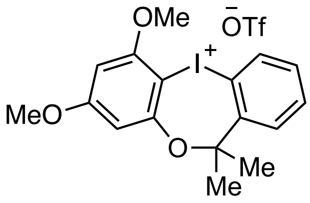

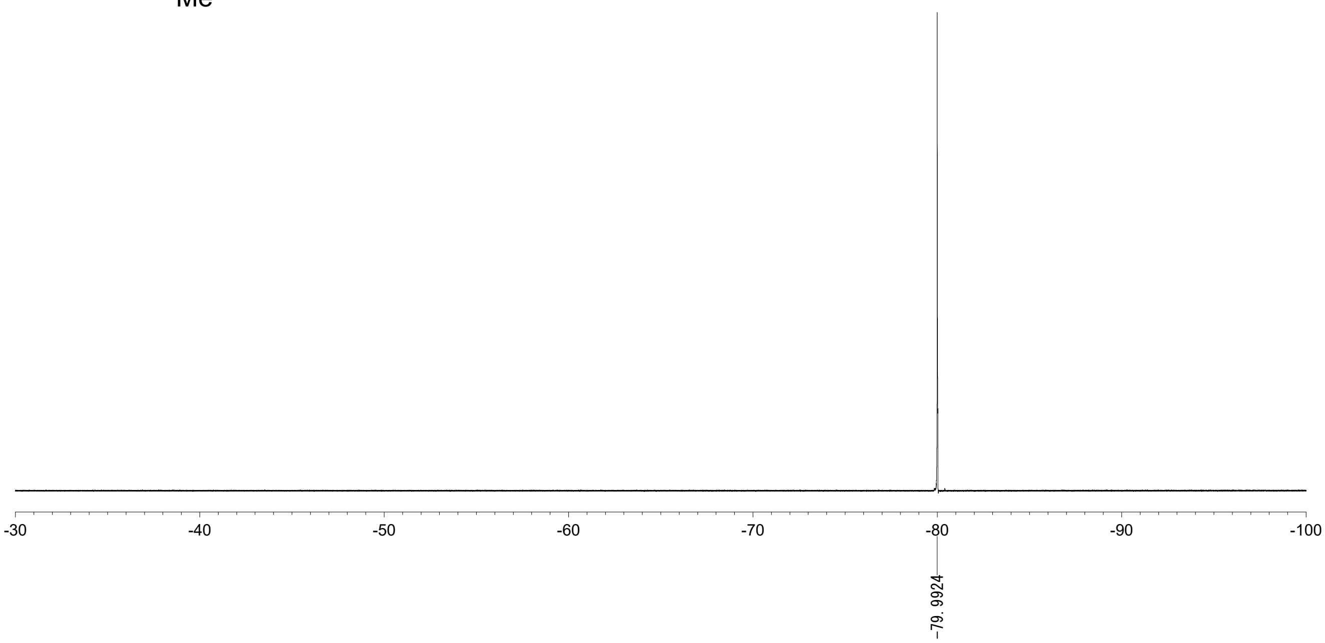

6-Chloro-11,11-dimethyl-11*H*-dibenzo[*b*,*f*][1,4]iodaoxepin-5-ium trifluoromethanesulfonate (3ha)

^{1}H NMR (400 MHz, CD_3OD)

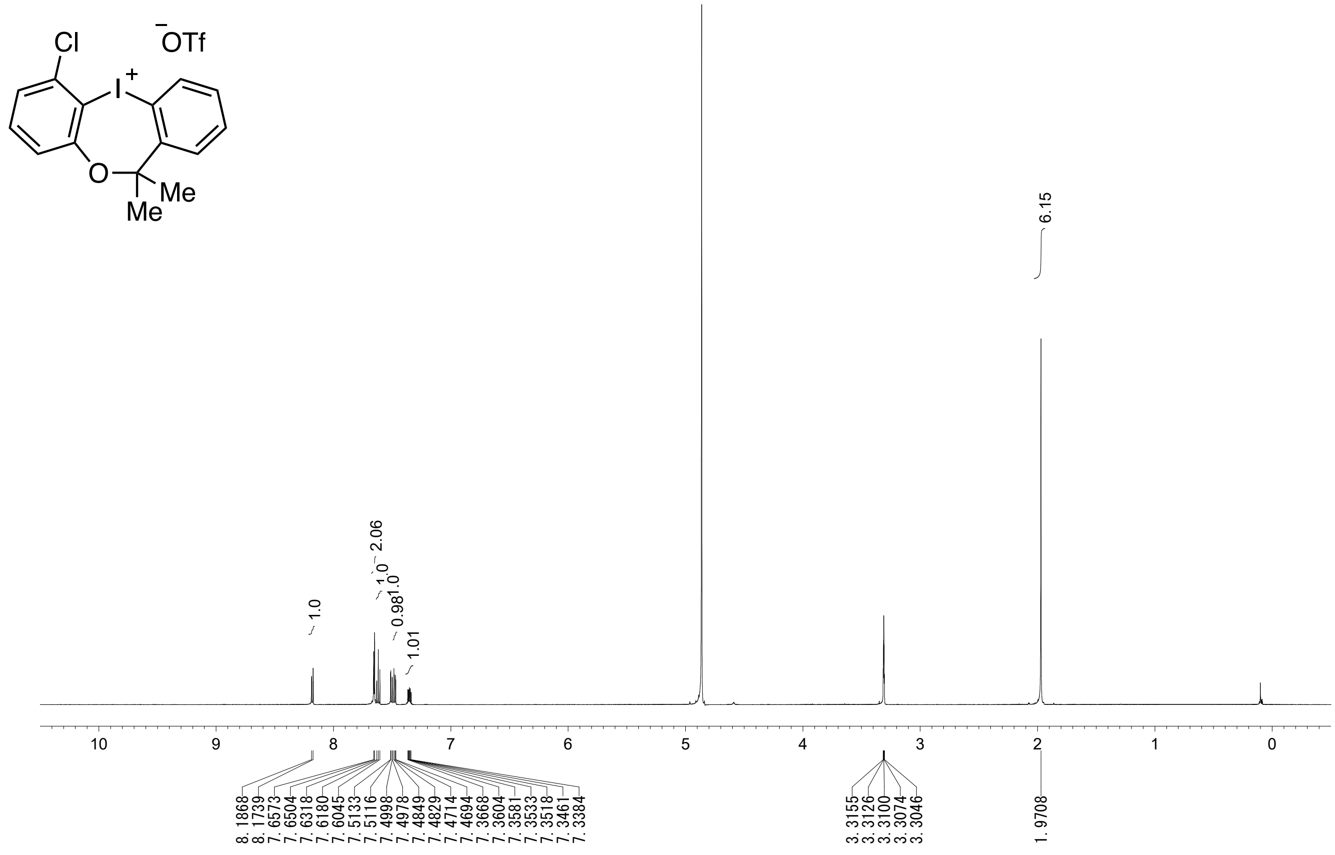

^{13}C{^{1}H} NMR (150 MHz, CD_3OD)

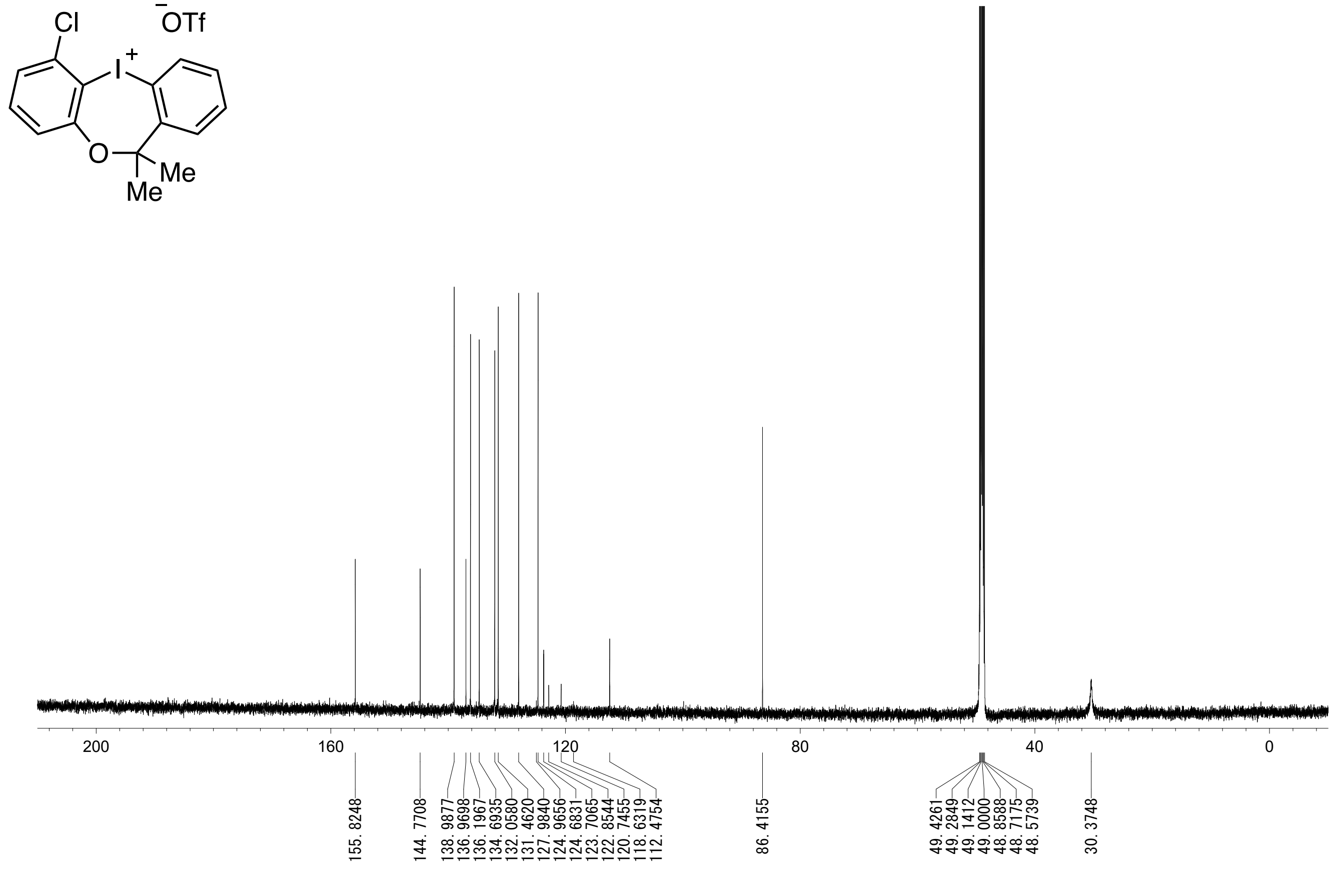

^{19}F NMR (376 MHz, CD_3OD)

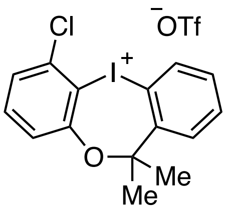

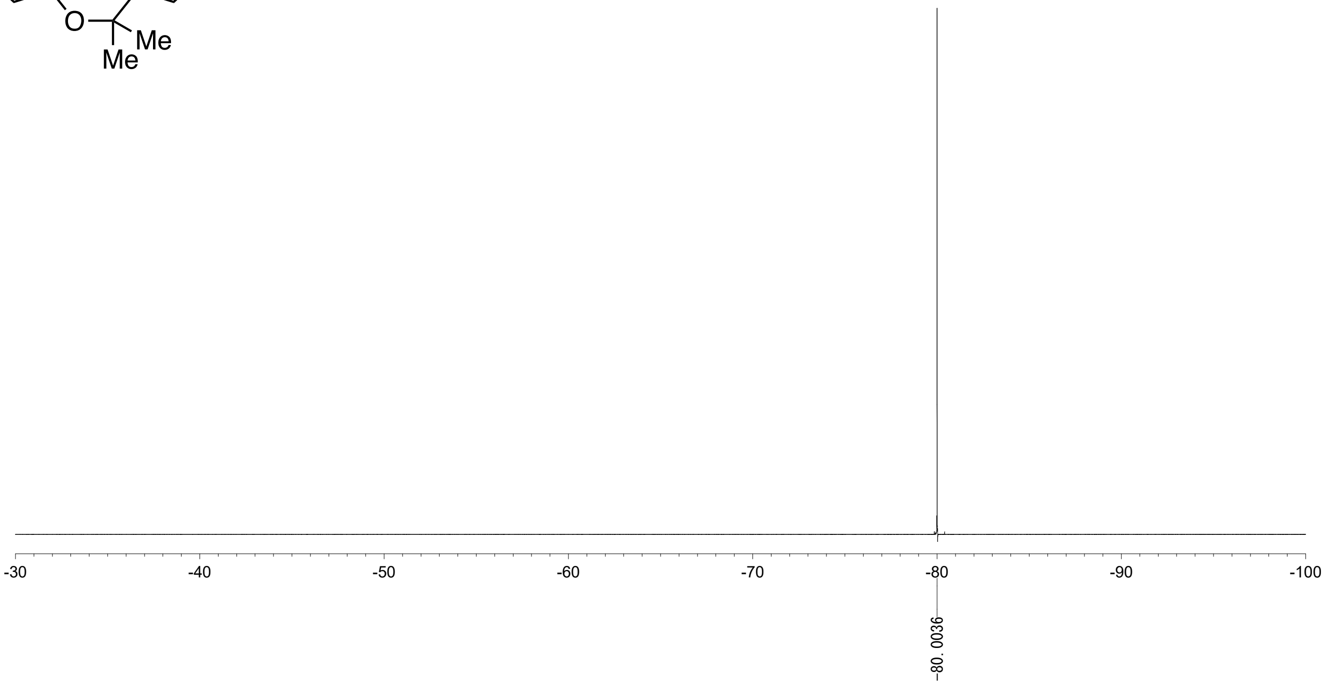

8,8-Dimethyl-8*H*-benzo[*f*]naphtho[1,2-*b*][1,4]iodaoxepin-13-ium trifluoromethanesulfonate (3ia)
^{1}H NMR (400 MHz, CD_3OD)

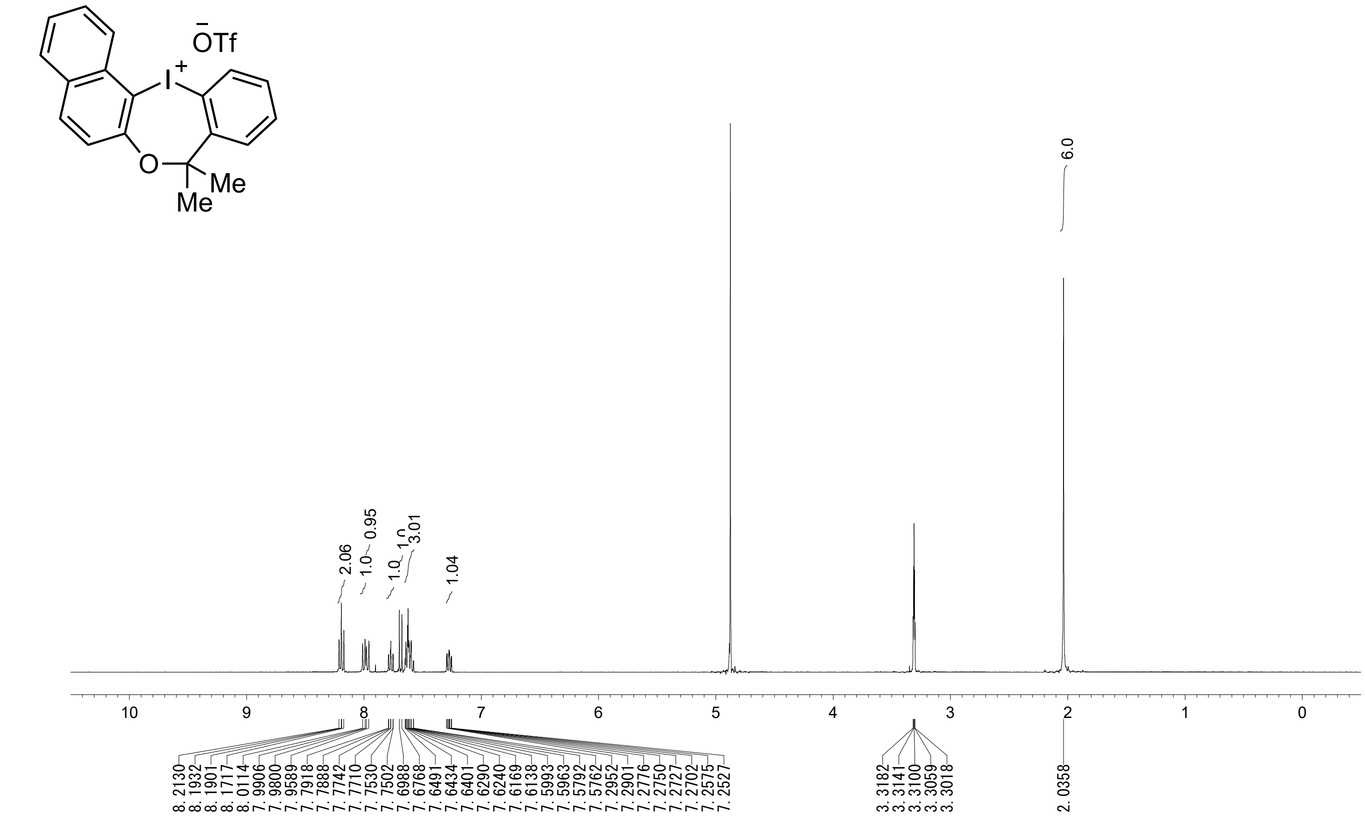

^{13}C{^{1}H} NMR (150 MHz, CD_3OD)

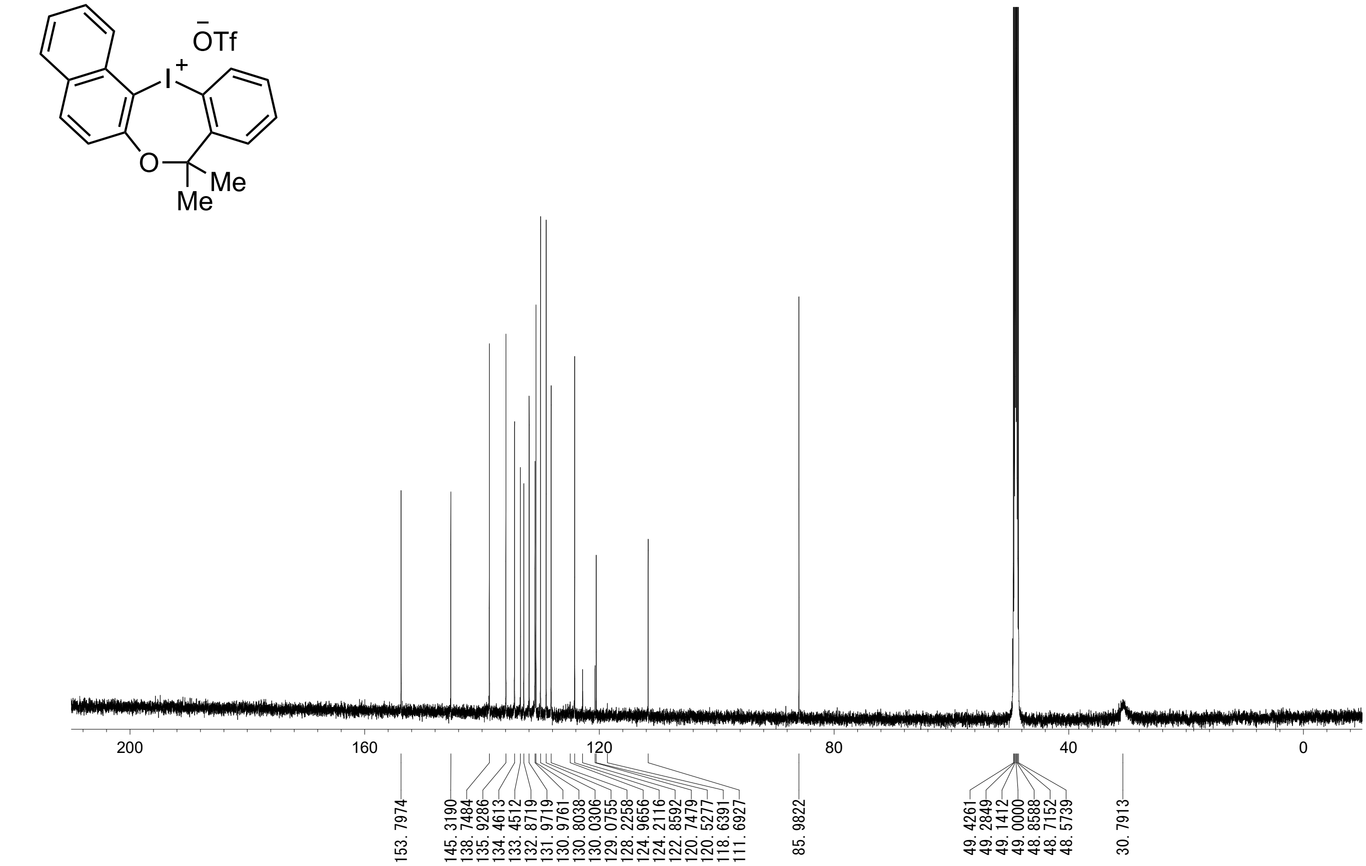

^{19}F NMR (376 MHz, CD_3OD)

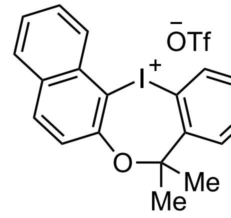

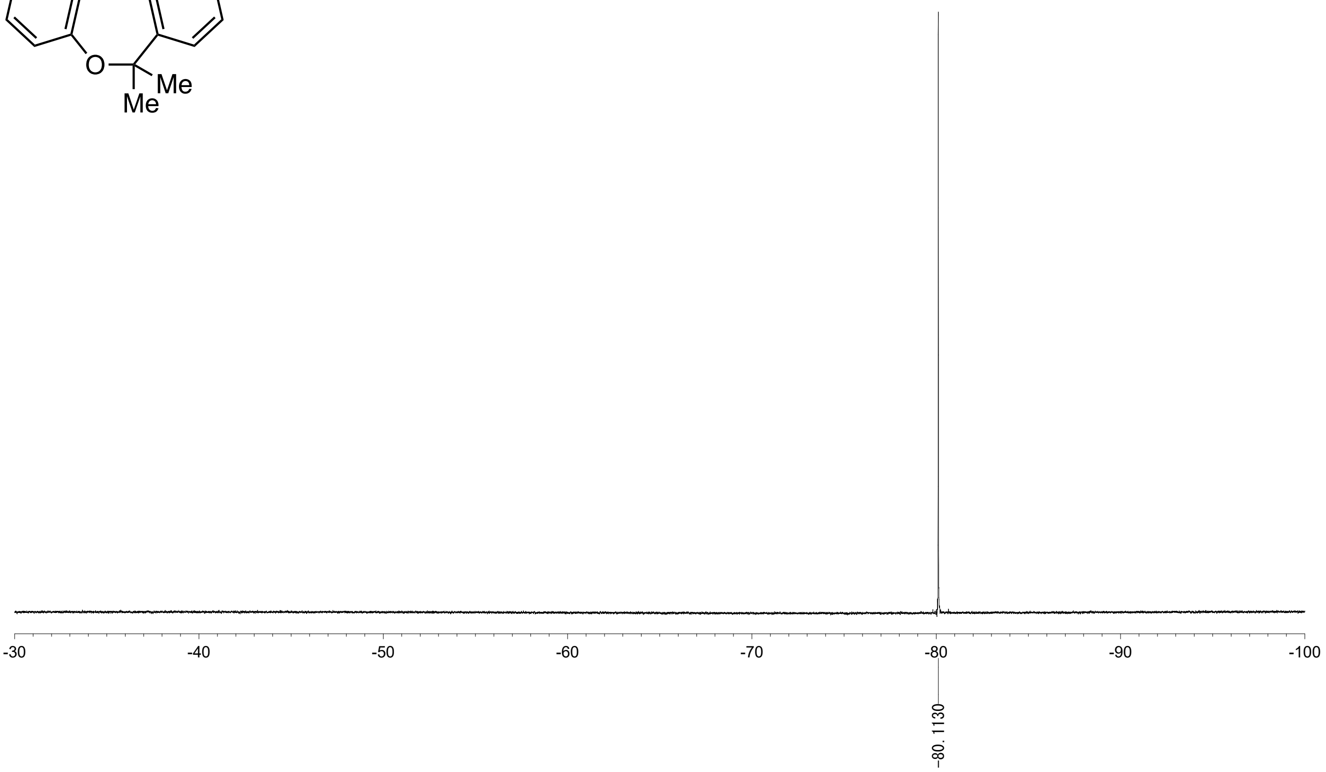

8,11,11-Trimethyl-11*H*-dibenzo[*b*,*f*][1,4]iodaoxepin-5-ium trifluoromethanesulfonate (3ja) and 7,11,11-trimethyl-11*H*-dibenzo[*b*,*f*][1,4]iodaoxepin-5-ium trifluoromethanesulfonate (3ja')

^{1}H NMR (600 MHz, $CDCl_3$)

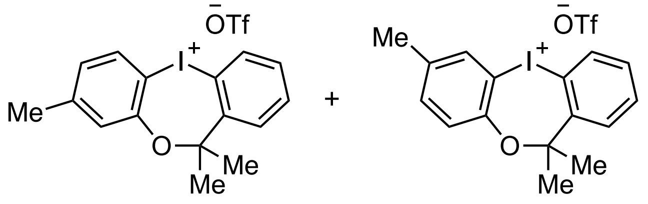

3ja 3ja'

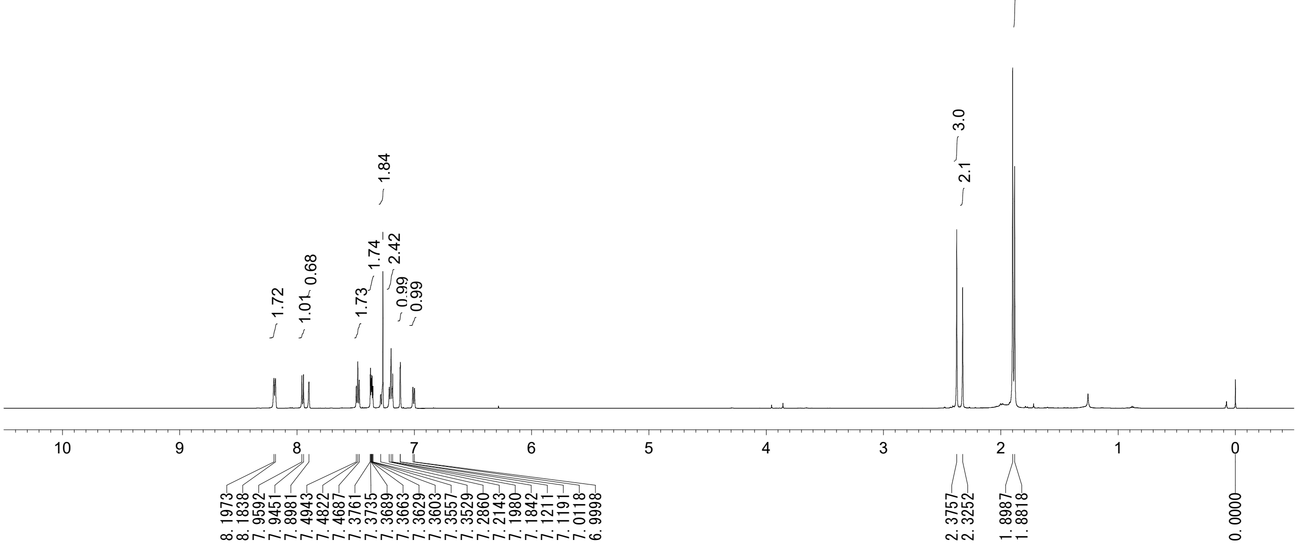

^{13}C{^{1}H} NMR (150 MHz, $CDCl_3$)

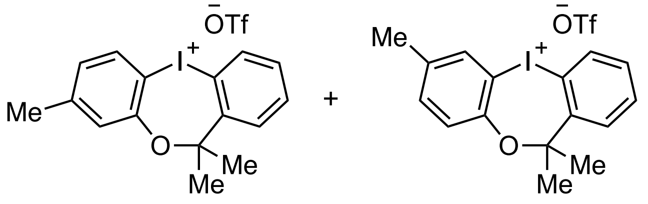

3ja 3ja'

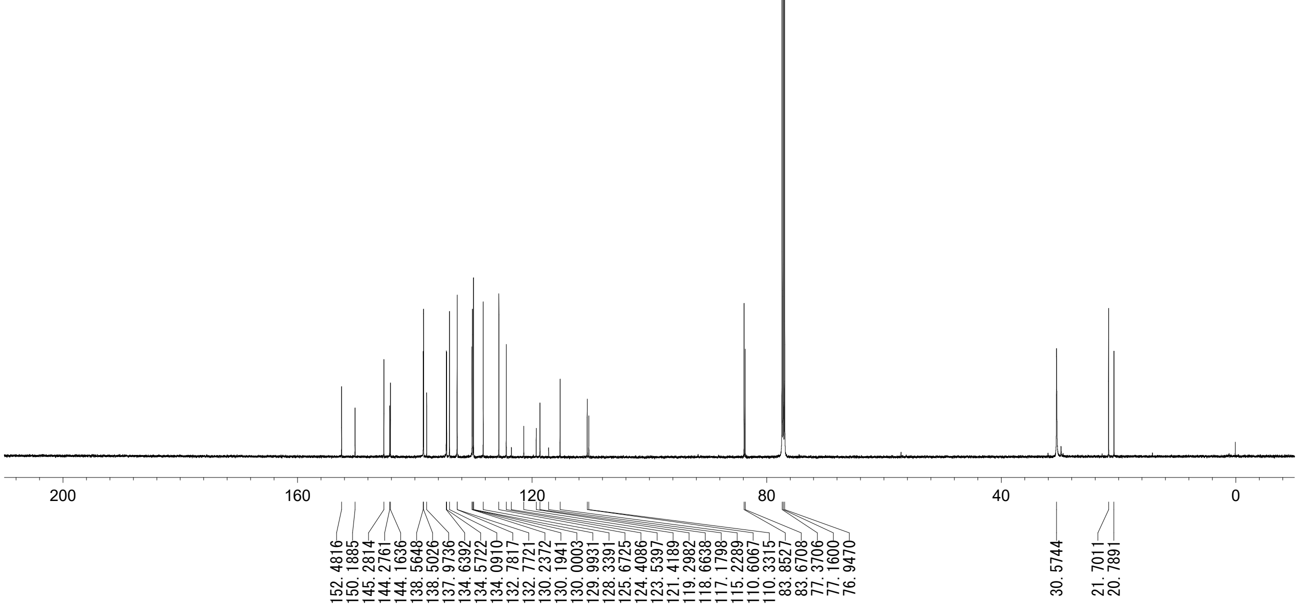

^{19}F NMR (565 MHz, $CDCl_3$)

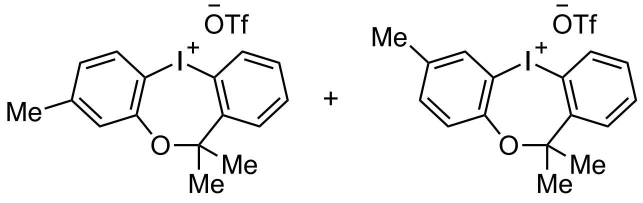

3ja **3ja'**

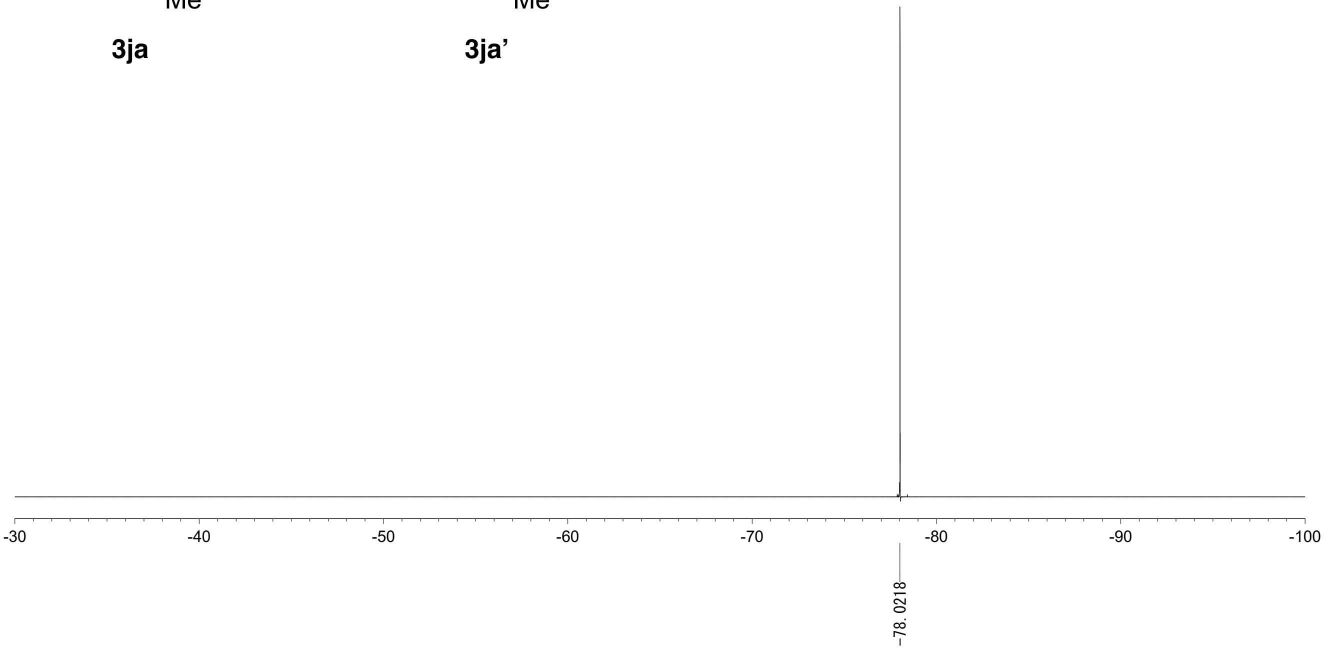

7-Chloro-11,11-dimethyl-11*H*-dibenzo[*b*,*f*][1,4]iodaoxepin-5-ium trifluoromethanesulfonate (3ka) and 8-chloro-11,11-dimethyl-11*H*-dibenzo[*b*,*f*][1,4]iodaoxepin-5-ium trifluoromethanesulfonate (3ka')

^{1}H NMR (600 MHz, CD_3OD)

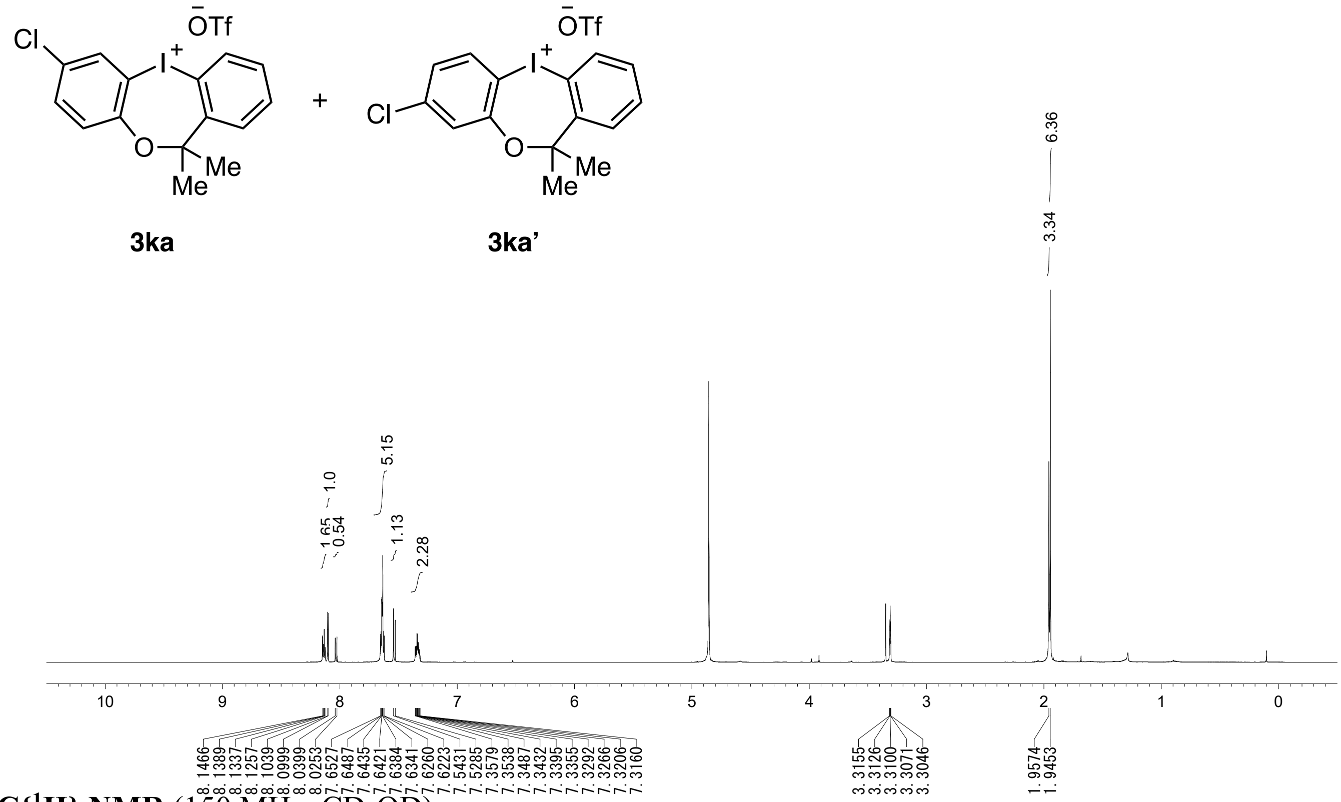

^{13}C{^{1}H} NMR (150 MHz, CD_3OD)

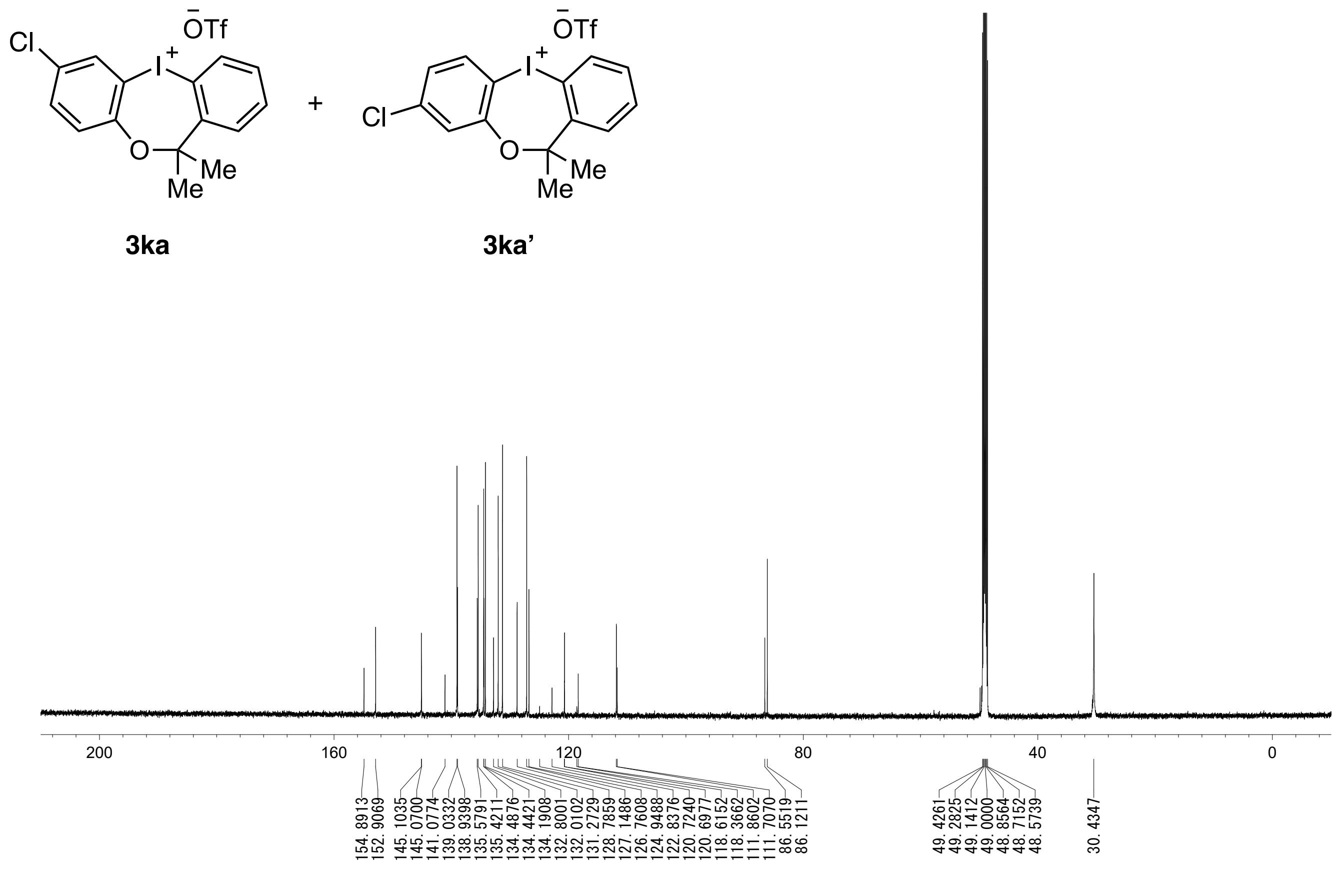

^{19}F NMR (565 MHz, CD_3OD)

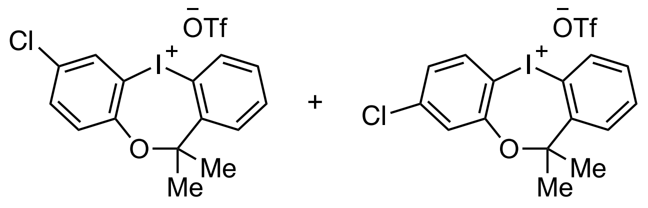

3ka **3ka'**

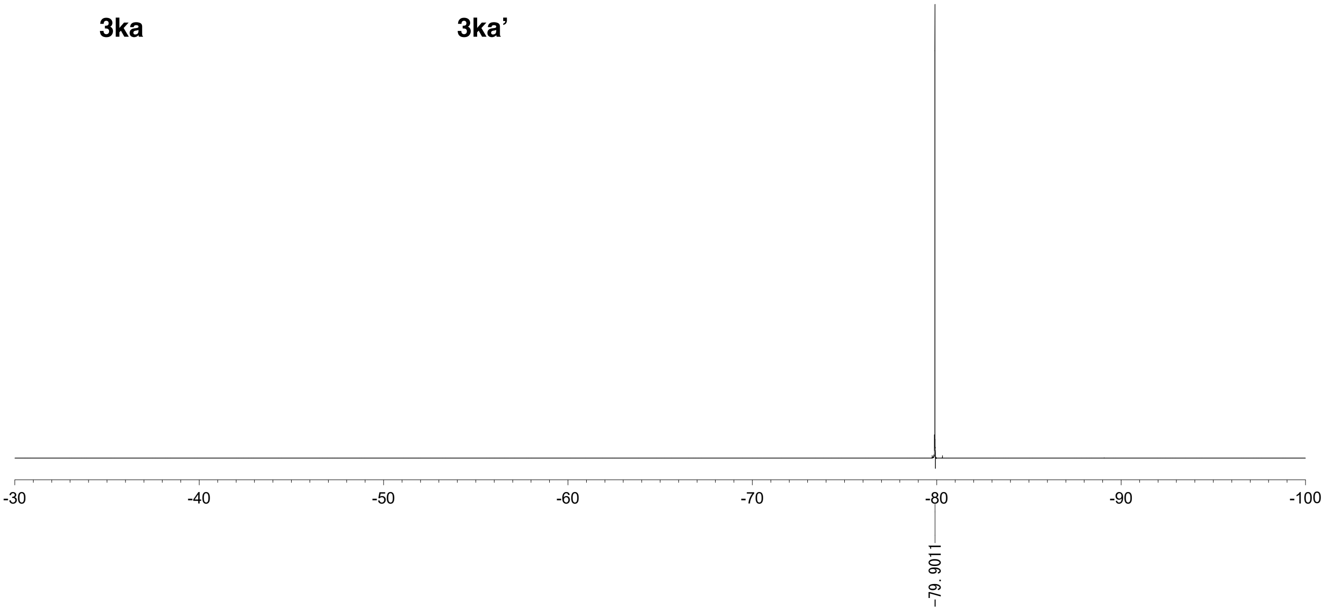

11,11-Diethyl-11*H*-dibenzo[*b*,*f*][1,4]iodaoxepin-5-ium trifluoromethanesulfonate (3ab)
^{1}H NMR (600 MHz, CD_3OD)

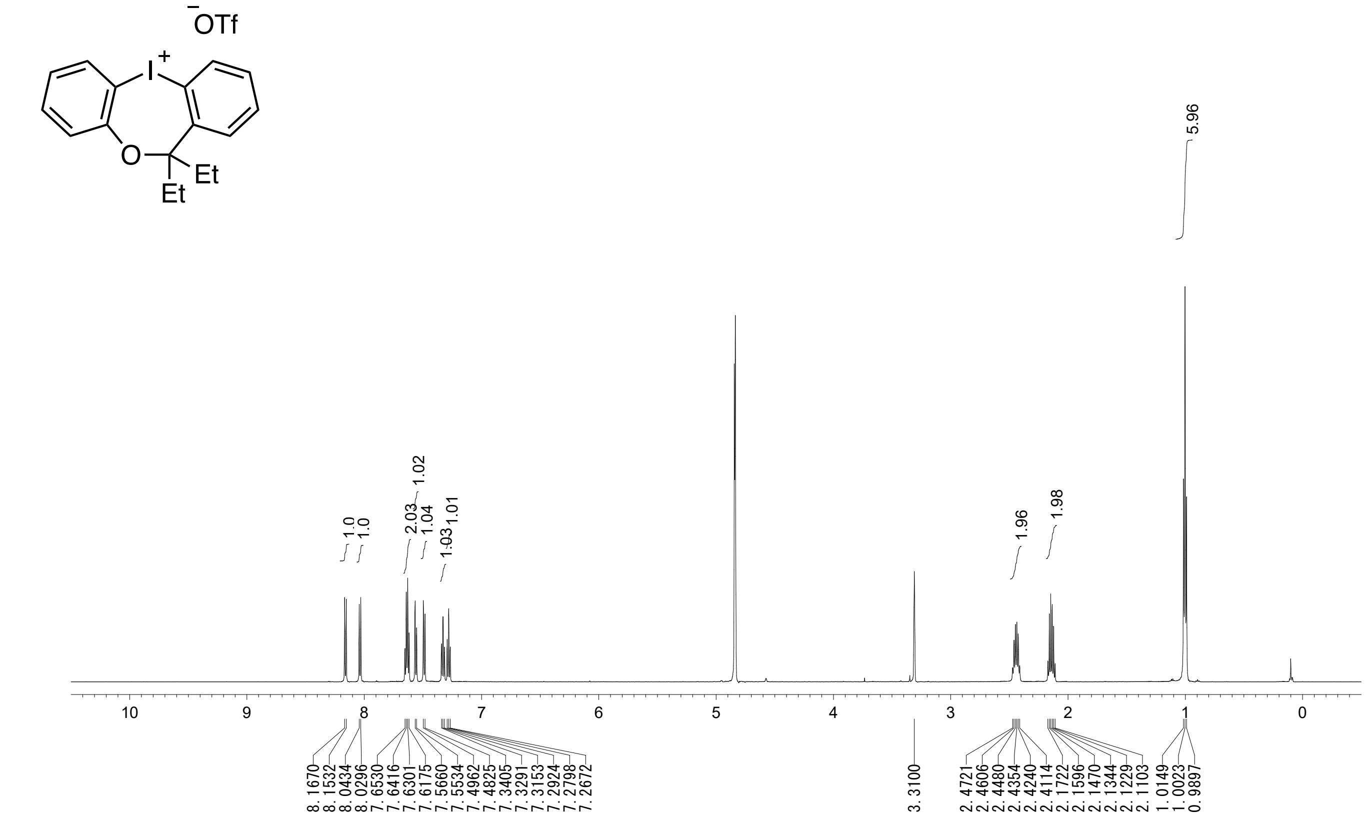

^{13}C{^{1}H} NMR (150 MHz, CD_3OD)

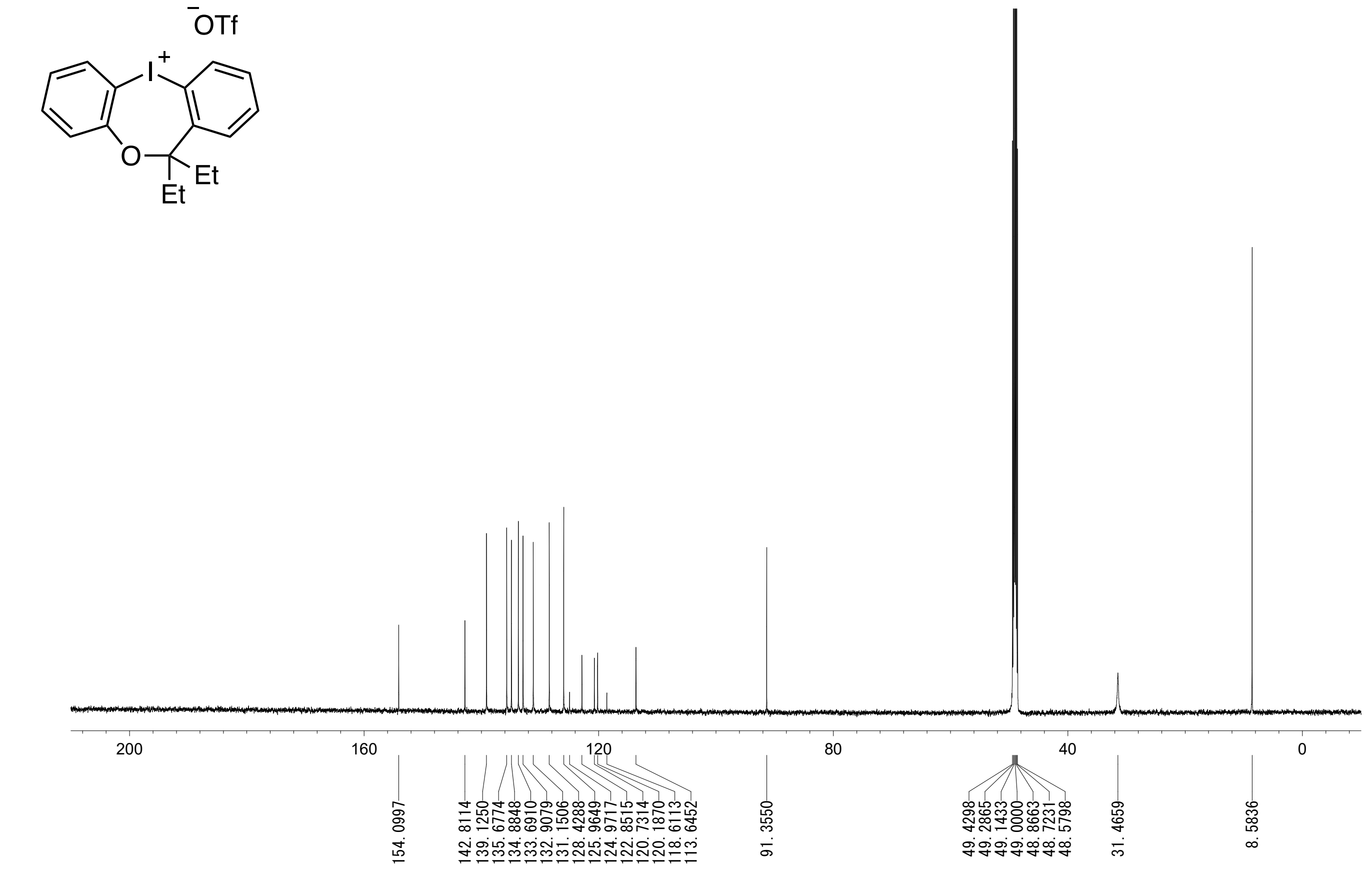

^{19}F NMR (376 MHz, CD_3OD)

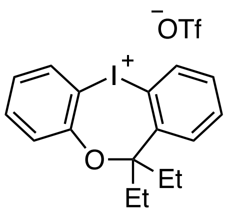

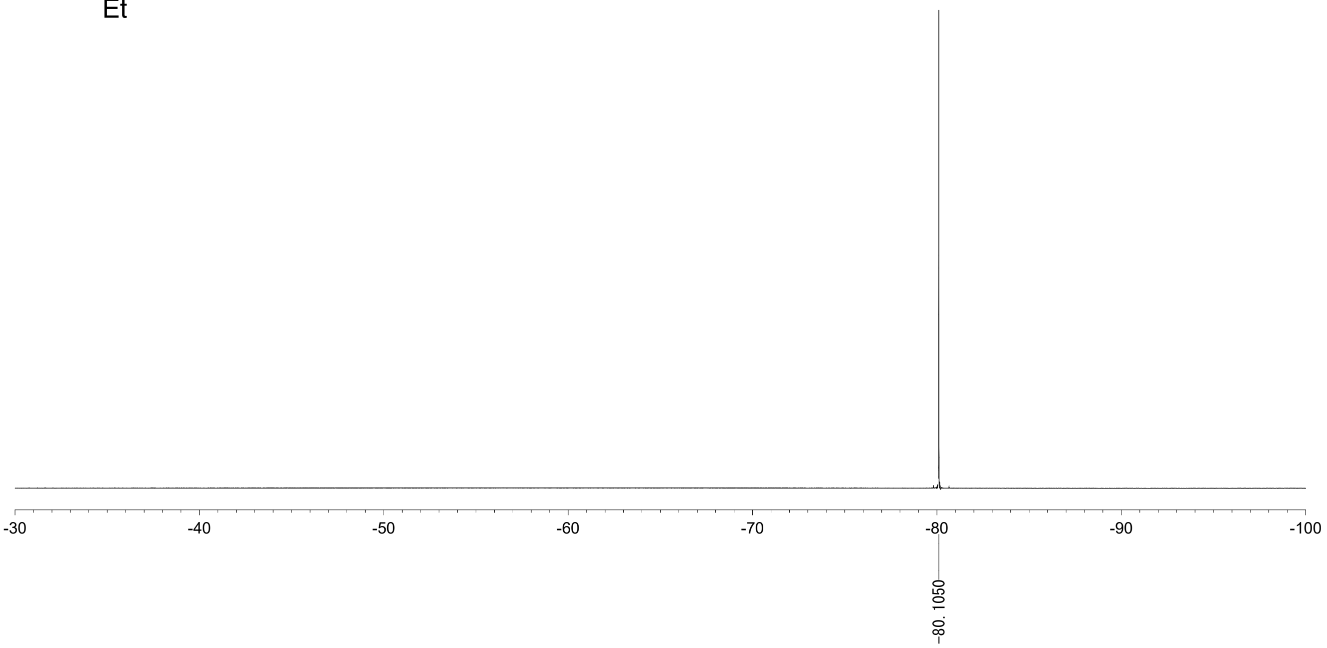

11-Methyl-11-phenyl-11*H*-dibenzo[*b*,*f*][1,4]iodaoxepin-5-ium trifluoromethanesulfonate (3ac)

^{1}H NMR (400 MHz, CD_3OD)

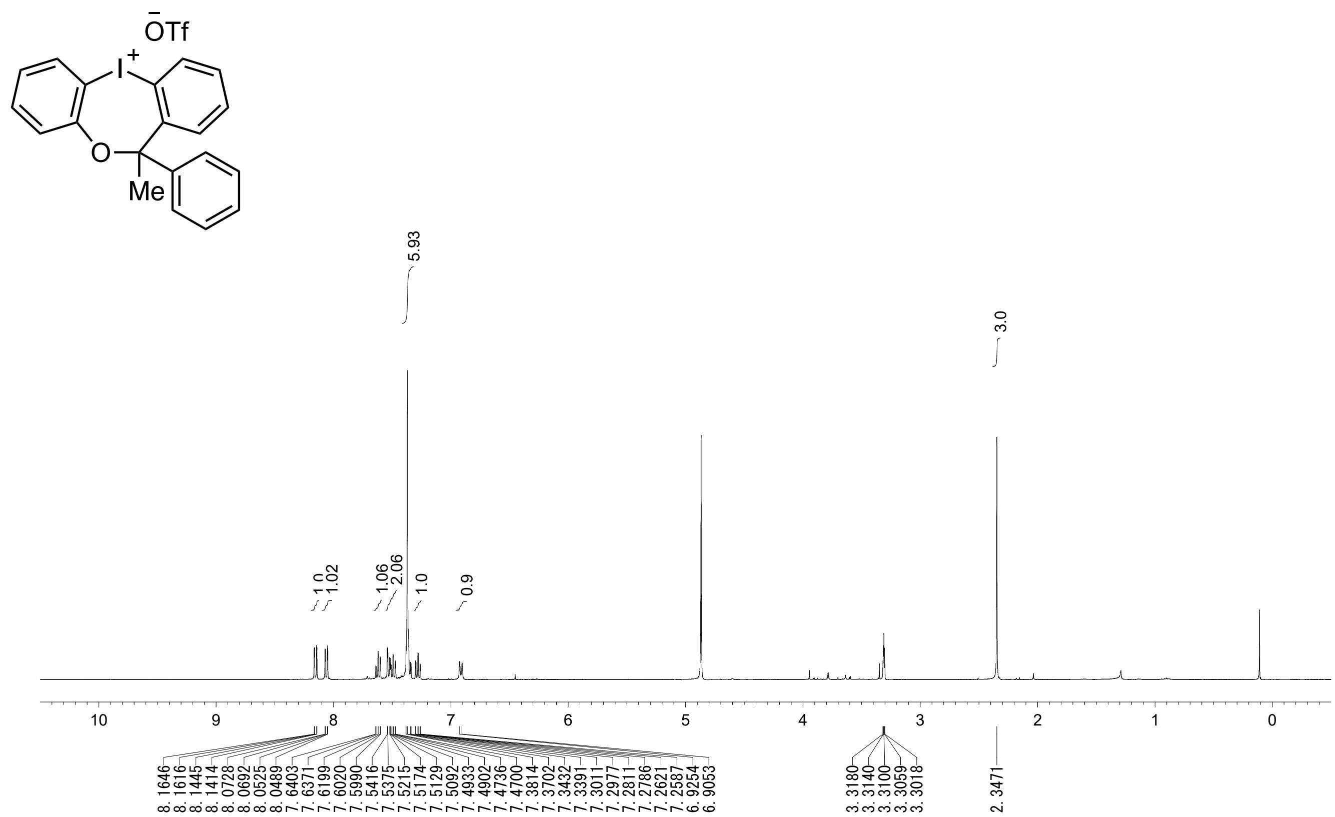

^{13}C{^{1}H} NMR (150 MHz, CD_3OD)

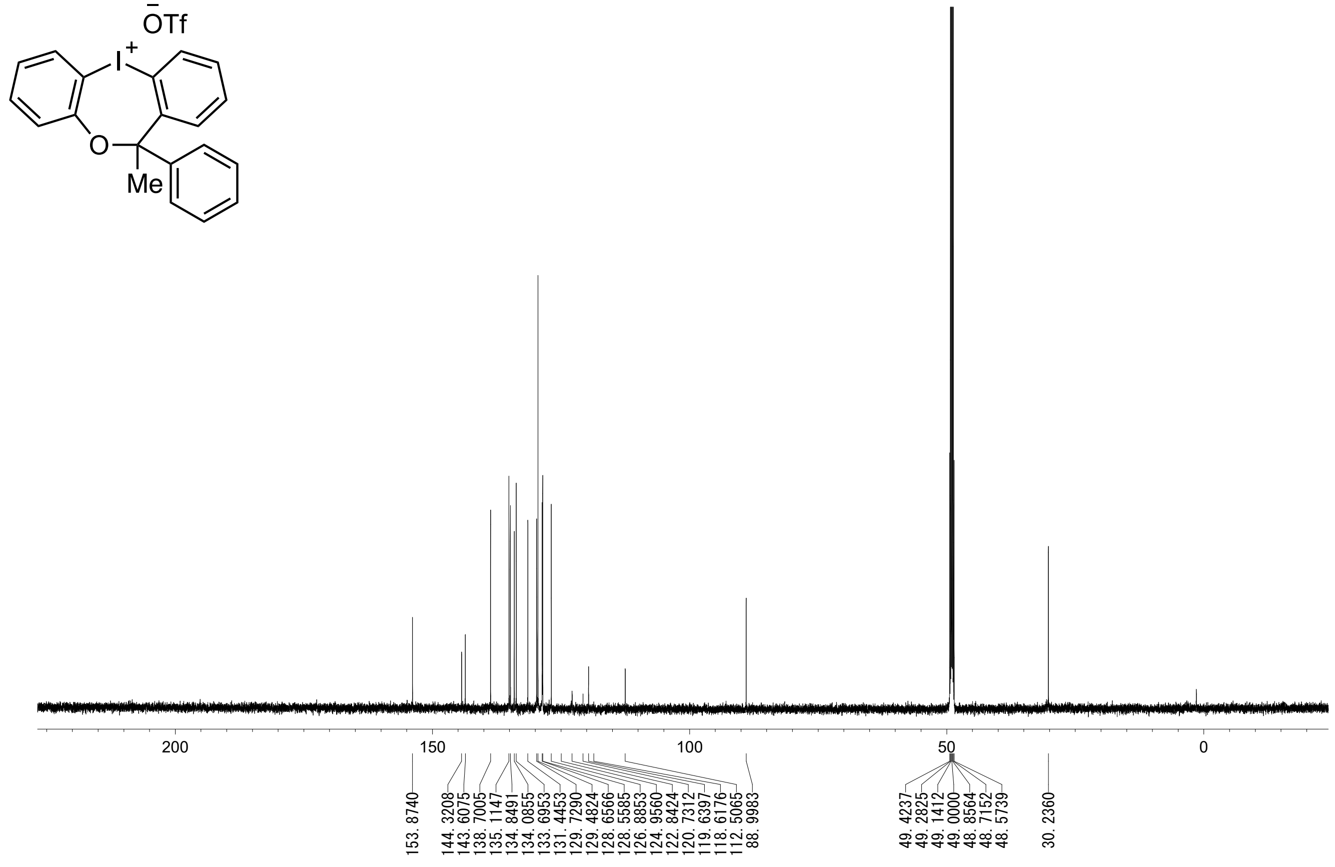

^{19}F NMR (376 MHz, CD_3OD)

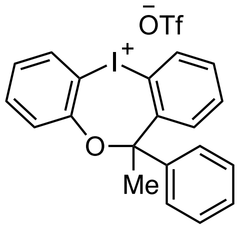

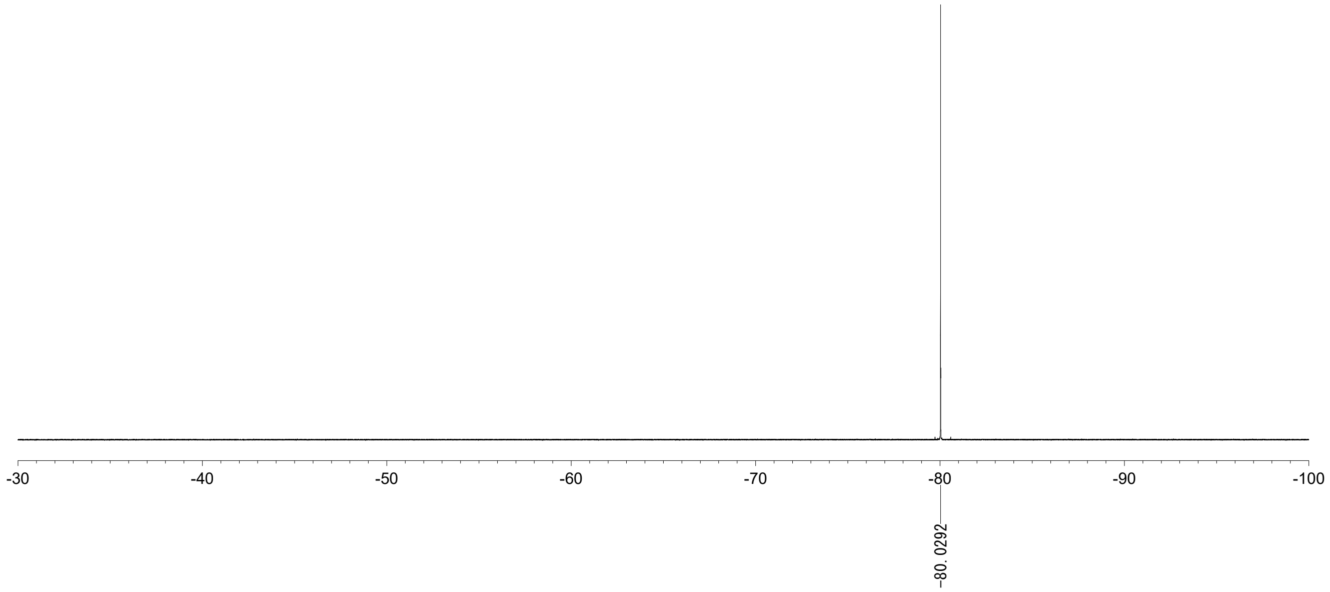

11-Methyl-11-(*p*-tolyl)-11*H*-dibenzo[*b*,*f*][1,4]iodaoxepin-5-ium trifluoromethanesulfonate (3ad)
^{1}H NMR (400 MHz, CD_3OD)

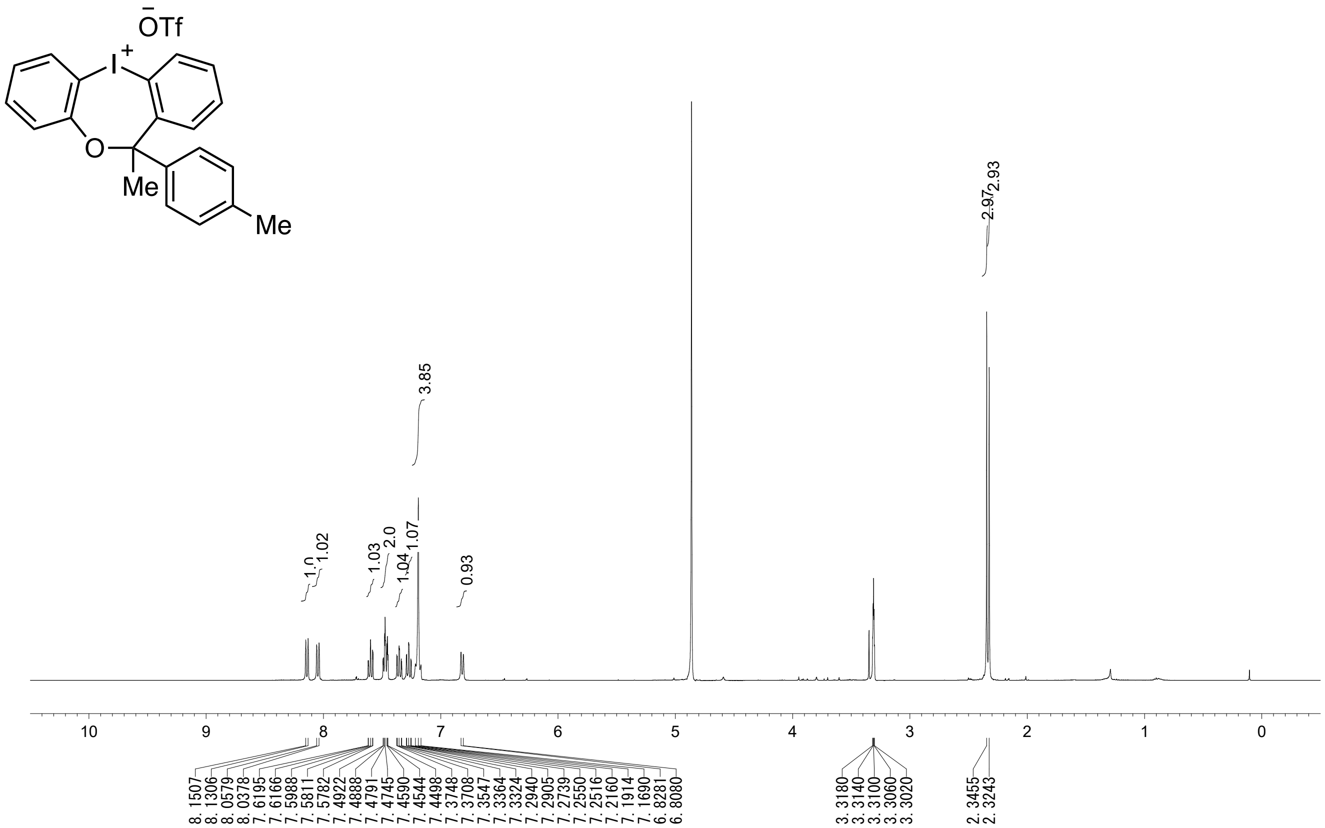

^{13}C{^{1}H} NMR (101 MHz, CD_3OD)

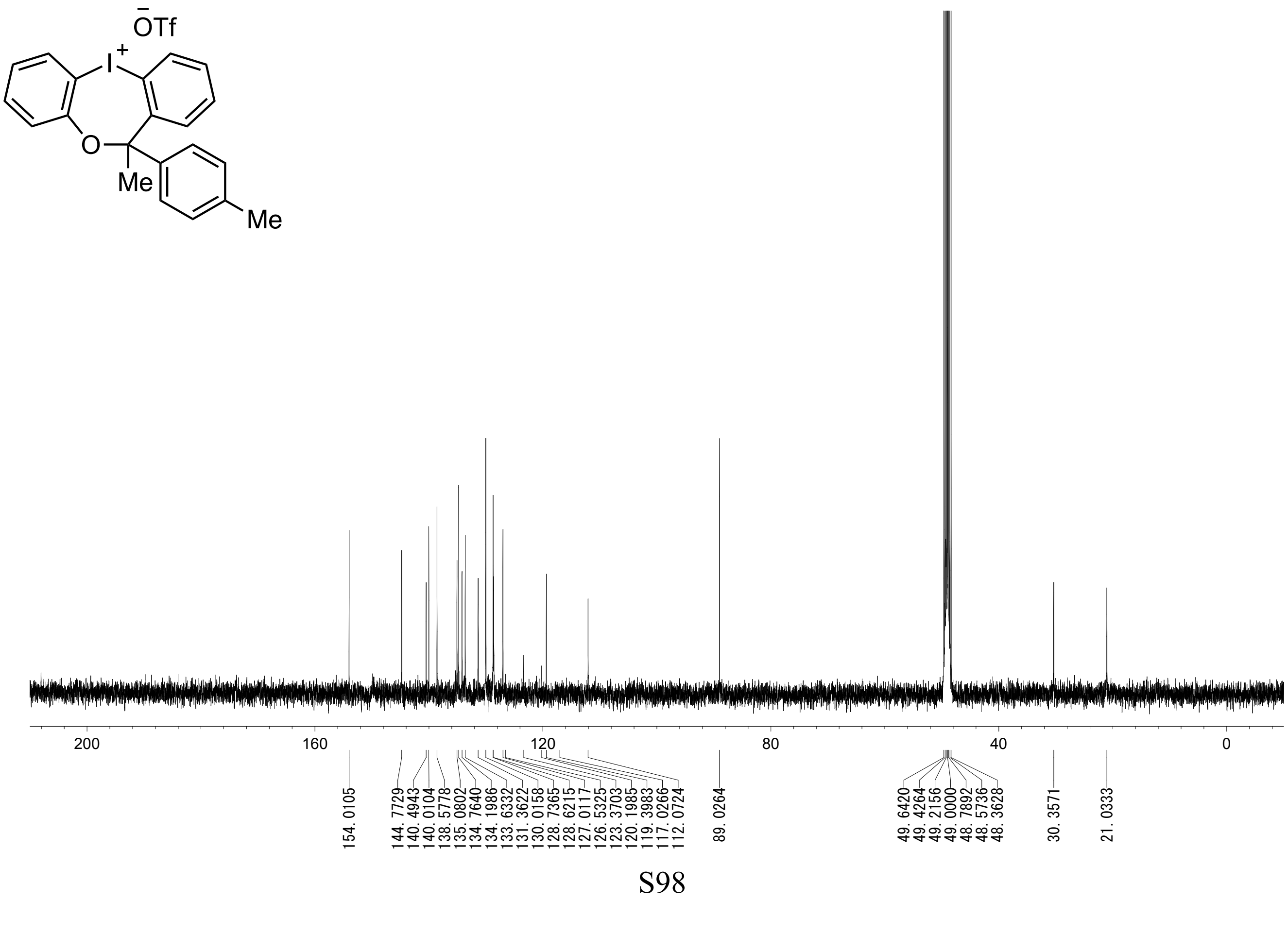

^{19}F NMR (376 MHz, CD_3OD)

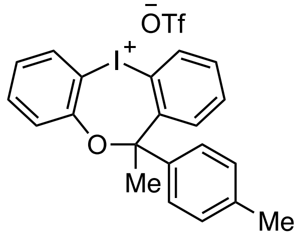

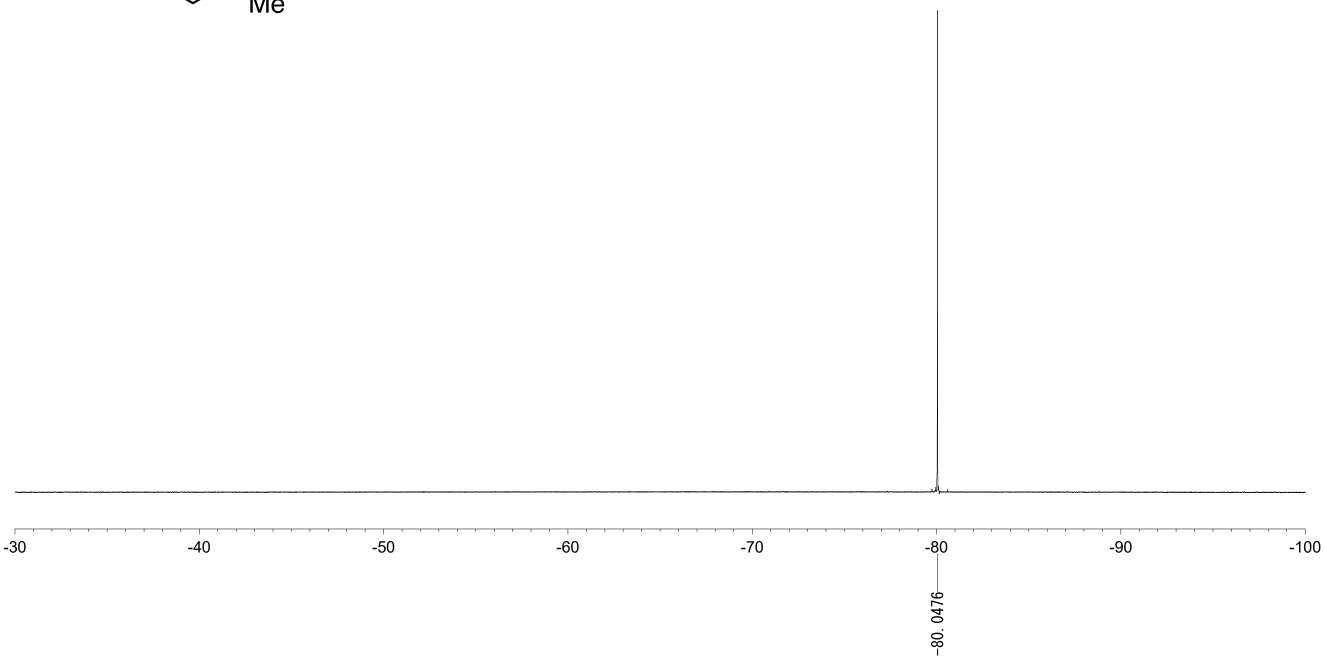

11-Methyl-11-(trifluoromethyl)-11*H*-dibenzo[*b*,*f*][1,4]iodaoxepin-5-ium trifluoromethanesulfonate (3ae)

^{1}H NMR (600 MHz, CD_3OD)

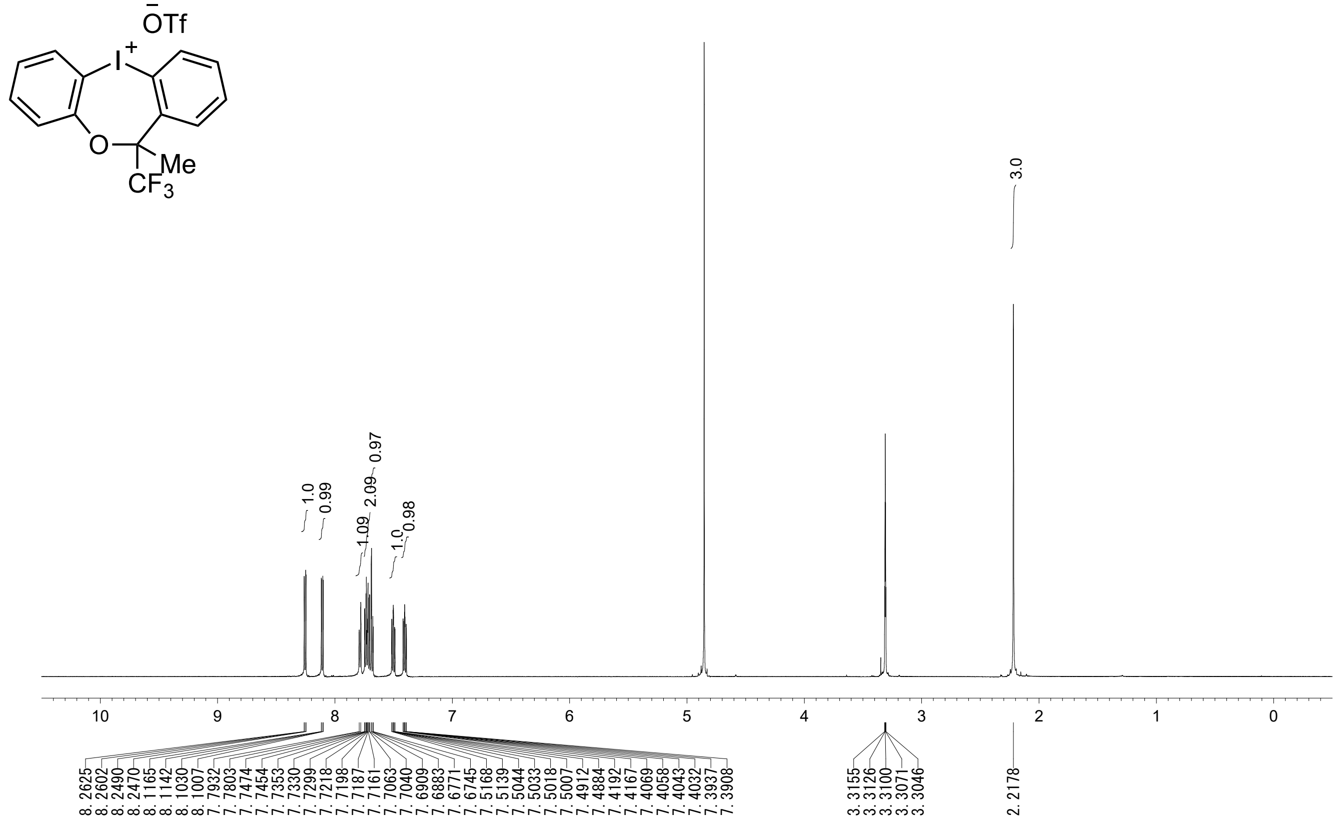

^{13}C{^{1}H} NMR (150 MHz, CD_3OD)

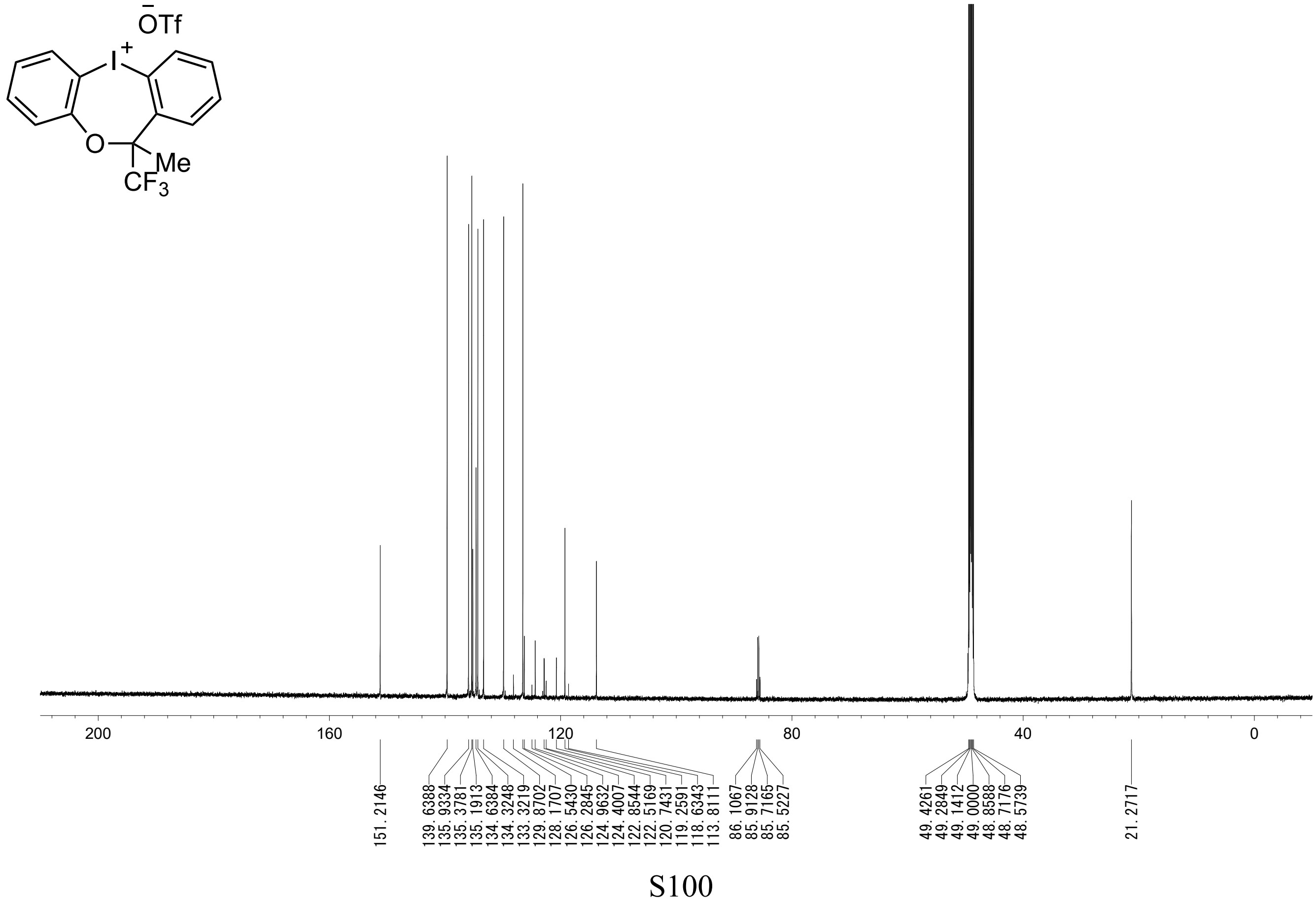

^{19}F NMR (376 MHz, CD_3OD)

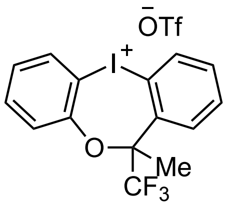

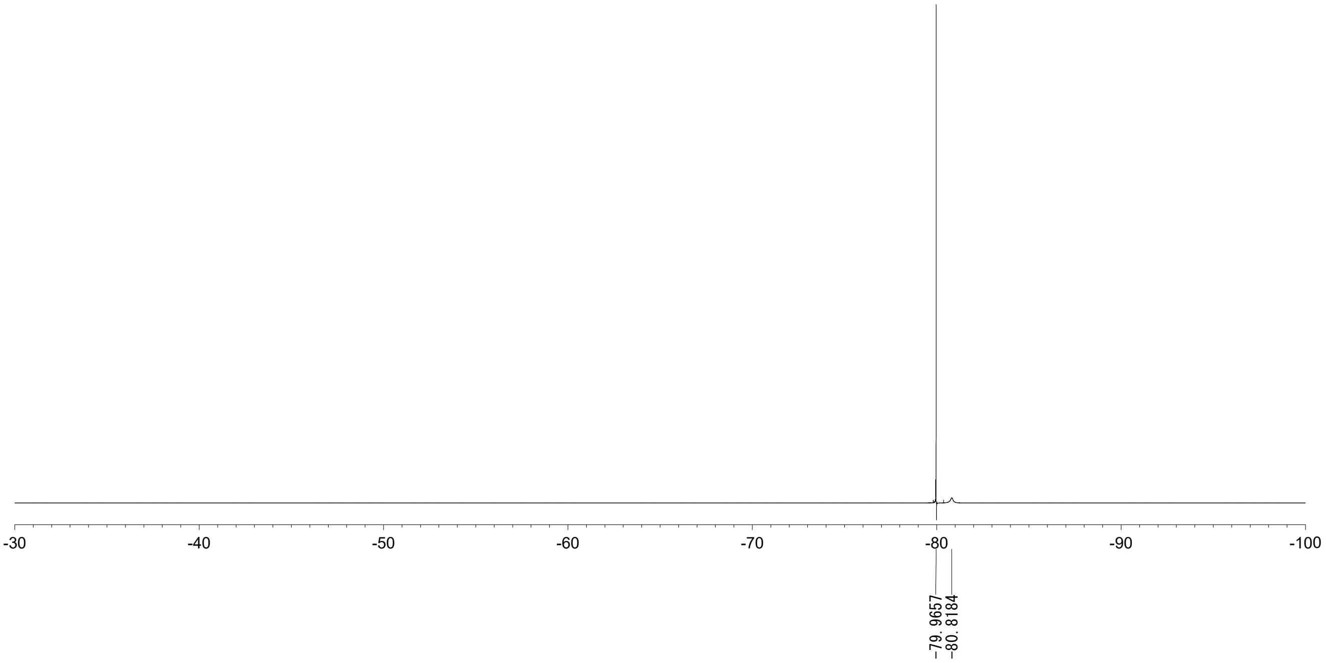

2,11,11-Trimethyl-11*H*-dibenzo[*b*,*f*][1,4]iodaoxepin-5-ium trifluoromethanesulfonate (3af)

^{1}H NMR (600 MHz, CD_3OD)

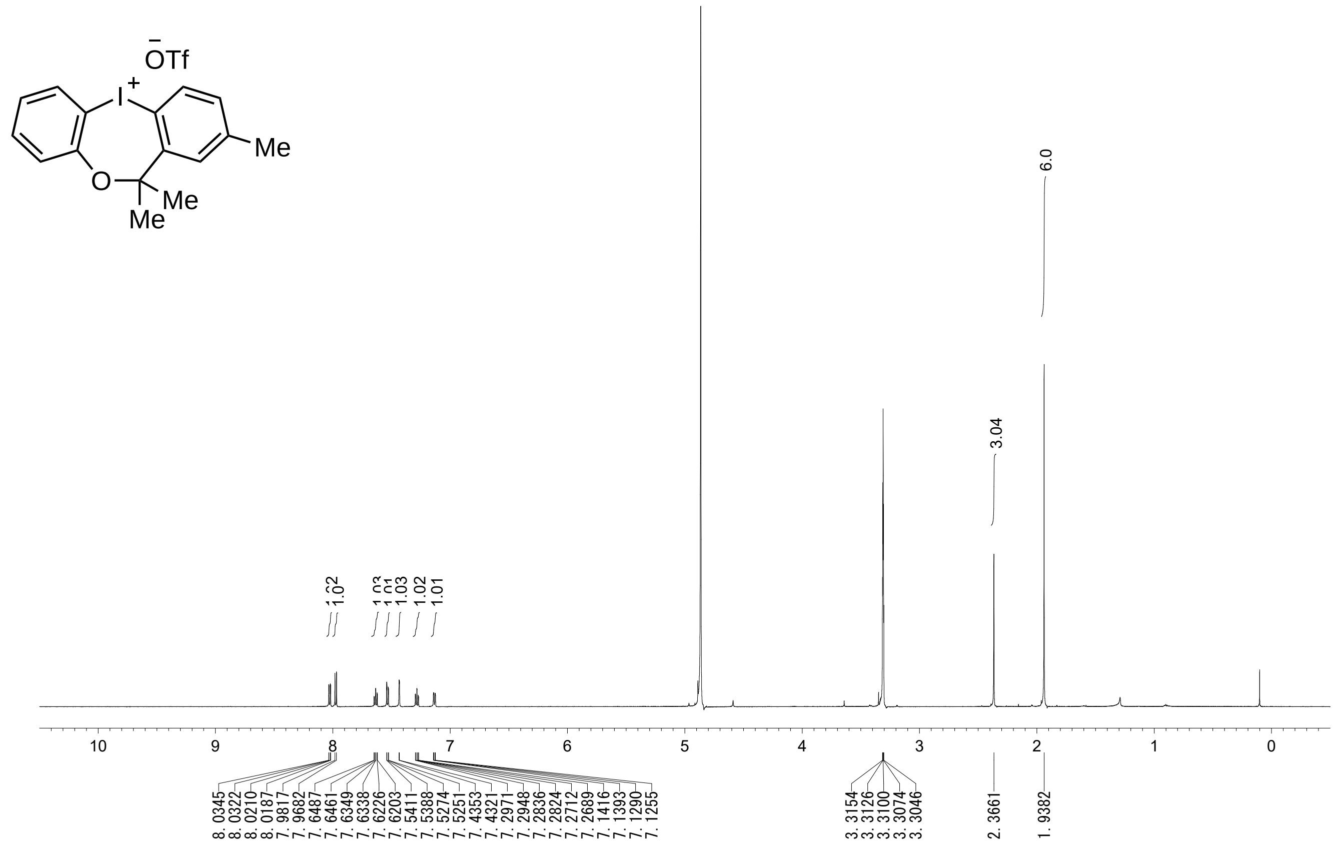

^{13}C{^{1}H} NMR (150 MHz, CD_3OD)

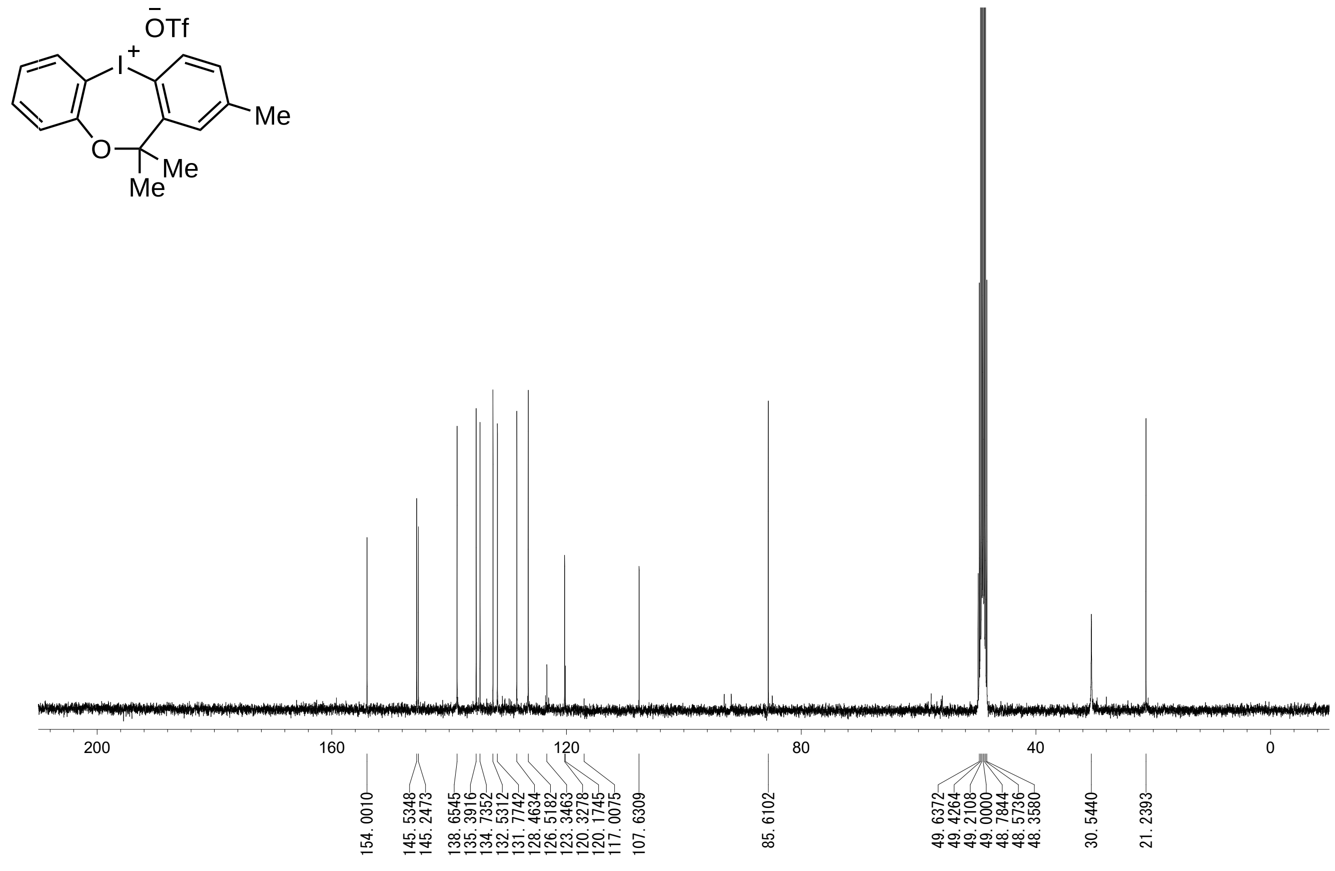

^{19}F NMR (376 MHz, CD_3OD)

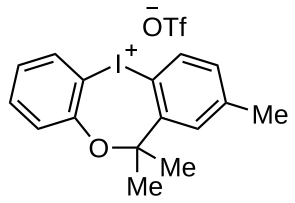

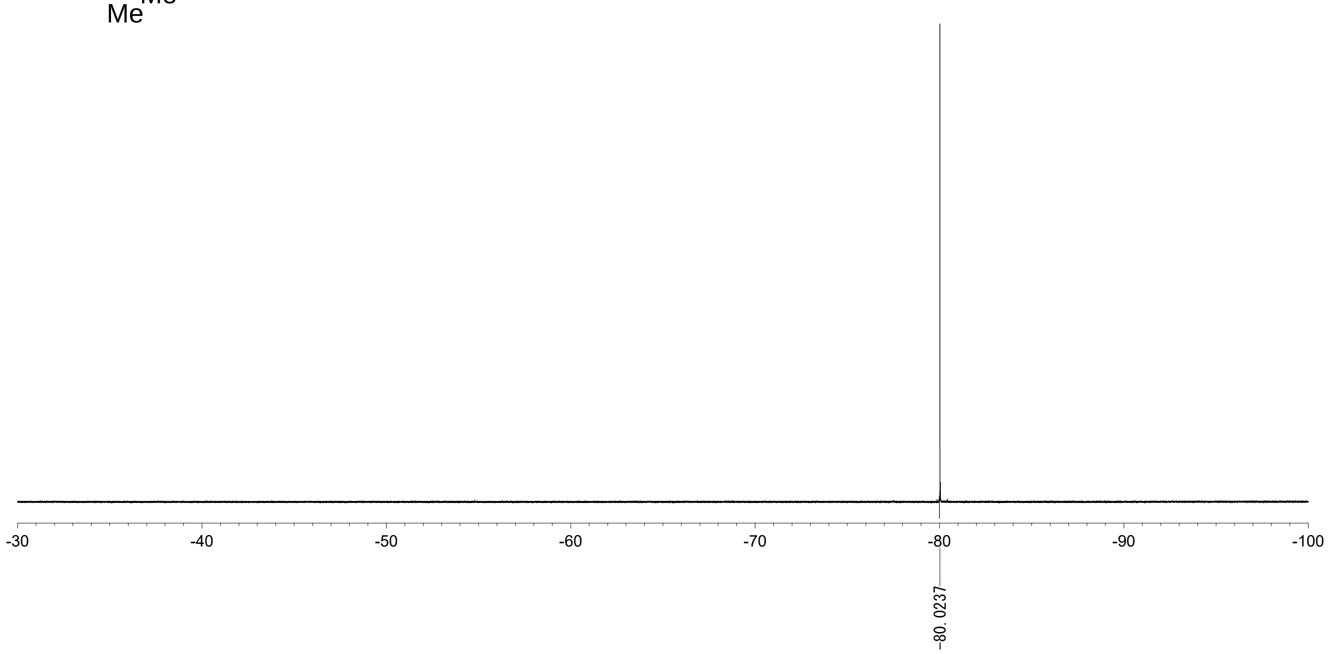

2-Methoxy-11,11-dimethyl-11*H*-dibenzo[*b*,*f*][1,4]iodaoxepin-5-ium trifluoromethanesulfonate (3ag)

^{1}H NMR (400 MHz, CD_3OD)

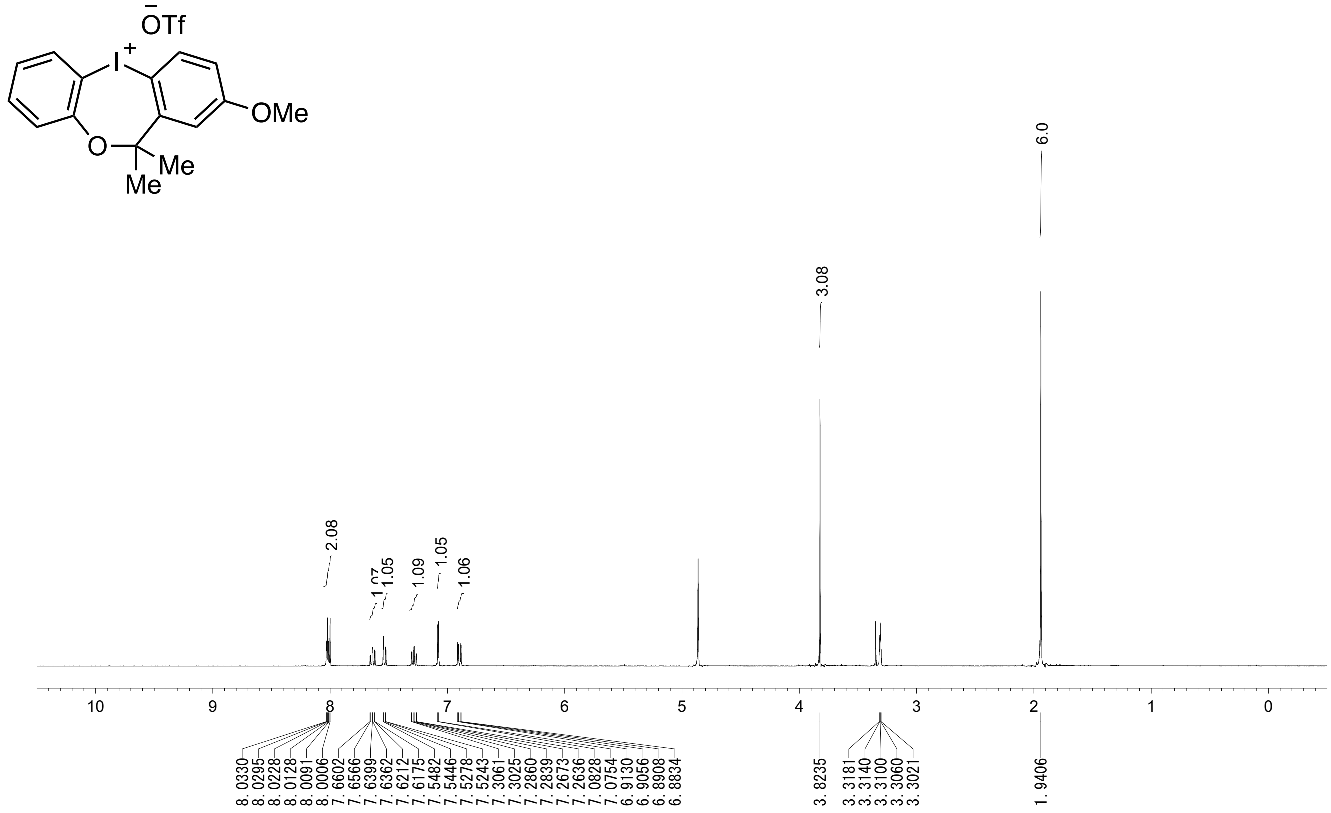

^{13}C{^{1}H} NMR (150 MHz, CD_3OD)

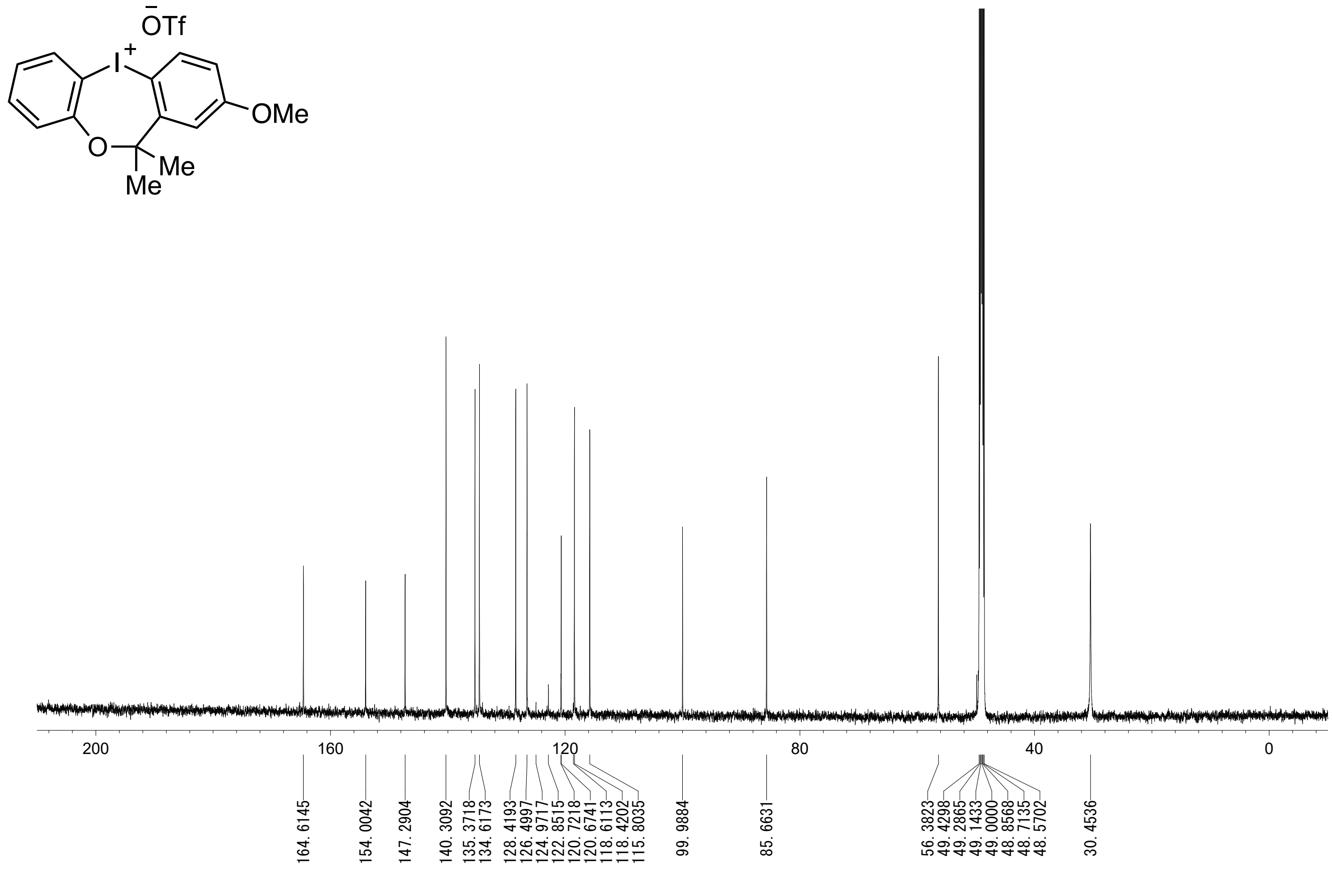

^{19}F NMR (376 MHz, CD_3OD)

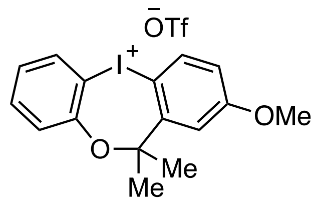

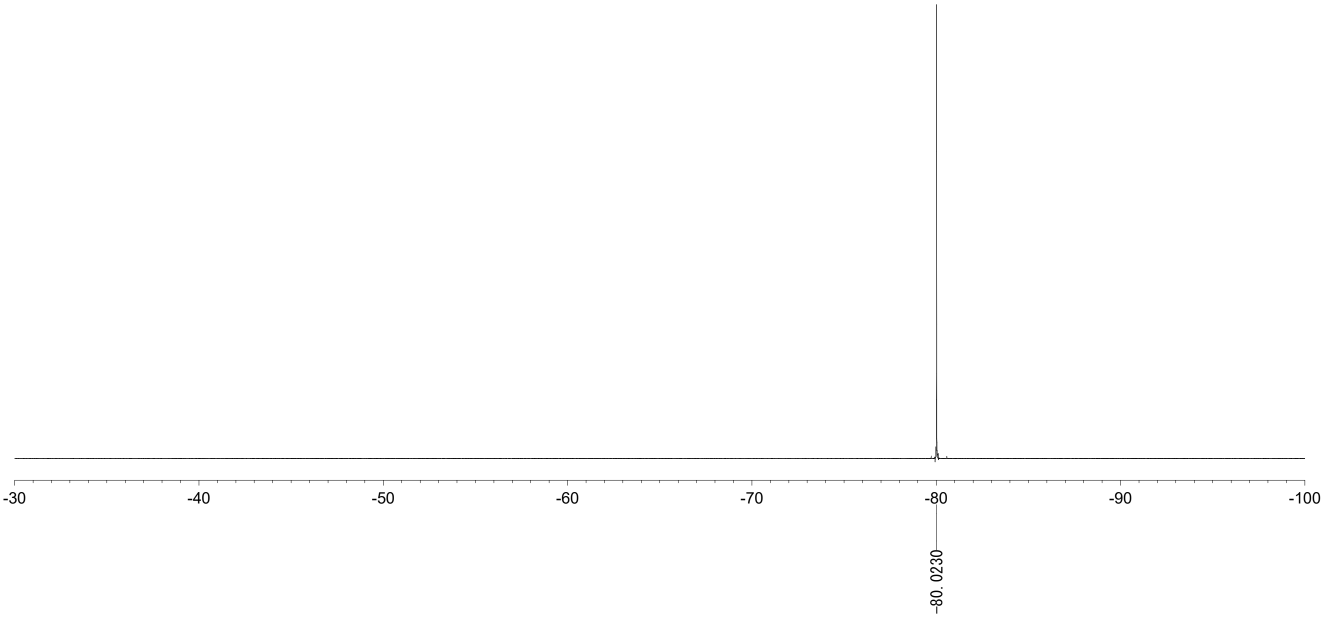

2-Bromo-11,11-dimethyl-11*H*-dibenzo[*b*,*f*][1,4]iodaoxepin-5-ium trifluoromethanesulfonate (3ah)
^{1}H NMR (600 MHz, CD_3OD)

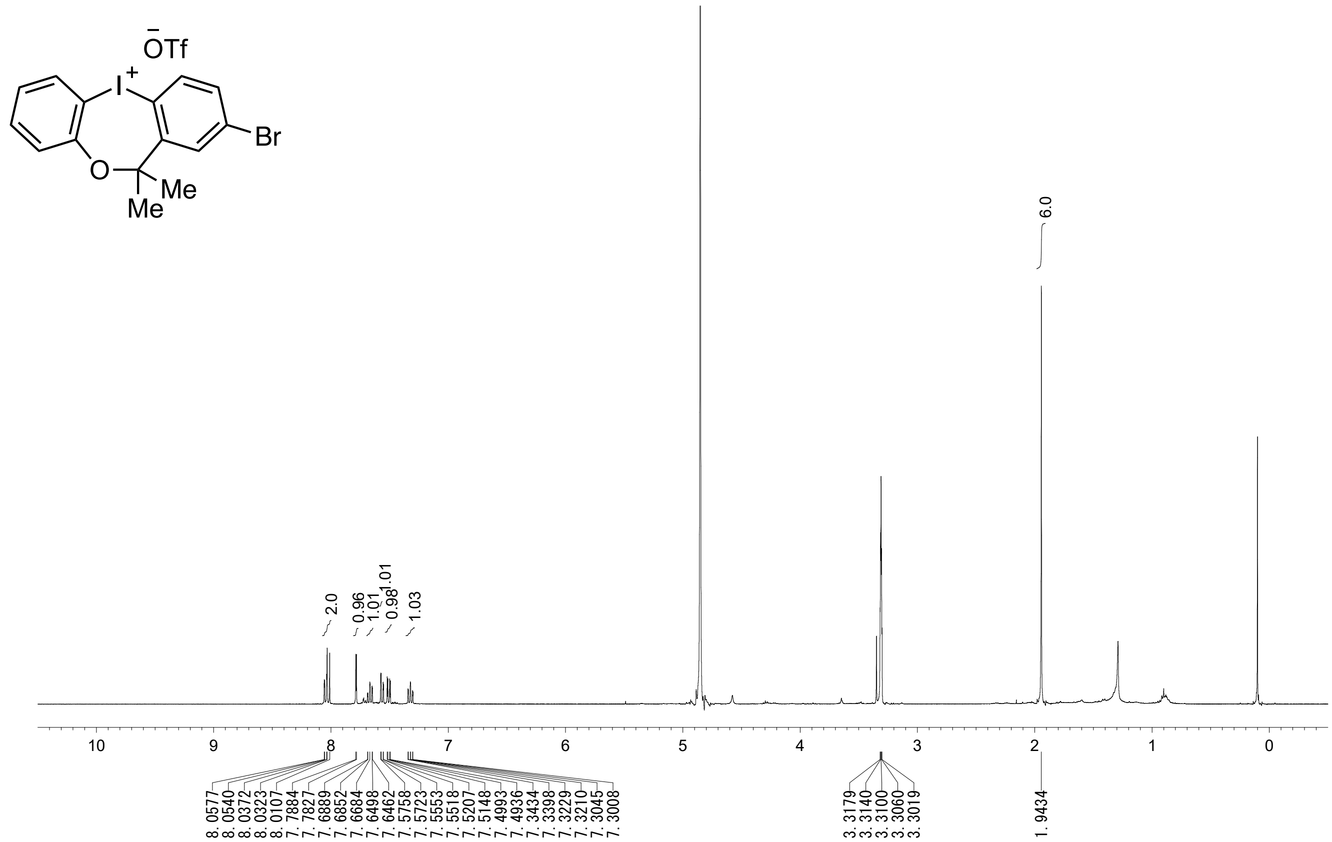

^{13}C{^{1}H} NMR (150 MHz, CD_3OD)

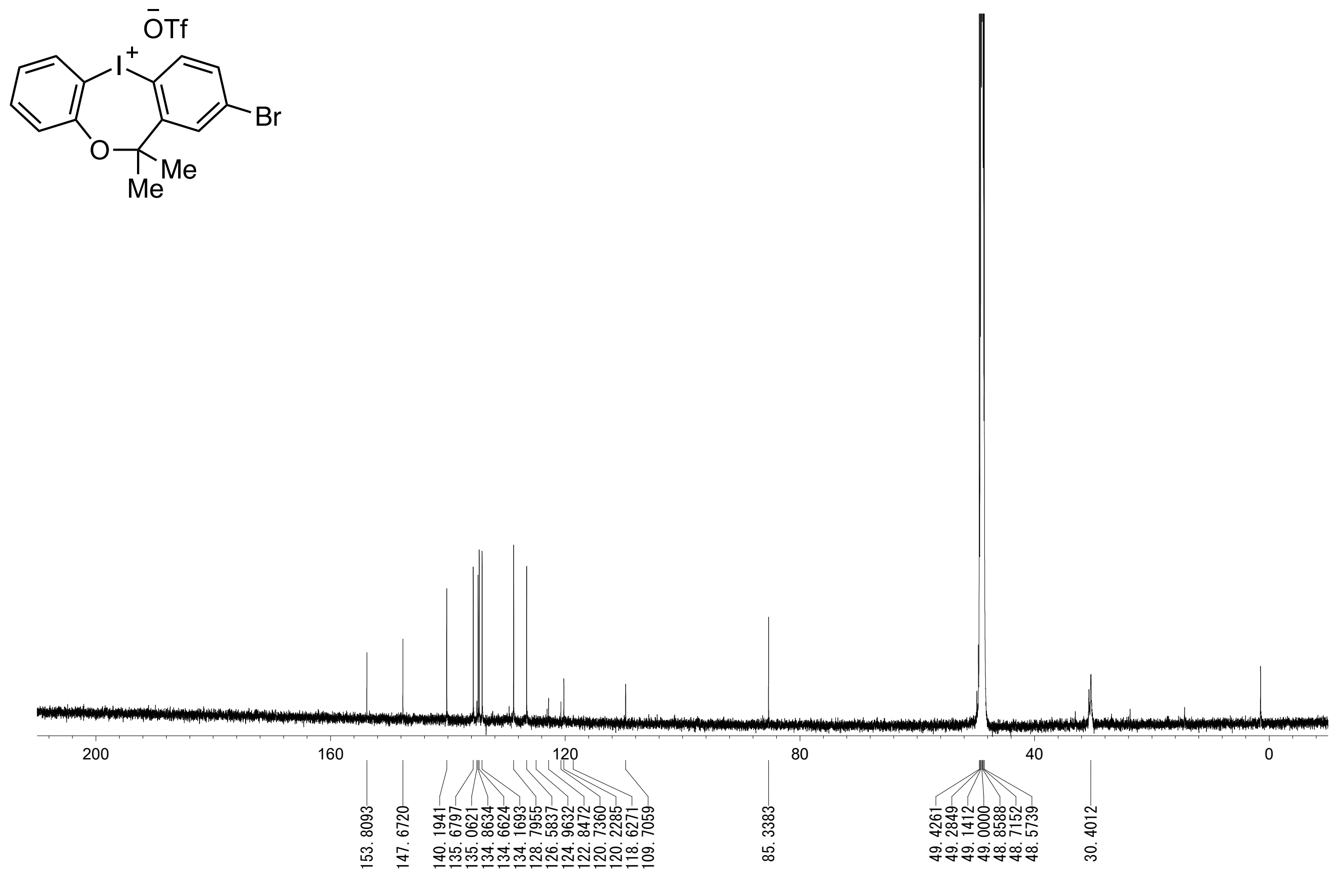

^{19}F NMR (376 MHz, CD_3OD)

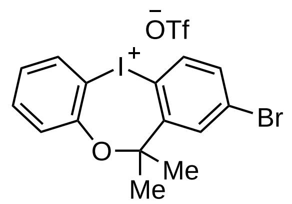

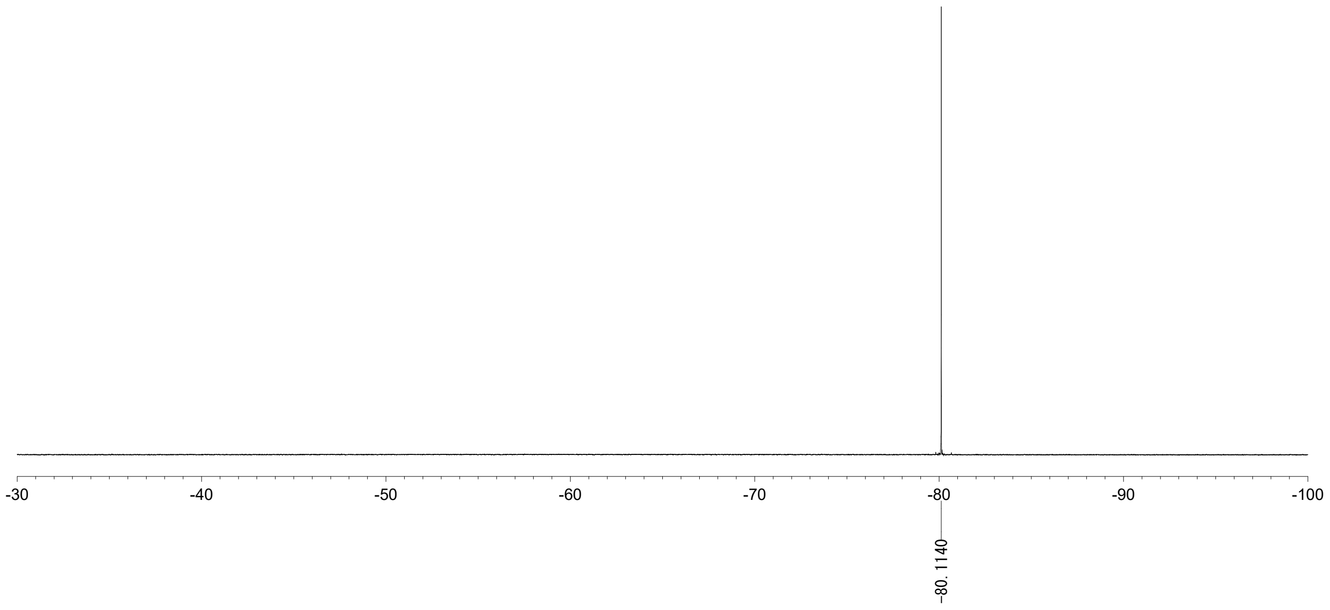

11,11-Dimethyl-5-(*p*-tolyl)-5,11-dihydrodibenzo[*b*,*e*][1,4]oxazepine (4)

^{1}H NMR (400 MHz, $CDCl_3$)

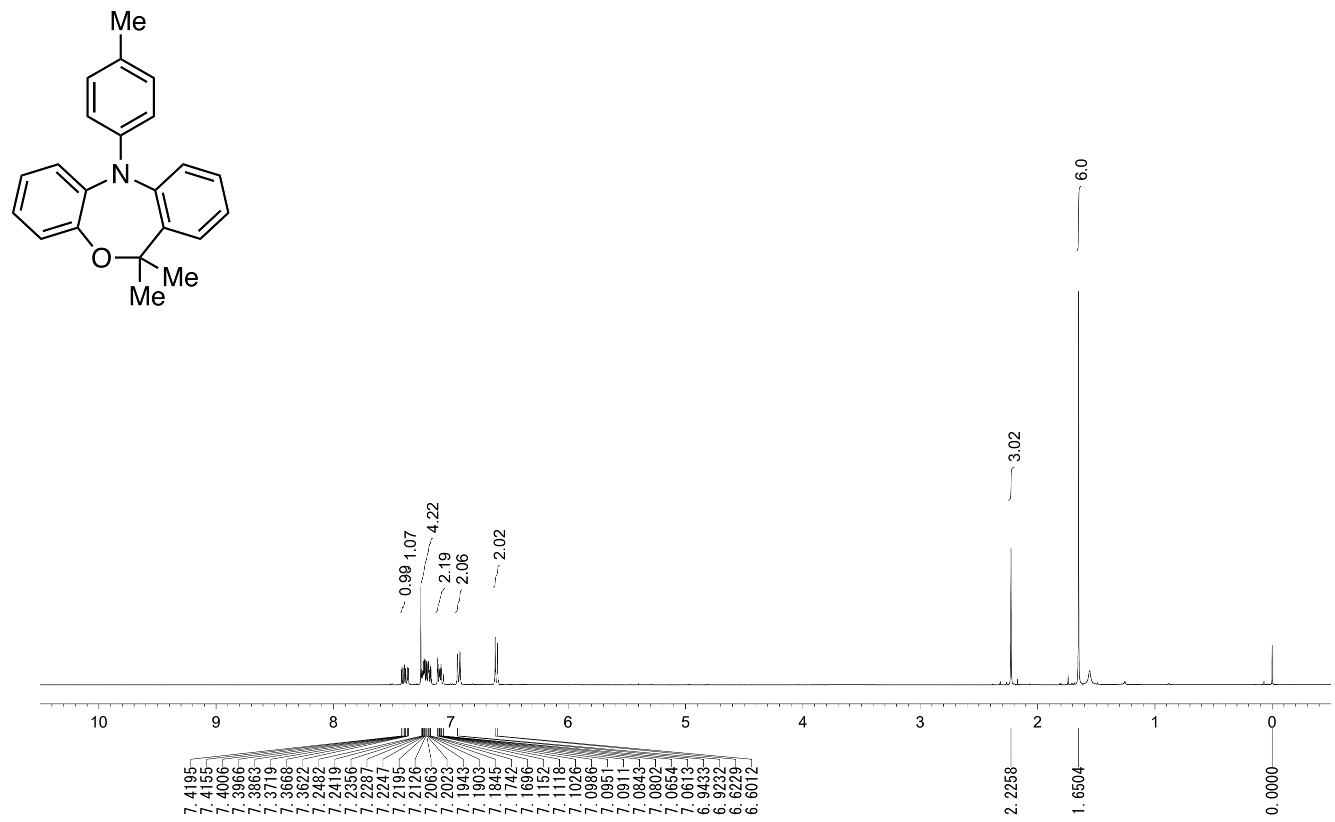

^{13}C{^{1}H} NMR (150 MHz, $CDCl_3$)

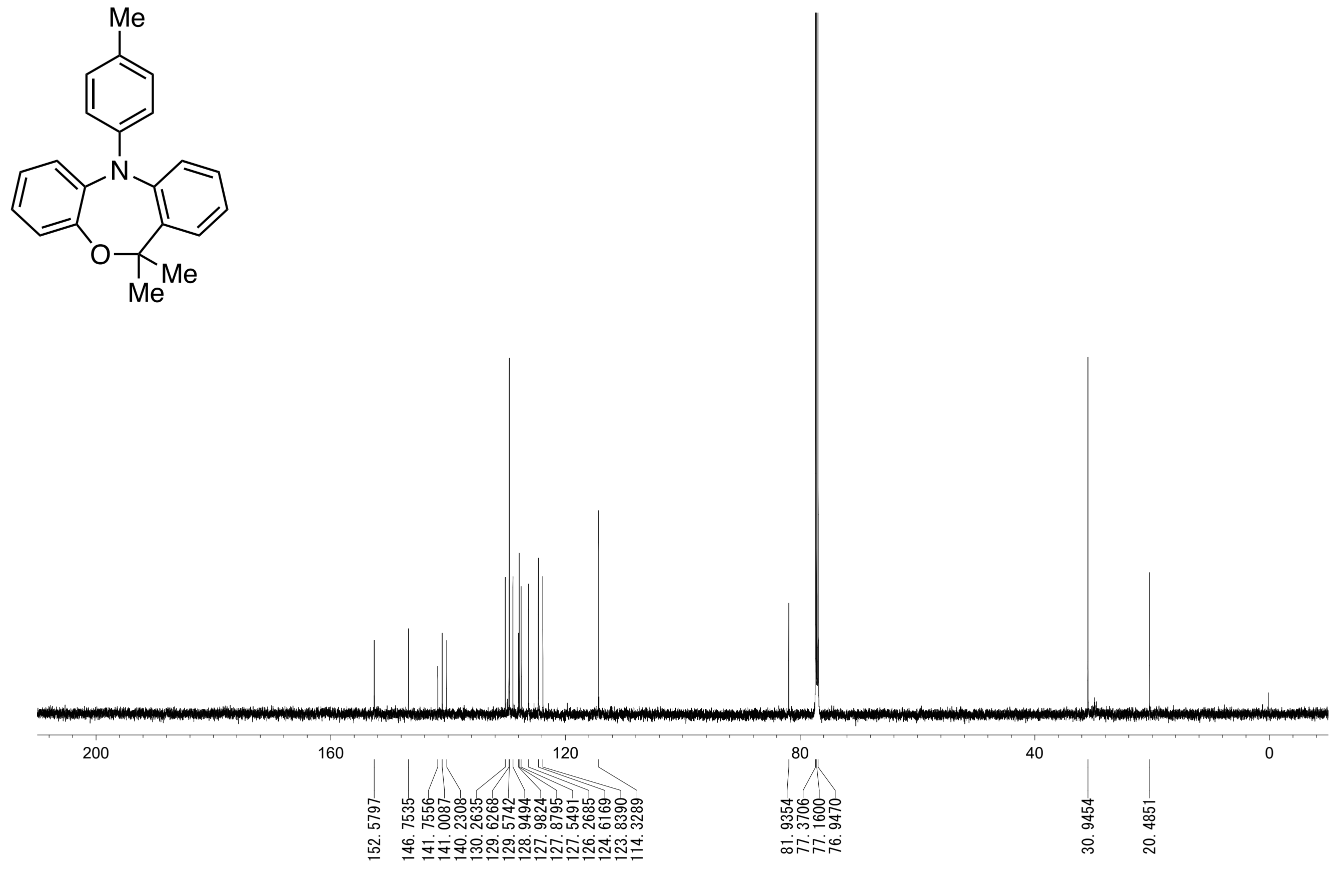

1-Iodo-2-(2-(2-iodophenoxy)propan-2-yl)benzene (I-5)
^{1}H NMR (600 MHz, $CDCl_3$)

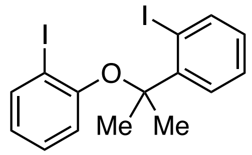

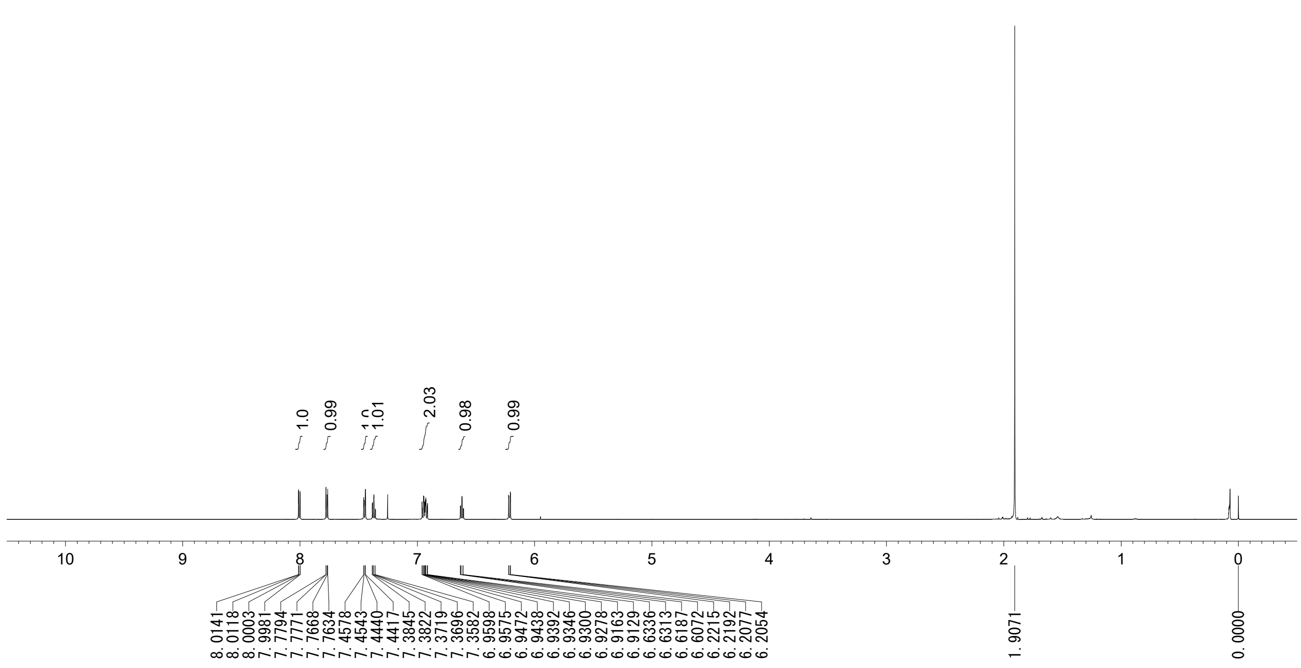

$^{13}C\{^{1}H\}$ NMR (150 MHz, $CDCl_3$)

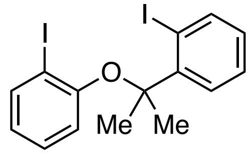

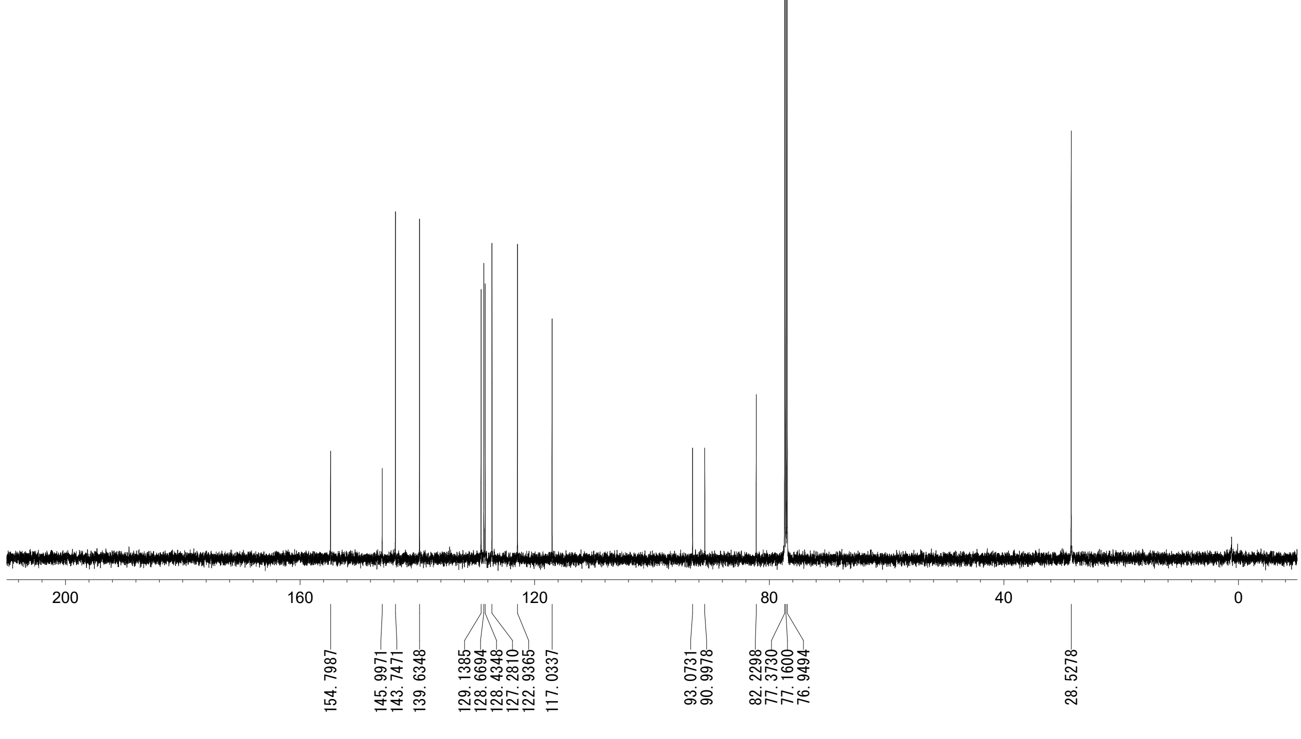

11,11-Dimethyl-5-phenyl-11*H*-dibenzo[*b*,*e*][1,4]oxaphosphepine 5-oxide (5)

^{1}H NMR (400 MHz, $CDCl_3$)

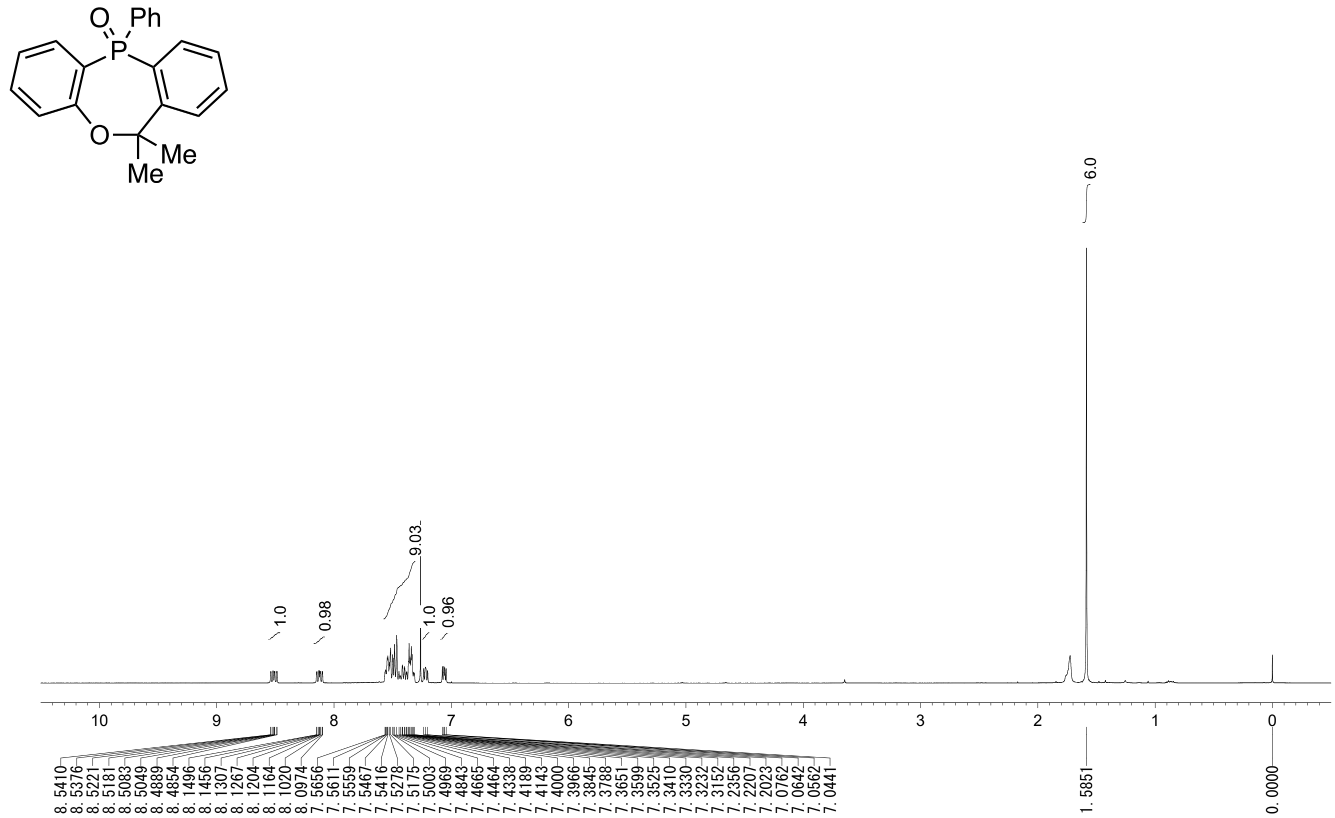

^{13}C{^{1}H} NMR (150 MHz, $CDCl_3$)

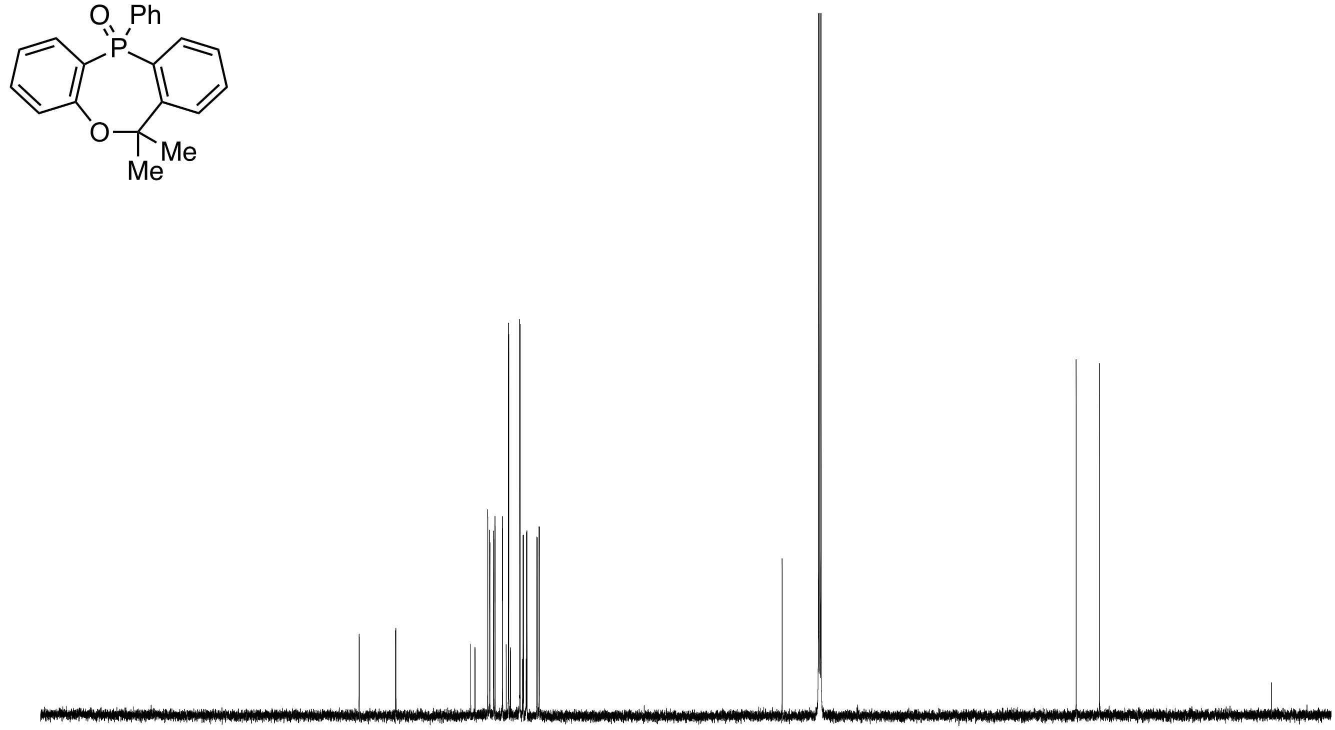

^{31}P NMR (162 MHz, $CDCl_3$)

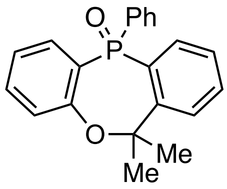

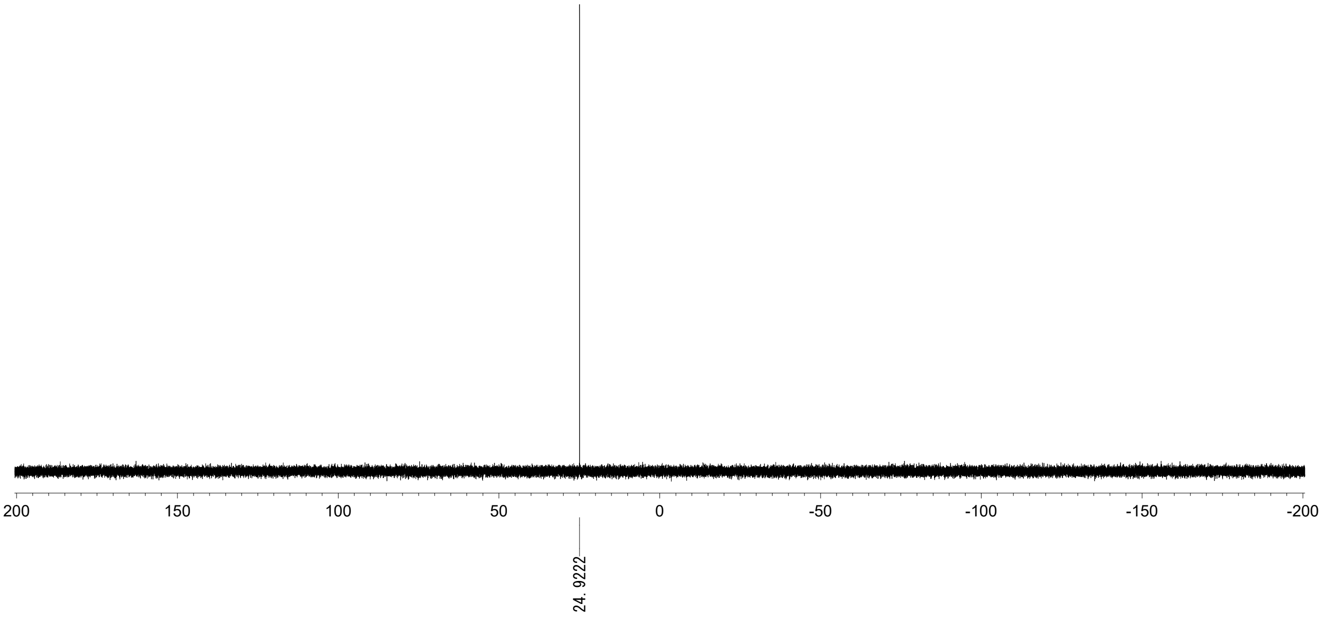

5,5,11,11-Tetramethyl-5,11-dihydrodibenzo[*b*,*e*][1,4]oxasilepine (6)

^{1}H NMR (600 MHz, $CDCl_3$)

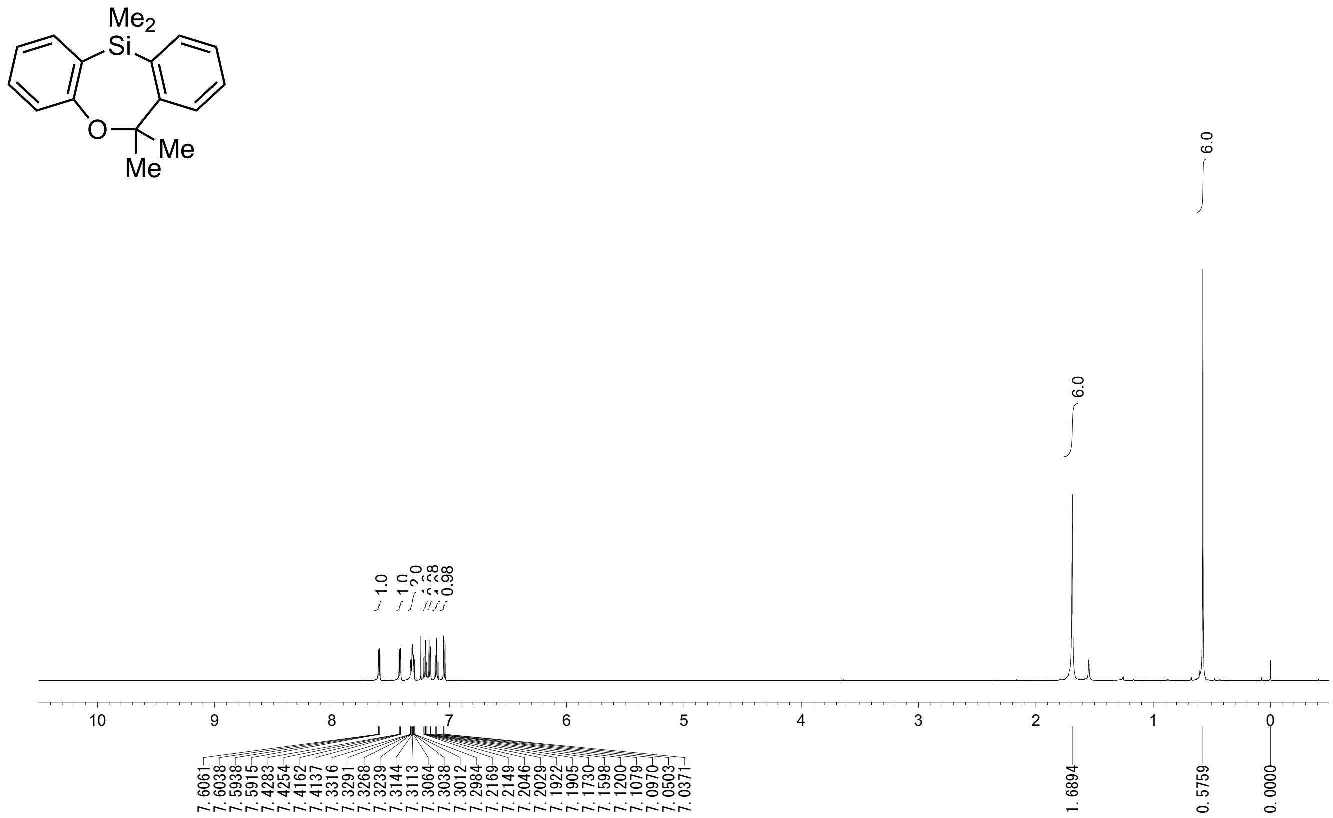

^{13}C{^{1}H} NMR (150 MHz, $CDCl_3$)

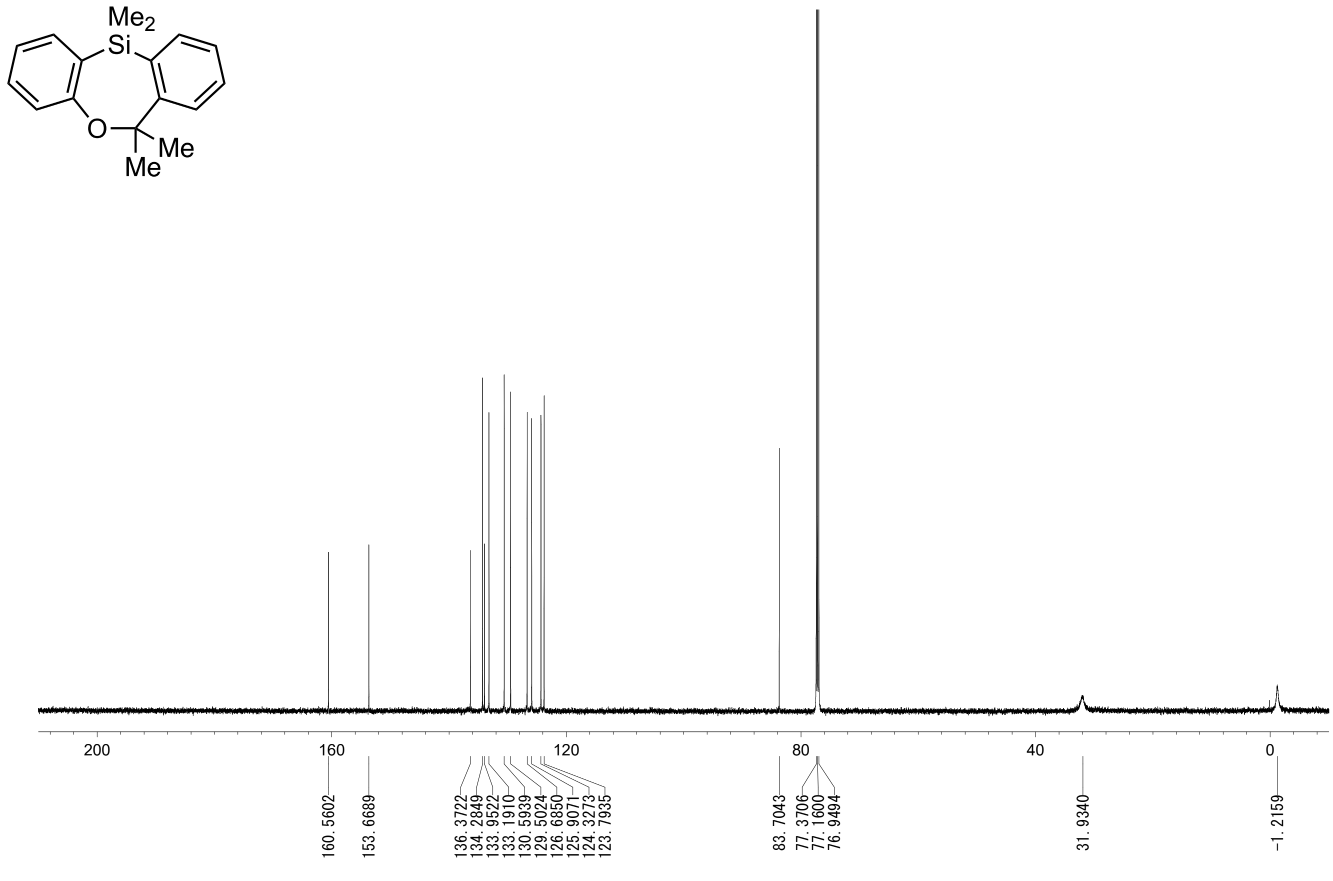

6,6-Dimethyl-6*H*-benzo[*c*]chromene (7)

^{1}H NMR (600 MHz, $CDCl_3$)

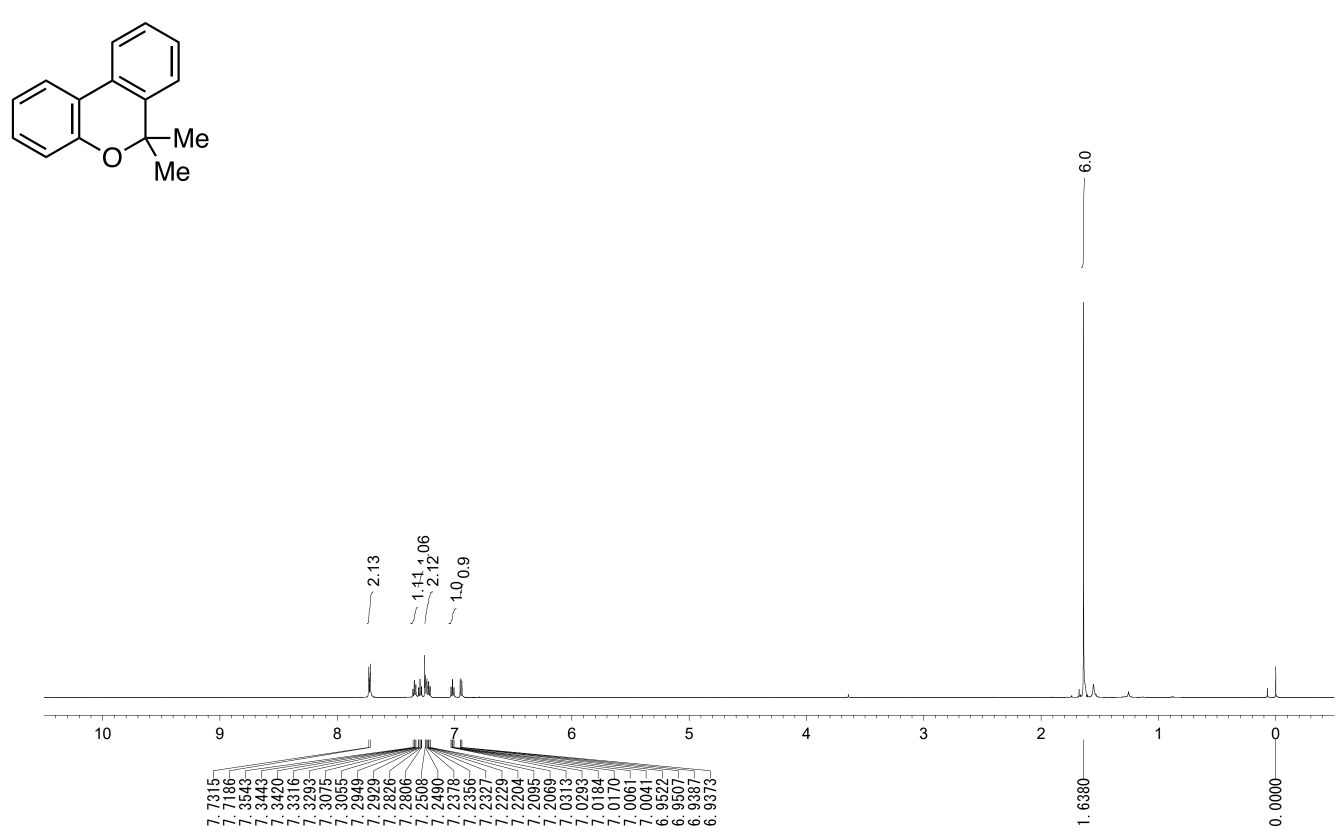

^{13}C{^{1}H} NMR (150 MHz, $CDCl_3$)

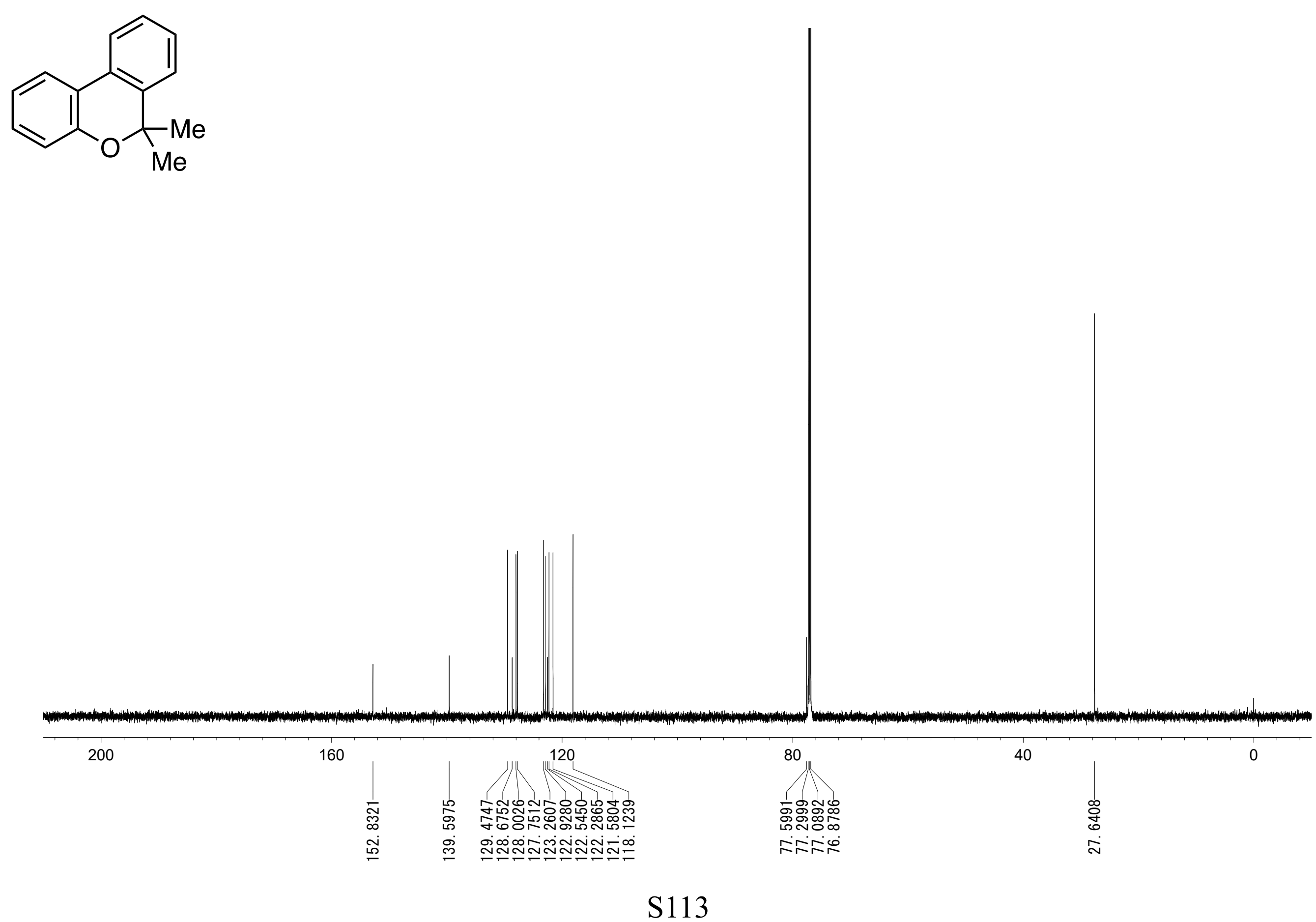

1-Methoxy-6,6-dimethyl-6*H*-benzo[*c*]chromene (8)
^{1}H NMR (400 MHz, $CDCl_3$)

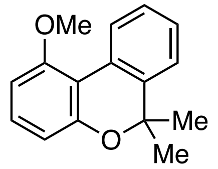

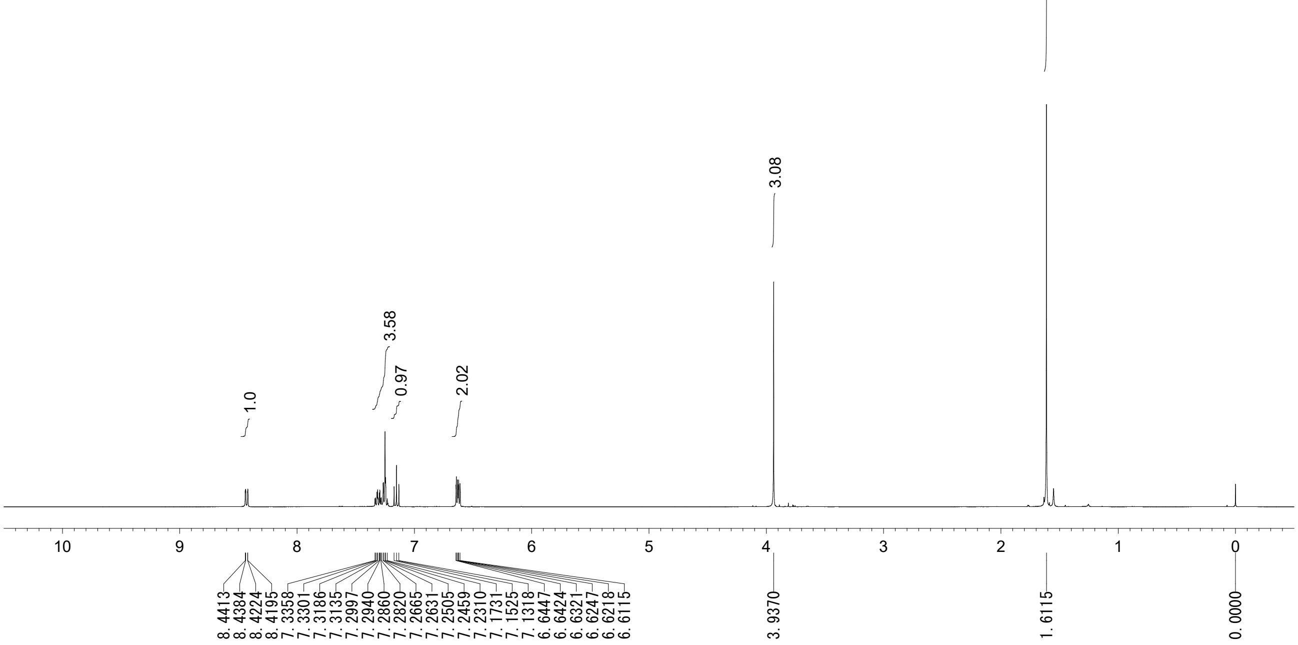

$^{13}C\{^1H\}$ NMR (150 MHz, $CDCl_3$)

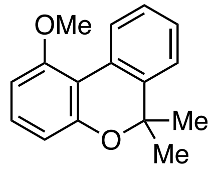

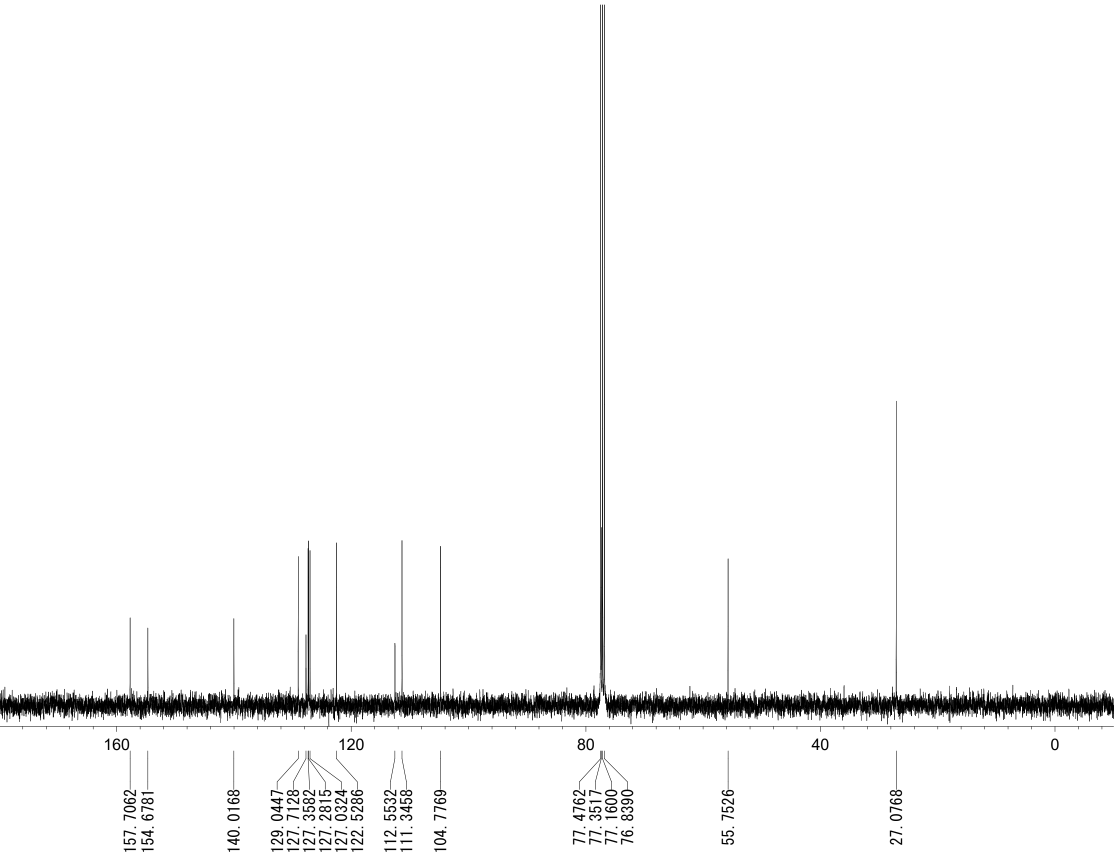

2-(Methoxymethoxy)-5-pentylphenol (S-6)
^{1}H NMR (400 MHz, $CDCl_3$)

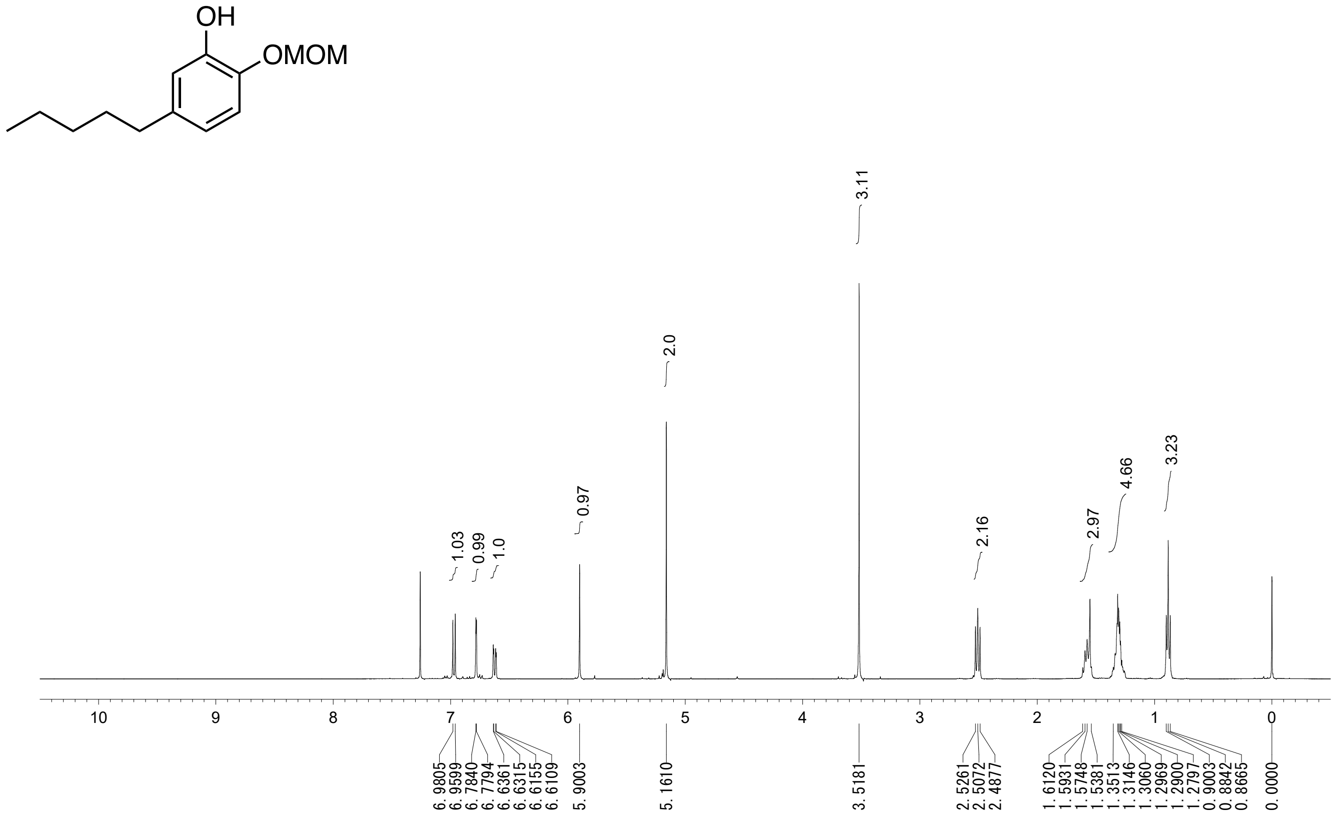

^{13}C{^{1}H} NMR (150 MHz, $CDCl_3$)

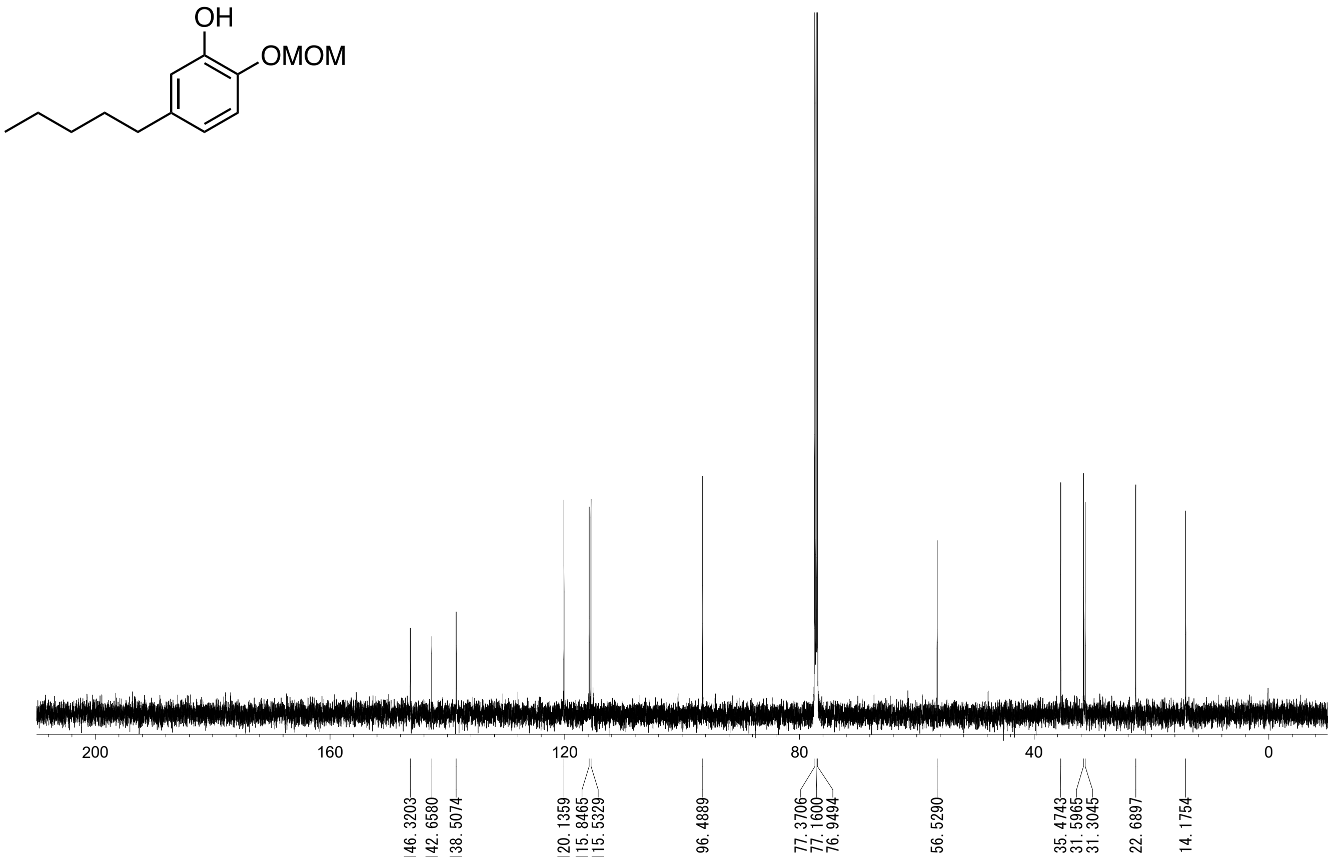

2-Methoxy-1-(methoxymethoxy)-4-pentylbenzene (S-7)

^{1}H NMR (600 MHz, $CDCl_3$)

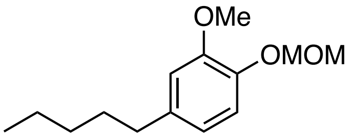

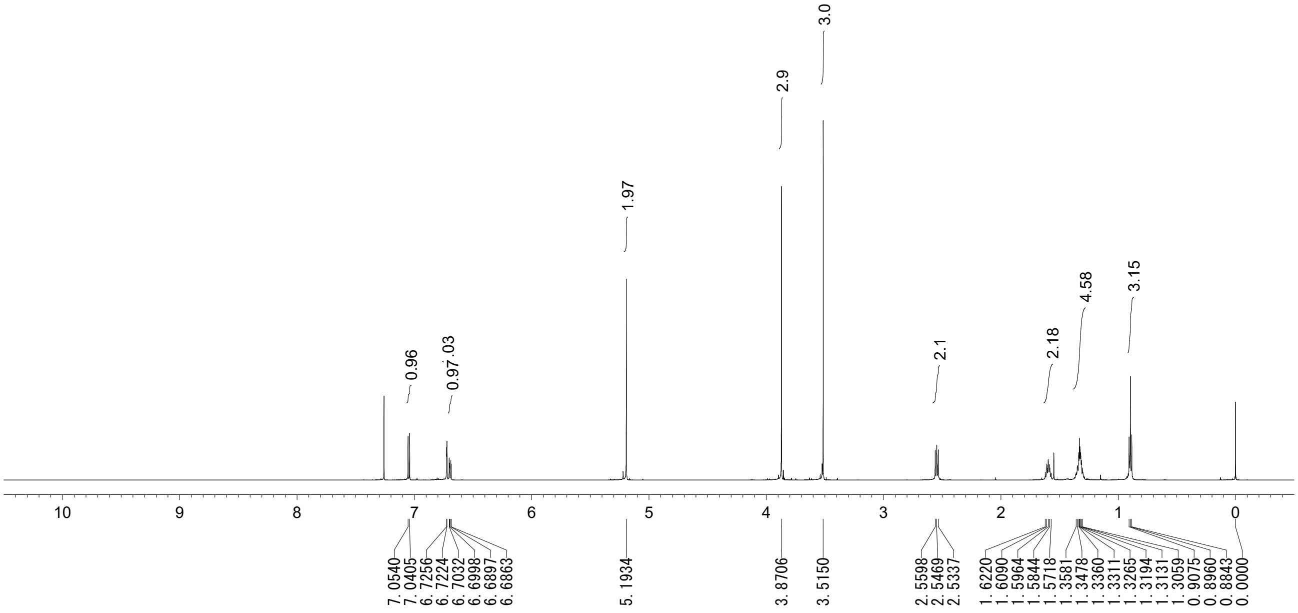

^{13}C{^{1}H} NMR (150 MHz, $CDCl_3$)

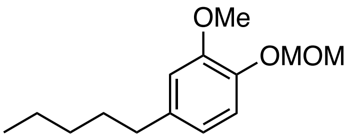

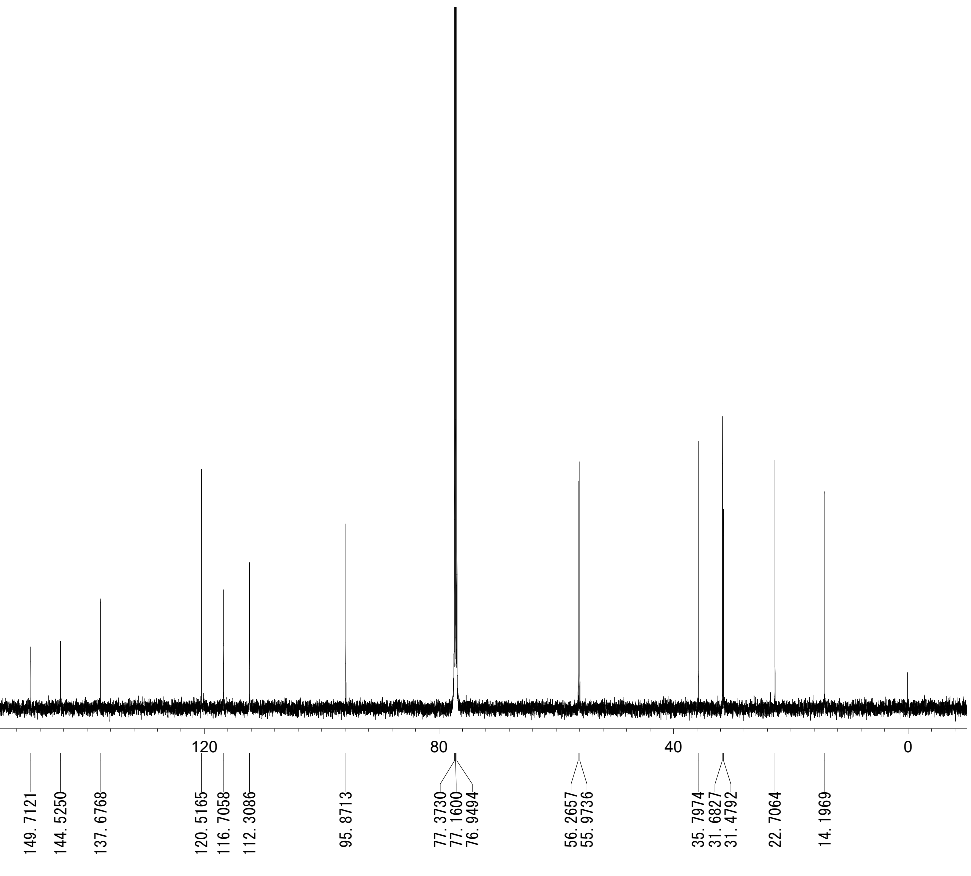

2-Methoxy-4-pentylphenol (S-8)

^{1}H NMR (400 MHz, $CDCl_3$)

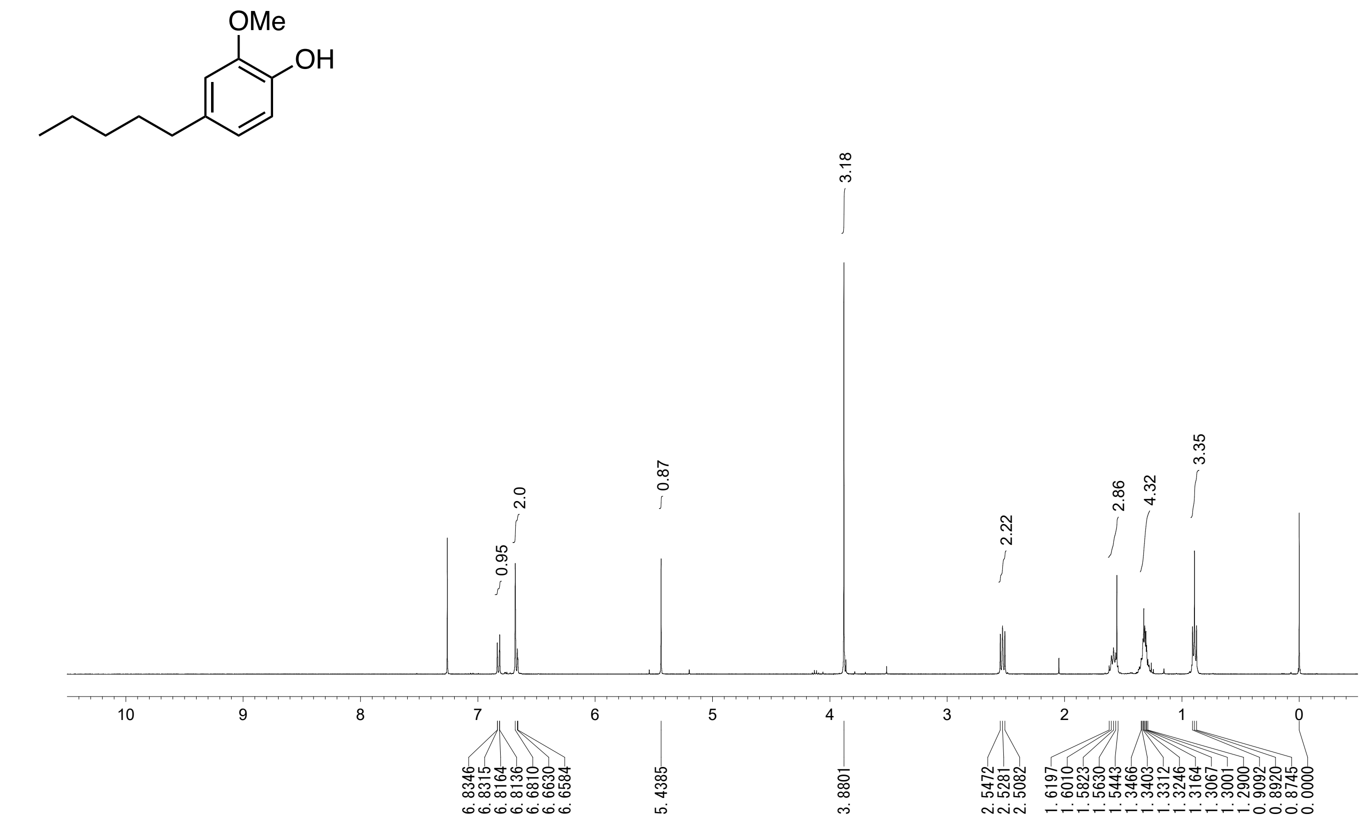

^{13}C{^{1}H} NMR (150 MHz, $CDCl_3$)

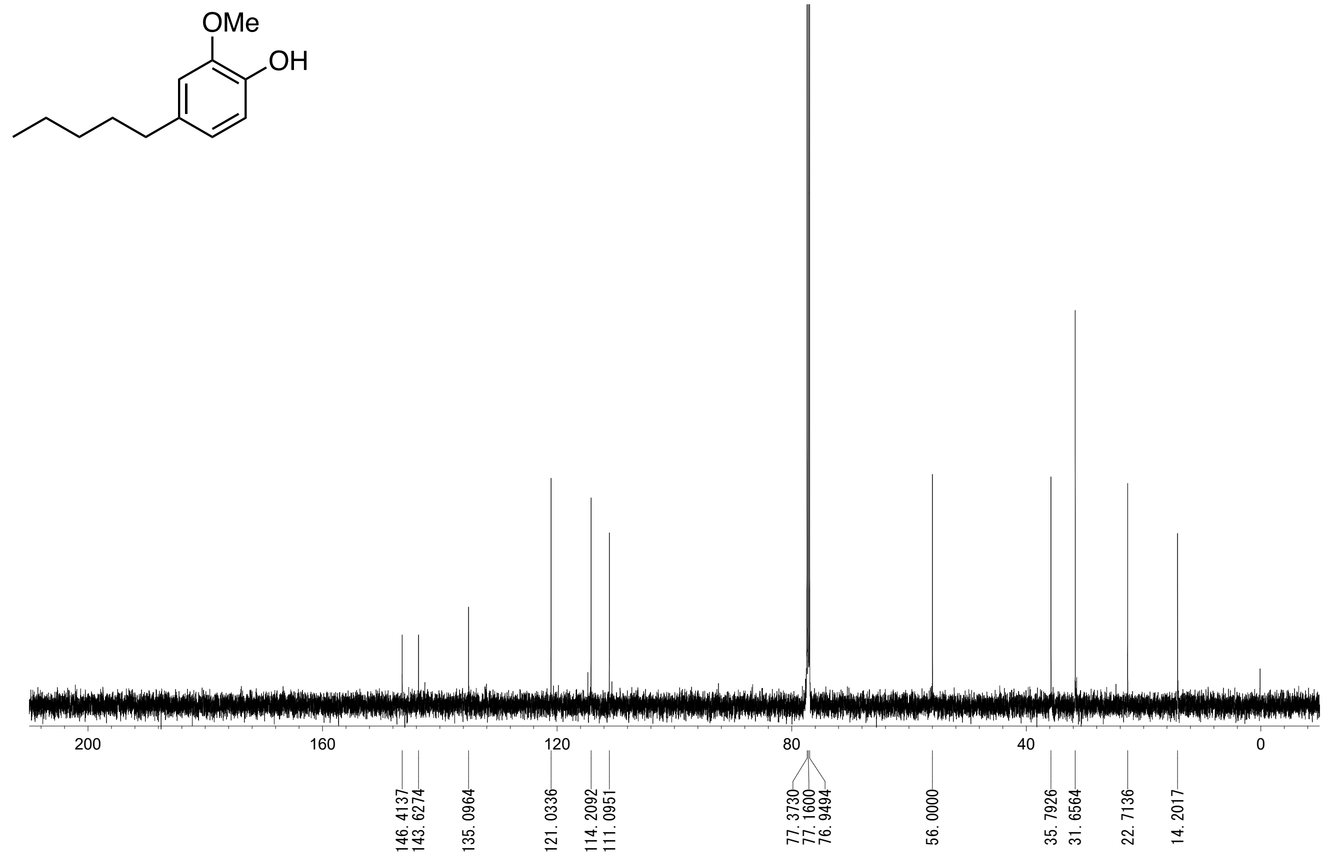

2-Bromo-6-methoxy-4-pentylphenol (S-9)
^{1}H NMR (400 MHz, $CDCl_3$)

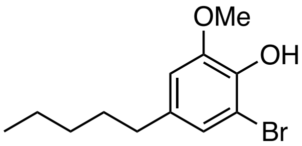

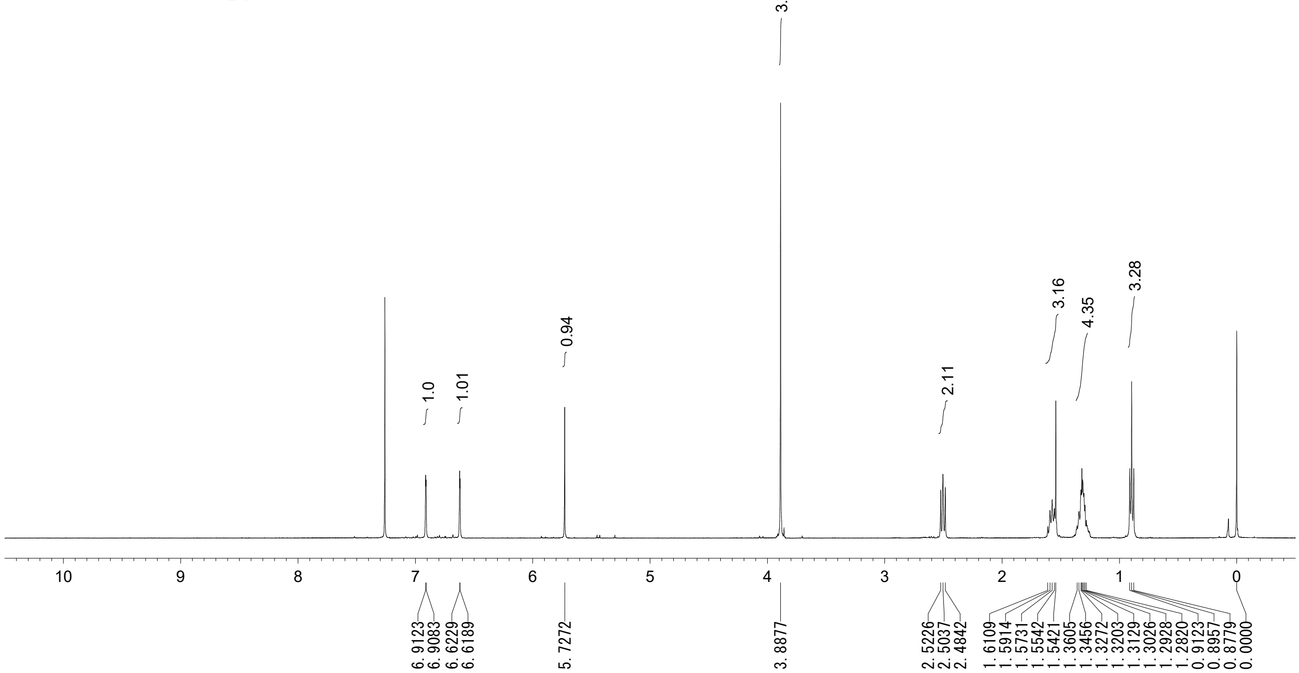

^{13}C{^{1}H} NMR (101 MHz, $CDCl_3$)

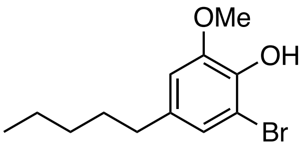

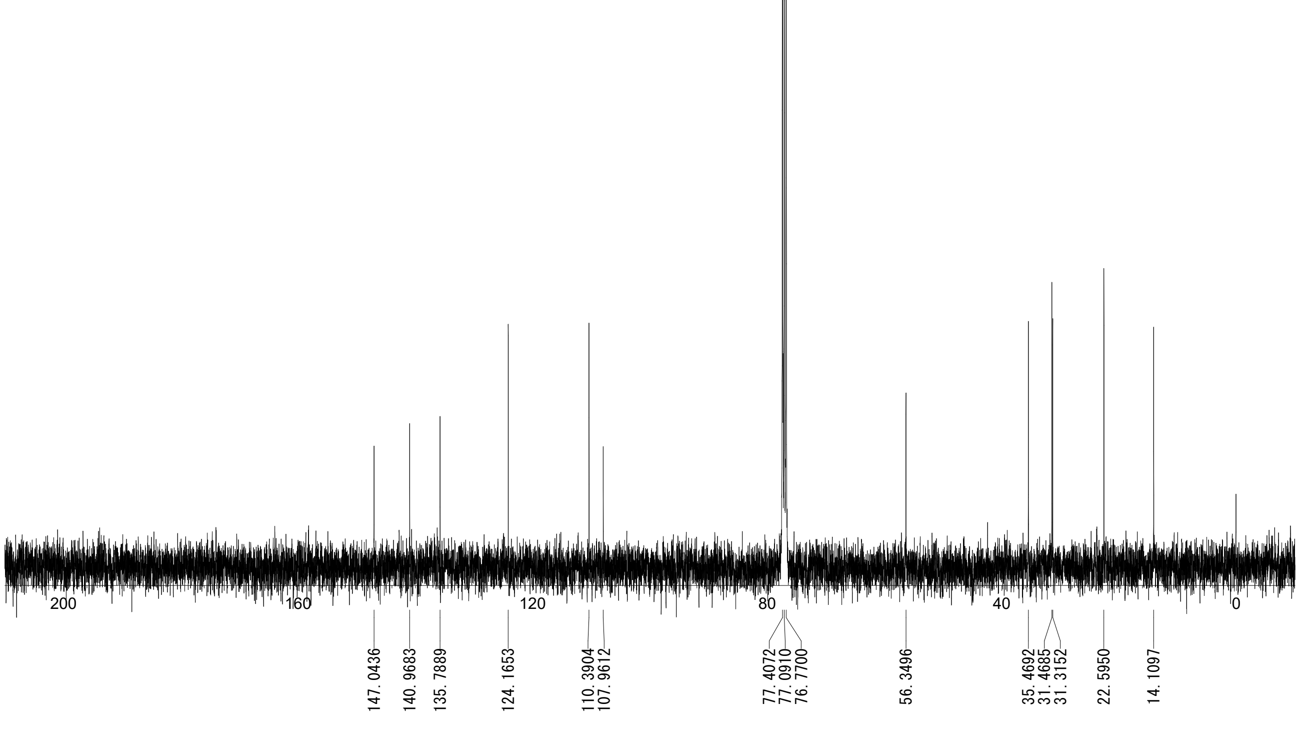

2-Methoxy-4-pentyl-6-(trimethylsilyl)phenyl trifluoromethanesulfonate (1l):

^{1}H NMR (600 MHz, $CDCl_3$)

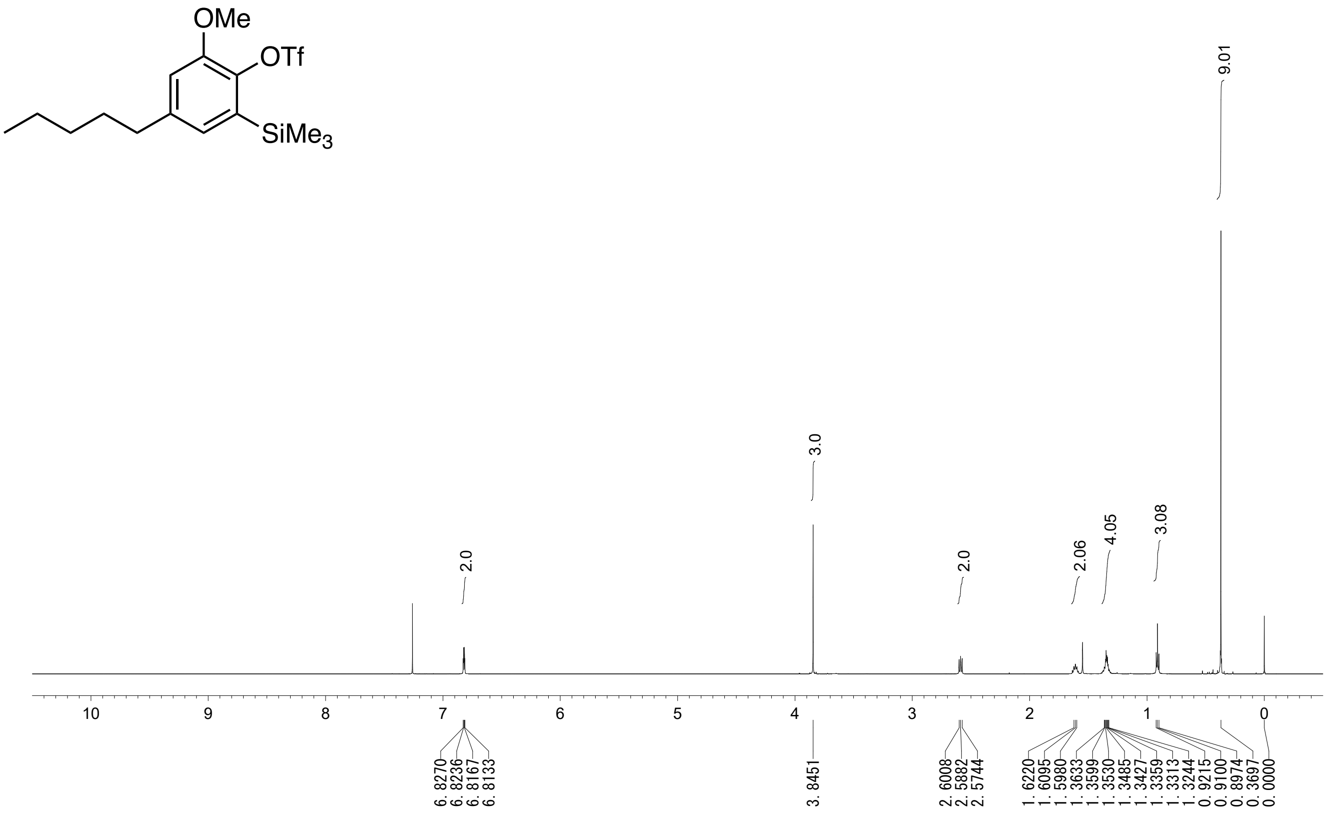

^{13}C{^{1}H} NMR (150 MHz, $CDCl_3$)

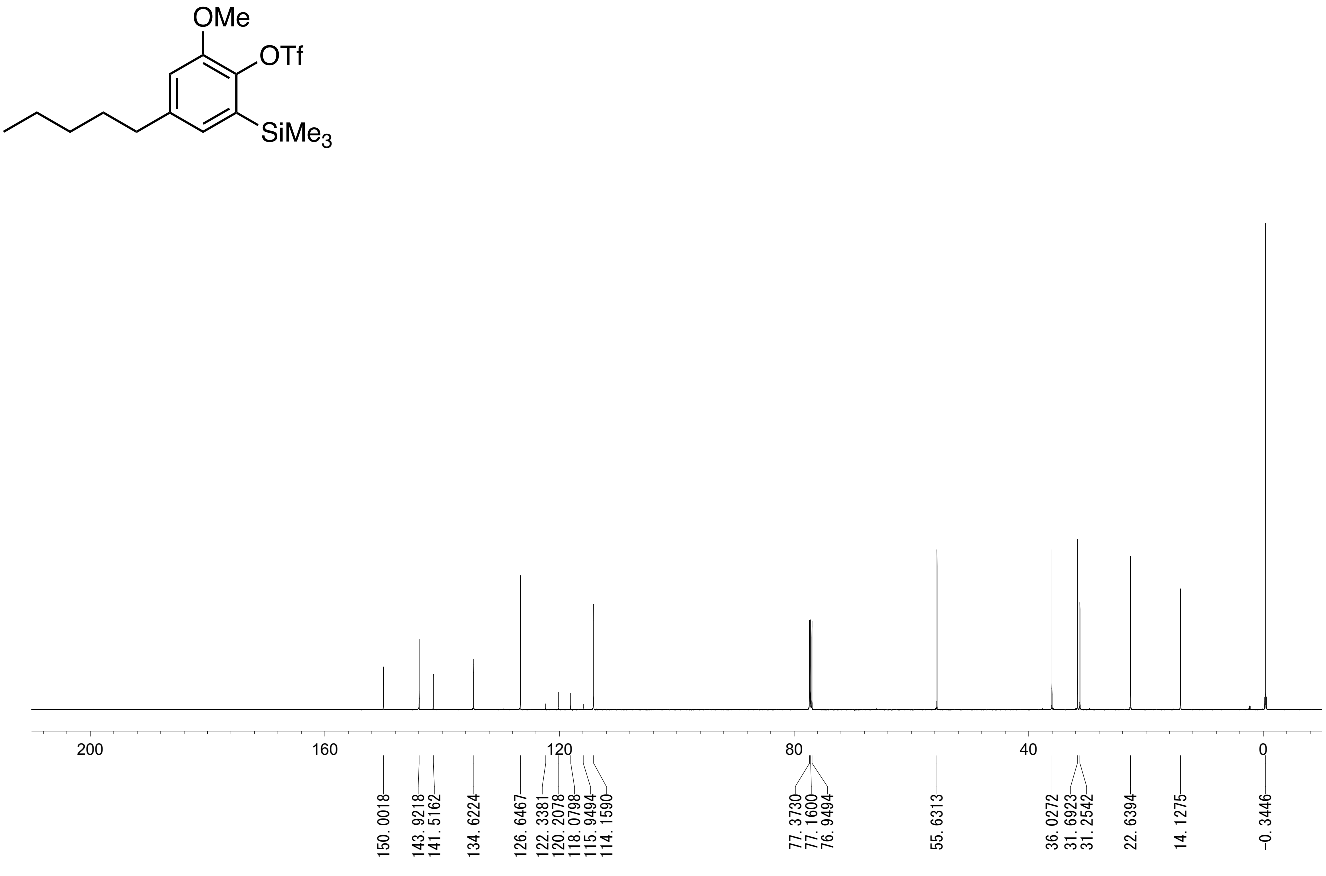

^{19}F NMR (376 MHz, CD_3OD)

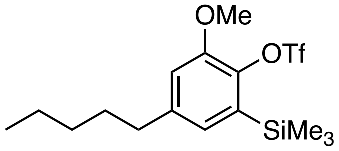

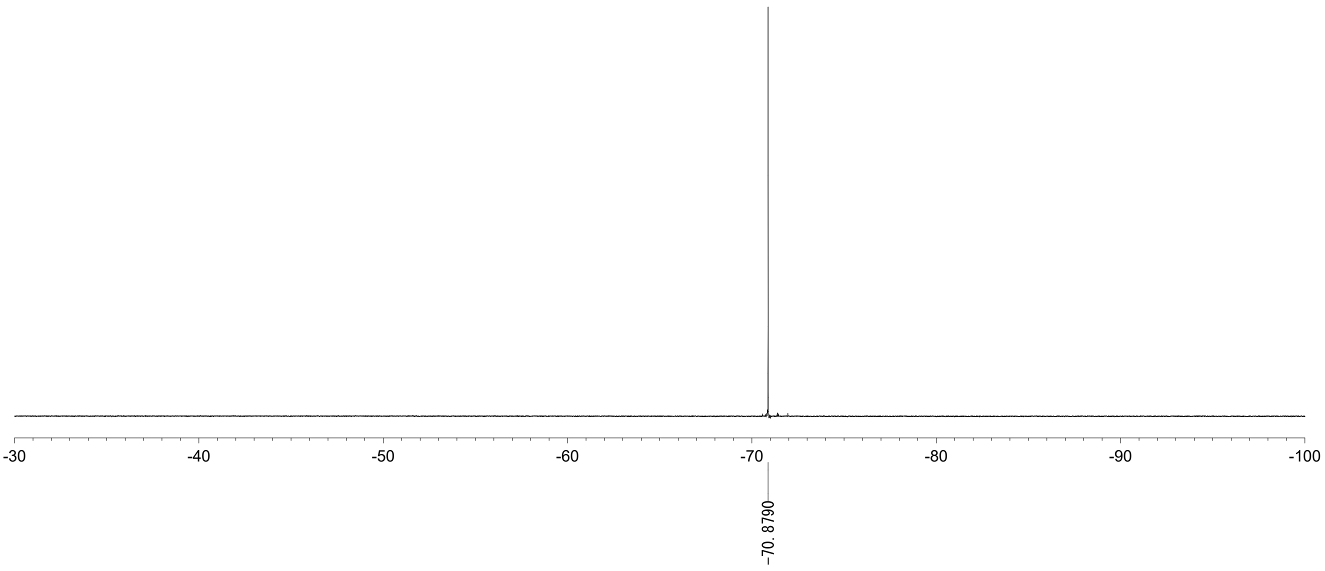

6-Methoxy-4,11,11-trimethyl-8-pentyl-11*H*-dibenzo[*b*,*f*][1,4]iodaoxepin-5-ium trifluoromethanesulfonate (3li)

^{1}H NMR (400 MHz, CD_3OD)

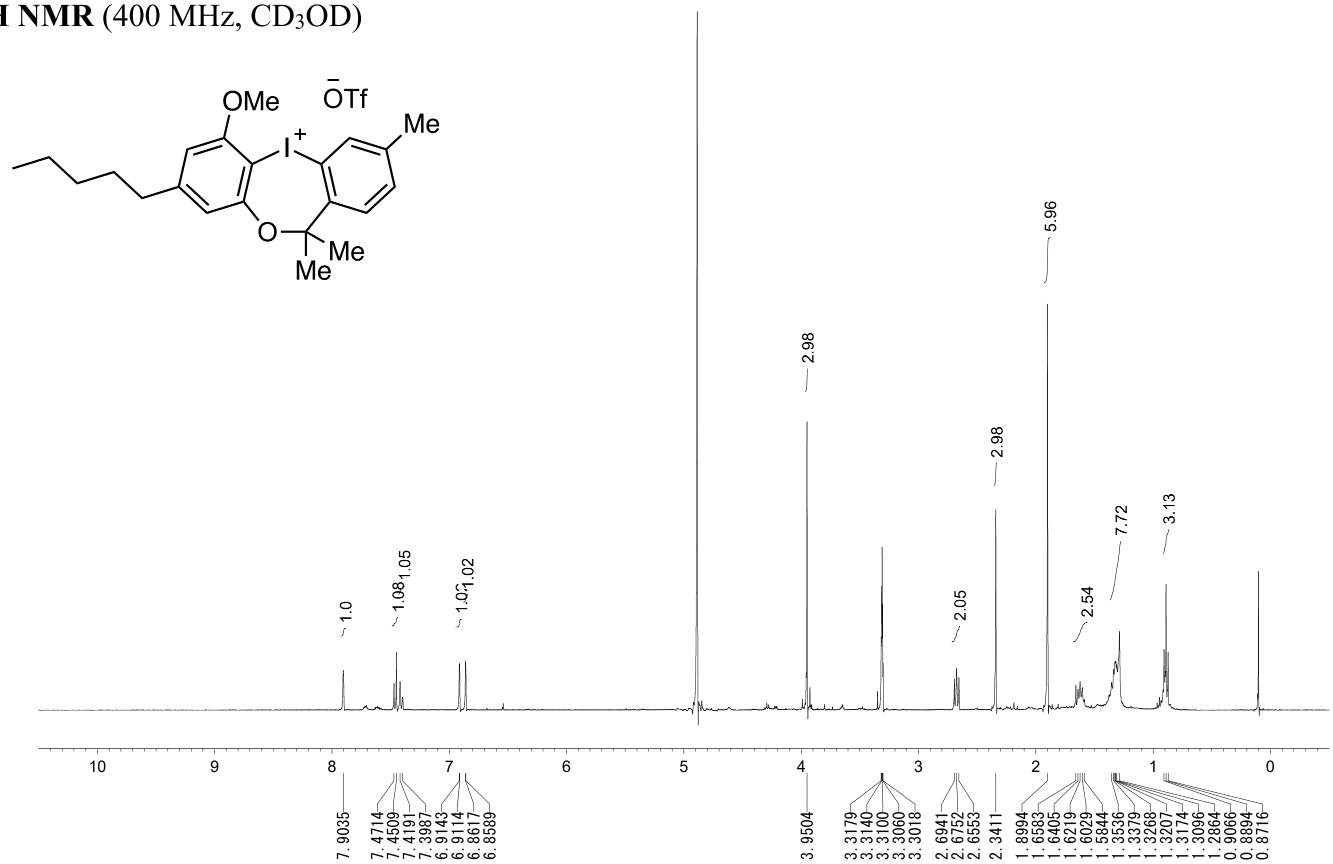

^{13}C{^{1}H} NMR (150 MHz, CD_3OD)

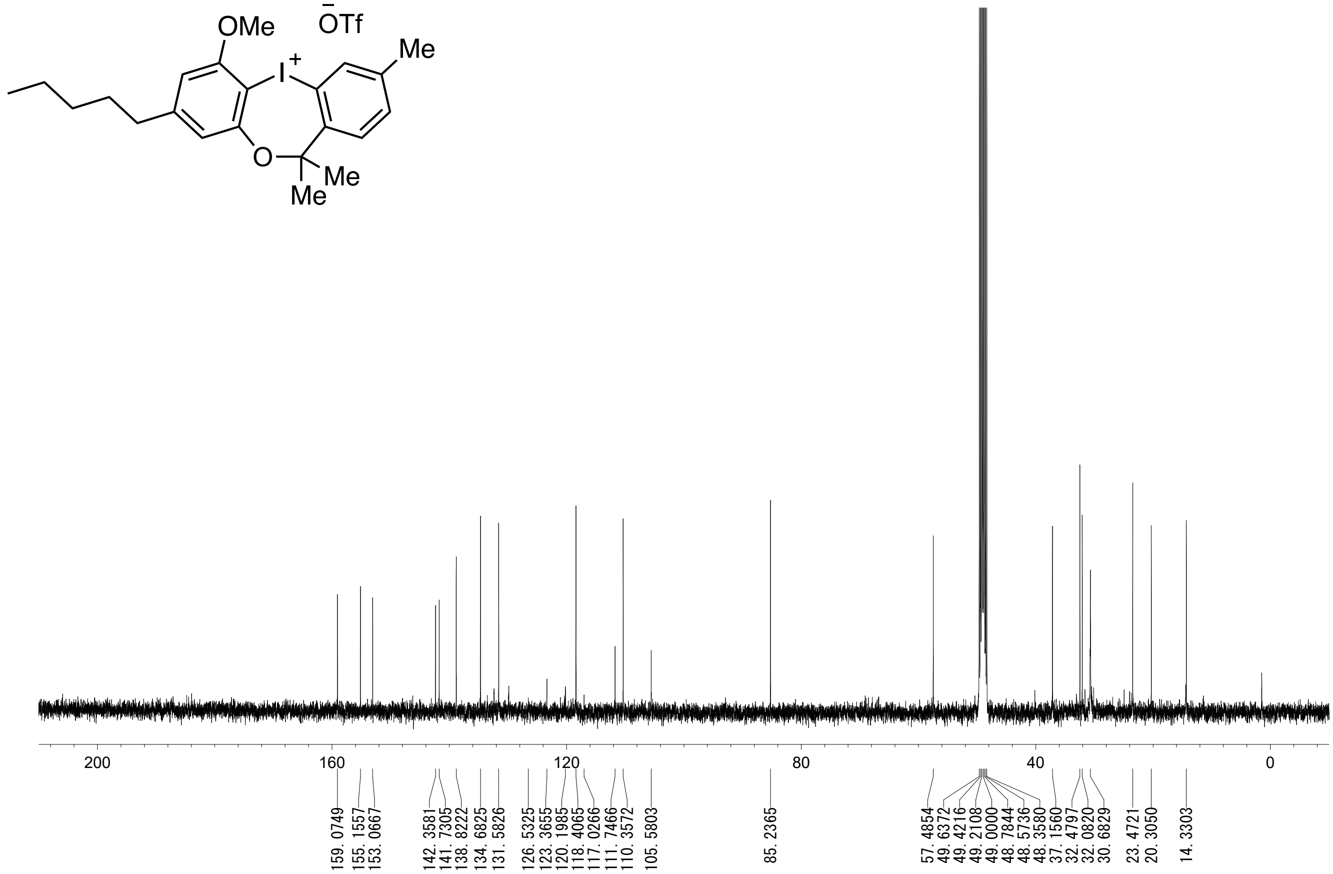

^{19}F NMR (376 MHz, CD_3OD)

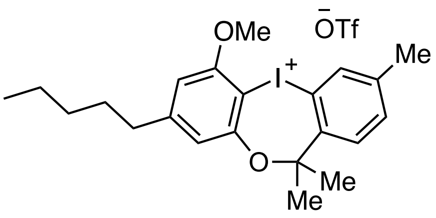

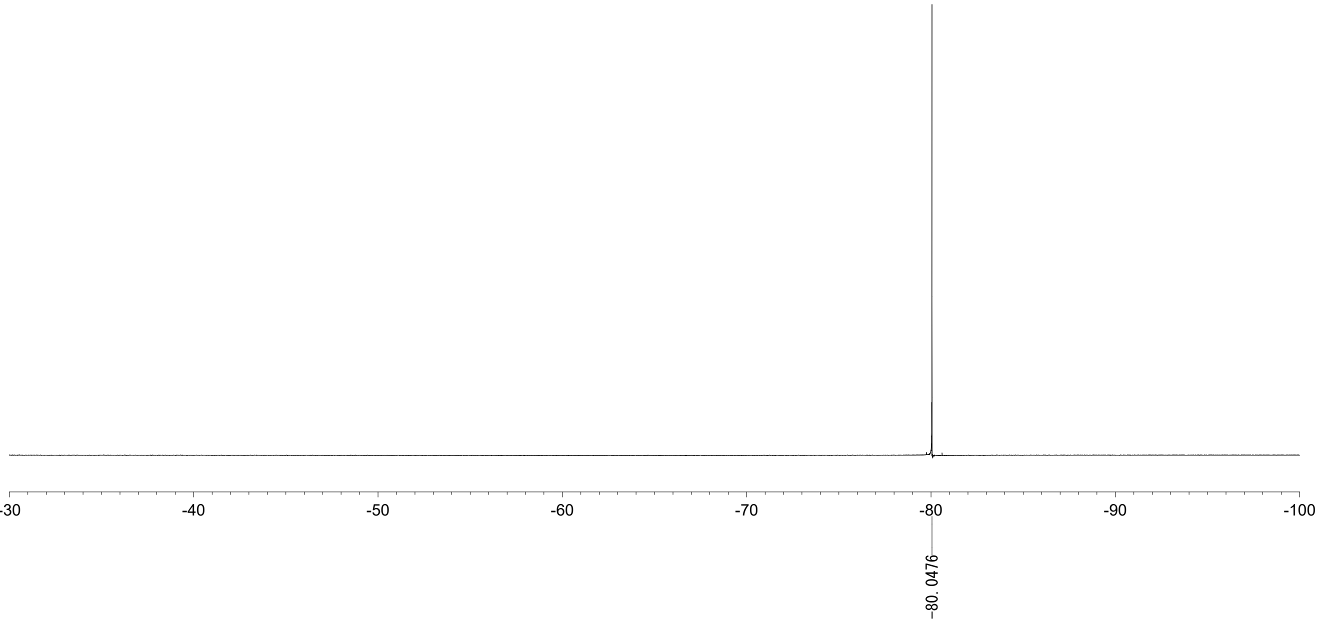

1-Methoxy-6,6,9-trimethyl-3-pentyl-6H-benzo[c]chromene (Cannabinol methyl ether)
^{1}H NMR (400 MHz, $CDCl_3$)

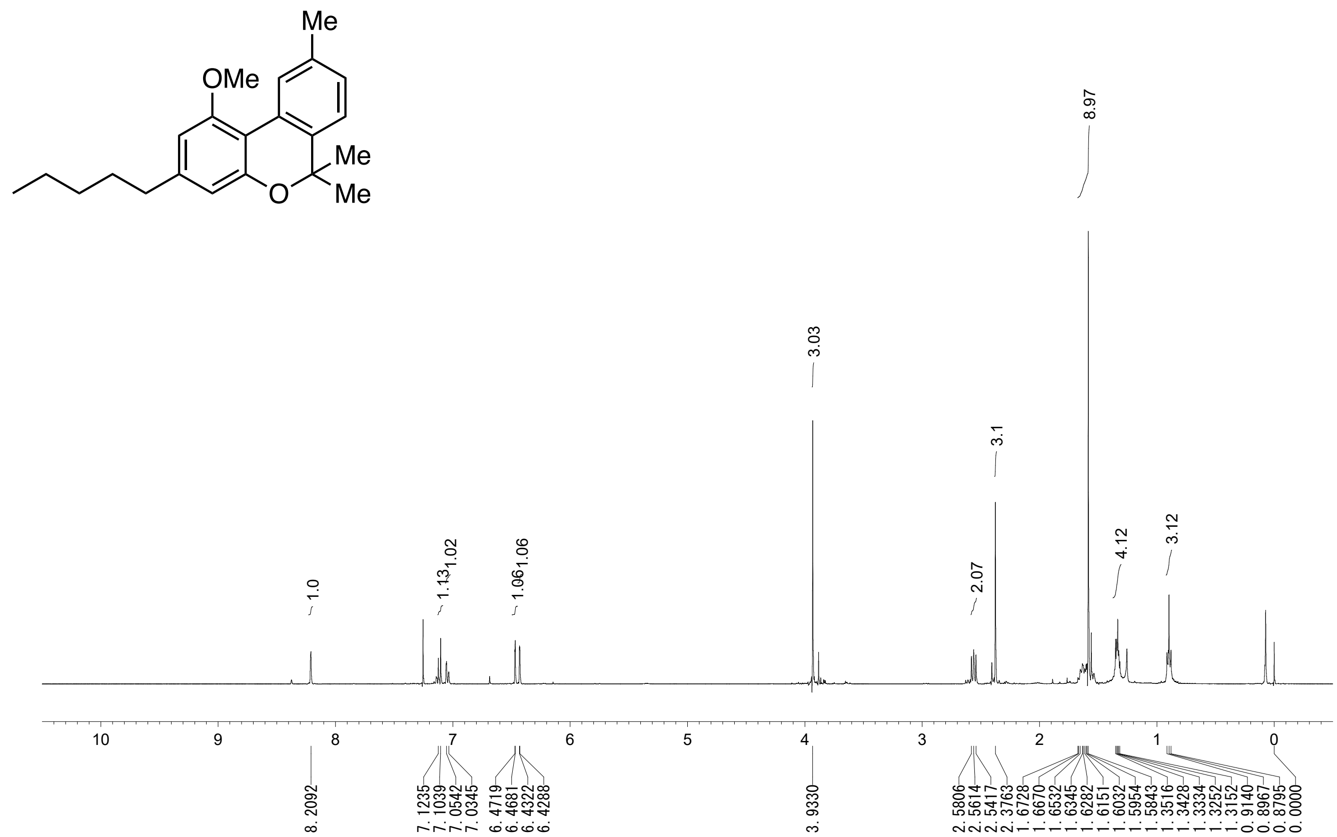

^{13}C{^{1}H} NMR (150 MHz, $CDCl_3$)

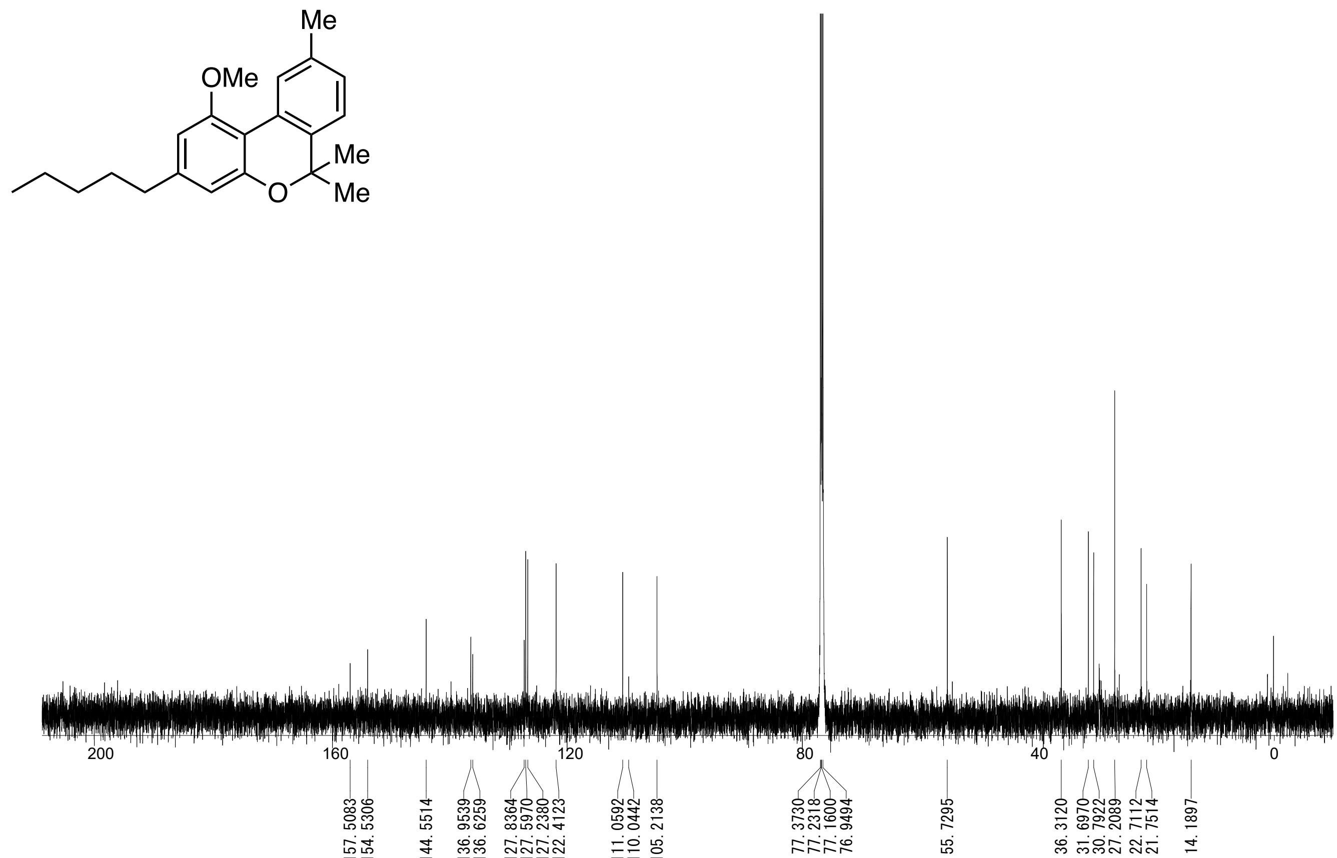

6,6,9-Trimethyl-3-pentyl-6H-benzo[c]chromen-1-ol (Cannabinol)
^{1}H NMR (400 MHz, $CDCl_3$)

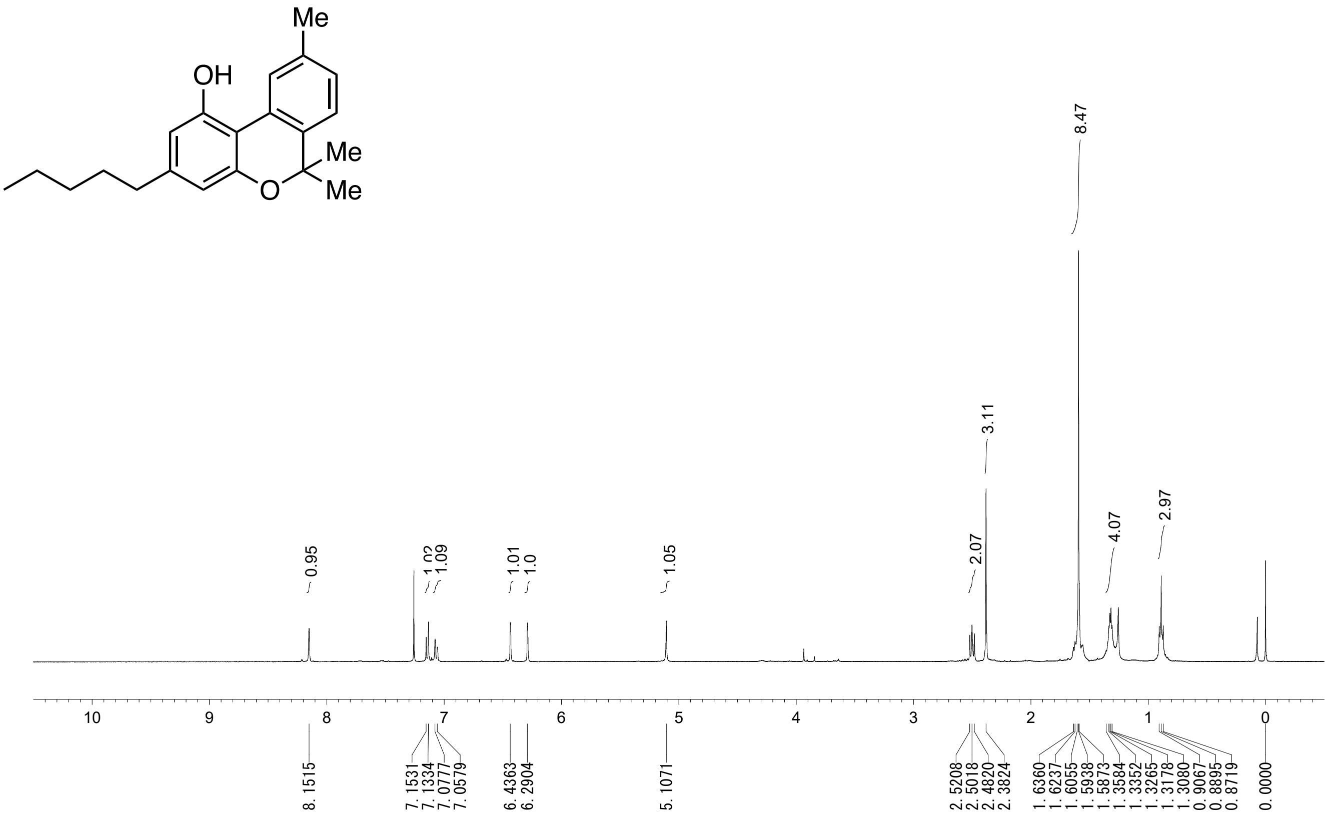

^{13}C{^{1}H} NMR (150 MHz, $CDCl_3$)

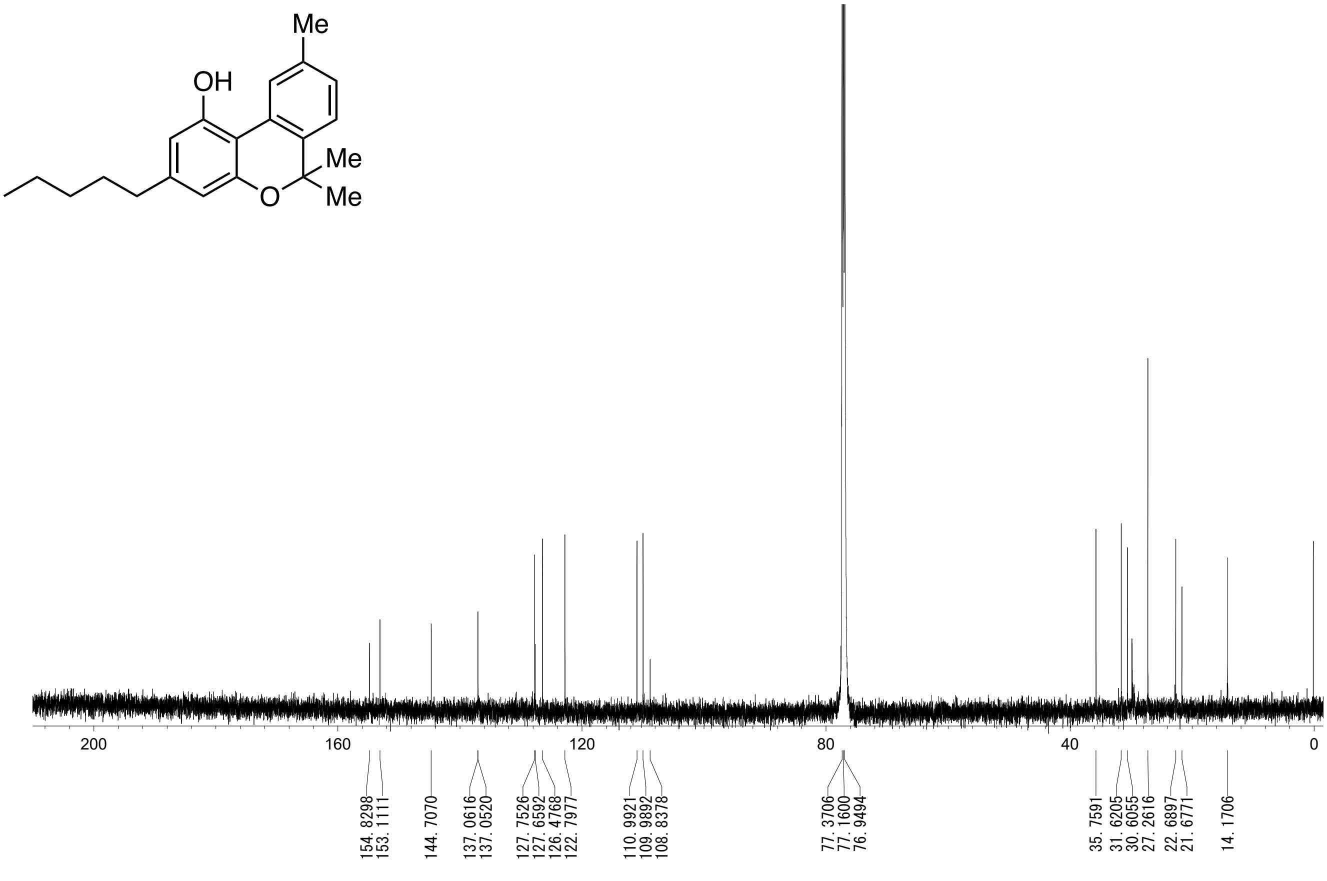

Supplement: SC-OLF-D6SC05160J-s001 [file SC-OLF-D6SC05160J-s001.pdf]
